# Transcriptome sequencing of three *Ranunculus* species (Ranunculaceae) reveals candidate genes in adaptation from terrestrial to aquatic habitats

**DOI:** 10.1038/srep10098

**Published:** 2015-05-20

**Authors:** Ling-Yun Chen, Shu-Ying Zhao, Qing-Feng Wang, Michael L. Moody

**Affiliations:** 1 Key Laboratory of Aquatic Botany and Watershed Ecology, Wuhan Botanical Garden, Chinese Academy of Sciences, Wuhan 430074, Hubei, PR China; 2 Department of Biological Sciences, University of Texas at El Paso, 500 West University Ave, El Paso, TX, 79968, USA

## Abstract

Adaptation to aquatic habitats is a formidable challenge for terrestrial angiosperms that has long intrigued scientists. As part of a suite of work to explore the molecular mechanism of adaptation to aquatic habitats, we here sequenced the transcriptome of the submerged aquatic plant *Ranunculus bungei*, and two terrestrial relatives *R. cantoniensis* and *R. brotherusii*, followed by comparative evolutionary analyses to determine candidate genes for adaption to aquatic habitats. We obtained 126,037, 140,218 and 114,753 contigs for *R. bungei*, *R. cantoniensis* and *R. brotherusii* respectively. Bidirectional Best Hit method and OrthoMCL method identified 11,362 and 8,174 1:1:1 orthologous genes (one ortholog is represented in each of the three species) respectively. Non-synonymous/synonymous (*d*_N_/*d*_S_) analyses were performed with a maximum likelihood method and an approximate method for the three species-pairs. In total, 14 genes of *R. bungei* potentially involved in the adaptive transition from terrestrial to aquatic habitats were identified. Some of the homologs to these genes in model plants are involved in vacuole protein formation, regulating ‘water transport process’ and ‘microtubule cytoskeleton organization’. Our study opens the door to understand the molecular mechanism of plant adaptation from terrestrial to aquatic habitats.

Understanding how organisms have adapted to different environments is a long-standing goal of evolutionary biology. With advancing technologies in molecular methods, identification of candidate genes for selection has advanced from examining few genes to thousands in non-model organisms^1^,^2^. RNA-Sequencing (RNA-seq) is a recently developed approach to transcriptome profiling that uses second-generation sequencing technologies. It provides unprecedented and powerful opportunities to address comparative genomic-level questions for non-model organisms^3^, besides its wide application for studying patterns of gene expression. Given the premise that positive selection is linked to potential selective pressures, the ratio of number of nonsynonymous substitutions per nonsynonymous site (*d*_N_) to number of synonymous substitutions per synonymous site (*d*_S_) is an indicator for measuring positively selected ‘candidate’ genes^4^. The *d*_N_/*d*_S_ ratio as applied to transcriptomes, has recently uncovered candidate genes related to environmental adaptation in many non-model organisms^5^,^6^,^7^.

Aquatic angiosperms constitute only 1–2% of extant angiosperms^8^, but are found in approximately 17% of angiosperm families representing about 100 evolutionarily independent origins^9^. Compared with terrestrial angiosperms, aquatic plants occupy a distinctive and in some ways more stressful ecological environment including low light levels, reduced carbon availability, sediment anoxia and mechanical damage through wave exposure^10^. Aquatic plants have adapted various life forms requiring different levels of physical change from terrestrial plants, generally being divided into emergent, floating-leaves, or submersed forms, the latter most extreme. Given these challenges and physical changes, the adaptive strategies of aquatic plants have long intrigued scientists^11^. There is now a strong understanding of the adaptive traits of aquatic angiosperms, including the reproductive systems, light requirements, phenotypic plasticity, and leaf economics, and so on.

However, there is still limited understanding of the molecular mechanisms of genetic adaptation of aquatic plants. Recent advances in aquatic plant genomics have been made including whole chloroplast genome sequencing for *Nuphar advena*^12^, *Najas flexilis*^13^, *Spirodela polyrhiza*, *Wolffiella lingulata* and *Wolffia australiana*^14^ whole mitochondria sequencing of *Butomus umbellatus*^15^ and whole genome sequencing of *Spirodela polyrhiza*^16^. The identification of genes for adaptation to aquatic life has only recently been explored, viz. Wissler *et al.*^17^ identified candidate genes for adaptation to marine life by comparing orthologous genes from two seagrasses and eight terrestrial species. However, candidate genes for adaptation in freshwater habitats have not been investigated. Contrary to the limited studies on aquatic plants, genomic approaches to better understand adaptation to different environments have been carried out in terrestrial plants^6^,^7^, and animals including cichlid fishes^18^ and dolphins^19^.

Genes associated with adaptions to aquatic environments are valuable genetic resources which could be used to enhance plant resistance to waterlogging in future. This may have particular significance given the rise in global sea levels over the past decades due to increased temperature^20^. The flood risk and wetland shifts would be aggravated by the sea-level rising, which impose waterlogging pressure to crops and natural plant communities^21^.

*Ranunculus* L. (Ranunculaceae) serves as a well-studied lineage that can be used as a genomic model for the study of plant adaptations from terrestrial to aquatic habitats. The genus is cosmopolitan with approximately 360 species (Plant List: http://www.theplantlist.org/) and recent phylogenetic hypotheses that include aquatic taxa suggest a single shift of a subclade to the aquatic habitat^22^. In the present study, three species were included. (1) *Ranunculus bungei* Steud. (also treated as *Batrachium bungei* (Steud.) L. Liou, 2n = 16 or 24 with x = 8^23^) is a typical aquatic perennial herb with submerged vegetative organs and emergent flowers (Fig. 1). The plant is distributed in the temperate to sub-boreal zones of the Northern Hemisphere, and used as an indicator of good water quality. (2) *Ranunculus cantoniensis* DC. (also treated as *Ranunculus chinensis* Bunge in China), is a terrestrial herb widely distributed in Asia (eFlora of China, http://www.efloras.org/). Its karyotype is 2n = 16, x = 8 in mainland China^24^. (3) *Ranunculus brotherusii* var. *tanguticus* (Maxim.) is also a terrestrial herb distributed in Xinjiang, Qinghai (China), Central Asia and Russia (eFlora of China), 2n = 32 with x = 8^24^. The latter two terrestrial species are used for herbal medicine in China^25^. The split between *R. bungei* and its terrestrial relatives is relatively recent, with an age of no more than 20 Ma^26^.

In the current study, we isolated transcriptomes of the aquatic species *R. bungei* and two terrestrial species *R. cantoniensis* and *R. brotherusii* using the Illumina paired-end sequencing technology in order to: (1) increase the genetic resources and obtain orthologous genes of the three species for statistical assessment in order to (2) identify candidate genes involved in the adaptive transition from terrestrial to aquatic habitat.

## Results

### Divergence time estimation

**Figure 1:**
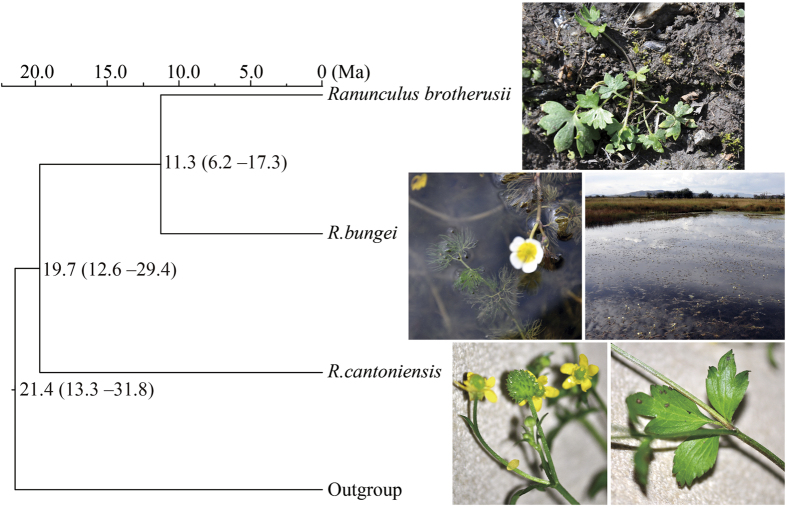
Phylogenetic relationship and photographs of *Ranunculus* species used in this study. The divergence times are given in millions of years. The plant and habitat photographs were taken by Ling-Yun Chen.

The ITS sequences obtained in this study were deposited in GenBank (no. KP336398–KP336400). Divergence time estimation using ITS suggested that *R. bungei* and *R. brotherusii* split from their shared most common ancestor at c. 11.3 (95% CI: 6.2–17.3) Ma. The two species split from the most recent shared common ancestor with *R. cantoniensis* at c. 19.7 (95% CI: 12.6–29.4) Ma (Fig. 1 & Supplementary Fig. S1).

### *De novo* assembly and annotation of unigenes

We generated 102–106 million clean reads per species, yielding c. 9.3–9.6 Gb of RNA-seq data per species (Supplementary Table S1). The clean reads were submitted to the NCBI Sequence Reads Archive (no. SRR1822558, SRR1822529, SRR1737526). *De novo* assembly yielded 114,753–140,218 contigs, with mean length at 342–357 bp and N50 at 649–727 bp. The unigenes, which were assembled by using the contigs, are 637–688 bp on average with N50 at 1,132–1,187 bp.

**Figure 2:**
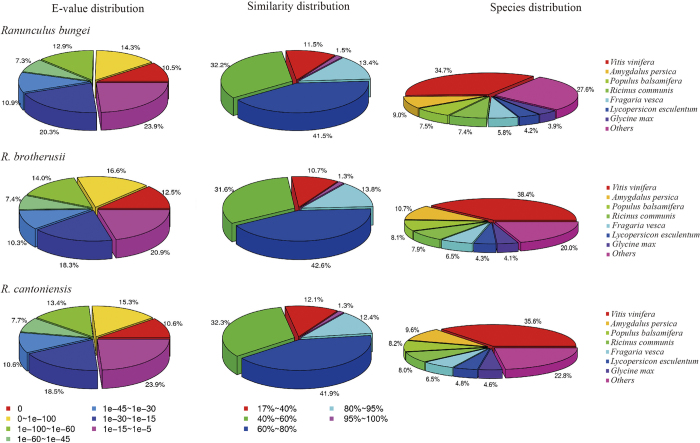
Summary of the unigenes of *R. bungei, R*. *brotherusii* and *R. cantoniensis* annotated to NCBI NR database with BLASTX.

All the unigenes were annotated on the basis of similarity to the public NCBI non-redundant protein database (NR), Swiss-Prot protein database (Swiss-Prot, http://www.expasy.ch/sprot), Kyoto Encyclopedia of Genes and Genomes (KEGG, http://www.genome.jp/kegg/), Cluster of Orthologous Groups database (COG, http://www.ncbi.nlm.nih.gov/COG/), Gene ontology database (GO) and NCBI nucleotide database (NT). The results indicated that 41,111 *R. bungei* (54%), 37,427 *R. brotherusii* (66%) and 43,565 *R. cantoniensis* (51%) unigenes have a significant match (E-value < 10^−5^) to public databases (Supplementary Table S1). NR has the highest proportion of successful annotations, while COG has the lowest proportion. The two top-hits for the three species in the NR database are *Vitis vinifera* and *Amygdalus persica* (Fig. 2). GO functional classification divided all the unigenes into three categories: cellular component, molecular function and biological process (Supplementary Fig. S2)

### Orthologous genes and *d*_N_/*d*_S_ analyses

The Bidirectional Best Hit (BBH) method^27^ with E-value < 10^−15^ recovered 11,362 putative 1:1:1 orthologous genes, while OrthoMCL^28^ recovered 8,174 putative 1:1:1 orthologous genes. The median length of orthologs (with alignment gaps) inferred from BBH and OrthoMCL is c. 930 bp and 770 bp respectively. After filtering the orthologous pairs with *d*_S_ < 0.01, *d*_S_ > 1.0, *d*_N_ **> 1.0 and pairs with aligned length < 150 bp, the two methods yielded c.11,000 and 7,600 orthologous pairs for each species pair, respectively (Table 1).

With the BBH orthologs and maximum likelihood (ML) method, the mean value of *d*_N_, *d*_S_, and *d*_N_/*d*_S_ of the three pair-wise comparisons was 0.033–0.054, 0.226–0.390, 0.150–0.160 respectively. Only 1–3 orthologous pairs with *d*_N_/*d*_S_ > 1 were found for each comparison. Taking 0.5 for *d*_N_/*d*_S_ and P < 0.05 as indicators of positive selection, 60–69 orthologous pairs were found for each comparison. In the two comparisons, viz. *R. bungei* – *R. cantoniensis* and *R. bungei* – *R. brotherusii*, 69 and 64 orthologous pairs were recovered to be positively selected respectively (Table 1, species pair 1–3).

With the OrthoMCL orthologs and approximate method, the mean value of *d*_N_, *d*_S_, and *d*_N_/*d*_S_ of the three pair-wise comparisons was 0.036 –0.059, 0.219 –0.376, 0.170 –0.184 respectively. Only 1–2 orthologous pairs with *d*_N_/*d*_S_ > 1 were found for each comparison. 48–68 orthologous pairs with *d*_N_/*d*_S_ > 0.5 (P < 0.05) were found for each comparison (Table 1, species pair 10–12). The comparison *R. bungei* – *R. cantoniensis* and *R. bungei* – *R. brotherusii* suggested 54 and 41 orthologous pairs were under positive selection respectively. More information is shown in Supplementary Table S2 and sequences for all the orthologous pairs with *d*_N_/*d*_S_ > 0.5, P-value < 0.05 are provided in Supplementary sequence data.

### Positively selected genes (PSGs) of *R. bungei*

**Figure 3:**
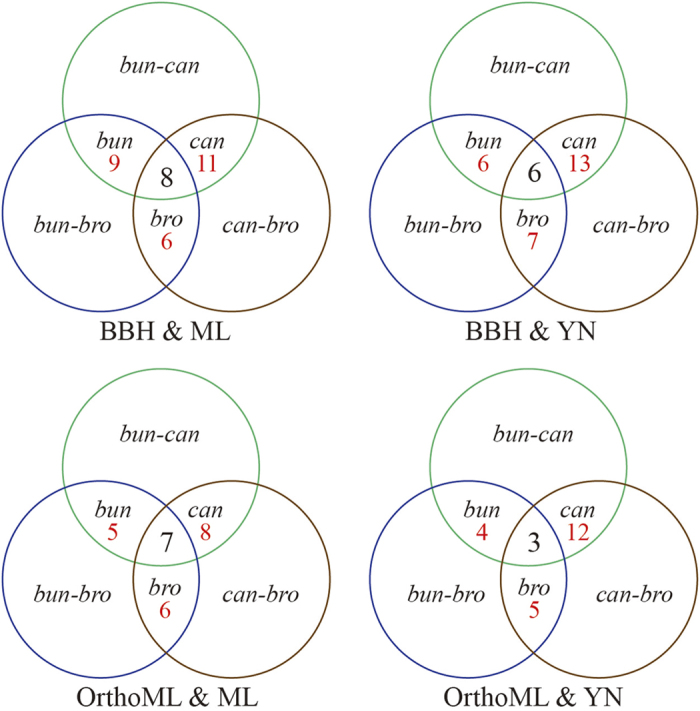
Numbers of PSGs shared among species-pairs. The numbers in red colour, for example, ‘bun 9’ indicates 9 PSGs of *R. bungei* are shared by *R. bungei–R*. *cantoniensis* and *R. bungei–R. brotherusii*, but orthologs in *R. cantoniensis*–*R. brotherusii* are not positively selected. The number in the centre of each Venn diagram indicates the clusters that are positively selected in all three species-pair comparisons. These data are counted by using Supplementary Table S2; orthologous pairs with possible alignment problems were excluded.

We counted the PSGs of *R. bungei* for which positive selection was indicated for the 2 aquatic – terrestrial comparisons but not for the terrestrial – terrestrial comparison. Nine PSGs of *R. bungei* were suggested in the BBH orthologs and ML analysis; 4–6 PSGs were recovered in the other three analyses (Fig. 3). In total, 12 PGSs were recovered, of which 2 were shared in all the analyses, viz. Unigene28957 and Unigene25660 (Table 2). In addition, 2 genes of *R. bungei*, which do not satisfy our strict criterion of PSGs, but were identified as a PSG in at least one aquatic-terrestrial comparison, might also participate in adaptation to overcome the transition into the aquatic habitats, viz. CL2856.Contig2 and Unigene36323 (Table 2).

The 3 orthologs within the cluster that includes Unigene28957 were matched to the same protein according to the TAIR 10 Transcripts database ( https://www.arabidopsis.org/) and the *Vitis vinifera* NR, supported that these were 3 orthologs and not paralogs. Similar results were found for 9 of the 13 remaining clusters (see Table 2). Species phylogeny inferred from cluster that includes Unigene28957 is congruent with that from ITS (Fig. 1). Similar results were found for 9 of the 13 remaining clusters (see Supplementary Fig. S3 for phylogenies of all the clusters).

According to TAIR 10, the best *Arabidopsis* protein match for Unigene 28957 is AT1G22060.1, a Leucine Rich Repeat domains containing protein, which is located in the vacuole and expressed during growth stages. Unigene32998 was best matched to AT1G34050.1, a member of the Ankyrin repeat family. The *Vitis vinifera* NR database also identified the gene as a member of the Ankyrin repeat family (Table 2, sequences for the 12 PSGs are provided in Supplementary sequence data).

## Discussion

The relatively high mean *d*_N_ and *d*_S_ value and low mean *d*_N_/*d*_S_ ratio obtained in the present study might be due to the relatively ancient splits among *R. bungei*, *R. cantoniensis* and *R. brotherusii* (11.3–19.7 Ma). As divergence time between two sequences increases, so too does the *d*_N_ and *d*_S_ value^29^. The orthologs of closely related species usually get low *d*_N_ and *d*_S_. For example, the mean *d*_N_ and *d*_S_ of *Primula poissonii* – *P. wilsonii* (split c. 0.9 Ma) was 0.007 and 0.027 respectively^30^, while the values of *Arabidopsis* – poplar (split c. 110 Ma) were 0.202 and 2.184^31^. The *d*_N_/*d*_S_ ratio might decrease over time^29^, e.g. the ratio for cattle – human is even lower than that in common human polymorphisms^32^. This is likely, in part, why we obtained a limited number of orthologous pairs with *d*_N_/*d*_S_ > 1 and P < 0.05. However, the split between *R. bungei* and its terrestrial relatives might be the most recent split between terrestrial life form and submersed aquatic life form among aquatic angiosperms. It was assessed by using Angiosperm Phylogeny Website ( http://www.mobot.org/MOBOT/research/APweb/), TimeTree ( http://www.timetree.org/) and an extensive literature search. For example, a comparable split within the Eudicots between terrestrial *Haloragis*/*Gonocarpus* and aquatic *Myriophyllum*/*Laurembergia* (Haloragaceae) occurred c. 35 Ma^33^. Whereas several deeper splits of well-known aquatic lineages were much older including the submersed *Ceratophyllum* and its terrestrial relative, (c. 148 Ma^34^), and the split between the submersed Alismatale (e.g. *Potamogeton*) and their terrestrial relatives (c. 124 Ma^35^). Therefore, the recency of the split between *R. bungei* and its two relatives makes this a good model system for identifying candidate genes for the adaptation to aquatic habitat.

In the present study, each pair-wise comparison identified more than 40 orthologous pairs that were positively selected. For example, 69 pairs for *R. bungei* – *R. cantoniensis* were recovered by the BBH and ML method. However, only the orthologs of *R. bungei* that satisfied the criterion of positive selection for the two aquatic – terrestrial comparisons (*R. bungei* – *R. brotherusii* and *R. bungei* – *R. cantoniensis*), but did not show positive selection for the terrestrial – terrestrial comparison (*R. cantoniensis* – *R. brotherusii*) were identified as being involved in adaptation to the aquatic habitat. Compared with some previous studies^3^,^30^, which only use one species pair to explore candidate genes for adaptive evolution, our study provides a more conservative approach to optimize the reliability of the methodology.

Aquatic habitats can be characterized by low carbon, oxygen, shaded conditions, sediment anoxia, wave exposure, sometimes also osmotic stress and limited nutrient supply^10^, which makes re-colonization of aquatic habitats by terrestrial angiosperms a challenge^13^. Plant ethylene, ROS (reactive oxygen species), low NO and O_2_ are important signals and/or regulators for water adaptation^36^. Some plants can adapt strategies to temporarily avoid or reduce problems associated with submergence^36^. (1) An ‘escape’ strategy whereby elongation of organs above floodwaters or (2) a quiescent strategy whereby the plant limits carbohydrate consumption and growth, and protects the meristem organ. Some of the genes that regulate these strategies have been determined and including *SUB1A*, *SNORKEL1*, *SNORKEL2* in rice^37^, *HRE1*, *HRE2* in *Arabidopsis thaliana*. Submerged aquatic plants usually have adaptions such as degraded cell walls in xylem and roots, well-developed aerenchyma and hydathodes^38^, which can all be found for *R. bungei*^39^.

The model terrestrial/amphibious plant such as rice and *A. thaliana* don’t possess most of these characters. Aquatic plants and terrestrial plants likely have some common mechanisms of water adaptation, but the aquatic plants also have some distinct mechanisms.

Among the 14 genes of *R. bungei*, which are identified as PSGs in adaptation to aquatic habitat, gene ontology identified Unigene28957 codes for an expressed protein located in the vacuole. The vacuole has an important role in regulating osmotic pressure (e.g. by accumulating proteins^40^), and regulation function can be extreme for freshwater aquatic plants^41^. Thus, Unigene28957 might participate in this osmotic regulation. This was supported by the result that in the terrestrial – terrestrial comparison no orthologous pairs to the vacuole were identified to be under positive selection. CL2856.Contig2 was defined as part of a solute carrier family 50 (sugar transporter), which regulates water transport, response to fructose stimulus, sugar transmembrane transporter activity, and root development^42^. This gene, thus could play a role in regulating osmotic stress and/or directly affect the development of roots of these aquatic plants (Table 2). Unigene36323, a putative DNA repair protein RAD50, which participates in microtubule cytoskeleton organization, mitotic recombination^43^, vernalization response and seed germination^44^, could be active in structural modifications (e.g. the cell wall) in changes from *R*. *bungei*’s terrestrial relatives.

The other 11 PSGs were identified as members of the Haloacid dehalogenase-like hydrolase (HAD) superfamily, Ankyrin repeat family, DUF724 protein family, etc. (see Table 2). Function information of these genes is very limited, so their direct relation to aquatic adaptation can not yet be determined.

Some genes that are postulated to be involved in re-colonization of aquatic habitats, such as the homologous gene of *LESION SIMULATING DISEASE1* that control the formation of Lysigenous Aerenchyma in *Arabidopsis*^45^, the homologous gene of *VACUOLELESS1* that is an essential gene for vacuole formation in *Arabidopsis*^46^ and the homologous gene of group VII Ethylene Response Factor, were not recovered as under positive selection in this study. In the molecular adaptation study of seagrasses^17^, 51 genes were identified to be under positive selection. Most of them were involved in translation, metabolism and photosynthesis such as the genes for utilizing CO_2_ and light. Some of the PSGs of *R. bungei* in the present study are involved in translation and metabolism. None are involved in photosynthesis, although submersed aquatic plants usually live in low dissolved oxygen and low light level environment.

Of course, there are still limitations in our ability to crossover with results from other studies. (1) Although we have more than 9 Gb of clean data for each of the three *Ranunculus* species, it likely provides only limited coverage of the genome. Similarly, genes that are known to facilitate salt tolerance such as the SOS were absent from investigation in the comparative genomic analysis of seagrasses^17^. (2) The pair-wise comparison method estimated the *d*_N_/*d*_S_ between two sequences; genes experienced strong positive selection at some nucleotide sites but with low average *d*_N_/*d*_S_ ratio may be neglected^47^, thus genes of importance may not always be recognized. (3) We are also limited in recognizing all genes that relate to specific function for aquatic adaptations, as some orthologs can’t be annotated by using the public databases, such as the NR and GO. This is a problem even for plants with whole genome sequencing completed, e.g. *Populus*^3^.

## Conclusions

In this study, we obtained transcriptomes of three *Ranunculus* species, and carried out statistical assessment of non-synonymous and synonymous substitution rates with pair-wise comparisons. In total, we detected 14 candidate genes that may be involved in the adaptation from terrestrial habitats to aquatic habitats. As this study did not have complete transcriptome coverage for these three species, our ability to identify genes involved in recolonization of aquatic habitats by angiosperms will benefit from analyzing more genomic data. Also, including more aquatic plant lineages and their terrestrial relatives, especially lineages with higher levels of genome annotations already available, for comparative analyses will be necessary to identify candidate genes important for aquatic adaptation in plants. The ultimate goal will be to verify the function of these candidate genes, and studies such as this provide a starting point for investigation. These 14 candidate genes provide a valuable resource to begin to understand the molecular mechanism of plant adaptation from terrestrial to aquatic habitats.

## Methods

### Plant material

*Ranunculus bungei* (36°56'56.60“N, 100°53'09.92“E; 3096 m alt.) and *R. brotherusii* (37°11'57.42“N, 101°32'18.33“E; 2820 m alt.) were sampled from Qinghai province, China in Sep. 5, 2013. *Ranunculus cantoniensis* was sampled from MangShan National Forest Park (24°58'57.67“N, 112°53'11.09“E; 770 m alt.), Hunan province, China in Oct. 20, 2013. Living plants of the three species were brought to the greenhouse in Wuhan Botanical Garden for cultivation.

### Phylogenetic inferences

The phylogenetic relationship and divergence time among *R. bungei*, *R. brotherusii* and *R. cantoniensis* were estimated including these taxa within a larger *Ranunculus* dataset. Genomic DNA of the three species was isolated from fresh leaves using an Ezup Column Plant Genomic DNA Purification Kit (Sangon Biotech, Shanghai, China). The internal transcribed spacer regions (ITS1, ITS2) and 5.8S gene of the nuclear-encoded ribosomal DNA were amplified following Chen *et al.*^48^. Sequencing using the PCR primers was carried out on an ABI 3730 automated sequencer at Tsingke Biotech Co. (Beijing, China). An ITS data matrix was created with the 3 sequences we generated and 81 *Ranunculus* sequences from GenBank (accession number were provided in Supplementary Fig. S1). 16 species represent 14 genera such as *Krapfia clypeata* and *Laccopetalum giganteum* were selected as outgroups following Emadzade & Horandl^26^. We then used BEAST v. 1.7.5^49^ with four independent Monte Carlo Markov Chains (MCMC) runs for 15 million generations, sampling every 10,000 generations. The first 10% of trees were discarded as burn-in, and the remaining trees were combined. We applied a lognormal relaxed clock and calibrated the phylogeny with two fossil calibration points according to Emadzade & Horandl^26^, one is the split between *Ranunculus* and *Clematis*, and one is the minimum age of *Myosurus*.

### RNA extraction and sequencing

A mix of tissues from leaves, stems and roots were collected at 12 am and 12 pm. One individual for each species was sampled, as the intra-species variation is low compared with the inter-species variation^50^and life-form of the three species is stable. Total RNA was isolated using RNAiso^TM^ Plus (Takara, Qingdao, China) and then treated with RNase-free DNase I (Takara, Qingdao, China) for 45 min according to the manufacturer’s protocols. The quality of total RNA was checked using 2% agarose gel electrophoresis. The RNA samples were then delivered to Beijing Genomics Institute (BGI, Shenzhen, China), and concentrations were checked by Agilent Technologies 2100 Bioanalyzer instrument (Agilent Technologies, Santa Clara CA, USA). The cDNA preparation and Illumina sequencing were performed at BGI. The entire process followed a standardized procedure monitored by BGI’s Quality Control System. The mRNA was isolated from total RNA using oligo (dT) magnetic beads using the manufacturer’s instructions for cDNA library construction. Double stranded cDNA was sequenced using the Illumina HiSeq™ 2000 sequencer (90 bp paired-end). Image data from the sequencer was transformed by base calling into raw sequence data, which formed the raw reads.

### *De novo* assembly and annotation

Raw reads were cleaned by removing adaptor sequences, reads with unknown base calls (N) more than 5%, and low quality reads (>20% of the bases with a quality score ≤10) using Filter_fq (an internal program of BGI). *De novo* assembly was carried out with the short reads assembling program Trinity v. 20130225^51^ using default parameters except for the following: mini contig length 100 bp, min glue 3, group pairs distance 250, path reinforcement distance 85, and min kmer cov 3. Contigs were assembled by Trinity into unigenes using pair-end information. The unigenes were then processed by the TGI Clustering Tool (TGICL) v. 2.1^52^ to remove redundancies, and assembled to acquire non-redundant unigenes as long as possible. We changed the default parameters of TGICL to put together sequences linked to other sequences by overlaps of at least 40 bp, and at most 20 bp overlap distance of sequence ends.

In order to get descriptive annotation, all of the unigenes were annotated based on similarity to the NR, Swiss-Prot, KEGG, and COG by BLASTX (E-value < 10^−5^). The unigenes were also annotated to NT by BLASTN (E-value < 10^−5^). With the results of NR annotation, Blast2GO^53^ was used to get Gene Ontology functional annotation. After that, WEGO^54^ was used to determine functional classification for all unigenes and to understand the distribution of gene functions for the species from the macro level.

Sequence direction of the unigenes was determined using the best aligning results between the unigenes and the protein databases. Incongruent results from different databases were settled by a priority order of NR, Swiss-Prot, KEGG and COG. Coding region sequences (CDS) of the unigenes were predicted by firstly aligning unigenes to NR, then Swiss-Prot, then KEGG and finally COG with BLASTX. Unigenes aligned to a higher priority database were not aligned to a lower priority database. The CDS were then translated to amino acid sequences with standard genetic coding using custom Perl scripts.

### Identification of orthologous genes

Identification of orthologous genes is critical to this study. To find clusters of orthologs among the three species, we adopted two strategies. Firstly, we applied a BBH method^27^, and ‘stringent filters’ to exclude paralogs^6^. The predicted amino acid sequences of each species were used as queries and targets separately to search against those of the other two species (BLASTP). The best hits of the longest isoforms with E-value <10^–6^ or 10^–15^ were retrieved. Orthologous pairs with identity <60 were excluded and only 1:1:1 orthologous genes in all three species were retained. BLASTP with E-value <10^–15^ reduced the number of the putative 1:1:1 orthologous genes by only 2%. The clusters that contained two or more unigenes from the same species were excluded from further analyses to eliminate potential paralogs. Clusters that included stop-codons were also excluded from further analyses.

Secondly, orthologous gene clusters were constructed using OrthoMCL^28^ with default settings according to methods in Wissler *et al.*^17^. Only clusters with at least one sequence per species were used in our analyses^17^. If more than one sequence of any species was contained in a cluster, all sequences of that species were removed except for the one sequence that showed the highest similarity to all other sequences of the cluster. The putative orthologous pairs were then aligned by MUSCLE^55^ with default parameters.

### *d*_N_/*d*_S_ analyses

The *d*_N_, *d*_S_ value and *d*_N_/*d*_S_ ratio were estimated in KaKs_Calculator v. 1.2^56^ using two methods, a ML method with model averaging^57^ and an approximate method with the YN model^47^. Both methods were applied to the BBH orthologs and OrthoMCL orthologs (Table 1). The proportion of synonymous and nonsynonymous substitution sites and maximum-likelihood score for each ortholog pair were calculated. Fisher’s exact test was performed to justify the validity of the *d*_N_ and *d*_S_ values. Three independent *d*_N_/*d*_S_ analyses were performed for each orthologous cluster, viz. (1) aquatic *R. bungei* – terrestrial *R. brotherusii*; (2) *R. bungei* – terrestrial *R. cantoniensis*; (3) *R. brotherusii* – *R. cantoniensis* (Table 1). Ortholog pairs with *d*_N_ > 1.0, saturated for synonymous substitutions (*d*_*S*_ > 1.0) an*d* with *d*_S_ < 0.01 were removed. In addition, ortholog pairs with P-value (Fisher test) > 0.05 or aligned length < 150 bp were also excluded. *d*_N_/*d*_S_ > 0.5 was applied as a threshold of positively selecte*d* genes. Swanson *et al.*^58^ estimated that more than 80% of genes with *d*_N_/*d*_S_ > 0.5 were under selection and the threshold value of *d*_N_/*d*_S_ > 0.5 has become widely accepted in recent studies^3^,^30^.

One additional filter was used to exclude paralogs: the aligned DNA sequences of candidate PSG pairs were checked manually to eliminate results due to poor alignment. We counted the number of ortholog pairs under positive selection between two species and the clusters under positive selection in all three species-pair comparisons. At last, we accepted the PSGs of *R. bungei*, which were identified to be under positive selection for both the aquatic *R. bungei* – terrestrial *R. brotherusii* and *R. bungei* – terrestrial *R. cantoniensis* comparisons (*d*_N_/*d*_S_ > 0.5, P < 0.05), but not for the terrestrial *R. brotherusii* – *R. cantoniensis* comparison (*d*_N_/*d*_S_ < 0.5 or P > 0.05). We think this conservative method can exclude pseudo-PSGs of *R. bungei*.

### Annotation and phylogenetic inference of the PSGs of *R. bungei*

In order to get more detailed annotation, orthologs within the clusters that include the 14 PSGs of *R. bungei* from the last step were annotated to TAIR10 using BLASTP. The orthologs were also annotated to the NCBI NR database of *Vitis vinifera* using BLASTP, as more than 30% of our unigenes were matched to the species (Fig. 2). In total, 14 clusters were annotated.

Phylogenetic relationships among the 14 PSGs of *R. bungei* and their orthologs in the two other species were estimated using protein sequences. According to results of BLAST to TAIR10 and NR database of the last step, the sequence hit with lowest E-value was used as outgroup for each cluster. Each cluster was aligned by MUSCLE^55^ with default parameters, and ambiguous alignment were manually deleted. ML and maximum parsimony (MP) analyses were performed using MEGA v.6.0.6^59^. ML was performed with Jones-Taylor-Thornton (JTT) model and Gamma Distributed (G); MP was performed with Subtree-Pruning-Regrafting (SPR) search method. Gaps and missing characters were complete deleted and branch support values were estimated by 100 bootstrap replicates. All other parameters were default values.

## Author Contributions

L.Y.C. and Q.F.W. conceived this study; S.Y.Z. and L.Y.C. carried out field work, laboratory work and data analyses; S.Y.Z. and L.Y.C. prepared the tables and figures; L.Y.C., S.Y.Z. and M.L.M. drafted the main manuscript; Q.F.W. polished the manuscript.

## Additional Information

**How to cite this article**: Chen, L.-Y. *et al*. Transcriptome sequencing of three *Ranunculus* species (Ranunculaceae) reveals candidate genes in adaptation from terrestrial to aquatic habitats. *Sci. Rep.* *5*, 10098; doi: 10.1038/srep10098 (2015).

## Supplementary Material

Supplementary Information

Supplementary Dataset

Supplementary Sequence Data

## Figures and Tables

**Table 1:** Summary of the Orthologous genes and *d*_N_/*d*_S_ analyses.

**Species pairs**	**Ortholog data set &** ***d***_**N**_**/*****d***_**S**_ **estimation**	**No. of ortholog pairs after filter**^a^	**Mean value**^b^			**No. of ortholog pairs**^c^ (*d*_**N**_**/*****d***_**S**_ **>0.5, P <0.05)**	**Mean value**^c^		
			***d***_**N**_	***d***_**S**_	***d***_**N/**_***d***_**S**_		***d***_**N**_	***d***_**S**_	***d***_**N/**_***d***_**S**_
1. *bun*−*can*	BBH & ML	10990 (944)	0.054	0.39	0.15	69 (735)	0.152	0.273	0.557
2. *bun*−*bro*		11162 (950)	0.033	0.226	0.16	64 (959)	0.094	0.172	0.543
3. *can*−*bro*		11977 (938)	0.051	0.369	0.15	60 (719)	0.143	0.258	0.553
4. *bun*–*can*	BBH & YN	10852 (950)	0.054	0.369	0.159	62 (830)	0.139	0.256	0.546
5. *bun*–*bro*		10973 (959)	0.033	0.213	0.171	62 (1144)	0.08	0.148	0.538
6. *can*–*bro*		10843 (943)	0.05	0.351	0.158	54 (847)	0.137	0.246	0.56
7. *bun*–*can*	OrthoMCL & ML	7600 (807)	0.059	0.398	0.161	67 (587)	0.183	0.331	0.555
8. *bun*–*bro*		7793 (803)	0.036	0.233	0.171	48 (874)	0.115	0.201	0.632
9. *can*–*bro*		7566 (798)	0.055	0.377	0.16	56 (584)	0.163	0.297	0.551
10. *bun*–*can*	OrthoMCL & YN	7523 (809)	0.059	0.376	0.172	54 (710)	0.148	0.269	0.549
11. *bun*–*bro*		7632 (812)	0.036	0.219	0.184	41 (1066)	0.107	0.189	0.64
12. *can*–*bro*		7496 (801)	0.055	0.359	0.17	50 (723)	0.163	0.287	0.601

*Bun = R. bungei*, *can = R. cantoniensis*, *bro = R. brotherusii*. Numbers before species pairs correspond to the sheet numbers in Table S2.

ML = maximum likelihood method with model averaging; YN = approximate method with YN model

^a^Filter = removing orthologous pairs with *d*_S_ < 0.01, *d*_S_ > 1.0, *d*_N_ > 1.0, aligned length < 150 bp.

^b^estimated using orthologous pairs after filter.

^c^estimated using the orthologous pairs with *d*_N_/*d*_S_ > 0.5 and P < 0.05, orthologous pairs with possible alignment problems were excluded. Numbers in brackets indicate mean length of orthologs (including alignment gaps).

**Table 2:** Genes of *R. bungei* recognized as candidates for adaptation to aquatic habitats. The genes are identified as PSGs in comparison to terrestrial taxa.

## References

[b1] Sapir Y., Moody M. L., Brouillette L. C., Donovan L. A. & Rieseberg L. H. Patterns of genetic diversity and candidate genes for ecological divergence in a homoploid hybrid sunflower, Helianthus anomalus. Mol. Ecol. 16, 5017–5029 (2007). 10.1111/j.1365-294X.2007.03557.x PMC2467390 17944850

[b2] Narum S. R., Buerkle A., Davey J. W., Miller M. R. & Hohenlohe P. A. Genotyping-by-sequencing in ecological and conservation genomics. Mol. Ecol. 22, 2841–2847 (2013). 23711105 10.1111/mec.12350 PMC3935057

[b3] Zhang J., Xie P., Lascoux M., Meagher T. R. & Liu J. Rapidly evolving genes and stress adaptation of two desert poplars, *Populus euphratica* and *P. pruinosa*. PLoS One 8, e66370 (2013). 23776666 10.1371/journal.pone.0066370 PMC3679102

[b4] Yang Z. Likelihood ratio tests for detecting positive selection and application to primate lysozyme evolution. Mol. Biol. Evol. 15, 568–573 (1998). 9580986 10.1093/oxfordjournals.molbev.a025957

[b5] Hodgins K. A. *et al.* Comparative genomics in the Asteraceae reveals little evidence for parallel evolutionary change in invasive taxa. Mol. Ecol., 24, 2226–2240 (2015). 25439241 10.1111/mec.13026

[b6] Vigeland M. D. *et al.* Evidence for adaptive evolution of low-temperature stress response genes in a Pooideae grass ancestor. New Phytol. 199, 1060–1068 (2013). 23701123 10.1111/nph.12337 PMC3840698

[b7] Koenig D. *et al.* Comparative transcriptomics reveals patterns of selection in domesticated and wild tomato. Proc. Natl. Acad. Sci. USA 110, 2655–2662 (2013). 10.1073/pnas.1309606110 PMC3710864 23803858

[b8] Cook C. D. K. Aquatic plant book, SPB Academic Publishing: Hague, 1990).

[b9] Les D. H. C., Cleland M. A. & Waycott M. Phylogenetic studies in Alismatidae. II. Evolution of marine angiosperms (seagrasses) and hydrophily. Syst. Bot. 22, 443–464 (1997).

[b10] Santamaria L. Why are most aquatic plants widely distributed? Dispersal, clonal growth and small-scale heterogeneity in a stressful environment. Acta. Oecol. Int. J. Ecol. 23, 137–154 (2002).

[b11] Darwin C. On the origin of species John Murray: London, 1859).

[b12] Raubeson L. A. *et al.* Comparative chloroplast genomics: analyses including new sequences from the angiosperms *Nuphar advena* and *Ranunculus macranthus*. BMC Genomics. 8, e174 (2007). 10.1186/1471-2164-8-174 PMC1925096 17573971

[b13] Peredo E. L., King U. M. & Les D. H. The plastid genome of *Najas flexilis*: adaptation to submersed environments is accompanied by the complete loss of the NDH complex in an aquatic angiosperm. PLoS One 8, e68591 (2013). 23861923 10.1371/journal.pone.0068591 PMC3701688

[b14] Wang W. Q. & Messing J. High-throughput sequencing of three Lemnoideae (Duckweeds) chloroplast genomes from total DNA. PLoS One 6, e24670 (2011). 21931804 10.1371/journal.pone.0024670 PMC3170387

[b15] Cuenca A., Petersen G. & Seberg O. The complete sequence of the mitochondrial genome of *Butomus umbellatus* - a member of an early branching lineage of monocotyledons. PLoS One 8, e61552 (2013). 23637852 10.1371/journal.pone.0061552 PMC3634813

[b16] Wang W. *et al.* The *Spirodela polyrhiza* genome reveals insights into its neotenous reduction fast growth and aquatic lifestyle. Nat. Commun. 5, e3311 (2014). 10.1038/ncomms4311 PMC3948053 24548928

[b17] Wissler L. *et al.* Back to the sea twice: identifying candidate plant genes for molecular evolution to marine life. BMC Evol. Biol. 11, e8 (2011). 10.1186/1471-2148-11-8 PMC3033329 21226908

[b18] Fan S., Elmer K. R. & Meyer A. Genomics of adaptation and speciation in cichlid fishes: recent advances and analyses in African and Neotropical lineages. Philos. T. R. Soc. B. 367, 385–394 (2012). 10.1098/rstb.2011.0247 PMC3233715 22201168

[b19] Sun Y. B. *et al.* Genome-wide scans for candidate genes involved in the aquatic adaptation of Dolphins. Genome Biol. Evo. 5, 130–139 (2013). 10.1093/gbe/evs123 PMC3595024 23246795

[b20] Vermeer M., & Rahmstorf S. Global sea level linked to global temperature. Proc. Natl. Acad. Sci. USA 106, 21527–21532 (2009). 19995972 10.1073/pnas.0907765106 PMC2789754

[b21] Jackson M. B., Ishizawa K. & Ito O. Evolution and mechanisms of plant tolerance to flooding stress. Ann. Bot. 103, 137–42 (2009). 19145714 10.1093/aob/mcn242 PMC2707321

[b22] Hörandl E. & Emadzade K. Evolutionary classification: A case study on the diverse plant genus *Ranunculus* L. (Ranunculaceae). Perspect. Plant. Ecol. Evol. Syst. 14, 310–324 (2012).

[b23] Yang Q. E. Cytology of eleven species in the genus *Ranunculus* L. and five in its four related genera from China. Acta. Phytotaxon. Sin. 39, 405–422 (2000).

[b24] Fujishima H. Karyotypes in four species of *Ranunculus* (Ranunculaceae): Karyotypic variation in Mainland China and the Japanese Archipelago. Chromosome Bot. 6, 97–106 (2011).

[b25] Zhong Y. M. & Feng Y. F. Advances in studies on flavonoids and lactones in plants of *Ranunculus* Linn. Chin. Trad. Herb. Drugs 42, 825–828 (1964).

[b26] Emadzade K. & Horandl E. Northern Hemisphere origin, transoceanic dispersal, and diversification of Ranunculeae DC. (Ranunculaceae) in the Cenozoic. J. Biogeogr. 38, 517–530 (2011).

[b27] Overbeek R., Fonstein M., D’Souza M., Pusch G. D. & Maltsev N. The use of gene clusters to infer functional coupling. Proc. Natl. Acad. Sci. USA 96, 2896–2901 (1999). 10077608 10.1073/pnas.96.6.2896 PMC15866

[b28] Li L., Stoeckert C. J. Jr. & Roos D. S. OrthoMCL: identification of ortholog groups for eukaryotic genomes. Genome Res. 13, 2178–89 (2003). 12952885 10.1101/gr.1224503 PMC403725

[b29] Rocha E. P. *et al.* Comparisons of *d*_N_/*d*_S_ are time dependent for closely related bacterial genomes. J. Theor. Biol. 239, 226–235 (2006). 16239014 10.1016/j.jtbi.2005.08.037

[b30] Zhang L., Yan H. F., Wu W., Yu H. & Ge X. J. Comparative transcriptome analysis and marker development of two closely related Primrose species (*Primula poissonii* and *Primula wilsonii*). BMC Genomics 14, e329 (2013). 10.1186/1471-2164-14-329 PMC3658987 23672467

[b31] Buschiazzo E., Ritland C., Bohlmann J. & Ritland K. Slow but not low: genomic comparisons reveal slower evolutionary rate and higher *d*_N_/*d*_S_ in conifers compared to angiosperms. BMC Evol. Biol. 12, e8 (2012). 10.1186/1471-2148-12-8 PMC3328258 22264329

[b32] MacEachern S. *et al.* Testing the neutral theory of molecular evolution using genomic data: a comparison of the human and bovine transcriptome. Genet. Sel. Evol. 38, 1–22 (2006). 10.1186/1297-9686-38-3-321 PMC2689288 16635453

[b33] Chen L. Y. *et al.* Historical biogeography of Haloragaceae: An out-of-Australia hypothesis with multiple intercontinental dispersals. Mol. Phylogenet. Evol. 78, 87–95 (2014). 24841538 10.1016/j.ympev.2014.04.030

[b34] Zeng L. *et al.* Resolution of deep angiosperm phylogeny using conserved nuclear genes and estimates of early divergence times. Nat. Commun. 5, e4956 (2014). 10.1038/ncomms5956 PMC4200517 25249442

[b35] Janssen T. & Bremer K. The age of major monocot groups inferred from 800+*rbc*L sequences. Bot. J. Linnean. Soc. 146, 385–398 (2004).

[b36] van Veen H. *et al.* Two *Rumex* species from contrasting hydrological niches regulate flooding tolerance through distinct mechanisms. Plant Cell 25, 4691–4707 (2013). 24285788 10.1105/tpc.113.119016 PMC3875744

[b37] Hattori Y. *et al.* The ethylene response factors *SNORKEL1* and *SNORKEL2* allow rice to adapt to deep water. Nature 460, 1026–1030 (2009). 19693083 10.1038/nature08258

[b38] Mortlock C. The Structure and Development of the Hydathodes of *Ranunculus fluitans* Lam. New Phytol. 51, 129–138 (1952).

[b39] Cheng X. Y., Liu M., Zhang X. X., Wang C. & Li B. S. Vegetative organ structures of Ranunculaceae in Northeastern China and notes on systematic implications. Acta Prat. Sin. 62–74 (2014).

[b40] Carter C. *et al.* The vegetative vacuole proteome of *Arabidopsis thaliana* reveals predicted and unexpected proteins. Plant Cell 16, 3285–3303 (2004). 15539469 10.1105/tpc.104.027078 PMC535874

[b41] Touchette B. W., Marcus S. E. & Adams E. C. Bulk elastic moduli and solute potentials in leaves of freshwater, coastal and marine hydrophytes. Are marine plants more rigid? AoB. Plants. 6, plu014 (2014). 10.1093/aobpla/plu014 PMC4025192 24876296

[b42] Williams L. E., Lemoine R. & Sauer N. Sugar transporters in higher plants – a diversity of roles and complex regulation. Trends Plant Sci. 5, 283–290 (2000). 10871900 10.1016/s1360-1385(00)01681-2

[b43] Bleuyard J. Y., Gallego M. E. & White C. I. Meiotic defects in the *Arabidopsis rad50* mutant point to conservation of the MRX complex function in early stages of meiotic recombination. Chromosoma 113, 197–203 (2004). 15309561 10.1007/s00412-004-0309-1

[b44] Gallego M. E. *et al.* Disruption of *the Arabidopsis RAD50* gene leads to plant sterility and MMS sensitivity. Plant J. 25, 31–41 (2001). 11169180 10.1046/j.1365-313x.2001.00928.x

[b45] Muhlenbock P., Plaszczyca M., Mellerowicz E. & Karpinski S. Lysigenous aerenchyma formation in *Arabidopsis* is controlled by *LESION SIMULATING DISEASE1*. Plant Cell 19, 3819–3830 (2007). 18055613 10.1105/tpc.106.048843 PMC2174864

[b46] Rojo E., Gillmor C. S., Kovaleva V., Somerville C. R. & Raikhel N. V. *VACUOLELESS1* is an essential gene required for vacuole formation and morphogenesis in *Arabidopsis*. Dev. Cell 1, 303–310 (2001). 11702788 10.1016/s1534-5807(01)00024-7

[b47] Yang Z. & Nielsen R. Estimating synonymous and nonsynonymous substitution rates under realistic evolutionary models. Mol. Biol. Evol. 17, 32–43 (2000). 10666704 10.1093/oxfordjournals.molbev.a026236

[b48] Chen L. Y., Chen J. M., Rorbert G. W., Temam T. D. & Wang Q. F. Generic phylogeny and historical biogeography of Alismataceae, inferred from multiple DNA sequences. Mol. Phylogenet. Evol. 63, 407–416 (2012). 22327014 10.1016/j.ympev.2012.01.016

[b49] Drummond A. J. & Rambaut A. BEAST: Bayesian evolutionary analysis by sampling trees. BMC Evol. Biol. 7, e214 (2007). 10.1186/1471-2148-7-214 PMC2247476 17996036

[b50] Chen J. M., Du Z. Y., Yuan Y. Y. & Wang Q. F. Phylogeography of an alpine aquatic herb *Ranunculus bungei* (Ranunculaceae) on the Qinghai–Tibet Plateau. J. Syst. Evol. 52, 313–325 (2014).

[b51] Grabherr M. G. *et al.* Full-length transcriptome assembly from RNA-Seq data without a reference genome. Nat. Biotechnol. 29, 644–52 (2011). 21572440 10.1038/nbt.1883 PMC3571712

[b52] Pertea G. *et al.* TIGR Gene Indices clustering tools (TGICL): a software system for fast clustering of large EST datasets. Bioinformatics 19, 651–652 (2003). 12651724 10.1093/bioinformatics/btg034

[b53] Conesa A. *et al.* Blast2GO: a universal tool for annotation, visualization and analysis in functional genomics research. Bioinformatics 21, 3674–3676 (2005). 16081474 10.1093/bioinformatics/bti610

[b54] Ye J. *et al.* WEGO: a web tool for plotting GO annotations. Nucleic Acids Res. 34, W293–297 (2006). 16845012 10.1093/nar/gkl031 PMC1538768

[b55] Edgar R. C. MUSCLE: multiple sequence alignment with high accuracy and high throughput. Nucleic Acids Res. 32, 1792–1797 (2004). 15034147 10.1093/nar/gkh340 PMC390337

[b56] Zhang Z. *et al.* KaKs_Calculator: calculating Ka and Ks through model selection and model averaging. Genomics Proteomics Bioinformatics 4, 259–263 (2006). 17531802 10.1016/S1672-0229(07)60007-2 PMC5054075

[b57] Posada D. & Buckley T. R. Model selection and model averaging in phylogenetics: advantages of akaike information criterion and bayesian approaches over likelihood ratio tests. Syst. Biol. 53, 793–808 (2004). 15545256 10.1080/10635150490522304

[b58] Swanson W. J., Wong A., Wolfner M. F. & Aquadro C. F. Evolutionary expressed sequence tag analysis of Drosophila female reproductive tracts identifies genes subjected to positive selection. Genetics 168, 1457–1465 (2004). 15579698 10.1534/genetics.104.030478 PMC1448773

[b59] Tamura K., Stecher G., Peterson D., Filipski A. & Kumar S. MEGA6: molecular evolutionary genetics analysis version 6.0. Mol. Biol. Evol. 30, 2725–2729 (2013). 24132122 10.1093/molbev/mst197 PMC3840312

