# Supplementary material for: Transcriptome sequencing of three Ranunculus species (Ranunculaceae) reveals candidate genes in adaptation from terrestrial to aquatic habitats
Source: Sci Rep. 2015 May 20;5:10098. doi: 10.1038/srep10098 (PMC4438715; doi:10.1038/srep10098)
Supplement: Supplementary Information [file srep10098-s1.pdf]

Supplementary Information

Transcriptome sequencing of three *Ranunculus* species (Ranunculaceae) reveals candidate genes in adaptation from terrestrial to aquatic habitats

Ling-Yun Chen[1,†], Shu-Ying Zhao[1,†], Qing-Feng Wang[1*], Michael L. Moody[2]

[1]Key Laboratory of Aquatic Botany and Watershed Ecology, Wuhan Botanical Garden, Chinese Academy of Sciences, Wuhan 430074, Hubei, PR China,
[2]Department of Biological Sciences, University of Texas at El Paso, 500 West University Ave, El Paso, TX, 79968, USA.

* Author for correspondence: Qing-Feng Wang
Address: Wuhan Botanical Garden, Chinese Academy of Sciences, Wuhan 430074, Hubei, PR China.
E-mail: qfwang@wbgcas.cn
[†] These authors contributed equally to this work

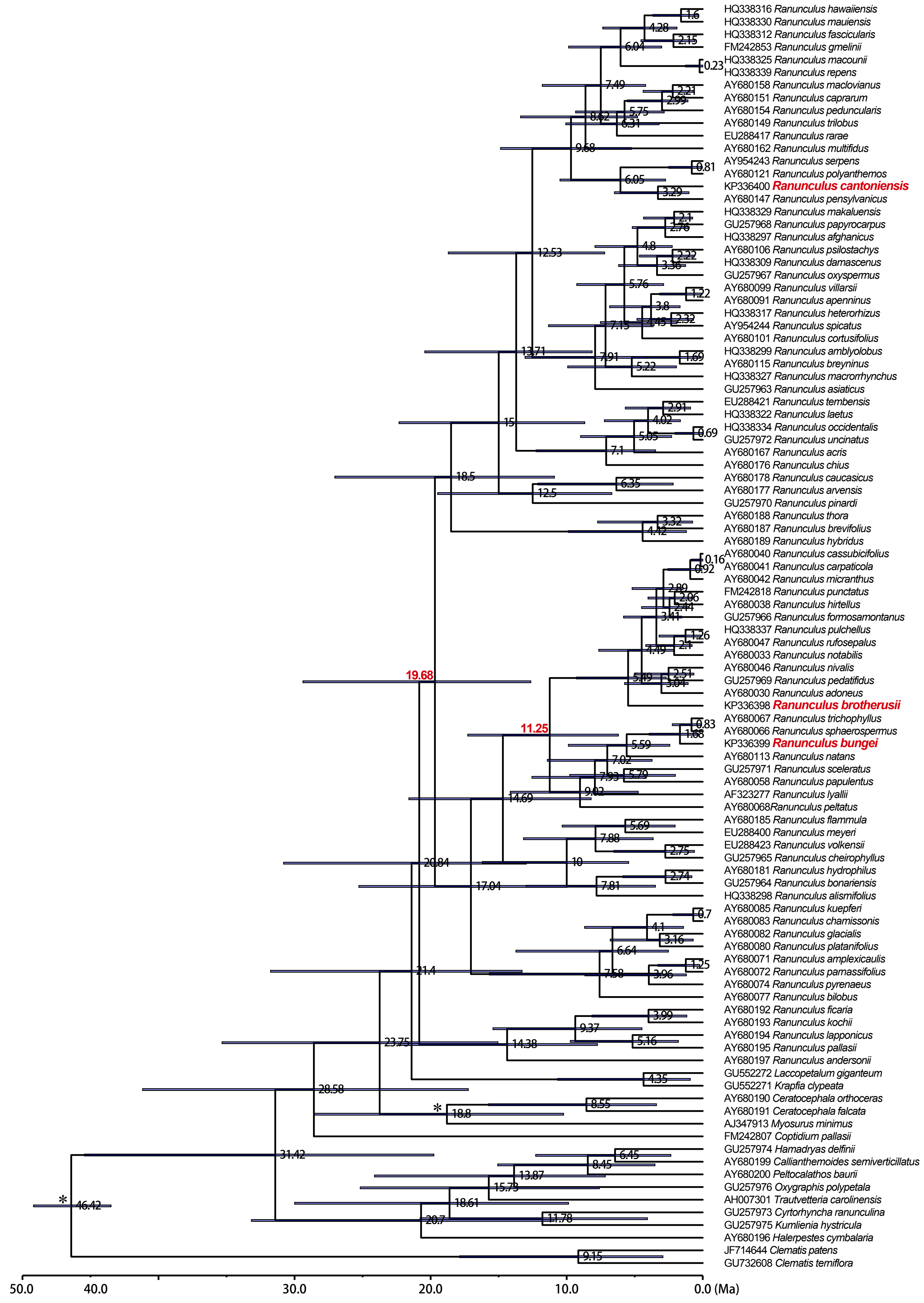

Fig. S1. Chronogram of *Ranunculus* using ITS. Blue bars indicate 95% highest posterior distributions, numbers along nodes indicate the mean ages, stars indicate the two fossil calibration points. The 3 species used for transcriptome analysis were highlighted in red colour and bold font. Genbank nos. were provided in front of species name.

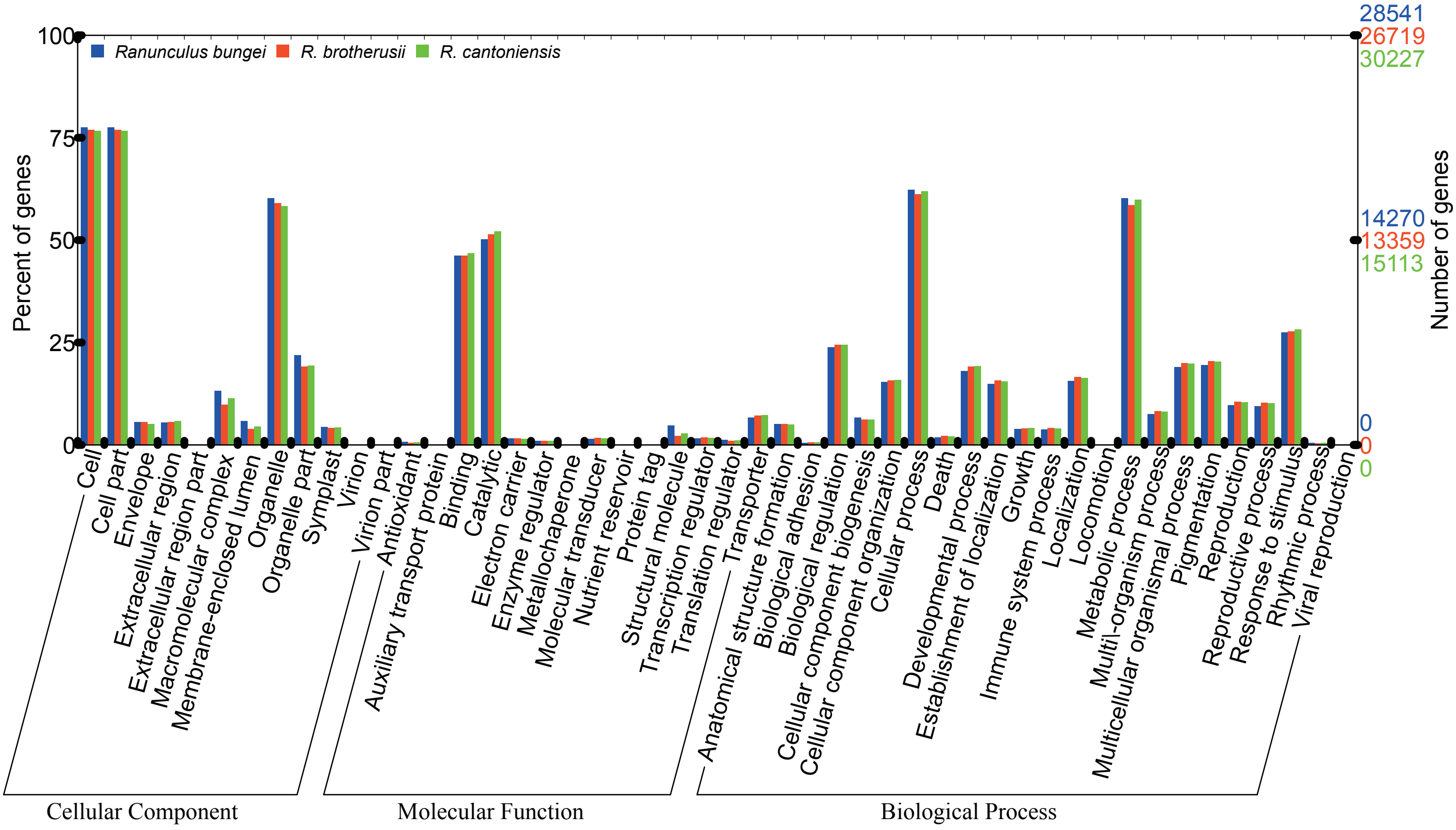

Fig. S2. GO categories of the unigenes of *Ranunculus bungei*, *R. brotherusii* and *R. cantoniensis* as plotted by WEGO.

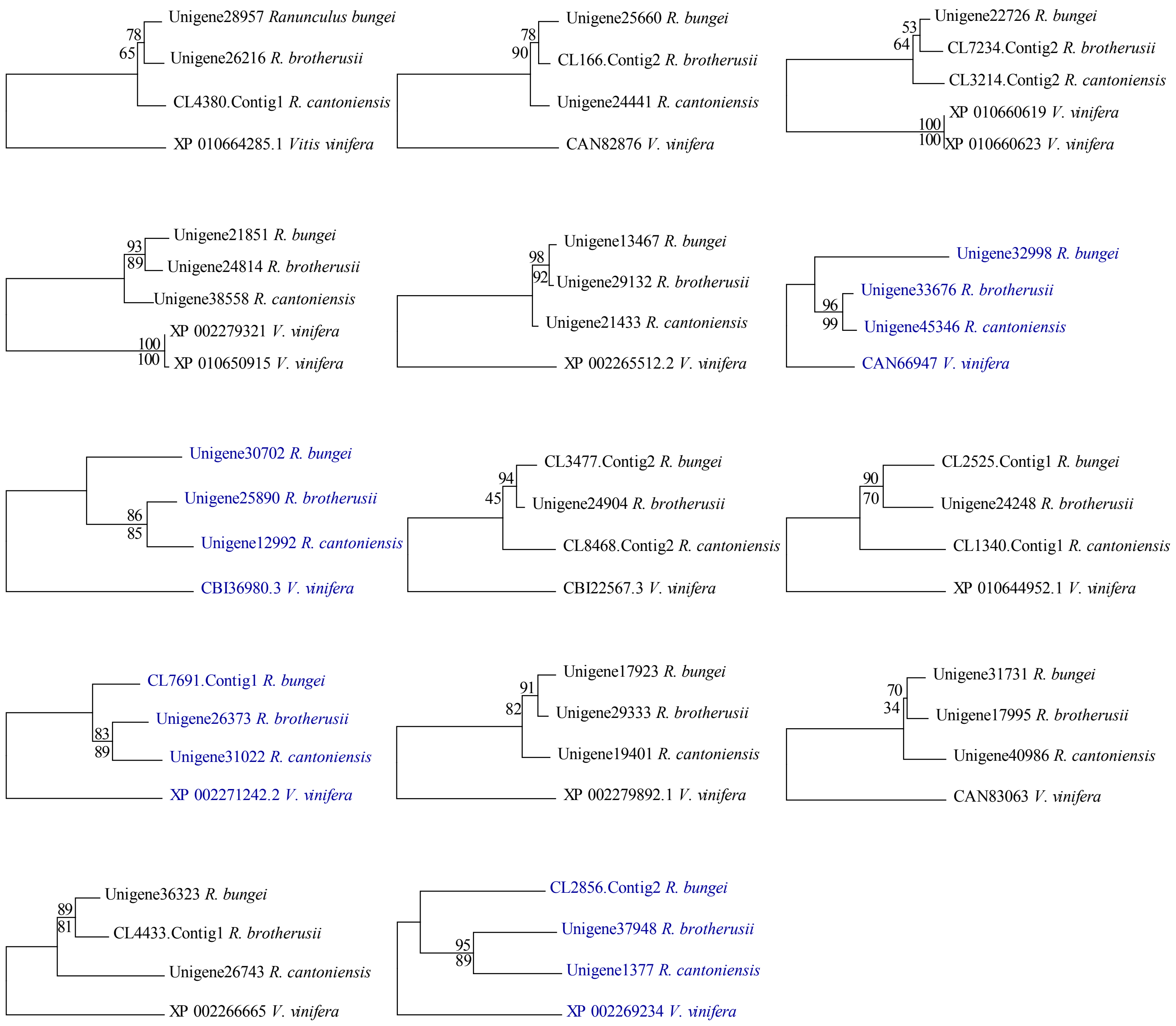

Fig. S3. Phylogenetic relationships of the PSGs in *R. bungei* and the orthologs in *R. brotherusii* and *R. cantoniensis*. Bootstrap values inferred from maximum likelihood and maximum parsimony are shown above and below the branches respectively. Orthologs with topologies inconsistent with that of ITS (Fig. 1) are in blue colour.

Table S1. *De novo* assembly from transcriptomes of *R. bungei*, *R. brotherusii* and *R. cantoniensis*, and annotations.

Sequences	*R. bungei*	*R. brotherusii*	*R. cantoniensis*
**Clean reads**			
No. of reads	106,256,806	106,580,162	102,823,982
No. of nucleotides (nt)	9,563,112,540	9,592,214,580	9,254,158,380
Q20 percentage	98.92%	98.97%	98.40%
N percentage	0.01%	0.01%	0.01%
GC percentage	45.22%	44.70%	44.08%
**Contigs**			
No. of contigs	126,037	114,753	140,218
Total length (nt)	45,026,691	39,928,897	47,992,342
Mean length (nt)	357	348	342
N50	692	727	649
**Unigenes**			
No. of unigenes	76,362	62,210	80,957
Total length (nt)	48,616,604	42,780,409	55,387,611
Mean length (nt)	637	688	684
N50	1132	1187	1170
**Annotated unigenes[a] number and percentage**			
NR	39,669 (52%)	36,419 (64%)	41,926 (49%)
Swiss-prot	25,448 (33%)	23,465 (41%)	27,663 (31%)
KEGG	23,305 (31%)	20,767 (37%)	24,531 (29%)
COG	14,894 (20%)	12,839 (24%)	15,098 (18%)
GO	28,541 (37%)	26,718 (46%)	30,226 (35%)
NT	25,575 (33%)	24,392 (41%)	27,542 (32%)
Total	41,111 (54%)	37,427 (66%)	43,565 (51%)
**Unannotated unigenes number and percentage**			
Total	35,251 (46%)	24,783 (57%)	37392 (44%)

[a] E-value $<10^{-5}$
