# Supplementary material for: Transcriptome sequencing of three Ranunculus species (Ranunculaceae) reveals candidate genes in adaptation from terrestrial to aquatic habitats
Source: Sci Rep. 2015 May 20;5:10098. doi: 10.1038/srep10098 (PMC4438715; doi:10.1038/srep10098)
Supplement: Supplementary Sequence Data [file srep10098-s3.doc]

Supplementary sequence data

Transcriptome sequencing of three *Ranunculus* species (Ranunculaceae) reveals candidate genes in adaptation from terrestrial to aquatic habitats

Ling-Yun Chen^1,†^, Shu-Ying Zhao^1,†^, Qing-Feng Wang^1*^, Michael L. Moody^2^

^1^Key Laboratory of Aquatic Botany and Watershed Ecology, Wuhan Botanical Garden, Chinese Academy of Sciences, Wuhan 430074, Hubei, PR China,

^2^Department of Biological Sciences, University of Texas at El Paso, 500 West University Ave, El Paso, TX, 79968, USA.

* Author for correspondence: Qing-Feng Wang

Address: Wuhan Botanical Garden, Chinese Academy of Sciences, Wuhan 430074, Hubei, PR China.

E-mail: [qfwang@wbgcas.cn](mailto:qfwang@wbgcas.cn)

^†^ These authors contributed equally to this work

********

**READ ME**

Sub-file 1 to 12 are the orthologous pairs with dN/dS >0.5, P <0.05 for each analysis in Table 1 in supplementary Table S2. The sub-file numbers correspond to the species pair numbers in Table 1 and the sheet numbers in Table S2.

Sub-file 13 (BBH orthologs) and 14 (OrthoMCL orthologs) are gathers of the clusters with genes of Ranunculus bungei positively selected.

All sequences have been aligned, and can be executed by program KaKs_calculator to estimate the dN, dS and dN/dS values (repeat the results in this study).

Please remove the title for each sub-file, then use the same method, viz. ML or YN as the file title marked, when you run the analysis. For the orthologs in sub-file 13 and 14, please use the method in Table 2.

This file should be divided to **14 plain ASCII text sub-files** (copy to new notepad) when you run the KaKs_calculator. In this case, more easy to use the ML or YN method.

All the sequences had been converted to fasta format to check the possible alignment problems.

Ranunculus chinensis is synonym of R. cantoniensis; R. tanguticus is synonym of R. brotherusii.

Please contact **Ling-Yun Chen, lychen83@qq.com**, if you need all the orthologs in this study or have any question.

********

**Sub-file 1. BBH_ML_bun-chi.cds_selected**

9843 Unigene44073_R-chinensis Unigene29908_R-bungei

XCTGATAAAAACATCACAGAACGGAAGAGTCTTTCGTTTGGAGATCACATGTTATCTTCCTTTGAAAATGCCATCTCTCCATCATCTAAAAGAAAATTTGCCTCTTTCAATCTAGCGGATCAGGACCATGTGCTCTCTTCCAAGAAACGCATTAGGTATGGCAGTCTGGATATTTCTGATTTTGGTGATTTTATACCACTGGACCCCATCGAATGCAGTCTTATGAGTCAGCAACCTTGTTCATCTGGATGTGTTCCTTCCTCCAAGCAAGTGAGTCTCGCGAGGGAGAAGAAGGGACGATCTTCAAGTCCAAGCAAGCACCCCCCTTATGTGCCTAACCATCCTACCTTGCAGCAACTAAGTTTTTTGAGGGAGAGTCTGAGTCCATCTTTAATTCCAAGTAAGCATTCCCCCTATGTGCTTGACCATTACTTTCTTGCAAAT------GCTGAAGGATCTCCATACCTAGACCTTCAAACTTCTGCTACTACTACCTCCTCTCACAAGCCAAGCGAAGAAAGAGATGAGAATGGGGTTTACACTGCTCTGCATTCTGATATTGAGGGCAAGAGAATTAGTGTGTTCGAACGCTTGAAGGAAGACAAGGAACCATATGATGCACATTTGCTGGTAAAGAAAATGGGATCTGCAGGAAATAATGCTGAGTTACTGGATGATGATGGGACTTCGGTTGACGAAATCATGGACTCATTGGAAAGGTGTAAAAAAGATAGGACAATCTCTACAGAAGAATCT---CGTAAAGGCAAAGTAAAAGATGTTGATGATGCTAAATCTGGTATTAAAAGTATTGAATTTGAGGGTGCATCTGAAGAAGAACAATTT---GATGTTGTATATCAAGAGATGCCAGTGAGCTTTAATAGACGA------------AAGATGCAAAGCGATATTAACAAAGGAAATGACGTCTCTAGTAGAACG---TTGATCAGGAGAAAATTGAAAAGGCCGTCTTTTGTTAAAGAAGAAGCCCAGGACAAAGAAGTTATCAGTGAGAGTTTGATTGGCAGAAGTGAATTCCGAGCTTCTCAAAGTTCTGGTTTTGAGGTTGATGATAATCCCGTTATCCATCACGACAACAACAAAACTGAATTTTTTGAAGACAAAGGTGAAGAAATGAGGGAGGACAACAGAAAACCCTTTATTCAAAACTTGGAATACAATGGTCACAGATCGGAAACAATGGCAGGAAGCCTGGATTTCAAGGGGAAAGTCAAGGGTTTCATCCAAGAGCTTACTGAGGATATAGCTGAGGAAGAGAAACAACAGTTACCAGAATCTTTTGGTGTTCAAAAGGTTAAGTACAGAAATATATGGCCAGACAXGGAGATATTTGCATCTCTTTTTTCTTATTCTTCAGGCCATGCTGATCTGAAAGCTGAGAAGGGX

---------------------------------------------------------------------------------------XXAAGAAAAATTGCCTCTTTCAATCCAGAGGATCAGGACCATATGTCTCCTCCCATGAAACGCATTAGGTCTGGCATTCTGGATACCTCTTATCTTGGCGATTTTATACCACTGGATCCCATCGAATCCAGCCTGATGCGCCAGAAACCTCTTTCTTCTGGATATGTTTCTTCCTCGAGGCAAGTGAGTCTTGTTAGGGAGAAGCTGAGCCCATCTATAAGTCCAAGTAAGCACCCTCCCTATGTGCCTGACCATCCTTCCTTGAGGCAAGTAAGTCTTCTGAGGAAGAATCTGAGCCCATCTTTAAGTCCATGTAAGCATCCCCCGTACGTGCCTGACCATCACTATCCTGCAAATTTAAGACCTGAAGGATCTCCATACCTAGACCTTCAACCTTCTGCCACTACTACCCTTTCCTACAAACCAAGCATAGAAAGAGATGACAATGGAGTTCTCAATGCTCTGCATTCTGATATTGAGGGCAAGAGAATTAGTGTGTTCAAACGCTTGAAGGAAAATAAGGAACCGTATGATGGACGTTTGAAGCTAATCAAGATGGAGACTGCACCAAATAATGACCAGTTACTGGATGTCGAAGGGACATCGGTGAACGAACTCATGGACGTATTGGAGACAAATAAAAAAGATTGGACAAAATCTACAGAAAAATCTCAACGGAAGGGCGAAGTGGAAGATGTTGACGATGCAAAATCTGGTATCACAAGCACCGAACCTGAGGATGCATCTGAAGAAGAGCAATGTGATGATGTTGTATTTGAAGAAATGCCAGTGAACTTTAATAGGCGAAATAGGATCCCAAAGATGGAAACGGAGATCAACGAAGGAAATGATTGCTCTAGTAGAATGCCGTTCAAGAGGAGAAAATTAAAAAGGCCATCTTTTGTTAATGGAGAAGTACATGACAAAGAAATCATCCGCGAGAGTTTGGTTAACACAACTGAATATGAATCTTCTCAAACTTCTGGTCTT---GTTGGCGTTAGTCCCATTATCCATCATGACAGCAACAAATATGAAATTTCT---------------------------------------------------------------------------------------------------------------------------------------------------------------------------------------------------------------------------------------------------------------------------------------------------

5488 Unigene27085_R-chinensis CL7407.Contig2_R-bungei

------------------------------------------AGAGATTCCCAGCGGCTTACAGAAAGATCAAAAATCCAG------------------------------------------CACGATGACATCGAACAGACGTCCATCTCCAATGCTCGATTCAGACAAAGGGAAGAGACTTCGGCTGCATCTATGAACGTGTAT---------------------GAGAAGAAAGAAGAGAGATATTCTCAGGCAAGTCAAAGTACAGCTGAGTTACAAAGGAAGTCTTCAAATGTTATGGGCACACGTAGAAGAGATTCTGGAATTAGGGATACTACCGAGAGTAGACTCAGTGATAGGCAAGATAATTTGGGATTGAACACTTATTTGATTGATGAAACA---------------------------------------------------------AGATCGCAGATTAGCCAACAGGATGAATTAAGAAGGGATTCCCAGCAGCTTACAGAAAGATCACAAATCAAGAACAATGATATTGAACAGACGTCCATCTCCAAAGCTCGATTCAGACAGAGAGAAGAGACTTCGGCTGCGTCTATGAACATGTCTGAGAAGAAAGAAGAGTTATATTCTCAGACAAGTCAAAGTATAGCTGAGTTACAAAGGAAGTCTCAA---CTATCCGAA---TCAGAGAGCCGCGACAATCATATTAGAAATACTTCGATATCACACACGAGGTTTGAATCGGCATCAAAGAACAGGGAAGATAATTCCACGAGTTCATTTCAGGAGGCAGAAAAGAAAGAAATGCACAGTGATTGGCATCTGCGAGACATCAAAATAACTGAACAAGATCAGAATCTATCTGATATGTCAGTGATAAGTGGCAATGATAAAGAAATAAAGAACACTTCCCAAAAAGTTATTGAAAGAAGAACGCACCCCGAAGAAGAGATTTCAGCTTCATACGTCTCCCAGCTGGCAAGCAACCGTGACAGCGACATCAGAAGGACTTCCACTTCACAAAGGCTTTCTGAATCTGTATTGAAG---------------------------------------------------------------------------------------------------------------------------------------------------------------------------------------------------------------------------------------------------------------------------------------------------------------------------------------------------------------------------------------------------------------------------------------------------------------------------------------------------------------------------------------------------------------------------------------------------------------------------------------------------------------------------------------------------------------------------------------------------------------------------------------------------------------------------------------------------------------------------------------------------------------------------------------------------------------------------------------------------------------------------------------------------------------------------------------------------------------------------------------------------------------------------------------------------------------------------------------------------------------------------------------------------------------------------------------------------------------------------------------------------------------------------------------------------------------------------------------------------------------------------------------------------------------------------------------------------------------------------------------------------------------------------------------------------------------------------------------------------------------------------------------------------------------------------------------------------------------------------------------------------------------------------------------------------------------------------------------------------------------------------------------------------------------------------------------------------------------------------------------------------------------------------------------------------------------------------------------------------------------------------------------------------------------------------------------------------------------------------------------------------------------------

TCGTCTTACTACTCGGTTTTGTCATCAGATGAAGTTGACAAGAGTGATGTGGATGAACAAGTTAATCATGGAAAACTGAGTGGGGTTTCTTTGAGCAGGAACAAGAAGGAACTGAAACGGTTTGATGATGATATAACTGTGAAGAACGTAATAAAACAATCAACAAAGAGCAATTATGATGCAACGGCTTCGACTTCAGGGGTGGTTGGTTCTGATCACCGTTTGAGGAATGAGAGGGAGAAGAAGTTTACAGACGTATCAATTCTTCGAGAGGAATCACAATTAGAGGCTTCAAATGTTATGGACAGCCATAGAAGAGATTCTGGAACTAGGTTTACTAGCCAGAATAGACTCAATAATAAGCAAGATAGTTCGGGATTAGCCACTTATTCGATAGATGAAACAAGATCACATGTTAGCCAACAGGATGGATCAAGAAGAGATTCTCAGCAGCTTACAGAAAGATCACAAGTTAGCCAACAAGATGTATCAAGAAGAGATTCTCAGCAACTTACAGAAAGATCACAAATCCGGAACAATGATATTAAACAGACGTCCAGCTCCCAAGCTCAATTCAGAGAGAGGGAAGAGACTTCGGCTGCATCTATGAACGTGTTTGAGGAGAAAGAAGAGAGATATTCTCAGACAAGTCAAAGTACAGCTGAATTACGAAGGAAGTCTCAACACCTATCCGAAAGATCAGAAAGCTGCGACGATCATATCAGAAATATCTCAATATCACATACGAGATTTGAATCAGCATCGAAGAACAGGGAAGATAGTTCCACGATTTCACTTCAGGAGGCAAAAAAGAAAGAAATGCATAGTGATCAGCGTTTGCGAGAAATTAAAATAACTGAAGAAGATCAGAATCTCTCCGATATGTCAGTGATAAGTGGCAATGATACAGCATTAACAAACACCTCCCAAAAATTTATTGAAAGAAGAACGCACCCCGAAGAAGAAATTTCAGCTTCATATGTCCCCCAGATGTCAAGCAATCATGACAGTGACATCAGAAGGACTTCCACTTCACAAAGGCTTTCTGAATCTGTATTGGAGAACAGAGAAGAGAGTTCAACTTCACTGCTAGCTTCACTTAGTGAAGCTGAAGAAAGGAACCAAACTGCTGAAAGAGTTGTTTGGGACACCAAATTTACTAAACATTCCGAAGGCCTAAAGCTTACATCAGAAACATCTGCCCACGACACGACAATATCTTCTGATTCCCATAAATATTTTGAAAGAAAAATGGGTCCTAGACAAGAAAATCCATCGTCAAATGTTGATTGGGGTACAGAAGGAAGAGGGGAAACCCAACAAGTAACATATCCCATAAAACAGTTAGGGTCAAGGACACAGTCTGAAAGGCTTACCGAAACATTAATGTTACAAGAGGGCTCTGCGAACTGGGCTTCCAGATCCCAATCATCCGATCTGAATAAGCAGAAGACTGAGCAACAAATGGATGAGCAAATGGAGCTTGGAAGAACCCTCCAGGTGATGGTAACTCCACCACCATCTCAGATTTTACATAGGACTTCCAGTGGAACGGTTTTCCACCAAAGACCCAATGCTGGGAACATTACAGGTGTTATATACTCTGAAACTCCTGGTAGTGGTTCTGGCACCATATATTCCGAAGAGATTACTCCATCTTCTTCCAATGATTTTATTGGTCAAACTAGTATGGATAACAATATTCGGAGCCACTCAAATATAATGCTGCATGACGACGCATTAGGTTCAGCTAATCGCCTAGAGGAATCTTCTACACAGTTTTATAGGCAGTTTGTGGAGAAATTAAGTCAAGAAGCATCAACTTCTGAATTTCCGGTGGGAAAGGTATCATCAGAGCCATCCCCTAGCAACAAAGATGCAGAAGACATACAACATAGTTTGAAACAGCAAGTACCTGATGATGTCCGCTCACAGGTGCATGATTCAAGGAAATCATCTTCTAGATCGGGAGCTAGAGGACCTTCAGATGAAGTTTGGGACATTGTTGACCCGTATTCGGAGAAGCCTGCGAGGGATGAAGATTCCAGTGAAGTCTCTTCTTCCACTAAAACTGCCGTTGCCAGAACCAGTCGATCCTTGTGGGGTATCATTGGAGATATAGTTCGATTGCGATGGGGGACACACTCAGAAACCCGCACATCTGCAAAGTCCCGTGGGAAGAGTTCATCGAATCAGTCAGTTGGCAGTGAAGCGTGGTTTTCCAGCCACGAGGTCGATGAAAAATCTGATGAGAATGTCAAAAAAGGAAAAAAAGTCATGCCAAAGTTGCCCAGAAATTCTGACCAATCCTTGAAAGCTGAATCTGAGAGTCTAGGAGGTGCTTTGGAAGGTACAAGTGCAGACAACATGGCATTAGTGGCTGAGGCTGATAGATTTGCTTCTTTGGATATTGTTAAAGGCAGTTCAGCATCGTCAACTTCGAAAGTGGAAAGCGACAGACTGGAATATAAGAGCAATCAAGGAATTACCTCCAGTACCTCAATACCCTCTGCATCACCACCACCTCCATCTAAACACTTCATGCGTTCTCCCGCTGTAAGGGGAGAAGTACCTGAGAGTAGTGAAGCTATGGCATCTAGAAGTGACTTGGGGCAAATGGAGCAACTTCGACAAAAGAGACCAACTGAAGGATCTGGAACTGAGATGAAGGATGGGGAACTAAAGCAAAGGAAGCTTCACAGGAATAAGCAGGTCGAGAAAGAAACATTTGAGGACTGGGAAGAAGCTTATAGGGTTGAGCGCGAACAGAGAATCATCGACGAAATGTTTATGAAGGAAGCTCTTTTAGAAGCAAAAAGGGCTGCTGATACTTGGGAGGTACCTGTTGGAGCTGTGCTGGTACAGCATGGAAAAATCATTGCTCGTGGTTGTAATTTAGTGGAAGATTTGCGTGATTCCACTGCTCACGCTGAGATGATTTGCATACGCGAGGCTTCAAAGACCCTTCAGACATGGAGACTTGCGGAGACAACACTTTATGTAACACTCGAACCGTGTGCAATGTGCGCTGGCGCAATTCTCCAATCCAGAATTGATACAGTTGTATGGGGAGCTCCTAATAAGCTTCTAGGAGCTGATGGCAGTTGGGTTAGACTTTTTCCGGGTGGTGGATCTGATGCAGGTGGAAGTTCTGATCTTACAAGTCAATTAGGCGGCCCAGTCCATCCGTTCCACCCTAAAATAACGCTCAGACGTGGGATTTTGGCAACAGAGTGTGCCGACGTTATGCAGCAATTTTTTCAACTGAGGAGGAAGAAGGTTAAGAAATCCGAGTCACCACCTCCAAGTTCGAGAACTCGTATTTTAACTCACCCCTCTAAATTAGTCTCGAAGATGCATCACATTTTTAACGTTATGTTCTGCTTG

4278 Unigene18391_R-chinensis Unigene20489_R-bungei

------------------------------------------------------------------------------------------------------------------------------------------------AACACTGGCAAAGAAGGCTCACGTGTTGGTGATAGTGATAACCAAGCAGATGATAATGGGAATGAACAAATTTTATCTGACGCGTTTGTATCAATTCCTGAAGAGAAGACCCATACAGATAATATGGCCCTTGATAACCTGGTGTCTGGTATCACCTCTAGTGTCCTTGGGTACCTAGAGGACACCTCT---------AATGACCACTTGGATCTTAGCTCCAATGAACATTCTATGGCCGTTGAGGAGAAGGCTGAGGAGACGCAGCTACCAGAAGTGGTGGATAAAGAGAATACCAGTCAACAGAATAACATCAGTCCAGATAAGCTTGCCACAGATTCCACTCAGTCACACAGTTTTGTTGATGAAAACTCAACTAAGTTTAGTAACCACGTAGATCATCGTGTAAATGGTTCTCATGAGGTCTTGAGCGACGAGCTTGACATGACACACCAGTGTGAAACTATGGTGCACGACTTGCATGAAAATGAAAATCACGTGGATCTTAGCTTAAATGGATTTGATGGTGACATAGATGACAAGGTAGAGGATGTGGCAGAACCCAGCAGTGAGAGATTCGCTGAAGAAAATGATACTCAGCTTGAAGAACCATGT---TGTCCTATAGAAAAAGATCTTCCAAATGTTGGCCCATCAGAAAATGAAGTCGGACTTCCTGAAGAAAATAACGAGGGAATTCTGGAAGCTTCTAGAGAATCATTGAATGTGGAGAAACCGTCTTCAGAGGTTGGTGGAAGGAAAAATGTATCGAAAAGAAAAAGGCTTGCAGTTGCAGTCGTAGGCGAAGAAGAATCA------------------------------------------------TTGCCAAAGAAAAAAGCAGCACAAAAGATTGTAAGAGGAACCAGGGAAGTGTCGCGCAGGAAAAGCCTAATTGGAGCTGGTACAGAATGGAAAGCGGGTGTAAGAAGAAGCACTCGAATGAAGTTCAAACCACTCGACTATTGGAAAGGGGAACGTCTCATTTACGGAAGAGTGCACGAGAGTTTGGTGACTGTAATTGGATGCGAAAAGTGTGGATCTCCTTGTGGGAACAAAGGAGATCCTAAACTCAAGATTTTTGATTTCAAGGAACTTCAGTCCATGGCA

GGTTCTCCAACCCCACCAAGATATCCGTTTCCTGCAGTACCAATGATGTTCAGAGATGATACAGACTCAGATCAGCCAGGAAATCCAGTCTCTTCGCCTGGGATTGAGAGTCCATTACCCATCAAAGATTCTATACATGTGGACAGCACTGATAGAGAAGGCTTACTTGTTATTTCTACTGGTAAACCAGCCGATGATAATGGGAATGGACCAACTTTATCTGGCATGTCTACATCAACTGCTAAAGAGAAGAACCGTGCAGATAGTATTTCCTTAGATAATCTAGTGTTAGGTATCACCTCTAATGTACTTGGGTCTGTAGAGGACAGCTCAACTAAAGTTAGTGACTGCTTGGGTCTTAGTTTGAATGAACATTATATGGCCGCGGAGGAAAAGGCCGAGGAGATGCAGCAACCAGAAGTGGTGGGCAAAGAGAATACCCGTCAACAGAGTAACATGGATCCAGATAAGCTTGCCACAGATTCCGCACATGCACACAATCTTGTAGATGGAAACTCAACTAAGTCTAGTAACCAAATGGACCATTGTGTAAATGGATCTCATGAGGGCTTGACCGACAAGCTCGAAAAGACACACCAGCCCGAGACCACCGTGCACGACTTGCATGAAAATGAAGATTATGTGGACCACAGCTTAAATGGGTTTGATATTGACATGGATGACAAGGTTGAGGATGTTGTAGAACCTAGCTGTGAGAGATTCACTGAAGAAAATGCTAGTCAGTTTGAAAAGCTATGCCCTCGTCCTATAGACAAAGATCTTCCCAGTGCTGGCCCATCAGAAAATGAAGTCAGACTTCCTGAAGATAATAACGAGGTACCTGGGGAAGCGTCTAGAGAATCATTGAATGTCGAGAAATCGTCCTCAGAGGTTGGTGGAAGGAAACAAGTTTTGAAAAGGAAAAGCCTA------GCAGTAGTAGGAGAAGAAGAGTCATCGAACTTTGGAAGCCCAAGCCTCCCACAGAACCATGTAAGAGAAAATCCCCCACACAAAAAGGCAGCACACAAACTTGTAATAGAAAAACAGCGAGTGTCACGTAGGAAAAGCTTAATAGGAGCTGGTACTGAATGGAAAGCGGGTGTAAGAAGAAGCACTCGAATGAGGATGAAACCACTCGAGTATTGGAAAGGGGAACGTCTCATTTACGGAAGAGTGCACGACAGTCTGGTGACGGTGATTGGATGCGAGAAGTGTGGTTCTCCTTGTGGGAACAATGGAGGTCCTAAACTCAAGGTTTTTAATTAC---------------------

4365 Unigene22543_R-bungei Unigene18722_R-chinensis

GCAGTAGCAGATGGCAAGGACATTGCGGAAGGAGCAGTGACTCTTGGACAACCTATGTCTACCACTTCTGATGTTAGCCCTTCAGAGAGCTTCTATAAAAACTCCAGTGACTATGGCGTTTCTGACAATTACACTGTTACAAATGCTACTAGCACAGTTGCGAGCTTAGCTGCATCCACATCTCTGCCTTCT---------CATGTATCGTCTGTTTCAAGTAAATCGACCGGTGCCCCGTTCATCTTTTCTTCCAAAGGTGTTGACAAAGCTACCCTGCTTCCATTATCATCGGCATCTGTTGTGACCGAGTCCATGGGCCTGAATGGGAAGCAGTCAGAAGAGAAAATTGAGGTTGTG---------------------AGCAGAGAGGGTGGTGATAATGTAGAGGCTGATGGTGAATCTTCATCCAAGACAGTACCAGTCTCCCTTGGAATATTTTCCTTTCAAGCTTCAAACAATTCAACTCTGAATAACGAGTCAAATACTTCCTCTCCTCTACTCACAGCCACTACTGATAAATCCAAGAGCAATGGTCTATTTGCACCCCTCTGCAGCATTACGACAACCACATCTGTTACGGCTCCTAACGTAACCACAAGCACCCCTAGTTTTATATTTGGAGCTTCAGCTACG---TCAGCAACTGGGACAGTGAGCGCTCCAGCATTTTCTTGGAACTTGCCGACACAGTCTACCACATCATCACAAACAGCTGGGGTAACTGGGCCGATTTCCTCCAGCACTAGTGGATCTATTTTTGGTTCTTCATATATCCCCTCCCAAGCGAGTCCCAGTACACAGTTGGCTGCCCCATCATCTGTATTTGGAACCTGTTTCTCCAACACAGGTTTCAATTTTGGATCTTCTTTGAGTGCACAAATGCGCTCATCCTTGGGCAGCTCTTCACCTTCCATGTTCACATTCACTTCAACTGCTACTCCCACTCCAATTCCACTGCCCCCTGTCAATAATAATGTTGAAAGGAGCATTGAAGAACCATCTACTTCATCTCAACCATCTCCATTTGTATTTGGCGGACCTTCATCATCAGCA---------GGAGGATCCAGTATGTTTCAGTTTAGCACCCAACAGAATCCATTCTCTGCAGGAGCAGGAGTATTCACAATTGGAGCAGGTGGCGGCGGTGGTGGGGACAATGACAAATCCAAGCGAAGAATTGTGAAAATTAATAGGAATAAATCGAAGAAA

------------------------------------------------------------------------------------------------------------------------------AACTGCCTTGTTACAAATGCTACTGGCACAGTCGCAAGCTTAGCTGCACCCAAATCTCTGCCCACCCCGCCATTCACGATATCGTCTGTTTCAAGTAAATCAACCAGTGCTCCAGTCAGCTTTTCTTCTAAGGATGTTGACAAAGCTACCCTGCTTCCATCCACATCGGCATCCGTTGTGACTGAGTCCATAAGCCTGAATGGACAGCAGTCAGAAGCAAAAGTGAAGGTTTTGAGCAGTTCGAATACTTCCAGTAGCAGAGAGAATGTTGACAATGTGAAAGCTGATGATGAATCTTCATCAATGACAGTAGCAGTTACCCCTGGAATATTCTCCTTTCAAGCTTCAACGAATTCAACTCCGAAGAACGATTCAAATACTTCCTCTTCTCCACTCACAGCCACTACTGATAAGCCCAAAAGCAATGGTGTGTTTGCACACCTCAGCAGCATTACGACAACCACATCTGTTACCGCTCCT---------ACCACAGCTTCTAGTTTTATATTTGGAGCTTCACCTACGCCATCAGCAACTGGAACAGTGAGCGCTCCCGTATTCACTTGGAACTTGCCGACACTGTCTGCCACATCATCACAAACAGCTGGGTTAACTGGGTCGATCTCCTCCAGCACTAGTGGGTCTATTTTTGGTTCTTCATATATGCCCACCCAAGCCAGTTCCAGTACACAGTCGTCTGTCACATCATCTGTATCTGGAACCTCTTTCTCCAACACAAGTTTTACATTTGGATATTCCTCGAGTGCACAAACGCCTACATCCTTAGGCAGCTCTTCGCCTCCTATGTTCTCATTCACTTCAACTGCTACTCCCACT------------CCACCTGTAAATAATAATGTTGGTATGAGCACTGAAGAAGCATCCAGTTCATCTCAACCACCTCCATTTGTATTTGGCGAACCTTCATCATCATCAGCACCAACAGGAGGACCCAGTATGTTTCAGTTTAGCACCCAACAGAATCCGTTCTCTGGCGGAGCAGGAGTTTTCGCTATCGGAACAGGTGGTGGCGGTGGTGGTGACATTGACAAATCCAAGCGAAGAATTGTGAAAATTAATAGGAATAAATTGAGGAAA

4844 Unigene22204_R-chinensis Unigene7563_R-bungei

------------------------------------------------------------------------------------------------------------------------------------------------------------------------------------------------------------------------------------------------ATTGACAGCAACCTTGAGTACAATGATATTACTAGTTGCAAAGGCGAAAGGCATGTCTTCTGCGTATGCGGCGATGGCGGGGTGTTCTGTGGTTCAGCTAGTAAAGTGGATTTAAAAGAAACATATGCACTGATTCTTAAGAATGCAATAGAAGATATATCTGGCTGTACTATGGAAAAGGGCTATGTCCTGCCCTTCCATTGGAGTAATAAGACTATTTCTCGAATCAGTTTTAAAGCGAGCACAGCCGGTCGCAGTGACCTGCCAGAGTGGGCACAGCAGCTGATACCCGTGCAAAAGCTGCGGACAAATGTGGGATCAAAAATCCGCAATTGTGTAGATAATGCTTTGGCTTTGGATCCTCCAGATTGGGCAAAGGAAATTCTGGAATACTCTAAAACCAAAGAAGTCTACAAGTCCAACGCGGCAGGGCCTACAAAGAGAATTGTTATTTCTGTGCTTTCGGAGCTGAACAGCACACTCAATGGCCAACCAGATGAGGAAAAGGGCAAAGGAAGGGAGGAAAATAGAACGAAAAGAGTGACAGATTTGATCACGGAGAAGTGCCGTAGTGTATTACATGATGTCATTGCTGGAAATAACGTAAAAGGTGTCTGCGGTAACCTCTCTTCATCGTTTCACAGGGATGACAATGCTCTTGAATTGCCTCCCACAGTATCTCTTCCTCTGGAGTTTGATGCCATTTATTTGAAATTAAGCAATGGTGATTATTCTGGGTCTCATCAAGCATTCCTTGAGGATGTGAGAGAGGTATGGACCAATATACATGTAGTCTGCAAGGATCAACCTGAATCAGTGCAATTGGCAGAAAACTTATGCAAACATTTTGAACTACTTTACAAGAAGGAGGTGCTCCCTCTCGTTGAAAAATTATCAGAGGCTTCTGAG---------------------------------------------------------------------------------------------------------------------------------------------------

AGCAGTTTTTCAAAGCTTCTTGGTAGTTCTGATACGAATTATAAAGATGCTCAACACATGTTGCTCAATGTGTTAATATGTGAACGGTCCAAGGTTTTGGAAGTTGGAGATCCTACCTTTTTTGCTTGCAATAGGACTGACAAAGAATGTGCACGTAGTGCGGATGATTTTAGTTTCATTTTGCTAAATGAACTTACATGGCCAGAATTAGCTCGTAGATACATTTTGACTTGCCAGTACATTGACAGCAACCTTGAATCCAATGATATTACAAGTTGCAAAGGGGAAAGGCACGTCCGCTGCCTATGCGGCGATGGTGGGGTGCTCTGTGGCTCAGTTAGTAGAGAGGCTTTAAAAGAAACATATGCACTGATTCTTGAGAAGGCAGTGGAAGATGTATCTGGTTGTACTTTGGAAAAAGGCTATGTCCTGCCCTTCGAATACAGTAATAGGATTATTTCTGGAATCAGTTTTGAAGCAAGTACAGTCGATTGTAGTAGCCTGCCCGAGTGGGCACAGCAGCTGATACCTGTGAAAAAGCTACGAACAAATGTGGGGTCAAGAATTCGTAAGTGCATAAATGATGCTTTGGATTTGGATCCTCCACATTGGGCAAGGAAAATTCTGATGGATTCTATCAGCAAGGAAGTCTACAAAGCCAATGCGGCAGGGCCTACGAAGAGAACTGTTCTTTCGGTGCTTTCGGAGCTGGAACTTGCGCACAATGTCCAACCAAATGAAGAAAATGGCAAAGGAAGGAAGGAAAATAGAACTAAAACAGTGACAGATTCGATCATGGAGAAGTGCCGTAGTGTATTACACTATGTCATTGCTGGAAATGATGTAAAAGCTCTCTGCAGTAAGCTCTCTTCGTCCTTTTACTGTGATGACAATGCTCTTAAATCGCCTCCCACAGTATGTCGTCCTCTGGATTTTGATGCCATTGACTTGAAGCTAAGCAATGGTGATTATTTTGGGTCTCATCAAGCATTCCTTGAAGATGTGAGAGAGGTATGGAACAAGGTACGCATAGTCTGGAAAGATGAACCTGGATCAGTGCAATTGGCGGGGAACTTATGCAAACATTTTGAACTGCTTTACGAAGAGGAGGTGCTCCCTCTTGCTAAAAAGTTACCAGAGGCTCCTGAGGGTGTTTGCAAAGCCTGTGGGATAGACAAAGATGATGAGAAAGTCCTATTGTGTGATACATGTGATTCAGAATATCATACTTATTGCTTGAAACCTCCACTTCCGAATATTCCGAAAGGGAATTGGTATTGTCCTTCATGCGCTGTA

2961 Unigene10369_R-chinensis Unigene31323_R-bungei

ATAATTCGATGTAATGTTTCGTATCTCCAAGTGTTGAATATATCTACTCCATTATTAGAAAGTTTGGAGTTAAGAGAATTATCAGTTGACTGGGATAGTCTTTGTACAGTAAAGATGGTTACTCCAAATCTTAAATCATTGAAGGTTTGTTCTTTTTCCAATAGCCAGTCCGAAATTACAGTGGACTATTCAATGGAAGATCTTCCTTCACTTATTCAGGCACATATTGACTTTTATCCAGCCGAAAACGAACTCTATGATAAGAAGTGTCATGAAGGTGTATTTGAAAACTTGTCAAAGGTTTACTATGTACAATATTTGCAACTATCTCTCGGTTCTATAAAGGTGTTAACAGATTTCCCAGAATTATTTGAGCAGCTTCCAGATCTATATCCCAATCTAAAACAACTGATCCTAATGTCATCACTATCATCTACGGTTCTTCCCATCCATGCAGTAATCAACTTCCTAAAAAGACTTCCCTCTTTGGAGAAACTTGTTCTGCAAAGTACTTCGGGATTATGGATTCAGACGTATACGGAAGAAGACTTGGAATCGTTCCCACCACACCATACTCTATTTAGX---------------------------------------------------------------------------------------------------------------------------------------------------------------------------------------------------------------

------------------------CTCAAAGTGCTGAATATATCTACTCCGTTGCTAGAAAGTTTGGAGTTAAGCGGAGTGTCGGTTGACAATAATAGTTTTTGTACAGTAAAGATGGTTACTCCAAATCTTAAATCATTTAAGGTTCATTCCTTTTGCTATCCGGTTTCCGAATTTACAGTGGATTATTCCATGGAAGATGTTTCGTCACTAATTCATGCACATATCGATTCTTATCTAGACGAAGATGACGTCTACAATAAGAGGCGTTCTAAAGGTTTATCTGAGATCTTTTCAAAGGTTTACAAGGTACAATATTTGCAACTATCTCTCCGTTCTATAAAGTTGTTAGCAGATCTCCCAGAATTATTTCAGCAGCTTCCAGATCTATATCCCAATGTAAAACAGCTAATGCTTATGTCACCACTACTATTGATTGCTCTTCCCATCCATGCAGTATTCAACATCCTCAAGAGACTTCCCTGTTTGGAGAAGTTTCTACTGCAAAGTATTTCGGAATTGTGGATTCAAACCTATACGGAAGAAGATTTGGCATTGAATGCAACCCACCATAGTTTATTTAGTTGTCTGAAAACCTTTGAGATTCAAAATTTCCAATGTCTCGAGGTCGAAATAAAGTTTTTGCAATCGATACTTGAGAGAGCTACTCTTTTAGAGAAGATGATTATCCAGACACAAACATCAGTGATATCATATGAAGATTTTCAGGAATTTAAGGAGAAGTTGTTGTCTGTACCTCGAGCTTCCTCAAGTTCCTCAATATCGTTTCTG

5330 Unigene26158_R-chinensis Unigene23342_R-bungei

ATGGAGGAAGACAAGAAGAAAAAGAAGAACAAGAAGAAGAAAAACAAACAATCTGCGAAACAATCAGATGAAATTATTAAAGATAATAATAATAATAATGGAGAAAATACGGTTTCGGAGCAGAATCATACGTCTGATTCGCAACAGATTCACACTGCAACTGCGCAGATCTTGAAAGATAACAAGAATGAAGCTGGAATGATGGAAGCAGATGTGAAGCAAGGCGGAGATCACACCAATGGTACAAACGGGGCTAATGGGCATGAAACAGAAAAGCAAATTTGGCTGCAAAAGGAGGCAATGCTAGAAGATAAAATTAAACAGCTGGAGGCACACAAGGGTTCTTGCATAGAGAAGGAGGTAGTTTTGGAGAATAAAATCTCACGCTTACAGAAAGAAAATAGTTTGTTGCTCCAAAAAGAGTCCAGCATTGACCAGCAGCTGAAAGACTTGCTGAATGAAAAAGCTAATTGGACTTCAAAAGAGACTGCTTTAGAGGACAGAATCAGACAATTAGAGAACGATGCTGGATCCTGGATCTTAAAAGAGAACTCATCAAGGGAAACGATAGATAGACTGAGCAAAGAGTATATGGAAGTACAGGCAAAGGTAAAGTTGCTGGAAGAATCTAGGGATGGTTTATTACAGAAAAAC------------------------------------------------------------------------------------------------------------------------------------------------------------------------------------------------------------------------------------------------------------------------------------------------------------------------------------------------------------------------------------------------------------------------------------------------------------------------------------------------------------------------

ATGGAGGAAGATAAGAAGAAAAAGAAGAAGAATAAGAAGAAAAATAAACAAGCTGCTAAACAATTAGATGAA---ACTAAAGCGAATAGTAATAATAATGGAGAAACTACG------GAGCAGAATCATGCGTTTGACTCG---------------------ATGCAGATTCTCTCGAAAGACGATACGAAGAGTCGGATGTTGGAAGCAGAT---------GGTGGAGATTACGCCAATGGTATAAATGGGGCTAATGCGGATCAAACAGAAAAGCAAATTTGGCTGCAAAAGGAGGCTATGCTAGAAGATAAAATTAAACAGCTAGAGACGTACAAGGGTTCTTGCATAGAGAAGGAGGTGGGTTTGGAGAATAAAATCTCAGTCTTACAGAAAGAAAATAATTTCTTGCTCCAAAAAGAGGCCAGCATTGACCAGCAGCTGAAAGGCTTGCTGAATGAAAAGGCTAATTGGACTGTGAAAGAGGCTGCTTTAGAGGACAGAATTAAACAATTAGAGAACGATACTGAATCCTGGATCTTGAAAGAGAACTCGTCAAGGGAAACAATTGATAGACTCAGCAAAGAATATATGGATGTACAGGCAAAGGTGAAGGTGCTGGAAGAATCTAGGGACGGTTTATTACACGAAAACCAGGAGTTGATTGAAAGATTATCATTTCTTCAGTCACAGGTTGAGCATCTCGAAGAAAAAAGATCTATCTCTGCATCCTCAGCAACAGAAGTTGAACAACTGGAGCACCCGACTATTGAAGCTGCAAATTCACTTGAAGACAAGTTGATCATAGAGACTGCCAAGGGTTCTGAATTGACAACCCAACTGCCCGAAATTGCTTCTTTTAATTCCAACGTGCCTGAGTATAGCAGCGAGATTTTAGAAACGCAGGAAATAGTGGAGGCTCCTGTGGTGGTTGATAATCGCGAGGAGACACAGTCACATGGATCTTATGAAACAGATGAGATTGTGCAAATTCCATTGGACGAAACGGAAATTCGGGAAGTGGAGGTTGAAGATGCTGAGGAAGATAAAGTCGGCATTGCACTGACGGAAGCTCCACTGACTGGAGCACCATTTCGGTTTATATCTTTTGTTGCTAGATATGTGAGTGGTGCCGATTTAATTAATAAGAACAACTCG

1105 CL4225.Contig1_R-chinensis CL61.Contig1_R-bungei

------------------------------------------------------------------------------------------------------------------------------------------------------------------------------------------------------------------------------------------------ATGGGTGTGATTAGTGGATGCTTAAAACAGTATCAGGCTAAACAATTGCAAAAGATTCGTGTTGAAATCTATGAAACCAAGGATGATGATTCTGAATGCGTACTAGAATGGCTTAAATTTGCTGTTGATAGAAAAGTTGAAGATGTTAATGTTGTTGCCCCGGTCGGTTTGAACCCCTCAACCTGGTACAAAGTCCCTGATTCCCTGTTTTCATCGCGATACTTACGAGTGCTTTCTTTGGGCTACTGTAACTTGTATCCACTTATTCATATCAACTGCTGCTTTGACTCTCTGGTATCTTTATCTCTGAAACAATTCCAAGATTATGATGGGGCACTGCAGTCACTAGTTTCTCGTTGTCATAATCTCCGATTCTTGTACTTTATGAATTATATTGGAAGGGAACGCCTTCAACTTCCTAAGCTATCTCTTTCGTTACCTAGGGTGCAAACCCTTCACATTGTCTCTGCTTCAGCATATCCTTTAATTGAGATTTTTGCTCCAAATCTGCAAAACTTGAGAGTTCATGGTCCCGTCTTTTGTAATCTACGTGTATCGAATTCTCCCCAGTTACAAAAGGCCTACTTCTTCTTCCGAAACTTCTCATTAACCGAAAGTAGGCAGTCTCCTCTTGATCTATCTCCTTCCAGACATCCCCATTTTTGGGGTTTCAATTTGCAAAGCATGGCAAACATTAAGTCTTTGTACCTTATGCATGATGTATTCAAGGTAATGTACACAGACGACTATGCGAGTGGAGCAAGAAAATTTGCTCTCAACAATCTAAAAGAGCTCGTTTTGGATCACATGCAATTGGATGCTACTGTATATCCTGTTGTGAATTTCTTGAGAGGGTGCCCTTATTTGGAGAAATTTTGTGTTATTTATGGTGTTGTCCAATCAGAGCCAAATCTCCTTGTCAAAATAAAGAAGTTGGCTATTGAACCCGATGATGATGATCGAAAGCTTCATTGTGATTTCCATGGATACTTTTTTCAACACCTCAAAAGGGTAAGGCTGAGAGGAATTCGAGGCGATGCAAAGCAAATGATACTTCTCAAGTTTCTTTTGGGAAAGGCGACATCTCTTGATAACGTT---------

ATAATTGAGGATATGAGGACTCGAGATCGAATAAGCGCTTTACCTGATGAAGTCATTCATCACATATTATCGTTGATGAAGGTGGAAGAAGCGGTTCCAACTAGTGTTTTATCGAAAAGATGGAGATATCGATGGACTTTCATGTCTAAACTCTGCTTCAATTATTACAGTGATGGTTCCAAAGTTTTTGAAAAACTTGGAGACAAATTTCATAGTTACACACCTCGTGGAAATTGTAATATCGCAGCAATTGATGGATGCATAAAACAGTATCAGGCAAAACAACTGCAACAAGTTTCTATTAAACTTTGTGAAAACAAGGATGATGCATCGGAATGCATACAAGAATGGCTCAGATTTGCCACTGAACGGAAAGTTGAAGATGTTCGTGTTGACGCTATCATCGGGGTGAACCCCTCGACCTGGTACAACGTCCCTAACCTTTTGTTTTCATCTCAATGCTTACAATCGTTGTCGTTGGGTCACTGTAACTTATATCCAACTTCTGATATCAAG---TGCTTTGATTCTCTGGTATCTCTATCTCTGACTGACTTTCAAGATTTTGATGGGGCACTGCAATCACTAGTATCTCAGTGTCATAATCTCCGGTTCTTGTACTTTGCGAATCACATTGGATATGATCGCCTTCAACTTTCTAAGCTATCTCTTTCCTTACCTCGGGTGGAAAGTCTTTGCATTGGCTCTGGTTCAGCATATCCTTTAATAGAGATTTTTGCTCCAAATTTGCGATGCTTGAGAGTTCATGGTCCATTCTTTTGTAATCTACGTCTATGGGATTCTCCCCACTTAGAAAAGGTTTACTTGCTCTTTCAAAACTTGTCCTTAACTAAAGTGAGGCAATCTCCCCCCGATCTATCTCCTTACGAAGGTCCCCATTTTTGGGGTTCCGCTTTGCAAATCATCACAAACATTAAATTTTTGCACCTCATGCATGCTGCATTCAAGGTCATGTACACAAAGGACTACATGAGTGGAACAAGAAGATTTACTCTCAACAATCTAAAAGAACTTGTTCTGTATGGCACGCGATTGGATGCTACCGTATATCCCGTTGTGAATTTCTTGAGAGGATGCCCTTATTTGGAGAAGTTATGGATTAGCTATGGTGTTACCCGATCAGAGCCAAATCTCCTTGTCAAAATAAAGAAGTTGGTAATTGAACCCAATGATGACGATCGCAAGCTTTGTTGTGATTTTCATGAATTTTTCTTTCAACACCTCAAAAGGGTAAAGCTGAGCGGAATTCAAGGTGATGCAAAGCAAATGATACTTGTCAAGTTTCTCTTGGGAAAGGCGGCATCTCTTGATAACTTGAGAGTGGAG

2790 CL3053.Contig1_R-bungei CL9467.Contig2_R-chinensis

XCTTTCTTGACGTTTCTTGTCGGGAAGTTACTAGCGACTGCGAAGGATGACGTGCACACCCATTTCCTTACTATTTTGAGTAGTCTTGATGAGATGAGGAAGTATGATTGGGCGGAGGTCATTTACTCTACTTTGCTGGTTCGGCTGGATACGTTCTCGACATCGTCTTTACATTCGGGAGCGATGGGTGGTTTTTACCCAGTAGTAGAGGCTTGGTTCTGGGAGAATTGTCGGGCATTGTACCATGTTGTGCCGGCTGAGGACCGCTACCCTAGACTTACAGCATACTTCGGTTACGGTGTACAGCGAGTTCACAGATCGCTCATTACTCTCCGAGATGCACGTTATCAGATTGAGGCGGCTACAGATAGTCAGATTGTATGGAGGCCTTGGATTGACTCTCCGCATGAGGATGCCACCATTGCAGCGTTACGACTCAGCCGTTCTAGGACTGTGTTTGACTATTGGGGTGAGGGCAGCGCTGTAGTTTACCTTGGAGACCGTCTTTGGAGACAGCTCACTGGTCGGATAGCAATCCCGATATCTATTTCGCTTCCACTCACCTCGATGATCTCGGAGATCAGGGCGGCATACGAGGACGAACTCGGGCAGGATGCAGAGGGACACTGCCTTTACGTGCGAGATGGTTGTTATATTGCTTGGTGGAGGCATGTGTCAGTTGGTCATCTC------------GTTGCTCCACGCTACCGAGATGGCAACGCGGACTACATTACCGATGATGCTGTTAGGGTTGATTTAGAGATTCCTGGCCCATCACAGCACACTGGGCGAGGCGTCCGGACACGAGCTCGTGCAGTCCTTCAGGCAGGTCCAGAGCCTGTGCTACTATCTCCGTATTTTTCTGCACACTCAGCAGATGGTGCCCTGGATACCCTCATGCTCCCCGATCAGCGTTTCATGCTATCAGATGCTGAGCTTGGAGAGGATTTCTCGAGATTGTCGATGGAGGAGCTTACACGTAGATACCGACGTAGCCTGCAGAAGATTGCTGGATTGCAGATTGAGTTAGTTGAGCGTACTCATGACTTGTCCCGCTCCGGTAGCAGTCGAGAGACTCGT

---------------------------------------------------------------------------------------------------------------------------------------------------------------------------------------------------------------------------------------XXCGGGGTAAGCAGAATCCGACCCGAACCCGACCCATTGCCATCCCTA------------------------------------------------ACGGTTAGGGAG---------------------------------------------------------------------------------------------------------------------------------------------------------------GACGGACGTTGG---------------------------------------------------------------------------------------------------CAGGATGCAGAGGTACACGTCCTCTACGTGAGGACAGGT---TATACTACTTGGTGGAGACATCAGTCAGTCGGTCATCTCGGTAGCGCATCTCTTGCGCCACGCTACCGAGATGGCAATGTGGACTACATTACCGATGAT---GTCAGAGTTGATTTAGAGACTCCCGGTCCATCAGCGCACACTAGACGAGGAATCCAAGCAAATGCTAGTGCAGTCTCTCAGCCAGGTCTAGAGTTTATGCTACTATCTCCGTTTTTTACGACAATCACACTAGATGGAGAGGAGGAAACAGTTGCAGTTCCCGATAAGGTTTTCATGATACCG---------------------------------------------------------------------------------------------------------------------------------------------------------

3965 Unigene5984_R-bungei Unigene16884_R-chinensis

CTTTCTGGTGCTAGTGCGAAGTCCAGTTTTATGAATCAAAATGATGGATTCGGAAGCCAAGGTGAGGCATCAGGAGCACCAACAGAGAGTGGTGGGTGGGGTTCTGCCCCTTTGACTACTGAAAGGAGTACTTGGGGCGATTTATCGGCATCAGACAATTTGTTTGGCTCCTCTTTGACAACCCTCTTCAGTTCTACCACTGCGCCTGAAAAAGAGGAAGAAGATGACCCTTGGGGTACCAAAGCAACAGCTACAAAGAGCACAAGCAGCTGGGGCAATGCAACATTATTTGGTGAAGATTCTGGAAAAGCTGGAGGAGATGACTCTTGGGGGACCAAAGTAACTTCTAAGAAACCAATCGATAGCTGGGGCAATGCAACAGGAGGAGACCCCGAA---GGAGGAGGTGACCCTTGGGGAACCAAAGCATCGACTTCCAAAGGAGTTACAGAGAGCTGGGGCAATGCTACAGGTCGAGTCCTTGAAAAAGACGGAGGAGATGACTCTTGGGGAAGCAAAGCGACGACCTCTAAAGAAGCTACAAACAGTTGGGGCGGTGGCATAGGCTCTGCTCTAGAAGAACCTGCAAAAGGCCAAGTTGAAGACTCGGACCCTTGGGGAAGCAAACAAACTCCTAAAAAACCGACAGAATCATCATGGGGCATCCCAAACACCGACGACAAAGGGAAAGATGTAGCAGAAAAAGTCGAAAGTGGATGGGGAAGTGCAGCAATTGTGCAAAACAATGACACCGGAAAAATTGTGGCTGGAAATGATTGGGGAGCAGGTGTTGGAAATAATGGTCAATCAAACCAAGATGATTCATGGGGCAAAGCTACACAAAGTTGGAAGGTGAAAGATGATTCAAGCAATGTGAACTCAGAATGGGGCAAGCCAAATGATGGGGCAACAAATACTGGTTGGAACAGCCAGAAAAGTAGTGACGGTGACCGAGGATTCAACGGGAATAAAAGAGGTATGGACAACCAACTGGATAGCTTGAATAAATCAAGGGATTTTGATGGGGGACGGGGTTCAGGTGGAAGAAGAGGTAGGGGAGGATTCAGAGGAAATGGAGACCAGCCTGGTGGTGGTAGAGGCAGAGGTTTTGGGAGGGGTCGTTCTTCTAATTGGAATAGTGGAAGTCGGGACAATGAAAAGACCAGTAATGATTTTGGGGGCCAAGGATCTACCTGGGGAAATTCC---------TCACAAGGTGCAGAAGAA------------------------------------------------------------------AAAATGAGTCAAGGGGGTGGTTGGGATAACAAAGCAAGTAGCTGGAAAAGTGGGGTAGCCGGTGCAGATGCGAAAAAATCTGCATGGGTGGACGCAAGTGGGGACCAAGGGGCTGGTTGGGGGAATGCTTCACAGGATGATGGGGGAAAGGCAAGTCAAGGAGATGGCTGGGGTAGCAGATCCAGCAGTGGTGCAACTGATGCAGGTGGGAACACCTCAAGTGGCTGGGGTCGGGCAAACAAGTCTGATACTAATCAGGCCTCTGGGTGGGGCAGTGGGGCTGCTGATTTGGGTGCAAACACGCCATCTGGTTGGGGCAGTGAAAGCATTTTAGGTACTCCGTCGAAAAAATCAGATACTAATCAGGCCTCTGGTTGGGGCAGTGGAACAGCTGAT---------------------------------------------------------------------------------------------TGGGGAGCTAACAAGTCAACAGGCTGGGGAAGTGGAGATGCTTCTGGAGACAATGGTGGTGGAAAATCTTATGGCGGGGGAAGGACATCAGGAGGATGGGGTGGAGGAGGACGTGACAGTGACGGGGGTGGTAGAGGTTTTGGCCGAGGAAATAGAGGCAGAGGTCGTGATGGTGATTCC---------------------TCAGATTTTTCTAAT---------------------------------------------------------------------------------------------------CGGGGTAGAGGTGGTTTTGGACGCGGTGGA------------------------------------------------------------------------------------------------------------------

---------------------------------------------------------AGCCAAGAAACATCAGGAGCACCAACAGAGAGCGGTGGTTGGGGTTCTGCCCCTTCAACTACTGAAGGGAGCACTTGGGGCGATTTATCGGCATCTGACAATTTGTTTGGCTCTTCTTTGGCAACTCCTTTTAATTCTACCACTGCGCGTGAAAAAGAGGAAGGAGACGACCCTTGGGGTGCTAAAGCTACAGCTAAAAATAGCACAAGCAGCTGGGGCAATGTAACAGCATCTGGTGAAGATTCTGCAAAAGCTGGAGGAGATGATCCTTGGGGGACCAAAGTAACTCCTGAGAAACCAATCGATAGCTGGGGCAATGCGAAAGGAGGAGACGCTGAAGACGGAGGAGGTGACCCTTGGGGAAACAAAGCATCGACCTCTAAAGGAGTTACAGAGAGCTGGGGCAATGCAACAGGTGGAGTCCTCGAAAAAGATGGAGGAGATGACCCTTGGGGGACCAAAGCGACAACCTCTAAAGACGCTACAGACAGTTGGGTCGGTGGCATAGGCTCTGCTGTAGAAGAACCTGCAAAAGGTCAAGCTGAAGACTCGGATCCTTGGGGAAACAAACAAATTCCTAAAAAATCAACAGAATCATCATGGGGCATCACAAACACTGAGGACAAAGGGAAAGATGTAGCAGAAAAAGTTGAAAGTGGATGGGGAAGTGCAGCAGCTGCACAA---AACGACACGGGAAAA------------AATGATTGGGGAGCAAGTGTTGAAAATAATGGTCAATCGAACCAAGATGATTCATGGGGTAAAGCTGCAGAAAGTTGGAAGGGAAAAGATGGTTCAAGCAAAGTGAACTCAGAATGGGGAAAGCCAAATGACGGGGCAACAAATACTGATTGGAACAGCCAGAAAAGTGGTGACGGA------GGATTCAACGGAAGTGGAGGACGTGCGGACAACCAGCAGGATAGCTGGAATAAATCAAGGGATTTTGACGGGGGGCGGGGTTCAGGTGGAAGTAGAGGTAGGGGAGGATTCGGAGGAAATAGAGACCAGTCTGGTGGTGGCAGAGGCAGAGGTTTTGGGAGGGGTCGTTCTTCTGATTGGAATGGAGGAATTCAGGACAATGTAAAGACCGGTAATGATTTTGGGGGCTCAGGATCTACCTGGGGAAGTACAGGAGGAAAGACCAGTGGAGGGGGGGATTGGGGAAGTACAAGCAGTTTCAACCCTCCCAAAGATACCTCTGCTGACAAAGGGTCTAGCTGGGGAAATTCTTCACAAGGAGATGGCTGGGATAATAAAGCAAGCAGCTGGAAAAGTGAGGCAGCTGATGCAGATGCGAAAAAATCCGCATGGGGTGACACAAGT---------GGATCTGGTTGGGGTAGTGCTTCACGT---GATGGAGGAAAGGCAAGTCAAGGAGATGGATGGGGTAGCAAATCAAGCAGTGGTGCAACTGATGCAAGTGGGAACACCTCAAGTGGCTGGGGTGGGGCAGATAAGTCAGATTTTAATCAGGCCTCTGGTTGGGGCAGTGGAGCTGCTGATTTAGGCGGAAGCAAGTCATCTGGTTGGGGCAGTGAAAGCATTTTAGGTACACTGCCTAAAAAACCAGATACTAGTCAGGCGTCTGGTTGGGGCAGTGGAGCCGCTGATTCAGGTGCAAATAATGAGTCATCTGGTTGGGGCGGTGGGTCCGCTGATACCGGTGCAAATAAGTCGTCTCGTTGGGTGAGTGGAGGCTCTAATTGGGGTGCAAACAAGTCAGTTGGTTGGGGAAGTGGAGATGCTGCTGGAGATAACGGTGGTGGCAAATCTTATGGAGGAGGCAGGATATCAGGAGGTTGGGGTGGAGGAGGCCGTGACAGTGACGGGGGTGGTAGAGGTTTTGGCTGGGGAAATAGAGGCAGAGGCCGTGATGGAGGTGGGTTTGGACGTGGTGGATTTAGAGGTGGGTTTTCAAATGGAGGGCGAGGTGGTAATGATGATGGTGAATCATCAGGTGGAGGACGTGGTTTTGGCCGAGGTGGTAGAGGGAGATTTTCAAATGGGAATGGTGATTCATCAGGCAGAGGAGGTTTTGGCAGAGGTGGTAGAGGGGGATTTAATGGAGGTAGAGGCTGTGGAAGGAGAGATGACTCAGATGGCGGCAGCAGCGGAGGTGGCTGGAGTAAGGGCGGAAGTAATGATGCAAGCGGTGGTGGCTGG

6213 Unigene12667_R-bungei Unigene32131_R-chinensis

GAAACAACAGATACTCAATCACTCCTTCATAAACCGATTTCTCAACTCACTGAAGAAGATATTGCTCAACTCACTCGTGAAGATTGCCGCAAATTTCTCCGGGACAAAGGTATGAGACGTCCATCATGGAACAAATCGCAAGCGATTGAGCAAGTTATTTCTCTCAAGACTTTGCTTGAACCTAGAACTGAATCGGAGAATCAGATTGTTGTTTCCCGGCAGAGAATTCAGGTCTCTCCACCGGAAAAATCAGCTCAGTTGCCTTTGAGTGAGAAAAGAAATGTCGATGACTTGAATCAAATATCGGTTTCTGCAAGTGAGAGTGGTTCTGGTCAGGGAAGAGGCTCACCTGTAGGTCAGGTGGCGGGGGTAACGGAACCTGCTCTTGACAGAAACGCCAACTTGGCCAAAGGACTGGTTGACCAGATGACAATTTTCTACTGTGGCAAGGTCTATGTTTACAACAGTGTGCCTTCTGATAAGGCACTTGCAATAATGCAAATTGCTGCAAGCAATACCACAATGCCTCAAGATTCTCCCGTTAGTGGAAATGCACCAATGCAGCCATTTGCATGCCAGTTACAAGTAGCCTCCATCAGATCAGGACATACCTTTTCTTCTGGCGTCTCTCCATCCATGCACATGGCGACTGAATTTTCCCAGCAATACAGAGATGTCGTAGCTCTTTCACGCGAGGTTGAACCTGAGGGCCCACAGAGTAGAAAAGCATCTGTTCAAAGATATCTGGAGAAACGAAAAGACAGGGGAAGATTCAAGATCAAAAGGAAAGGGGAGTCATCTTCGAACTTAGAGATGTATTTGAATCCTCATCAACCTAGGGATCAAAATCCCAATGAGCAGTCAAGTCAGAGCAGGGGATGTTCCCCTCCCCAACCCAGACCACCTCACACTCCGACACGATGCAGTTCAGTGGATAACCAATCACCTAAAAAT---GCTGCACTCTCCATTGATCTCAAC

---------------------------------------------------------------------------------------------------------------------------------------------------------------------------------------------------------------------------------------------------------------------------------------------------------------------------------------------------------------------------------------------GTGCATGACAGAAACCACAACCTCACCAAAGGGCCGGTTTGCCAGATGACAATATTCTACTGTGGCCAGGTCTATGTTTACGACTATGTGCCTGCTGATAAGGCTTTTGCAATAATGAAAATTGCTGCAAACAATAACCCAGAGCGTCAAGAATCTCCCGCTATTAGAAATGCACCAATTTCGCCGTTTGCATGCCAGTTTCAAGTATCAGCCATCAGATCAACACTTGCCTTTTCTCCTGGTGTCCCTCCATCAATGCACGTGATGACTGAATTTTCCCAGCAATACAGAGAGGACTTGGCTCTTTCACGCGAGGTTGATCCTGACGGCCCACAGAGCAGAAAAGCATCTGTCCAACGATATCTGGAGAAGCGAAAGGACAGGGGAAGATTCAAGATCAAGAAGAAAGCCGAGTCATCTTCAAACTTGGACACGTATCTGAATCCTCAACAACTTAGGGATCAAAATCCAAATGAGCAGTCGAGTCAGAGCAGGGGATGTTCCCCACCCCAACCCATACCGCCTGTCACTCCTTCCAGATGCAGTTCAGTGGATACCCAACCACTCAAAGATGCTGCTGCAGTATCCATTGATCTCAAC

6172 Unigene30934_R-bungei Unigene31771_R-chinensis

---------------------------------------------------------------------------------------------------------------------------------------------------------------------------------------------------------------------------------------------------------------------------------------------------------------------------------------------------TCCACCCAATCACGTGAAAACAGTACTCGGGATCCTATTCTGTCGAAGAAACTACACGATGGCAAGGATTTACTTGCGGAGCAAGCAAGCGAGTGCGGCTTTAGTTGGGATCATATGATTCTGCCAAAGAAGGAATGTAATAATGGGTGGGGTAAGCAAGAAGGGGTT---GCCATCCAATCAAATGAAAATGATGGGTGGGGCAATCGTCAATCAGTTGCCACACAATCAAGTATTCCATCAAAGAAAATAGATGATGGCAAGAATTTGCTTGCGCAACAAGCAGGGGAAGAGAATAGTTGGGACCATATCATTCCTCCAAAGAAGGAATATCATGAAGGATGG---------GAATCTGTTTCCATTGAATCAAGTGAAAAGGATGGTCCCCATCCTATTTCGTCAAGGAAAATAGATCATGGCAAGGAGGATTTACTCGTCAAGCAAGCAAGTGGACACAAAGGTGGTTGGGATCACATGATTCCTCCAAAGAGGACCAATTATGATGGGTGGGGTAAGAAATCAATTGCCATGCAATCAAAGGAAAACAATGTGGACAGGGATTCTACTGGAAAGAAAGTAGGTAAGAAATCGAATGAAGACGACAACAATGGACGTGATCTACGTTGGGATCTGACTCTTCCAAAGAAGGAACATTATGATAAACGGGCTACAAATTCTATTAGCCCCACACAATCAAATGGACGTGACCTTTGGCGGGACCATATTCGTCCAGAGAGGGAACCATGTCATGTATGGCGGAGAGATTTCACCACCACAAAATCAAGGGAGGAGAATGCGCGTAGGGACCTACCCGCTATCCGTTCAAAGAGCGAACCACGTAATGGTAAGGGAAAGGATTTCGCTTCCACACAATCAAGGGAAGAGAATGCACGTAGGGACGCCCCCATTCGTCCAAACAAGCATAATGAACGGGAACATGATATTGGTACTAGGAGAGAACATAATGACGGC

GGATATTCCCGGTCAGATGCTCCTCCTCCTGGATATTCACGGTCAGATGATGCTCCTCCTGGATTTTCAAGACCACTTACTACCAATGCTTCTGATGACAGTCACATTGTTCCACCACATCCTAAGATGTCACATGTTGCTAAAGAAGTCAATACACCTGGTTCATCTAACTGGACTAAGCTCAGAGAGGAGGTCTCATCATCTAAGGTAACTGCCAGCAAGGATTCTAGGGCTACACCCTCCAATGGAAACAATGGACGACGCGTACACCCTATAGTGAAAACAGCGGACCTTCCGGTTTGTTCATGGAAGGAAGGCCATGATGGCAGGGCACGAGTCTCTACCCAACCACGTGAAAAGAGTACTCGGGATCCTATTCCATTGAAGAAACCACATGATGGAAAGGATTTACTTGCAGAGAAAGGAAGTGAGCGCGACCGTAATTGGGATTATATGATTCGGCCAAAGGAGGACTACAATGATGGGTGGGGTACTCCTCGATCAGTTGCTGCCATCCAATCAAATAAAAATGAGGGATGGGGAAATCCTCAATCAGTTGCCACACAGTCCCATATTCCGTCAAAGAAGTTAGGTGCTGGTAACGATTTGCTTGGGCAACAAGCAAGTGGAGAGAATAGTTGGGACCATATGATTCCTCCAAAAGAGGAAATTCATGATGGGTGGGGCAATAAGGACTCTGTTTCCATTGAACGAAGTGAAAGGAATGGTCACAACCCTATTCAGTCAAGG---------------AAGGAAGATTTACTTGGCAAGCAAGCAGAT------------GGTTGGGGTCATATGATTCCTCCAGAGACAGCTAATCATGATGGATGGTGTAAGAAA---------------TCAAATGAAAAGAATGGGGCAAGGGATTCTGCT---------------AGAAAGGAATTCAATGTAGACAACAGTGGACACAGTTCACGTTGGGACGCGACTCATCCAAAGAACGAACGTTATGATGGATGGGGTACAAATTCAGTCACCCCCAAGCAATCAAATGGACGAGATCTTCAGTGGAACCCTATTCATTCAGAGAGGGAACCTAGGCATGGAAGGGGGTCAGATTTCACCACCACACAATCAAGGGAGGAGAATGCACGTAGGGACCTCCCT---ATTCGTTCAAAGAGTGAACCACGTGATGGGCGGGGTAAGGATCTTGCTTCCACACAA------------CATGCACGTACTGATGCCCCTATTCGTCCAAACAAGTATAACGAACGAGAACGTGATAGCTGGCACAGGGATCGTCGACGAAAT---

767 Unigene34148_R-bungei CL3230.Contig2_R-chinensis

------------------------------------------------------------------------------------------------------------------------------------------------------------------------------------------------------------------------------------------------------------------------------------------------------------------------------------------------------------------------------------------------------------------------------------------------------------------------------------------------------------------------------------------------------------------------------------------------------------------------------------------------------------------------------------------------------------------------------------------------------------------------------------------------------------------------------------------------------------------------------------------------------------------------------------------------------------------------------------------------TTAGGTCGTTGCCGTAATGTGCGAAACCGTGTTCAGGTGTTACGTCAAAATTGGGATGCCTTCAGAAATGGGTCGTTGGTCTTTTCTAATTTATTTAATTCTGATGCACAAAACAAGAGGAGACGCAGTACAGAAGAAATTAATGACAGGTCAAGTAGAGCACAACCATCATCTTCCCCGAACACTACCAAGGATAATGCTTCTGGTGTCAAGTTAGATAAGAAGGGCCGGTATGATGTTGATAGAGCCTGGAAAATGTTGGATATTGCAAATTCAGTTCAAAGACCTCGCAAGTGCACCCGAAGCAGTCATCCGATATCTAGTCCTCAACTAGGTAAACGGAACGTGTCGAAGGCAGCAAATAATACATGCTCAACCCCTCCAATGTCTAGCAGCAGAAGTTTTCAAGCAGGTCCATCTCAATCTTTTGATCCTTTGTTAAAACAGGCCCGTTTAGGAGTTCAAGGTCCAAAGTTAGTACAAAGAGGTCACCTGGGAAACCAAAGCGGTTCACATGATTCTAGTCATCAAGTAGGTAAACAAGATGTGTTAATGGGGGCAAATAATACAAGCTTAGCTGTTTCCATTTCTAGAAGCAAAAATTTTCAACCCGAGGATCTTAGGCTTGAACTTGAGACGAACATTAGGCACCAATGTCTTGAATTAGAACAACAGAAGTTAAAGTCTGGTCAGAGAGGGATTAGTTTGGATACCTTGAGGACATCATCATGTTCGAGGCCTCCCAGTGTT------------------------------------------------------------------------------------------------------------------------------------------------------------------------------------------------------------------------------------------------------------------------------------------------------------------------------------------------------------------------------------------------------------------------------------------------------------------------------------------------------------------------------------------------------------------------------------------

GAGATGGACTCCAATTCCTCCCCTAACAAACGGCAGAAAACACAATCTCCACCCTCAACTCCTCCTTCATCATCTCACAAAGGAAAACAAAAGATCATTGAAGAAGAAGAAACCAAAAATGAATCTCCAAATTCAGTCTGCGGAATTTGTTTCGAAGGAAAATCAATCCGAGGATGGATTGATAGCTGCGATCATCACTTTTGTTTCGTTTGTATTATGGAATGGTCTAAAGTTGAATCTCGTTGTCCTTTATGTAAACAAAGGTTCAATTCTATTACCAGGAAACCTCAAAACGGAGTTTTGGTGATGAATCAAAGGGTTGTTAATGTCCCCGTTCGCAATCAGGTTTGGCATCCTCTAGGAAATCAAACAATTACTGAGCATTCTGATCCATATGCTGATGTCAAATGTATTAAGTGTCAGAATTCATCTGATGACCATCTGTTGCTACTTTGTGATCTTTGTGATTTAGCGGCTCATACTTTCTGTGTGGGTTTGGGGTACACTGTGCCAGAGGGAGACTGGTACTGTGAGGATTGTACAGTTGTGAAGAATGAGCACTCTGATGGGGAAACTGATCCTGCCTGTCAGTATTCAACGGCTTCTTTCAGAACTATGGAAGTTGAGAACAATCTTCCAGACTCGAGTTATGGCGTCACAACCACTGACAGTGATACTGACTATTTGGATTCAAACAGTTTTATCTCAGCTCCGAATGTTTCTATTTCTGATATTATACGGCAATCACGTCCACGCCAACAGCTTCGTGATAGATTAACACCGAGAACCTCTAGGCGTCAATCTGTTTTATTATCTCATCGGTCTGACAGAGGCATCGTATCAGATAGTGTAGGGACTGATTCTAGTGCAAGTGGTATCATATCAGACAGTGCAGGGACTGATTTTAGTGCTGCAGGCATAATCTCAGATAGTGCAGGGACTGCTTCTAGTGCTAGGACCTTAGGTCGCTGTCGTAATGTGCGAAACCGTATTCAGGTCTTACGTCAAAATTGGGATGCCTTCAGAAATGGCTCTTTGGTCTTTTCTAATTTATTTGATTCTGATTCACAAAATAAG---AAACGCGGTAAAGAAGAAATTAATGACAGGTCAACTAAAGCACAACCGTCATCTGCCCCGAATACTGCCAATGATAATGCTTCTGTTGTCAAGTTGAATAAGAAAGGCCGGTATGATGTTGATAAAGCCTGGAAAATGTTAGATATTGCGAATTCAGTTCAAAGACCTCCCAAGTGCAACCAAAGCAGTCATCATATATCTAATCACCACCCAGGTGAACGAAATGGGTCGAAGTCAGCAAATAATACAGGCTCAACCCTGTCAATGTCTAGAAGCAGAAGTTTTCAACCAGGTCCACCTCAATCCTTTGATTCTTCATCAAAACAGGCCTGTTTAGCAGATCAAGGTCCTAAGTCAGTACAAAGAAGTCATCTGGGAAACCAAAGCAGTCCTCACAAACCTAGTCATCAAGTTTGTAAACAAGATGTGTTAAATGCAGAAAATTATACAAGCTTACCTGTTTGTGTATCTAAAGGCAAAAAGTCTCAACCAGAGGATCTTAGGCTTGAATGTCAGGCTAACGGTTACCACCAAGGTTTTGAATCAGAACAACAAAACCGAAAGTTTGTTCAGACAGGGATTAGTTTGGATACCTTGAGGACATCATCATGTTCGAGGCCTCCCAGTGTTAATCCGTCACAGTCTTCTGGGCTTTCATTGCCAATTCCCACTCATATGCTGGATCAGGATGATCTTTCCGAGAAAAGAAGAAGTGATGAGATTAGTGCCTCTCCAAGCTTAATTGAAAGGAGAAGTATTACTCGGTCAGCATCTCAAACAGAGTCCCTTTGGGGAGGCTCCAAACGATTGCAAAATAGATTAGAGTCGTGCGCATCTTCCATTTTGAAGGAGAAACAATCTAAGGAAAGGGGAAAGGAAAAGAGTTATTCCAAAATTTTATCAAAGAAAGATAATGATGCCAAGTCTGAAGTCCAATCATTGGTTAAGCTCAACCTGAAATTGCTAAACAAAGATAAACAACTAGGGGTTGATGGATTCAAGGAAGTTGCAAGGCTTGCAACTCATACCATACTGGCAGCATGTGGATTTGAGCATAAGGAGTCCAGTGTCCGCTCCTTTCCTAACACAATATGTCGCCACAATGATCAGACCAAGCACCTTCACATGTCCAATCTGATGCCAAGTGCTTGCAGAGAATGCTTTTATGGATTTGTAAAAGATGTTGTCAGCTCCATCATG

326 CL1975.Contig1_R-chinensis Unigene28849_R-bungei

------------------------------------------------------------------------------------------------------------------------------------------------------------AAGAAACAATATATTAGTGTTCTTCAACAATATGAGCAAGTTTATTTCTTCAGGAAAAATCTTTCTGTGGTTACTAGACCTAAACCAGTGGTACCAATTAGACGTGCAGTAGCTTACGGAGGTGAATCATCTTCGAATCCTATACTCGTGGGAGAAACAAAGCAAAAGAAAGTTTCAGGA------------------------------------------------------------------------------TCTGAGCTAGGTACTGTGATGGGGAAAATAGATGGCAAGTTTGACCAAGGTTACTTAGTGACAGTAACAGTTGGGTCAGAAATATTTCATGGAGTGCTCTACCACCCTGCACATCCTGAAGTGTTCTCACCACCTTCAACCAACAACGTGGTTAGCAATCCTTGTAATCCAGGCCCATCGAGGAACCCCCCTCCCGGTTTTCCTCGTACTTCAGAACAACCGTCACGTCGTAGGAAGAGGAAG---CGCCGCAGAGATGTGGACCCCACACGTCCAAAATCAAGCAGGAGTGCCTACAACTTTTTCTTTGCTGATAAGCACAATGAGTTTAAAGCACTTTACCCAGATAGAGAGCATAACTTCACCAAAATGATTGGCCATGCTTGGAACAATCTGACTGACGAAGAAAGACATGTATATCAAGAAATTGGGAACAAAGATAAACAAAGGTATCAGACGGAAATGAAGGTGTATGAAGAGAAA

CCTGTAATTGGAGGAAAGGAGCTAGATTTGCATTTGTTGTATGTTCAAGTTACTAATAGAGGTGGATTTGACAAGGTAATGGGAGAGAAGAAATGGAGAGAGATAAGAACAGCATTTGATTTTCCTTCAACTGCAACAAGTGCTTCTTATGTGTTAAAGAAACAATATTTGAGTGTTCTTCGACAATATGAGCAAGTTTATTTTCTCAACAAAAGTTTTTCTTTGATTACTCAAGCTTCACCAGTGGCATCATTTCGACCTGCAATAGCTGACATAAGTGAACCGGCTTCAAATCCTATACACATGGGTCAAACAACACAAAAGAAAGTGGCATCATTCAGACCTGCGATAGCTGACATGGGTGAACCAGCTTCGAATCCTATACACATGGTACAAACAACAGAAAAGAAAGTATCTGAGCAGAGTACTGTGATGGGGGAAATAATTGGCAAGTTTGAGCATGGTTACTTAGTGACAGTAACAGTTGGGTCAGGGATATTTCGTGGAGTACTCTACCACCCCGCACAGCTTGAATCTTTCTCACCGCCTTCATTCAACAAT---------------AATAATCTAGGCCCATCA---AGCAGCCCTGCTGCTGTTCCTTGTAATTCAGATCAACCCTCACGTCGTAGGAAGAGGAAGCGCCGCCGAAGAGATGTGGACCCCGCACGTCCGAAATCAAGTAGAAGTGCTTACAACTTTTTCTTTGCTGCGAAACACAATGAGTTCAAAGCGCTTTACCCAGATAGAGAGCAAAACTTCACCAAAATGATTGGCCATGCTTGGAACAGTTTAACTGACGAAGAGAGACATGTGTATCAAGAAATCGGGAACAAAGATAAACAAAGGTATCAGATGGAATTGAAGGTGTATGAGGAGAGA

3154 Unigene10862_R-chinensis Unigene12383_R-bungei

AAAGAAATTGAGGAACCAAAGACAATTGAAAAAGAAATTGTTTCCTCGCCCATAGAAGAGAAGGCGATTCCTGAAGCTCCTCTGACAGAATCAGAGGTGAAGTCAGATGAAAAGACAATTGACCAACCTGCAATTTTAGAGGATGAAAAGGCTGCTGATGCCGCTGTGTTGGAGACCCCAGCTGAAAAGGCAGATGCTATTACAGAAGTCATTGATTCAAACGAAAATGTTTCTTCTGTACCAGCTCCTGAGACTAAAGATGTTCCTGAACCAGCAGTTGAATCAGTCGAGAAACCATTGGAGTCACCTCCTATATCAGAACCAGCAGTTGAATCAGTTGAGAAACCATTAGAGTCACCTCCTACTCCAGAACCTGCAGTTGAAGATGCTGAGAAAGTGATATTAGCACAAGATGCTCCTGAACTAGCAGTGGAGACACCATTAGCTTCCCCTCCTACTGAGGAACCACATTCTTTAATTGAAACCACGGTAGAACAAGTTTCTGAGGAGTCAGAAACTAAAGAAGTTGAAAGATCAGAATTAGCTATTCCAGAACCAGTAGTAGAGAAACCAGATGTAGTCGCAGAAGAACCCGAAGTAGAGATACTGTCAGAATCTAAAGAAGTTGAAAAATCAGAATTCGCTGTTCCAGAACCAGTAGTTGAGAAAACATTAGACTCACCTCCAATTCCAGAGCACGTATCTGAAGTTCCAGAATCATCCGTCACAGATACAATTGCACAAGAACCAGAAGTAGTGATATCATCAGAAATTAAAGAAGTTAAATTTTCAGATGTACCTGTGGAAGTACCAATAATAGTAGAAGAAGTTCTAGAACCATCTGTCAAAGATGCTGAAAAGGAGACATTAGCACAAGAAGTTGAAATTCCAGAAGCAGCCATAGGGGTCGCTGAAGTAGCGCAACCATTAGAAGAAGTCTCTGTTTCAGATTCTAAAGTGGTAGAATCAGCAGAACCAGTTGTAGTGGTAGAGGAGAAACCACAAGACCACTCAGAAATTCTCGATGAAGTTGAAAAGGAAACTGACGAGAAGGTCGAGCTAACTGAGAATGTACTTGTGAAAGAGGAAGAGTTGCCAGCCAGTGAAGTTAAGCAGGCTACTGAACCCTCAGTAGAAACAGAAACTAGTAAAGGTACACTTGCTTCTGAAACTCCATTGGCTCCTGTAGACGAAGTACCAACTGCTATTCCTGAACTCAAAGAGTCTGCAGAAGGGGTACCTCTCGAGGACATTACAATTGAAGAAAAGAAAACAGAACCTGAGACGACTATTGAGACAGAAACCAGTAAAGATACAGTTGTTTCTGAAAGTCCATCGGAGATGACTGTTGAAGTTACAGAAAAGCCTCTGGACATTGCTACCAAAGAGGAGAAAGAAGACGAGTCGTTGACTGCAAAAACTGAAGAGGTAGTTGATCCCGTT---------CTTATTGCCGAAAGATCTTTCCAAGTGGAAGAACCATCTAAAGATGTTGAAGTGGTAGCAGAAGAGAAGAAGGAAGTTAATAATGCTCCGAACGAGACTGAAAAGACAGCCAAAGAGGTTGAGACCGAAGAAAAGAGTACTCCTGTAGAAGACAAGAAAGCAGACGACAAAACAGTGGAT------------------------------------------ACTGAGGAGAAGACTGATGTCATTGTCGATGACGTGAAAACACAGGAAGTTCCCAAAAAGCAATCGACTAGCATCATGTCAAAGGTAAAGCAGTCGCTTGTCAAAGTAAAGAAAGCGATTGTTGGGAAATCGCCGAACTCGAAAACTGTTACTGCAGATGCAAAGGAAGATATAAAGGTA

------------------------------------------------------------------------------------------------------------------------------------------------------------------------------------------------------------------------------------------------------------------------------------------------------------------------------------------------------------------------------------------------------------------------------------------------------------------------------------------------------------------------------------------------------------------------------------------------------------------------------------------------------------------------------------------------------------------------------------------------------------------------GAACCAAAAGCAGAGATCCCATCAGAAATCAAAGAAGTTGAAAATACAGATGTAGCTGTGGAAGTACCTGTAATAGTAGAAGAAATTCTAGAACCATCCGTCAAAGATGCTGAAAAAGAGACAATAGCACAAGAAGTTCAAAGTTCAGAAGCAGCCGTCGAAGTAGCTGAAATAGATAAACCA---TCAGAAGTCTCAGTTTCAGAGTCTAAAATAGTAGAATCAGCAGAACCTGGTGTAGGGGCTGAGGAGAAACCACAAGAACACTTGGCAACTCTTGATGAAGTTGAAAAGAAATCTGGCGAAAAGTTTGAGCCAACTGAGAATGTACATGTGAAAGAGGAAGAGTTTCCGAGCAGTGAAGTTGTGGAGGCTACTGTACCCTCAATCAAGACAGAAACTAGTAAAGTTACACTTTCTTCCGAAACTCCCTCTGCTCCTATAGAAGAAGTGCCAACTGCTATTCCTGACCTTAAGGAGTCTGCAGAAGCCGTACCTCTCAAGGACATTAAGGTTGAAGAAAAGAAAATGGAATCTGAGACGGCTGTTGTGACAGAAACTTCTAAAGATAAAGTTGCTTCTGAAACTCCACCAGAGATGGCTGTTGAAGTTACGGAAAAGCCTCAGGATATTGCCATCAAAGAGGAGAAAGAAGATGAGCCGTTGATTGCCAAAACTGAAGAGGTAGTTGATCCCGTTCCTGCTACTATTGTTGTTGAAAGATCTTTACAAGTAGAAGAACCATCTAAAGATGTTGGAGTGGTAGCTGAAGAGAAGAAAGAAGTTTATAATGCTCCGACTGAAACTGAAAAAACAGCTAAAGAGTCTGAGACAGAAGAAAAGAGTACCCCTTTAGAAGAGAAGAAAGTAGACGACAGAACTGTGGATAATGCAAGCAGTGAACCAGTGTCGGAGCCCAAGGAAGCAACAGCCGAGGAGAAGACTGATGTTACTGTTGATGACTCGAATACAAAGCAAGTTCCCAAAAAGCAATCGAATAGCATCATTTCAAAGGTGAAGCAGTCGCTTGTCAAAGTAAAGAAAGCAATTGTTGGGAAATCGCCGAACTCGAAAACTGTTACTACAGATGTC------------------

3701 Unigene15071_R-chinensis Unigene30374_R-bungei

XCAAGTTCATGCTTGTTTATTGTTCTACTTATCAGTGGGGCGATATGGCAGACTAGTTACAGTAGAGGGGGATATGCCTCTACACTAGCACTGACAAAGGAGAAAACTGAT---AACTACATCTATGGCGACTTTTCTAATGGCCTGATTTACAGTATGAAGGCTAATCGAGGTAGAAGAATTAGGAGGCAAAGGCAACCTGCTTCACCTTTTCCAAACAGGAACGGGCATATGTCTGTACCTCCATCAACACCAATTTTTCCACGCTCTCCTCCTGCTGCATTTAACCCTCCACCCTTCCCTCCACXX

---XGTTCATACTTGTTTTTTGTGTTGATAATCAGTGGGGCGATATGGCAGAATAGTTACAGAGGAAGAGGATATGCATCTGCACTCGCAGCGAGAGAGAAGACAACTGAGAACAACTACATCAATGGCGACTTCTCTAATGACCTGTTTAACAGTGTGAAGGTGAATCGAGGTAGAAGAGGTAGGAGATCAAGGAGACCTGCTTCACCTTTGCCTAATAGGGCTGGTCATATGTCTGTACCTCCAACACCACCCCTTCCTCCACCTTGTTTTCCTGTAGTTGACACCCCGCCCCCX------------

134 CL2525.Contig1_R-bungei CL1340.Contig1_R-chinensis

---------------------------------------------------------------------------------TTACCAGATGGGATTGGAGGTTTGATTAATCTTAGGTACGTGGACCTGAGCTCATGCTCTAAGTTGATACACCTGCCACAGCGAATTGGAGAATTGAGCAACCTTAGACACCTGAATTTGAGTGGGTGTTATGAATTAATTCAAATGCCGATAGGGATTGGCAAGTTGAGTTGGCTGGAGACACTAGAGAGGTTCGTTGTAGGCCCAGAAGACAGT---GGAGCGGATATCACAGAATTGCAAGGACTAAACCATCTACGAGGATATCTGGAGATACAGGGACTTGAACATGTGAGAAATGGAGCAGAGGCCAAACAAGCCAATTTAACGGCAAAGCAAAAACTTTCTTCTTTGAAGCTGCAGTGGTGGCGG---GACATCTGCAGCAACAGTGAGGATGTGATCCAAGGGTTTCAACCTAGTCATGAAAGTTTAAAGGAGTTATCTATTTCTGGATATGGAGGCTTAGTATTTCCGAGTTGGATA------------------------------------------------------------------------------------------------

CTTCAAAAATTGCCCAAATCTATAAGCAAGTTGGTCAATCTTAGACACCTGAACTTGAGGTGGTGTAGGAGATTAATCCAATTACCAGAAGGGGTTGGAGATTTGATCAATCTTAGATACATGGACTTGAGCCATTGCTATGCGTTAATAGAGCTACCGCAGCGAATTGGAAATTTGGTTAGGCTTATACACCTGGACTTGCAGAATTGTATTAGATTAACTCAAATGCCAATTGGGATGAGCAAGTTGAGTTGCCTTGAGACGCTAGGGACATTTGTTGTAGGCCGAGAAGACACTGGCGGAAGCAATATCACAGAACTGCATGGACTAAACTGTCTAAGAGGGAATCTAAAATTAAGGGGACTTGAGCACGTGAGAGATGGAGTTGAGGCAGAACAAGCAAATTTAGCGGCAAAGAAAAACCTTTCTGATTTGGAGCTGAGGTGGTCCCGGGGGAAGATGAGCAGAAACAGCGAAGATGTGATCCAAGGGCTCCAACCATGTCAAGAGAATCTTCAGAAATTGTTTATTGAAAATTACGGTGGAACAATATTTCCAAGCTGGATGAAACTTCTCGTGAATTTGGTTGAGATCATGCTCTGGTTTTGTTCCAAATGTGAAGACCCTCCACCACTCGGCCACCTTCCGCTCCTGAAGACCCTA

34 Unigene1947_R-bungei CL1068.Contig1_R-chinensis

---------------------------------------------------------------------------------------XTAACCTTGCAACCTGCCTATACCTCGTTCGAAGCTCTTCCTGAGGGAATTGTAAATGTTTGCGAACAGAAAGTCCAAGTGGAATCAGAGGTATCTCCATCAACTGACACTGGAAAACTAACCACTGAGATGGAAGCTCAAGATAATTCTGGACATACTTGTCCTGGCTCTGTATCAAATGATTCAGATATAAGTGCCAACTTGAATACTTTAGATACAGGGGATCTTGAAAAGAGCGAACAAAAGGAAGCAGTGTGCAGCAGAAGAATGTCTGATAATGTTTCCACAGGACAAGAGGCTGCCTTTTCTGAAGGTAAGGATGTAAGCGAAGGX---------

XXTGATTGTGCAATCCCATCAAATGTGGATCACTGTATTGATTGTATTGATCTTAACAATGTTAAACAAGTCAACAAAAGTCTGCTTGTACCTGTGCAACCTGCCTATACCTCTTTCGAAGGACTTCCTGATGAAACTGTGAAGGGTTGTGAAGAAAAGATCCAGGTGAAGCCGGAGGTATCTCCAACAACTGACACTGGTGAACTGACAACTGAGATGGGAGCTCATGATAATTCTACTCATCTG------GGTTTTGCATCAAGTGATTCAGGTGAAACTGCCAACTTGAACCCTTTGGGTGCAGGGGATCTTGAAAAGAGCCAACAAAAGGAAGCAGTGTGCACCAGCAGAACAACTGAAAATGTTTACACCGGACTGCCTGAGGAAACTGTGAATGGCTGTGATGAAAAGGCCCAAGTGAAGTCA

9703 CL2066.Contig1_R-bungei Unigene41890_R-chinensis

---ACATCTACATCGAGAGGAGAAGCAGGAAAGATTTATTACAATCACACCGATTCGTGCAATTTATCAAGATGGACGACAAAAGAATGTCACTCATTCATGTACAATGGTAGAGCTTGGCAACAAGTTTCTGATTTCTATTCCAGTTTAGTAAACGGAAGATATTCACTACCTCTCCTACTATTCCAATTCCAAAAACAGAGGCCTTTGAACACGGAGCCAAGAGTTAAT---------AATGAAGCTATGGAATCTGAGTTGGAAGGCGTCTCTATGGAAAGTAGAAGCGGC------

CAAATTCAAATTCAAATTCAAACCAAGGATTATTCTCATTATAATCACATCGATTCCTGCAAATCATCAAGATGGACGGCTAGAGAGTGTCATGAATTCATGTACAAT---AGAGATTGGAAATATGTTGGTGATTTCTATTCTAATTTAGTTCTTGGAAGATATTCACTTACTCTCTTACTCTTCCAAATACAA---CAGAGGCCTTTGTACTCGGAGTCAAGAGTAAATGAAGCTACGGAATCTGCTGAGGATTCTGAGTTGGCAGGCGTCTTTACGGAACGGAGAGCTGGGAGATGG

605 CL2794.Contig3_R-chinensis Unigene14569_R-bungei

TATCAATTAAATCAGGTATGGGCAATTTGGGACGATGAAGTGGACGGAATGCCTCGTTTGTATGCTCGGATACAAAAACTCTTGCTTGCAGAACAGAAGGTTGAGGTTTCTTTTTTGGAACCTGTAGCATTTACAAATGAAGAAAACCAATGGTTGTCTGAAAAGAAATATCCTATGGCGTGTGGGAGGTTTAACTTAGGTAATTCGACGAGCATTATAGATGTTTCTGATTTATCTCATAAAGTTAATCTTGAGAGAGATGTGCCCAGTGAAGGGATAAAGATTTTCACTAATATAATGGAGGACACTTGGGTATACATTTTACCTAGAGCTGGTGAGATTTGGGCAGTGTTCAAAGATTGGAATGATAATTGGACACTTCATGATTTGAGTCATGCCCGGTATGAGCTGGTGGAAGTAGTATCTCGGTTT---ACTAAGAGGTCTGGTATGACAGCTATCTGCTTAACCAGAGTGGGCAAAACTGGAGCAGTGTTTGGGAGAGGGTTCCCTAGGGAAGAAATGAATTTGCGGCTCTATACGCGAAATCAACTATTCCGATTCTCCCACTCTATTCCTGCTTCCAAATTGCAAATTGAGGAAATGGAGGGTGTTCCGAATGAGGCATGGAGTCTCAACCCTGCAGCTGTGCCA------------

---------------------------------------------------------------------------------------------------------------------------------------------------------------------------------------------------------------------------------------------------------------------------------------AAGATTTTCGCAAATGTATTGGAAGAGACTTGGGTGGTTATTTTACCTAGAGATGGTGAGATTTGGGCAGTGTTCAAAGATTGGAATGCTAATTGGACACTTCATGATTTGAGTTGTGCTCGGTATGAGTTGGTGGAAGTGGTATCTCAGTTTCCTAGTGCGAAGTCTGACATGACAGCTATCTGCTTAACCAGAGTGGGCAAATCTGGAGCTGTGTATGGGAGAGGGTTCCATGAGGGGGAAATGAATTTGCGGCTCTATTCGCAAAAGCAACTATTGCACTTCTCCCACCGTATTCCTGCATCCAAATTGAAAGTTGAGGAAATCGAAGGCGTTCCTAATGAGGCATGGGAGCTCAATCCTGCAGCTGTACCCCTTCATCTTCTT

5075 Unigene25660_R-bungei Unigene24441_R-chinensis

ATTTCGAAAGGAATTGATGACGATGATGATAATGATGAAGTGTCGTCTGATTTTGATTCGGAGTCGGATAGGGCGGAGAGTTCGTCACCTGATGCTAGTATGGCAGATATCATGCCAATGCTTGATGAGCTCCACCCGTTATTGGATTTGGAGATTTCACAGCCTGCTCTTGTGTCAGTGGAGGATGATGATGATGTTTCCGAGGAGTCAGTTAGCTCCACTGATGAGTCGGAGGAGTCAGAGGAGGAGACTGAAAATGTGGAGATAGAAGATGAAGATGTGGAAGAAGAAGAAGAGGAGGAGCAACAACAAGAAAATGATGATGGAACGGAAGCTGTTGTAACGTGGACAAAGGAGGACGAAAAGAATCTAATGGATCTTGGTAATTCTGAGCTGGAAAGAAACCGGCGGTTGGAGAGTTTGATTGCTAGGAGAAGGGCAAGGAATAGCTTTAGAATGATGATGACTGAGAAGAATTTGATAGATTTCGAGGGTATGGATCCCCCATTTTCCATGGTGCCAATATCAACTGCAAGGCGGAATCCTTTTGATCTTCACTATGATTCTAATGAGGGAATTGATTTGCCACCGATTCCTGGATCAGCTCCATCTGTTTTGGCACCAAGGAGAAATCCTTTCGATATTCCTTATGACCCACTTGAAGAGAAACCAAATCTTCAAGGGGGAAGCTTTGATGAAGAGATCTTTTCAAACCTCGAGAAGGATATGCACTTCAGGAGGTATGAGAGCTTTAATGTTGGACCTTCTTCCCTAGGAGAATCCATGCAAGATAAGTCGAGCAGAAAGTTGAGACCTTATTTTGTACCAGAACCATTGCACTCAGAAGGGATTGGTTATTCCACTTTTCAAAGACAAATGAGTGAATTAAGTGAGTCCAAAATGAGTTCTGTTACGGAGGTTGAGTCAGTTTCTTCAGTGTCAGATCACGATAATCACAAGAATATCGCCGAAGACGAATCATTCCAATCTATTTCCCATGTTGAACAAGAAGTGTCTACCTCGATGTCCCATGTTGAGCATGATCGCGATCGTATTGAACTTGAAAGCTCATCCTCAGAAGAAATAGACATGTCTGAGGTTGAAGGAGATCAAGAATATCATAGAGGTGCTACTGTAGGTGAAATTGAAGGAGAATTATCCCAACCAATCCCTCGATCTGAACAAGAATTGTTTCATCCGATATCCCATCTTGAAGATGCTCACGACCATGTAGAAACAGAGGGCTCAATTTCTGAAGAAGTAAACATAGCCGAGGTTGATCAAGAATATCAGAGAGATGTTATTGTAGACGAGGAAAATGCAACAGAATATACTTCCGTCGAGATGCAATCCATTCCTTCACCTGTGGGGTCTGATACTGGTGATGTGACAGCTGATGTAGTCGAAGAGAAGCACACAGAATTGAGCTCTTCCTCTTCATCTGAAGCGAACACATGTGAAGGATTAGACAGCTCAGAGCAGAAAAGCGATGATAATATACTTAATAATTCAGCGGAACCATCTATTGAGGGTTCTGGTCTCCGGAGTGAACTTGTAGAAGTAGAAGATATTCATGCAAAGGGACCTCTATATGATTGGAATTGGAACCCGTCAGAAGTAGAGAAGACACTTTCTGGCATATCAATTATTGAAGAAGCTTTAAATTATGTGGATAGAAGAGCTCTAACTTCTACTTCATCGGCAGCATCAGACATAGCTGAAGAAGCTGGGGAAGTAGGTCCAATTCCAGTTCAAGTTGAAAGAACCATTTCATTTATGGACGAGGAACCACTGCTGCATAATGGGAGTATGGAAGAGGTTTCGCATTCACATGCAGTTGATGTGAACAATTTAAGCTCTACCGAAGTAACTGAGACTAATGAGCATGATGTTATTGAAGCTGCAATTTCTGAAGATATCCAGAATGAGTCCAACTTAACTGCTCCTGTGACGCCGTTAGTAACTGAGCAAGTCATATGTGGCTCAGGTTCATCTTCATCAGAAACAGAATTTGAAGATGGAAGTTCATTGAGCAAGGAAGAAAATGAGCACCAACAAGTCCATATAACTGCCACTGACCTCAATCCAGTCCATATTGATTTTTCTAAGTCAAATGTGGATGTGGCAGACCGCAAAGCTACAGGAGTGTCATCTGAAGGTTCAGTTCGTATCATGTCAGAGGTTCCACCACCCTTGTTGGAGGAAACAATGGCTCATTCGTCATCTGGTGGTGATCATGAAAGCTCTCAGGAACCATATAGTCTGTTGGCTGTTTCTGCCGTTATGTCCAAAGCTAATGATGATTTCTCTGAGGTGGTGCATGTGGATGGTGGAGCGGATTCCTCGAGTGACACTGTTATGGTTTCTTCCCCTGCTTTGGTGACATCAGAAATTTCTGACAATGACCCTAATGATGAACACCAGAGAAGTCTATACTTTGAGGATTTCGAGCACGCAAATGTTTCACAGAGAGCATCTGGGATTGATCTCACTGACAGAGAGTTA---------GATGGAATCACAGAGATTGATGAAGGATTCCTGACAGAGTTGGATGGAGTTGGCGACTTTGCTGTTGAAGAAGAGAGCCTTACGAGGCTACACGCCGTTCATGGTGAACCATCCCAATATGTTGAAAGCCACTCCCATAGTTCCCAATATTCAAGAGAGGCCCTCGCACACGCTCATGAACATGATTTTACCCGAGAGCAAAATGAATCCGTGGATGCTTCTACAGTACTTTCACCAAAGTTGGAGTCAGCCATTGAAATCACATTAGATTCCAAAGAATTGATGGTTGAAAGTGCCGAAGAAGAAAATTCCAAAGACAAAGAAACTGGTGAAACTTTAAGTTCAAGCAACATAGATGACAGTAAGACAACCACAGAACTCAAGGCAGTTGATGATGTGAATGTTGAATCTCTGGAGACAAACATAAACCAAACTGAAAACATTTCTAATGAAGTTGAGAGCCTCGCAGAGTTACAAATTGTCAAAGACACCTCTTCCAAAGATTTTGAAGATCCTGAAGCAAGATCAGAGGAAGAGGTCCATTTGAGCTCCCCACAACTCTCAAAATCAAGAGATATCTTAGCAGAAGTAGTCGATTCAGATATCAAATCACCTTCTGAAACAAGTGAATCAAAGGAGGCCATGAACCAAGCCAGTGTTACAGGGATACAAGTTCTTGAAGATAATGTTTCCAAAGATAATACCCACAAAATAGAGGATTTGCATTCCGAAATTTCCACAGGGACAGTTGAGTCATCCCTAGACGTGGTATCACACCCCGAAATAACTGGACCCCAGAAGTGTGTGGTACAACCAAGTCGCGCAGAGGTACAAGTTCTTGAAGACAATGTTCCCAAAGATGTTGAAAACAACTCCCAGAAAACAACAAATTCGCAGGAAGTTGGAACAGAGATCCATGCAACTGAGAGGGAACCGCCAGTTGAAGAAGATCAGCCAAAACCAAGTGAATCAGTGGACGCACCGGCAGAAATTTTGCCCAAGATAGCCAAAGTACATCCCGAAGGATTCCCTGCCGAAGAAATTGAATCAGTCTTTAGTGTTAAAGAAACAGAGGAGGCTTCAAGCTCGAGCGCAACAGTTGTAAAGAAGAAGAAGAAAAGCTCTGGTAAGTCTGGCTCTAGCTCAAGTTCTAGTTCTAGTTCTAGCTCC------------

---------------------------------------------------------------------------------------------------------------------------------------------------------------------------------------------------------------------------------------------------------------------------------------------------------------------------------------------------------------------------------------------------------------------------------------------------------------------------------------------------------------------------------------------------------------------------------------------------------------------------------------------------------------------------------------------------------------------------------------------------------------------------------------------------------------------------------------------------------------------------------------------------------------------------------------------------GAGGCTGAGTCCGTTTCTTCAGTGTCAGATCAAGATAATCACAAGAATATCACCGAAGAAGAATCGTTCCAATCTGTTTCCCATGTTGAACAAGAAGTGTCTACATCAATGTCCCATGTTGAGCATGAACATAATCATATTGAAATTGAAAGCTCATCCTCAGAAGAAGAAGTCATGTCCAAGGTTGAAGGAGATCAAGAATATCACAGAGGTGGCATTGCAGGTGAAGTTGAAGAAGAATTGTCTCAACCAATCTCTCAAGCTGAACAGGAATTACTTCATCCAATGTTGCATCTTGAAGATACTCACGACCATGTAGAATTAGAGAGCTCAACCACTGAAGAAAATGATGTAGTCGAGACTGATCAAGAATTTCAGAGAGATGCTATTGTAGACGAGGGAATTGCAACAGAATCTACTTCCGTCGAGATGCAATCAATACCTTCACCCGTGGGGTCTGAAACTGCTGATGCGACACCTGATGTAGTTGAAGAGAAGCACACAGAATTGAGCTCTTCTGCTTCATCCGAAGTGAACACAAGTGAAGGACCAGAAAGTTCAGAGCAGAAAAGAGATGATAATATCCTTAACAATTCAGAGGAAACGTCTATTGAAGGTTCCAGTCTCCGGAGTGAACTCATAGAAGGAGAAGATATTCAAGCAAAGGAACCTCTATACGATTGGAATTGGAACCCTTCAGAAGTAGAGAAGACGCTTTCTGGCATATCAATTATTGAAGAAGCTTTAAATTATGTGGACAGAAGAGCTCTAACTTCTACATTTTCAACAGTGGCAGACATGGCCGAAGAAGCTAGGGAAGTAGGCTCAACTCCGGTTCATCTGGAAAGAACCATTTCATACATGGATGAGGAACCACTGCAGCATAATGGGAGCATAGAAGAGGGTTCGCAGTCACATGCAGTTGATGTAAACAATTTAAGCTCTATGGAAGTAACCGAGACTAATAACCATGATGTTATTGAAGCTACAATTTCTGAAGATATCCAGGATGAGTCTAACTTAACTGCTCCTATGACGCCGTTAGTAACGGAGCAAGTCGTATGTGGCTCAGGTTCATCTTCCTCAGAAACAGAAATTGAAGATGGGAGTTCCTTCAGCAAGGAAGAAACTGAGCACCAACAGATCCATATAACTACCACTGGCCTAAATCCTGTCCATACTGATGTTTCTCATTTTATTGTGGATGAGGCAGACCCCAAAGCTACAGGAGAATCATCTGAAGGTTCAATGCATATCATGTCAGAGGTTCCACCACCCTTGTTAGAGGAAACAATGACTCGTTCGTCATCTGGCGGTGATCATGGAGGTTCTCAGGGACCATCTAGTCCGGTGGCTGTTTCTATCGATATGTCCAAAGCTAATGATGATTCCTCTGAGTTGGTGCATGGGGATGGTGGAGCGGATTCCTCAAGTCACACGGTTATGGTT---TATCCCTCTACTTTGGCAACAGAAGTTTCTGAGAATGACCCTAATGATGAAATCCATAGAAGTTTACATTTTGATGATTTGGAGCATGCAAATGTTTCACAGAGAGAAACTGGGGTTGATCTTACTGACAAAGAATTAGATGAGGTCGACGGAATCACCGAGATTGATGAAGGATTCCTGACAGAGTTGGATGGAGTTGGTGACTTCGCTGTTGAAGAAGCGAGCCTTCCGGGGCTACACGTCGTTGATGGAGAACCATCTCAATATGTTGGAAGCCACTCCCATAGTGCCCACGATTCAAGAGAGTCCTTCACAGACACCCATGAGCATGATATTACTCCAGAGCAAGATGAATCCGTGGACGAGTCTGAAGTACTTTCATCAAAGTTGGAGTTAGCCATAGAAGTCACATTAGATTCTGACGAATTGATGGTTGAAAGTGCCGAAGAAGAAAAGTCCAAAGATGAAGAAACAGGTGAAACTTCAAGCTCAAGCAACATAGATGAGAGTAAGACAGCTACAGAACACGAAGCAGCTGGTGATGCGAGTGTTGAGTCACTAGAGACAAGCATAAACCAAACTGAAAACATTACTAAAGATGTTATGAACCTTACAGGGTTACAAATTGTTGAAGACGCCTCATCCAATGATGTTCAAGATCCTGAAGCTAGATCAGAGGAAGAGCTTCATTTGAGCTCCCCACAACTTTCAAATTCAAGAGAAATGTTAGCAGAAGTAGTCCATTCAGATATTAACTTGTCTTCTGAAACAAATGTATCCGAGGAAGCCACGAATGAAGCAAGTGTTACAGGCATACAAGTTCTTGAAGACAACGTTCCGAAAGATAATCTCCACAAAACAGAGGATTTGAATTCAGCTGTA---------------------------GATGTAGCATCACATGCTGAGATAACTGAATCTGAGAAAGATGCTGTAGAACCAAGTCTCACAAAGGTACATGTTCTTGAAGACAATGCTCCCAAAGATGTTGAAAACAACTCCCAGAAAACAGAGGAATTGCAGGAAGTTAGAACAGAGGTCCACGTAACTGAAAGGCAACTGCCAGTTGCAGAAGAGCAGTCCACACCAAGTGAACCTGTCAATACACCACCTGAAATTTTGTCCAAGGTAGCAAAAGTACATCCTGAAGGGATCCCTGTCGAAGAAAGTGAAACAGTCTCTACTGCTGGGGAAACAGGGGAGGCTTCAAGCTCAAGCACAACAGTTGTAGATAAGAAGAAACGCTCCGAT---AAGTCTGGCACTAGCTCAAGTTCGAGTTCTAGCTCCAGTTCAAGTGATTCTGAT

3892 Unigene16317_R-chinensis Unigene476_R-bungei

---------------------------------------------------------------------------------------------------------------------------------------------------------------------------------------------------------------------------------------------------------------------------------------------------------------------------------------------------------------------------------------------------------------------------------------------------------------------------------------------------------------------------------------------------------------------------------------------------------------------------------------------------------------------------------------------------------------------------------------------------------------------------------------------------------------------------------------------------------------------------------------------------------------------------------------------------------------------------------------------------------------------------------------------------------------------------------------------------------------------------------------------------------------------------------------------------------------------------------------------------------------------GAAGGCAAGAAATACGGGCATAATGTTCTTTATACAGCTTACTTGTTTGCTAAGCTAAGTTCTATCCTAAAAATCACTCCTGATACAAACAACTACACAAATGTTTTCAGCCCAATTTCTGACAATCTTGAGGAGGAAATTGACAGAGATGATCTGTGGATGGCAAATCCAACTAAAGTAAGCACAAAAGAATTGGTTTTCATATGGGGATTTAGAAAGGAAATGTCTGGTAACGAATTAAAGAGCCATTTACATGAAAACAATGGTCTGATCTCAAACGAGTTTGTGGTTCGAACAGTAGATAAGAATTGTGCAATTATAAGATTTAACAAGCCTGGTTGTGCTGAGGAGTTTATTAAGGCGATAGACGCTGGTGGATTGAAAAAAATGGCCTCAGAAGGGGTGAAAGTAGCAGGGTATGAGGCGTATGAGAAGGTATGCGAGCTGAGTTTATTTGAGATGGATTTAGCGGATTCATTGGACAAGGCGTTGGCAATACGTGTAGATGACGTTTCCAAATCCAATTTTGAAAAGGAAAAGCAAGTGTATTGGAGTAGTGATTCAATTGTGCTAGATGAAATA

TTGCAGAGACGCCTCATCTCCACCTCCACCACCACCACTACTAAACCCTGGTCTTCCACAGTAATCCAAGTCTCGAAATCAAATTTCACAGAATCCCTAATTTCTCTATCAACACAAATTCAATCCTCAGATTACATCGCAATCTCTTCATCAAAAACAGGCGGAGGAACTCTCTCTTCACCATCTCCATGGACTCGTTGTGTTCATCCTTATCTCGATACTCCTCAAACTTCTTATCTCAAATCCAAATACGCCGCTCAGAAATTCCAAATCCTTCAATTCATGATTTGCCCCTTTTCAATTACTAATTCCATCGTCGAAGCTCATCCGTATAATTTTCATTTGTTTCCTAAAGATGAAATGAGTGTTGGAATGCCGGAGTATAGTTTTTCGTGTCAGACGTCCAGTTTGACAGCTATGGCGAGACAAGGGTTTGATTTTAATACATGGATATATGATGGCATATCATACCTATCTAGAGCACAGGAATCGACGGCCAAGCTTCAACTTGGTAACCATATTGGTCATATAACTCCAACCAATCTCTCTTCGGCCCGTTCAGTTGCTGATTCAATTTTCATGGAAAGGATTAAAACACGAGTTACTCACTGGAGAAATGCATGCAAAGATAAAAGAAAGGGCACAGAGGAAGCGCTAGTCAAATCTTTAAGGAAACTTATTTTAGGCGGTGAACTTTATGGTTCAAGGCCATGCATGAGTGTGGATGTTTGCAGTGAACATCAAGTGCAGCTTGTACTAGAGATTTTAAGTGATTGTTCTGATGATCTTGTACCTGTAATCGTTCCTGAGCAAGGTGGGGGAGCCAAGGCTGTGCGCGTCGTGCTGACAAGTTCAGAAGAGGACAAGTGTCTTCTCAAAGCAGAACTTCATAAAATCGAGGAGGAACAAAATAGGAAACTTAGTGGATTTCGGGAAGTGATAGATATGATATCAGCTTCTCAGAAACCAATTGTTGCTTACAATTGCCTCGAAGAATTTTCTTTCATTCATTCAAAATTCCTGTCTTCCCTGCCATCAACTGTGAATGAGTTCATGTCCTCTTTACGCATAGTGTTTCCCCAGATCCTTGATGTTAATTATTTATTGAAGGAGATTGGACCTCTTAAAAAGGCGAATAATATTCATTCTGCTATTTCCCACATTAAGAGACAGTTCTTTATACCCCTCGATATGAAGACTCCCTCCAAAGCTCAGGGAGAAGAAAGCAAGAATTACGGGCATAATGTTCTTTATACAGCCTACGTGTTTGCTAAGATAAGTTCTATCCTAAAAATCACTCCTGATTCAAACGACTACATAAACATTTTTAGCCCAATTTCTGGCAATCTCGAGGAAGCAATTGACAAAGATAATCTGTTGGCTGCAAATCAAACTAAAGTAAGCACAAAAGAATTGGTTTTCATATGGGGATTTAGAAAGGGAATGTCTGCTAACGAATTAAAGAGCCTTTTACATGAAAGCCATGATCTGTTCTCAAAGGAATTCGGGGTTCGATTAGTGGATAAGACTTGTGCAATTATAAGATTTAAGCAGCCGGGGTGTGCTGAGGAGTTTATTAAGGCGATGGACTCTGGTGGACTGAAAAAACTGGTCTCAAACGGAGTGAAAATAGCGGGGTATGAGGCATATGAGAAGGTATGCGAGTTGAGTTTCTTTGAGATGGATTTAGCGGATTCATTGGACAAGGCATTGACGATACGAGTGGATACTAATTCCAAATCCAACTTGGAAAAGGAAAAGCAAGTGTATTGGAGTAGTGATTCAATTGTGTTAGATGAAATA

8824 Unigene38558_R-chinensis Unigene21851_R-bungei

GAAAAGTATGAGAAAGAAGCTAATGAAAATGGGCGAGATAGACAAGAAGGTAAGAGCATATTAAGAAAATATGAATCTGAACCAAACTCAAGAGATGATCGGGGTCAAGCAAGAAGAGGTGATTATGATAGAAAGGAGAGGCCTGGAAGGCACGAGTCAGAACCAAATCAAAGAGAAGACCATGATCGAAAAGCAAGGGAGAACAGGTCAGTATTGCGCGATTCAGATAGAAAGCCAAGAGAGGACCATGATAGAAGGGAGAAGTTAGGAAGGCATGAGTCTGAATCATATAGAAGAGAAGATGGTGGTAGGAAAGCAAAAGAGGATTATGATAGAAAGGATAAATCAAGAAGACACGAGTCAGAATCACATAGAAGAGAAGACGGTGACATGAAAGAAAAAGAGGATCTCGATAGGAAGGAGAAGTCAGGAAGACATGTGTCCGAATCAAATCCGAGAGAAGATGGTGATAAGTCAAGAAGGCATGAGGCAGAGCGAAATAGAAGAGATGATTATGATCGGAGAGATAGTGTTAAACGATCACGGTATGATGAAGATTCATCTTCCCGTCATCACAGGGAGGAAGATGAAGTGCAGGCAAGCCGA---------

GAAAAGTATGAGAAAGAAGCTAATCACAGAAGGCGAGATAGACAAGAAGGTGAGAGTGTATCAAGAAAATATGAATCAGAACCAAATCCACTAGATGACTGGGATCGAGCAAGAAGTGGGAATCATGATAGAAAGGAGAGACCAGGAAGGCACGAGTTAGAATCAAATAAAAAAGAAGACCATGATAGGAAAGCAAGGTTGAACAGGTCAGGATTGCGTGAATCAGATAAAAAGCCAAGAGAGGACCCTGATAGAAAGGAGAAGTCAGGAAGGCTTGAGTTAGAATCTTATAGAGGAGATGATGGTGATTGGAAAGCAAAAGAGGATCATGACAGACAGGAGAAATCTAGAAGACATGAGTCCGAATCATATAGAAGAGAAGATGGTGATAGGAAAGAAAAAGAGGATCTCAATAGGAAGGAGAAGTCAGGAAGACGTGTGTCAGAATCAAATCCGAGCGAAGATGCTGATAGGTTAAGACGGCATGAGACAGAACGAAATAGAAGAGATGATTATGATCGGAGAGATAATGTTAAACGATCACGGTGTAATGAAGATTCATCTTCCCGGCATCACAGGGAGGAAGATGAGGTGCAGGCAAGGCGATCACGGAGG

6003 Unigene13912_R-bungei Unigene30373_R-chinensis

---------------------------------------------------------------------XXGTCACCCAATGTAAGTTTGAGAGAAGTATTATGTGTTGGACTTGCTAAAAATGAAGGTAAGCTTAATAACGGCTTAGAAATCAAGACAGCCGATGTGAATCATACACACTCACTTTCAGGAAATCTAGAAAATCACTTGCAAGAAGACTTCCATGCGGAACCTTCAAGTATTACTAAAAAAGATGGAATAGTGCAGGTTCTGGATAATAGTGCAGCGGTCCCATCTTCTCAGGATGTGGCTAATATTTCTGAGCAGTGCAAAATTAGTGGTAAGCTTCAACAAATGAATGAGATGTCACAGGCATCTGCG------------------------------------------------------------------------------------------------------------------------------------------------------------------------------------------------

XTCCAGAAAAAAAGCCATCTTCCTAAATGTCGATTACTGCAGCTCGAGGAGGCACTTAGAGACCGTGGTGCGACATCCAATGAAATTTTGAGAGAAGTATTATGTTTTCAACTAGCTAGAAGTAAGGATCTCCTCAATAATGGAACAGAAATCCAGGCACCAGATGTGAATCATACACGCTCACTTTCAGGAGATCAAGAAAATCACTTGCAAGAAGACTTCCATGCCGAACCTTCAAATACAAGTAGAAAAGATGGAACAGTACAGATTTTGGAGAATAGTGCACCTGTCCCATCTTCTCAAGTTGTAGCGAATATTTCTAAGCAGTGCAGAAAAGTTGGTGTGCTTCAACCAGTAGATGAGATATCATCGGCTTCTCCTCCAAAAAGTTTGACCGATACAGTGCGAGTTGACCATTCCAATGATCATTTAGTAACTGGAAAGAGAAAAAGATGCCTGTTTGAGAAGGATACTCTTTTGGATAGTGTGGATACTGATCAAGTGTTTGTACCTGAAACCCTAGAGAGTTCACATAGGAAAAATATATTATTGGTTCAATCTGAAGCAGCAGCA

10934 Unigene915_R-chinensis Unigene29610_R-bungei

---GTTGAAGAAGATGAATATGAAGAAGACTATGAAGAAAGAGAACCAGAAGAGTTAGATGCAGAAGAAGCTTATAATAATCAAATGCAAAGAGAACAACAATTGCGAAACCCCACACACATCATCCCCCCAATCCCAAAACGAAGACGAACTGAACCAAATCATCAAGATGAAGATGAAGATAAATCCATCCCTAAAGATGATGAAAATGGGTTATCCATTACTTTATCTGATCCTGATGTTCTTGATTGTTCCATTTGTATGGAACCCTTGTCTCCTCCTGTGTTTCAGTGTGAGAATGGACATATAGCTTGCTCAACATGCTGCACCAAACTCGCCAACAAATGTCCATCTTGCTCATGGCCTATTGGGTACAACCGTTGCTTAGCAATCGAAAAAGTGATTGAATCCGTCAAAGTCTCTTGCTGCTACAAACCCTACGGATGCTCTGATACATTCAGCTACTCACAGAAACTCACACACGAAGAACAATGCAAGCACACTCCATGTTCGTGTCCAATCTCTGAATGTACCTTTCGAGGCTCATCTAAACAACTCTCTCTCCATTTCAGCACCAAACATTGGTCATCTGCTCAACGTGTTCGATATAACTGCCCCATCACAGTCAGTATAGGTAAGTTTGATTCATATCTTGTTTTTCAAGGGGAAGAAGATGATCATTTGTTTGTTCTGAATAACCAAATCGAGCTCATTGGTAGTGCTCTCAGTGTGTGGTGTATATCTGGAAGTTTAGGTGGAGGGCTTGTGTATGATATTATAGTGAGGAAAGGGGTTAGCTGTCTGAGACTGCAATCTCAGACTAAG

TCTCTTGAAGGAGATGAAGTAGACACAGATCAAGAAGAAGAAGGTTCAAGCAGTACACCAATGCCTCCAAGAAATCAGAAAAAGATGAAATCCAGAGAC---------AATGAAGAACAACAACACAACAGCATCCCAAATCCGAAACGAATTCGACCTTCT---------------GAAGATGAATCCATCACTAAAGATGATGAATTTGGGTTATTTATCACTTTATCTGATCCAGAAGTTCTTGATTGTTCCATTTGTATGGAACCCTTGTCACCTCCTGTATTCCAGTGTGAGAATGGACATATAGCTTGCTCAACATGCTGCACCAAGCTCGCCAACAAATGCCCATCTTGCTCATGGCCTATTGGGTACAACCGTTGCTTAGCCATCGAAAAGGTGATTGAATCCGTCAAAGTGTCCTGCTGCTACAGACCCTATGGCTGCTCAGATACATTTAGCTACTCACAGAAACTCACACACGAAGAACAATGCACGCACACTCCATGCACGTGCCCAATAGCTGACTGTACCTTTTGCGGCTCATCCAAACAACTCTCTCTCCATTTCAGCACCAAACATTCTTCATTTGCTAAACAGGTTCGATATAACTGCCCCGTCACAGTCAGTTTAGGTAAGTTTGATTCATATCTTGTTTTTCAAGCGGAAGAAGATGATCATTTGTTTATCCTGAATAACCAAATCGAGCTTATTGGTAGTGCTGTCAGTGTGTCGTGTATATCTGGAAGGTTAGGTGGAGGGCTTTTATACGATATGATAGTGAGGAAAGGGTGT------------------------------

764 CL3214.Contig2_R-chinensis Unigene22726_R-bungei

---GGTGCATCTGAAGCAACAGTTAAAGAGGAAGTGGGTATGACTCATTCAAAAAAGAGAAGCAAGGAG------CGTGCTGGTCAGAAAAACAAGAATTTGCTGGACAGCATCTGTATTTCAGCGAACACAATTACTAAATTTCTCAAACCGGGTAGCTCATTTCCAGAAAACTCAGAACATTCTTTGGCTAGCAGTATCTCAAGTATGGAAGCGCTTTCAGATGATAAACTGACTGTACTGCAGTCACTACACCCATCCAACTGTGAAAATGATACTCAAACCCATATACCCGATGTATGTAATGAGACCGAGTCACCCAGAGGCCTCAAAACATACAGGAGGAAGGATAAAAATATATTCCCTTGGGCTAAGAACTCAGAATCCAGCATACTAAAGGATCAGCTTGAGGTTTCTACCAACTCTCTGATTACTTCAGTAAAGGAAGGTGAAATTAACTCAAAGAACAAAAATGAGTTAAATATAAGTCCAAACGTTCAACACCCACCTCATAACAAGATCCCTGAGGAAGATAAATCGGGCCAGGCATTGAACATCTCCCCCGGTGCTAATATAGTACTGGAACAT------------AATCAAGAAAATGATTCTGCTACTTGTTCCAATCAGTTACGACAGGAAAATGTGGCTTATTCAAGGAAGTCTTTCGAAGCTTGTCCTAACAAGATATTTGCATCAAAGAAGACACATAGAAGGGACAAGATGAGTTCTACTTCTGATCCTTCTCCTGAGTGTCCGAATTCTTCTTCGCATGAAGAAACTACCACATCCTCTTTAAATGAATGTCAAATGGAGGCAATAGCACCTTCAGTTGAGAGTGTTAGTAAAGATGCTGTGATGGTAGGGAATGAGATTATAAAGGATGTGATTCAAAAGAGATCTGCTTCTCCTGCAATAATACATACGACTCAGTTGCGCAGGAAGCTTCTTATTCTTGATCTTAATGGCTTGGTTGTTGATATTGTTTCTTCTCTTCCTGAGGGATGTAAAGCTGACACGTGGATAGCAAGAAAAGCACTTATAAAAAGGCCATTTTGTGATGACTTCTTGAAGTTCTGCTTTGAGACATTCGATGTTGGTGTTTGGTCATCAAGGACTAGAAGAAATGTAGACTCTGTTCTTGATTTTATCATGGGTGATATGAGACATAAACTGATATTTTGTTGGGACCAATACCACTGCACTGACACGGGCTTCAGCACAGTTGAGGAACAACACAAGCCTATGTTACTAAAGGAGCTAAGGAAAGTCTGGCGTAAGTTTGGGGAGTACAATGAATCCAATACTGTTTTGTTAGATGATTCTCCTTACAAAGCACTGATAAACCCGCCACACACTGCCATCTTTCCTTGTTCATATGAGTTTACGCAAAAAGATGATAATGCATTGGGACCTGGAGGCAATATCCGAAAATATTTGGAAAATCTAGCGGCGGCCCCTCATGTTCAGAAATTTATAGAAGAAAACCCATTTGTGCACCCTTTTGGGCAGCGTGCCGTGACAAAGAGGAATACATCTTGGGGTTACTACCTTAGGGTTATC

ATTGGTGCATCCGAAACAACAGATAAAGAGGAAGTGGGTGTGACTCATTCAAAAAAGAGAAGCAAGGAAAAGAGGCGTGATGGTCCGAAATATGATAATTTCTTGGACAACGGCTGTGTTTCAGACATAACAACTGCCAAACAACTCGAACCAGATAGCTCATTTCCAGAAAATACAGAACATTCCTTGATTAGCAATATGTCAAGTAAGGAAGCCCTTTCAGATGATAAGCATGCTGTACGGCAGTCACTGCACCCACCCAACTGTGAAAATGATAATTTAACTCGTACACCCAATGTATGTAATGAGACTAAGTCACCCGTAGGCCTCAAAACATACCGGAGGAAGGATAAAAACTCCTGCCCCTTGGCTAATAACTCGGAATCCAGTATATCTAAGGACCAGCTTGAGGATTCTGCCAACGGTCCGATTACTTTGGAAAAGAAAGATAAAATTAACTCAAGGAACAAGAATGAGTTAAATATAAGTCCAAATGTTCAACATTCACCGTATAACAAGTATCCTGAGGAAGATAGATTGGGTCAGACATTAAACATCTCCCCAGATGCTAATATTGAACGGGAACACGAACCAGTTCCAGATCAAGAGAATGATTCTGCTACTTGTTCCAATCAGTTACCACAGGAGAAGGTGAGCTCTTCAAGCAAGTCTTGTGAAGATTGTCCTAACAAGAGATCTGCATCAAAGAAGATACCTAGAAAAGACGAGAGGAGATCTATGTCTGATCCTTCTTCCAAGTGTCCATTTTCTCCTTCCCGTGAAGGAACCACCACATCCCCTTTAAATGAATTCCAAATGGAGGCAGTAGATCCTTCAGTTGAAGGTCTTAGCAAAGATGCTGCAGCGGTAGGGAATGAAATCGAAAATGATGTACATCAAAAGAGATCTGCTTCTCCTGAAATAATACATACGACTCAGTTGCGCAGGAAGCTTCTTATTCTTGATCTTAATGGTTTGCTTGTTGATATAGTTTCTTCTCTTCCTGAGGGCTATCAAGCTGACACGTGGATAGCAAGAAAAGCACTTATAAAAAGGCCATTCTGTGATGACTTCTTGAAGTTCTGCTTTGAGACATTCGATGTTGGGGTTTGGTCATCAAGGACTAGGAGAAATGTAGACACTGTTATAGATTTTATCATGGGTGATCTAAGACATAAACTGCTATTTTGTTGGGACCAATACCACTGCACTGACACGGGCTTTAGCACAGTTGAGGAATATCACAAGCCTATGGTCCTAAAGGAGTTAAGGAAAGTCTGGCAAAAG------------------------------------------------------------------------------------------------------------------------------------------------------------------------------------------------------------------------------------------------------------------------------------------

2491 CL8468.Contig2_R-chinensis CL3477.Contig2_R-bungei

TCCTCTACACCTAAGTCAGGTATTGGAAAATCAACGCCCCAACTAGTTATTGAAATCAATGATAGTGACAGCGAAAAAGAAAATGAAGCAATTCCAAACGAAACATTTCCAAACATTGTTACTACAAAGACATATCCAAGCCCCGAAAACAAGGAAAATAGTAGCAGTTCTTGTGAGAAAATTTCACCAGCTTCTAAACATAAACGAAAACGATTCAATAATATAGTCAACAGCGATAGTGATGATGGTTCAGACAATGAGATTAGGGCACGAAGATCTCCAAACAATGTTGTGATGAAGCCATGTTTAAGTCAGAAAGGCATTGAAATTAAC---------------------------------------------AGCAAGGACGATGATAACCGTGTAAATAAGGATGTACTGAAGAATGAGATTGCAAAGGTCTTATCTGGAAGTAGGAAAAGAGAAGCAGAAGCTTGTTGGAAGTATGAATTTGACATGTACAAATCCTTGGAATCAGATCCCATACTCTGCATGAAAGCTGTTTGTACACTTTATAGACGTCGCTTGTTT------------------TCATCTTCGAAACTGAATGAACGC------------------------AGGGGAATTAACATAGCCAAGTTTCTTCTGGATGGGAATGATGAGTGTGAGATGAAGAAGTCCGTTGAGGAGTTGGAGATGTATGATCCAAAAGGATTACAAGAATGTTCAAGATTGGCACAGCACTATTCCGATCAGCTGTTCACAATTTTTAAGAACAAGGAAGATCCATTCTTC

------------------------------------------------------------------------------------------------------------------------------------------------------------------------------------------------------------CGGAAACGATTACTCAGAATAGTTGACACTGATAGTGATGATGGTTCAGACAACGAGACTAGGGTAGAAAAATTCCCAAAGAAGGTCGTGACGAAGACATGTTCAAGTCAGAAAGGCGATGAAATTAACAGCAGTTGCGAAAGCAGGCGGGCACTCAAAGTCACAAACAGTGACAGCAAGGAAGATGATAATCTTGTAACTAAGGATCCACCTAAGGATGAGATTTCCAAGATCCTATCGGGAATCAGGAAATGG------GAAGCTTGTTGGAAGTATGAAGTTGACATGGTTAAATCATTTGAAATAGATCCCCTACTCTGTATGAAAGCTGTTTGTACACTTTATAGACGTCGCTTGTTTGAAAAGAAAGCCGATGGTGGCTCTTCAAAGCTGAACAACCGCAGGTTTAGCCTATCCTGTGAAGAGAGGGGAACTAGCATAGCCAAGTTTCTTCTCGATGGAAATGAGGAGTGTGAGATGAAGAGGTCTGTTAAGGAGTTGGAGGTATACGATCCCAAAGGATTACAAGAATGTCAAAAATTGGCATATCACTATTCCAATCAGTTATTCACAATCTATCAGAACAAGGAAGACCCATTCTTC

3060 Unigene22382_R-bungei Unigene10613_R-chinensis

------------------------------------------CTTCATAAATGTGCAGAGAAGGATCTCAAAGAACACCTTCAAAAAGAATCACCATCCCCTGATTTCCTTGCGTTCCGTGTAAAACTCACTGAGTTGACTAATGCAACTCGGAATTTCTTTGAGAACCTAGTTCGGGCATTACAGAATGGCTTGGCTGACGTGGCATCTGGTAGTGTAAGAAATCCAACTAGCTCAAGGTGG---GAAGCAACTAAGAAGGGAAAAGGTGGGAAGGGTTCGAGAGCTCGATTAATTACGTCAAGAGCAGCAACATCAACCCGGGTCCAGGAACCCAGAGATGTTAGCAGCCAGGAAGTCGCCAGTAGTCGGGAAGTCGCCACTACTCAGGAAGTCGCC------------------------------------AGTACCCAGGAAGTTGATGTTCCATGGACGTGTGAACACTGCACCTATTACAATGCAAATGCTGGTCCCATTTGCCAGATGTGCGGCCGACCTCAGATCTCATGGACTTGTGGGCACTGTACTTTTTTGAATGCGGAGTCGTCAACGAACTGCCAGATGTGCAATGAA

TTTGAGTATCTTCAAGGTGAGGCAGAATCTGGATTAGAACGTCTTCATAAATGTGCAGAGAAAGATCTCAAAGAACACCTTGAAGCAGAATCACCATCCCCTGATTTCCATGCGTACCGTGTAAAACTCACTGAGTTGACTAATACAACTCGGAATTTCTTTGAAAACCTAGTTCGTGCACTACAGAATGGTTTGGCTGATGTGGCGTCTGGTAGTGTCAGCTACCCAAGTAGCTCAAAGTACCAAGAAGCAATTAAGAAGGGAAAAGGTGGGAAGGTTTCGAGAGGTCGCCCCTCTGCTTCGAGATTACTAGCAGCATCCCGGGTTCAGCTACCCAGTGTTGTTAGCAACCAGGAAGTCGCCAGTTCTCAGGAAGCCGCCACTAGTCAGGAGGTCGCCACTAATCAGGAAGTTGCCACTAGCCAGGAAGTTGACACTAGTCAGGAAGTTGATCTTCCATGGACTTGTGAACACTGCACCTATTACAATGTGAATGCTGGTCCTGCTTGCCAGATGTGCAGCCGTCCTCAGATCTCATGGACTTGTGGGCATTGTACCTTTTTGAATGCAGAGTCATCAACAAACTGCCAGATGTGCGATGAA

7329 Unigene27303_R-bungei Unigene36565_R-chinensis

AACACTAACCTTCACATAGAGTCACTGTCAGATGGGGAGTCAGAAGTTAGCAGCCAAGTCGCCTCCAATATATCCATTCAAGAAGGCTCGTCAGGT---------------------------------CTTTCGAATCTGTTGTACTCTGCTCCTGGCTCAGTATCCCTTGACCTAACTCTTAGCTGCAAGGCGAGTGATGGAGAGTCCGAAGGTAGGAAAGAATCAATGGGACTCGCATTGTCCAGCAGTAGTGAGAGTAGCAGCGATGCTGTGCCTACTTCGGGACCATCTGTCCAAAGAGTATTCTCTTGCAACTATTGTCACCGGAAGTTCTTCAGTTCACAAGCTTTAGGTGGTCATCAAAACGCGCATAAAAGGGAGAGGACACTTGCGAAGCGAGCTATGAGAATGGGTATCTTCTCAGATAGGTATTCAAGCTTAGCTTCTCTTCCTCTTCACGGGTCTTCATATCGTTCCCTGGGGATAGATGCTCACTCCTCGCTCCACCACATGATTACACCGTCAGAGAGTCCCTTTGTATTCAGAGATAATGCAAGGTTTGATCATGGATATCTCGAGCCGCCTACGTTCATAGAAGATAGTCGTAGGGCAGAT------TGGCCTGGGAGTTTTCGACAGATGATGGCCGAGGAGATTCGTGGTCATCCT---TTTGGATTTATGAGAACAGCAGATTTCAATTATCTCGCAGAGGCTCCACCACCTGTGACAAATTTGAATTTGCCCACTCCAGATCTAACTCTGAAACTA

------GATCTTTACATAGAGTCATTGTCAGATGGTGAGTCAGATGTTACCAGCCAATTCGCCTCCAATATATCCATCCAAGAAACCTCATCAAGTCCTGCAAAAAGCTTCACTGGCAATTCTTACTGCCTTGCAGATCTGTTGAACCCTGCCCCCAAACCAGTCTCCCTCGACCTAACTCTCAGCTGCAAGGCGGGTGTTGGAGAGTCAGAATGTGGGAAGGAATCAATGGGAATCTCATTGTCGAGCACTAGTGAGAGTAGCAGCGATGCTGTGCCTAATTCGGGACCATCTCTCCAAAGAGTATTTTCTTGCAACTATTGTCACCGCAAGTTCTTCAGCTCACAAGCTTTAGGTGGTCATCAAAATGCGCATAAAAGGGAGAGAACACTTGCCAAGCGAGCTTTGAGAATGGGTCTTTTCTCAGATAGGTTTTCAAGCGTAGCTTCTCTTCCCCTTCATGGGTCTTCATATCGCTCCATGGGAATAGATGCTCACTCCTTGCCCCACTACATGGTTACACCCTCAAACTCTCCCTTTGAATTCAAAGAGAATGCAAGATTTGATCATGGAGAACCCGAGCAACCTACATTAATAGGTGACAATGTTCGCGCAGAACTATGTTGGCCTGGGAGTTTTAAACGAATGTTGGCTGAGAGT---CATGGTTATCCTGTTTTTGGGTTAATGAGACCTACAGATTTCAATTATCTCGCACAGGCTCCACCAGCTGAGATGAATTTTAGTTCATCCACTCCAGATCTAACTCTGAAACTC

5083 Unigene32035_R-bungei Unigene24486_R-chinensis

GCTATTGTTGAACAAAATAATGGATCTAAGGCTTCTGGGCCTGTGGTTGAGCAGGAGCAACATGAGAAAGAGAAGCATTTTTGTGAGGCTGACTATCCTCCAGGTTTTGGACCTGAATCGAAAAGCTCGAATATAGCTCAATTATCCATCGCTGATGTGGAGTCATGTCATCTAGGAGGAGTCATAATGAAACAGAGCACCTGTGCAATCAGTCAAGCAAATAAGAACATTACAAAAGTCATGGAGAGTGTAGACAGTGCACTGCATTTATCAGTGCAACCGGCAGTTTACGAATACTTCAAGAATTTTGTTGGCGATGAATTTTCTAAAGCTTATACTGCTGTTGGAGATGAAACAAATAAGGACGACAACCTCACTTCAAGGTCTTCTTGTCAGACTACTGAACATGGCCCTCTTGATGAAGAACCTAGCTTGAGGACAAAATTTTCTGGAGGAAGTATGCCACGCAATGACATCGAAACTGATGCACACCCGCTTTCTTCTGTTCTTCGGTTGGAAGGATCTTGCTCGACGCATACTCTTAGCCCATTTGAAAGATTGGGACTCCCAATAGTCAACACGAACAACTGCATAGCGTCTGATGAACCACCACCTCCCGGAACCGAGGATAGTTTACTACCATCAACCTTACCATCTCAAAATATAAAATTTCAGCCACCAAATTCAGAA------------------------------------------------------------------------------------------------------------------------------------------------------------------------------------------------------------------------------------

---------------------------------------------------------------------------------------------------------------------------------------------------------------------------------------------------------------------CGAGCAAATAAGAACGTTACAAAAGTCATTGAAAGAGTAGAGAGTGCACTGCATTTGTCAGTGCAATCGGCAGCTTACGAATATTTCAAAAATTTTGTTGGCGAGGAATTTTCTAAAGCTGACACTGCTGTTGGAGATGAAAAAACTGAGGACTATAACCACACTTCAAGATCTTCTTGTCAGACTATCGCACATGGCCTTCTTGATGAACAACCTGGCTCGAGGACAAAATTTTCTGGAGGAAGCAGGGCACTCAATGACACTCAAACGGGTGCACACCCACTTTCTTCTGTTTCTCTGTTGGAAGGATCTAGTTCGACGCATACTCTTAGCCCATTCGAAAGGTTGGGTCTCCCAATTGTCAACGTGAACAACCTTATAGAGTCTGATGAACCACCACCTCCTGGAACTGAGGATAGTTTACTACCA---ACCTTGGTATCTCAAAATGTGAAATTTCATCCACCAAATACAGAAGAGCATTATCCTAAGATGAGTGAACATGTGTCTTTGGCAATGTGCCGACAAAAGCTGCACGATGCTGTGCTTGAAGAGTGGGGTGCTTTCTTTTCCAATTTTGCCCTCCGTCGGTGCTATCGAACATGGTGTGATTCAAGAAGACAGGGAGCTAAGTTTCAGCAAGAAGCAGGCAAATCGAATAGAACGGAAGTTGCAAAATCTCCCGCTGTACCGAAATCTTTTAGA

778 CL3259.Contig4_R-chinensis CL1334.Contig1_R-bungei

------------------------------------------------------------------------------------------------------------------------------------------------------------------------------------------------------------------------------------------------------------------------------------------------------------------------------------------------------------------------------------------------------------------------------------------------------------------------------------------------------------------------------------------------------------------------------------------------------------------------------------------------------------------------------------------------------------------------------------------------------------------------------------------------------------------------------------------------------------------------------------------------------ATGTCAATGCAGCGACTAGTTGATGTTATTGGAGTTGTTCAGAATGTATATGCTACTGTGGGGATTAAACGGAAGTCGGAT---GAAACTGAAATACCAAAGCGGGACATCCTGATAGCTGATGACTCCAACAAGACTGTTGTAGTATCTCTATGGAATAAACTAGCAGAAACTAAAGGGAGTGAATTATGGAATGCTTTCAATGACGGTGCACATCCTATTATTGCAATAAAATCTCTTAAAGTTGGAGATTACAATGGTGTTTCTCTGTCAACATTGAGCCGAAGTACTGTGACGATAAACCCAGATGTACCTGAATCAAAGAAGCTCAAATCCTGGTATGAGTCTGAAGGCAAAGGTGCCACAATGGAACATGTTGGAGCTGGCCTAGGCTCAAATTCTCCAATA---------AGATCTATGTACACTGAGCGAGTGGTTATTTCTGATATTAATAGTAACCCATCCTTGGGTGATGGAAAGCCTGCTTTCTTCTCCTTGAAAGCATATACAACCTATATCAAGCCTGATCAAGCAATGTGGTACCGAGCTTGCAAGACTTGTAACAAGAAAGTAGTGGATGGT---AGTGACAGGTGTGAGAACTGCTCCAAGGAATGTGAAAGCAGCTTGAGATATGCGATGTCAATGGTAGTTAAGGACCATTCTGGTCAATTCTGGGTTACTTTGTTCAATGACCAGGCAGAGAAAGTCCTAGGCTATTCTGCTGATGACCTTAATATACTGAAAGAG---------ACAAATGACGGATCTTTCAAGAGGGTTTTAGATAAAGCCTTATTTGTTCCTCATGTCTTTCGCATTGGGGTGCAACAGACTGAATTCAACCATGAGAAGAGGCAAAGGATTCAAGTTAAATCTCGGGCGTCGATTGATTTTGTTGCTGAATCACAGTTTTTATTGAAAGAGATTGAGAAGATGGAGGGATCT

ATGGCGGATTCAATCACCGCAGATGCTGTTTCGACTATCATGGCAAACCCTAATGCTGAAATCCCAGATCTCGTTGTTCAGGTTGTCGATCTCACAAAGCGTGGAGCAAACAGATATATGTTTTTGGCTAATGATGGAAAGACAAAACTCAAGGCAGCACTTTCACCGACGTATTCTCAACAAGTCGAATTAGGAGAGCTTCAGAACTTTGGACTAATTCGCCTTGTTAATTATCTTATCAATGATATTAAACCAGTTGGAAAGTGTTTACTGGTTAACAATATGGAGATTGTCACTCCAGTTCTTGAGAAGGAGATCACAGCTGAACCAACACAAGAGGAACCTAGAATTATTTTGAAGCCAAAAGAAGATGTTGGGATTCTTTTGAAACCAAAAAAAGAAGTGGTGGCTAAATCTGCTGCGGAGATTGTGCATGAACAGAATGGAAACTCTGCCCCTTCAGCGCGGATGGCCACAACAAGAAGAGTTTTTCCCATTGTTGCCTTGAACCCTTACCAGAGTCCCATCAAGGTCCGAGTTACTAATAAAGGCAATTTAAGGACTTACAATAGCGCTAAAGGAGAAGGTCATGTTATCAATGTTGAATTAACTGACGAAGATGGAACCCAGATCCAAGCCACAATGTTTAATGAAGCTGCTAAAAAGTTTGATAAAATATTTGAAATGGGGAAGGTCTACTACATATCTAAAGGAACTCTTAAACTTGCAAACAAGAAGTACAAAACTGTGGAGAACGCATATGAGATGACACTAAATGAAAATTCTACAGTTGAGTTGGTCACTGATGAAGAAAGTATAATCCCTAAAATGATATACAATTTTGTCCCAATTGGTCATTTGGCTTCGTATGTCAATGCAGCGAAACTAGTTGATGTTATTGGGGTTGTTCAGAATGTGTCGTCTACTATGAATATTCGGAGGAAGTCAGACTTTGTAACTGAAATACCAAAGCGGGACATCACAATAGCTGATGACTCAAAAAAGACTGTAGTAGTATCTCTATGGAATGATCTAGCAACCAGCATAGGGGAAGAATTGTTGGATGCTGTCAACAAAGGCACAAGTCCTATAATTGCAATAAAATCCCTAAAAGTTGGAGATTTCAATGGTGTTTCTCTCTCAACCTTGAGCCGAAGTACTGTGATGATAAACCCAGATCTACCTGAATCAAAGAAGCTCAAATCCTGGTATGACTCTGAAGGCCAAGGTGCCACAATGGAGCATGTTGGACTTGGCCTAGCCTCAAATTCTCCTACTTATGGACAAAGATCTATGTACACTGACCGAATGCTTATTTCTGATATTACCAGTAACCCATCCTTGGGTGATGGAAAGCCTGTTTTCTACTGCTTGAAAGCATATATAAACTTCATTAAGCCTGAGCAACCAATGTGGTACCGAGCTTGCAAGAGCTGTTCCAAGAAAGTAACCGACTCAGAAGGCAACTGGTGTGCAAACTGCAATAAAGAAAGTGAAAGCAGCTTAAGATATGCAATGTCAGTGAGAGTTATCGACTTTACTGGTGGAGTTTGGATTACTTTGTTCAACGAGCAAGCAGAGAAAATACTAGGCTGTTCTGCTGATGAGCTTGATGCAATGAAACAGAATGAAGGCACAGATAGTGAATCCTTCAAGGAGGTTTTACAGAAAGCAATGTTTGTTCCTCATGTCTTTCGCGTTGGGGTGCAACAGACCGAATTCAACGAAATGAAAAGGCAAAGGGTACAAGTTAAAGCTCAGGCGCCAATTGATTTTGTTGCCGAGTCGCAGTTTTTATGGAAAGAGCTTGAGAATATG---------

5038 Unigene24119_R-chinensis Unigene13137_R-bungei

AGGATCATGAAAGAGGGCTTGCCTACAAGCAATATATCGAATTCAAAACATCAGAAAGGTATGAAGAAGATGAACAAACAAAAGTATCAGTCGACAAATGGTTCTGGCTTTAAGTTTGAAGCAGCTCAAGAGGTTGAGATTTGTACACCTGGATCACAAGATTCAAGATATGGTACAAGTCAAAGACATAACTCGAGTTCAGAATTCCCGATGCAAAACAGATTTGGCTTCCCATCTGTACTTAACACGACTCAAAACAAGTTCATTGCTGCAAATTTACCATTGGAAGAGACGTGGTATGCTAGTCCAGAAGAGCAGGATACAAGGGAACATACATTTTCATCAGATATTTACTGCCTCGGGGTTCTTCTTTTTGAGTTGTTCTGCTGCTATGAATCATGGGATGCTCATACAGCAGCAATGTTGGACCTCCGGTATCGGATCCTACCTCCAACTTTTCTATCTGAGTATCCTAAGGAGGCTGGTTTCTGTCTCTGGCTACTTCACCCTGAACCATCTTTACGACCTAGAGTGAGGGAAATCCTACATTCTGAAGTAATTTGTGGAACACATGAAGTGGGTCAAAGTCAGCTACAATTACCTGTTGAGGAAGATGAAACGGAATCTCAGATATTGTTTCATTTCCTTTTATCTTTAAAGGAGCTCAAGCATAAACGCGCCTCAAAGTTGGTCGAAGAAATTGGTGGCATAGAGGCAGATATTGAGGAAGTTAAGAAAATGTACTTAAAAAGAACTGATGAGGTTCTCTCTAAGGACCTCTCTAGTATGAGAGAAACTCTTTTAAAAGAACCTGCACCTTCTCAAGCCAGGTTGATGAAGAATATTAAGCAACTTGAAATTGCTTACTTCTCAATGAGATCTCATATCCAGCTCCCAGATGCGGATAGAACAGGCCGCTTGGATAGGGATGTACTAAAAAATCAAGAGGCCTGGTTGGCAGAGAGAAATGAGAATGAGGGTGACCAT------ACTGATTACCTGGGATCCTTTTTTGATGGCTTATGCAAGTATGCTCGGTATAACAAGTTTGAACTACGC------------------------------------------------------------------------------------------------------------------------------------------------------------------------------------------------------------------------------------------------------------------------------------------

------------------------------------------------------------------------------------------------------------------------------------------------------------------------------------------------------------------------------------TCCCCATCGGTACTTAATACAACTCAAAACAAGTTCATTGCTGCAAATTTACCATTGGAAGAGACGTGGTATGCTAGTCCAGAAGAGCAGAATAAAAGGGAACATACATTTTCATCAGATATCTACTGCCTTGGGGTTCTCCTTTTTGAGTTGTTCTGCTCTTATGAATCAAGGGATGTTCAAACAGAAGCAATGTTGGACCTCCGTAATCGAATCCTACCTCCAACTTTTTTATCTGAATATCCTAAGGAGGCTGGTTTCTGTCTCTGGTTTCTTCACCCTGAAGCATCTTCACGGCCAAAAGTGAGGGAAATCCTACATTCTGAACTAATTTGTGGAACACATGAAGGGACTCAAAGTCAGCTACAATTACCTGATGAGGAAGATGAAACAGAATCAGAGATTTTGTGGCATTTCCTTTTATCTTTGAAGGAGCTCAAGAATAAACATGCCTCGAAGTTGGTCGAAGAAATTAATGGCATAGAGGAAGATATGGAGGAAGTTAAGAAAAGGTACTTAATAAGAACTGACAAGGTTCTCTCTAAGGATCTCTGTAGTACGAGAGAAGCTCTTTTAAGAGAACCTGCACCTCCTCAAACCAGGTTGACAGAGAATATTAAGCAACTTGAAATTGCTTACTTCTCAATGAGATCTCAAATCCAGCTCCCTGATGCTGATAGAGCAGGCCGCTTGGATAAAGATTTACTAAAAAATCGAGAGGTCTGGCTGAAAAAGAAAAATGAGAATGAGGATGACGAGAATTCTACTGATTACCGTGGATCCTTTTTTGATGGCTTATGCAAGTATGCTCGGTATACCAAGTTTGAACTACGAGGGACATTAAGAAGTAGTGATCTTGTCAATTCCTCAAATGTAATTTGTTCATTGAGTTTTGACCGTGATGGAGAATACTTCGCTGCTGCCGGGATTTCAAAGAAAATCAAAATCTTTGAGTTTAACAGGCTCTTGAATGATTCAATTGACATTCATTACCCAGTCGTCGAAATGACGAGCAAATCTAGGCTCAGCTGTGTTTGCTGGAACAACTATATAAAGAACTATTTGGCTGCAACTGATTATGTTGGTGTCATCCAGTTATGGGATGCAAGCACTGGT

5432 Unigene36323_R-bungei Unigene26743_R-chinensis

---------------------------------------------------------------------------------------------------------------------------------------------------------------------------------------------------------------------------------------------------------------------------------------------------------------------------------------------------------------------------------------------------------------------------------------------------------------------------------------------------------------------------------------------------------------------------------------------------------------------------------------------------------------------------------------------------------------------------------------------------------------------------------------------------------------------------------------------------------------------------------------------------------------------------------------------------------------------------------------------------------------------------------------------------------CTGGATAGGCTTCGCATGGTCTATGAGGAATATCTAAAACTTGGCTGTGAAACTATTCCTCAAACAGAGAAACGCTTAAATGAACTGATGGAAGATCTGGATCAAAATAAACAGGCTCTCGATGATGTTGTTAGTGTTTTAGCTCAGGTTAAGTCGGAGAAGGATGCAGTGGAAAACTTGGTACAACCGGTAGAAGATGCGGATAGGCTCTGGAAAGAGATCAAAAGTTTGGAAGAAATTGTCATAGACTTGGAGTCTAAGCTTGATGTTGGAGGGCAAAGTGTGAGATCTGTTGAAGAAATTCAGTCAGAGCTGAGTGCTTTGAGGTTTTCAAGCGAGGTGGTGGAAAAAGAGTTAGAGAAGCTACGAGAAGACGAGAAACACATGCGAGATGATTTGCAAGCTATTAGGAATCGCTGGCATTTATCAAGGGAAGAGAAAACAAAAGCAGCTAATATAATTGGCATGATTAAAAAGGCAGAAGAGGATCTT------------------------------------------------------------------------------------------------------------------------------------------------------------------------------------------------------------------------------------------------------------------------------------------------------------------------------------------------------------------------------------------------------------------------------------------------------------------------------------------------------------------------AATATTCTTTCGGAG------------------------

CTGAGGGAATTTGAGAAGGACTTGCAAGATAAAAAGAAAAGCAATGATGAGGAATTAATGGCCCTATGGGCTAGATTTGAGGCTGGAAATGATCGCTGTAGTGAACTAGAGGGTCAGAAGCATGCTAAAGCAGAGATGAAGATAGGTATTATTAAGCGGATCAAAGATAAAGAAATTGAGCGAGATGCGGCAGAATCCGAACTTTCTATACTTTCTTCCAATAGCATAGATCTCGATGAAAAAGAAAATAACCTGAAAATAGAGTTTGAAAGAAGGGCACGTCAGCTAGAAGAAAAGAACTTTAAGGGGTCATTACAGCAGAAAAAAGATGAAATGCGACTTCTGGATGACAAAATACAACATTTGGACCGAGAGAAAGATGTCATTGCCAGTAATTCTGAGGACAGAGTGATACTAGACTTAAAGAGAAGGGAACTGGATGATCGTAAGAAGAAACACAAAAAGATAATGGAGGAGAATAGAGATAAAATTAGAGGTGTCTTGAAAGGGAGGCTTCCATCTGAGAAGGATCTCAAACAGGACATTTCTCAAGCTATAGGGTCACTAAGGAAAGAGTATGATGATTTAAACTCAAAATCAATTGAAGCAGACAGGGAGGTGAAAATTGCTCAAATGAAAGTGCAAGAGGTGAAGAACAATCTGTCTAAGTTCAAAAAAGATATGGAAGGAAGGAAAAGAGTAATGGAGACAAAAGTCCAGTCCTTGTTGACGCGTTCTTTTGATGTCGATTCTTATCCAGAAGTTCTGCAGGAGGCAATGGAGAAACGAGATATGTTGAAAAGCAAATACAACATGGCAGATGGTATGCGTCAAATGTTTGATCCCTTTGAAAAAATTGCACGCGCACATCATTTTTGTCCCTGTTGTGAACGCTCTTTCTCGCCCGACGAGGAGGATGAATTTGTTAGAAAGCAAAGGGTGAAAGCTTCAAGTTCCACAGAACACATGAAGGCGCTGGCAGTAGAGTCATCAAAGTCAGATTCAGTTTTCCAGCAGCTGGATAGGCTTCGCATGGTCTACGAGGAATATCTAAAACTTGGCTGTGAAACTATTCCTCAAACGGAGAAACGCTTAAATGTACTGGAGGAAGATCTGGATCAAAAGAACCAGATCCTTGATGATGTTGTGGGTGTTTTAGCTCATGTTAAATCTGAAAAGGATCTAGTAGAAATCTTGGTTGAACCGGTAGAAAATGCTGATAGGCTTTCTAAAGAGATCAAAATTTTGGAAAATCACGTCAGAGACTTGGAGTCTAAGCTTGATGTTGGAGGGCAAAGCTTGAGATCTATTGAAGACATTCAGTCAGAGCTGAGTGCTTTGAGGTTCTCAAGGGAGGTGCTGCAGAAAGAGGAAGACAAGCTACAATTAGATTGGAGACACATGAGTGATGATTTTCAAGCTGCGAAGACTCGCTGGTACGAGGCAAGGGAGGTTAAAGCAAGAGCAGCTAACATAGTCGGTGTGATTGCAAAGGCAGAAGAAGAACTTGTTAGTCTTGCGGTGGAAAAAGATCAACTAGATCAGGATGAAAAGCAATTGGAAGAAGCACTTGTTCCTTTGTCCAAGGAGAAAGACAACTTGCGACGAGAGCATGAAGTGATGAAAGCAAAGCTTGATAAAGAATATGAAGAGCAGGCTGATGTTAGAAGAAACTACCAAGCCCAAGTAGACGAACTTTCTGCTATTGCTTCCAAAATTAAAGAGTATATGGATGCTAGGAGAGGAGAAAAGCTGGAGGAGATGCAAGAGAAAATATCACTGAATGAAGCACAACTTAAAAGATGCTCAACTAGGAAACATGAAATTTCTACGGAACTAACCAACAGTAACGGCTTGGTGCAGAGTCAGCTTGAAGTGAAACGAAATATCGAGGACAACTTGAAATATAGGCAAACAATTGCTGCATTAGAACAAGCTACAGAAGAGATTGTATCACTGGAAAATAGAATATTGCAGGTTGGGGATCTTGCCACTTTTGAAACTGAGTTTAAAAAACTGCTACATGAGAAAGAGAGACTAAAGTCAGAGCTG

408 CL2222.Contig2_R-chinensis CL1730.Contig8_R-bungei

------------------------------------------------------------------------------------------------------------------------------------------------------------------------------------------------------------------------------------------------------------------------------------------------------------------------------------------------------------------------------------------------------------------------------------------------------------------------------------------------------------------------------------------------------------------------------------------------------------------------------------------------------------------------------------------------------------------------------------------------------------------------------------------------------------------------------------------------------------------------------------------------------------------------------------------------------------------------------------------------------------------------------------------------------------------------------------------------------------------------------------------------------------------------------------------------------------------------------------------------------------------------------------------------------------------------------------------------------------------------------------------------------------------------------------------------------------------------------------------------------------------------------------------------------------------------------------------------------------------------------------------------------------------------------------------------------------------------------------------------------------------------------------------------------------------------------------------------------------------------------------------------------------------------------------------------------------------------------------------------------------------------------------------------------------------------------------------------------------------------------------------------CCAAAGGCTGCGACACATGCTTTCCCTGGATCATCTGTTCGCGAGGTGTTTTCTTCGCGTTCTTGGGCTTCTGTTCAAGTTATAACTGCCGATCAGCGCGCTGAGCTTCTCAAGCGTATAGTACGTGATAATCCAGACAAAAAGATTTCTTTCTCTGACTGTGTCAAAATTGCCAAGGATCTAAATCTTACCATGGAACAGGTGCTCTGCGTCTCTAATGATAAGCGTCAGTCATGTCTAAATACATATCAAAAGGATGTAGACCCTATCACAAGTAATTTTGCATCAGCTTCTCGGAAAAGAAAAAGATCAAAAGAAACACCTTCAAAGCATGCACAACCTGGAAGAATCGGTGTTAGCTCTGTTGATCAAGAGGACCATGATGTTTTCTTTCAAGATCGGGATAAAGATGTACAACAGACAACCACAGATGATCCTGGGCTAGTTGAAGAAGGTAGACAGGAGGATATGAGTTTCATTAGCTGTTGTACACTTCCAAGGTTGAAACCATCACGACGGGGAAGGTTTATGTGGACAGAAACATCTGATAGGGAATTGGTGATCCAGTATGTAAGAAACCGTGCCGTCCTTGGACCCAGGTTTCATCGCACTGAATGGGTCTCACTTCCTGACCTACCCGCCTCTCCAGATACTTGCCGTAGACGTATGCATTTAATATTAAAAAGTCCTATGGTTGGGAAAGCAGTGATGCGCCTTTGTACTTTGCTTGGCGATCGATATGCCCGCCACCTGGTCGAATCTCAACAGAAGGAACTATTGATCTGTAATAGTTCCAGGCATATTCTTCAGAAATGTTCAACAGTAGCAGTTCCCCAGAGAGAAATTCTAGAGGAGAATTTGAAGGAGCAACAATGGGACGATTTTGAGGATGAGAACATAAAACTGGCTCTCAGTGAAGTGCTCCGATGCAAACAGTTGGCCAGACCAGAATTCAGTAGGAGAAGTGGCTCTACCCCTAAAAAC------------------------------------GATTCACATGAAGACCGTTTCCAAGAAGAATTTGGATTGGTTCCAGGAACCAGTAAAGGCGAGGAACCAGTAAAAGTGAGCCAGCATAGATCAAGATGCCACCGCCTTCCCGGAAAGTTCTTAAAACTTCCTAATGAAGGTATCGGTGTCAGCAGACAAGCTTATGAGTCATTGGCTGTTTCCAATGCTGTGGAGCTTCTTAAGCTCGTATTCTTAAGCACCTCATTGGCACCCGAAGTGCCAAATTTGCTGGCAGAGACTTTAAGACGTTACTCCGAACACGATCTTTTCGCGGCCTTCAATTACCTCTGTGAGAAAAAATTCTTGGTTGGTGGTAAGGGAAGTCAACCTTTTGTACTCTCCCAGAAATTTCTACGCAAGGTTTCATCTTCTCCTTGTCCCCCTAACACCGGAAAGAGAGCTGCTAAATTTGATAGTTGGCTTAGAGAGATCGAACAAGATCTGATGGAAGACGAAATCTATCTTAATGAGGGCTTACAATGTGGTGACATCTTTCATTTACTCGCTCTAGTCTCTTCCGGCGAATTATTCATTTCTCCAACCTTACCAGATAAAGGTATCGGAGAGGCTGATGAGGATGATAATAATAATTACACAGTAAAGCGCAAAAGAGATAATAATGCTGATGATAATGTTAAAAAGTTCAAGTCAGTATCGACAGAAGAAGGTTCA------AATCGCCTACCAAAAGGTTTTCCAGGTATCGATGTATCTGTTAGCCGGATCACCATTTCACAAGCTGATGCCATTGGATATTGCCAAGGAACTTTATCTGTAATTGATACTACTCACTCAGGAGCAACTAGTAGTCAGTCTCTTTCTGATATGTTCGGGTCTATGGGAGCAGCGGAAAGAAGGGTTGATGGATCAACTTGGGAAGCTATGACTAGCTATGGAAATCAGTTATTGGGGACCTCTTCTGCTATTTTTTTGGATTCTGATCTATTTAAAACTACTTATTCCTCCATTTACCGAGCTGGTGATCAAGGGCTGAGATTTGAACAAATCTCTGAATTACTGTCTTTAGAGGGAGAGATGGTTGAGTTTATAGTTGATGTGTTTCAAGTGTTTGGAGTAGCAGTCAAGGTGAATGCTTACGATGACATCCGCATTGTAGATGCATCTTTTGTTGACAAGTATTTTTTAACAACAATTGAAGGTCGGTACCAAGGTCTTAAGCCAGCCCCATTAGCTGTAAAATCTTCAACCAAAAACGATGAGAATACTCCACGAATCCCACAAGAGTGTCACACGGATCCAGAAACTATTTTGAGTAGCGATGATGTTCATAGAGTGACCCTTGTGAATCTTCCAAAGGATGTTTCATGCAATTACGTGACTGCCTATGGACACTCTGTGCGAATTGAAAGTGAGGTTGAGACACTTGAAAGAAGAAAGAATACTACA------------CATTCCTTTAGGCCAATTTTGCCATGGATTAATGGAGATGGTACCACAAACCCTATTGTCTATAAAGGACTTGTTCGTCGTGTGCTTGGGATAGTCATGCAATACCCTGGTATATTAGAGGATGGTGTAATATAC------GGCATATTGAATCCGCAG---------------------------------------------------------------------------------------------------------------------------------------------------------------------------

TCTCTACAAAAGCTTGAGATCACCAAGTTTGATGCATTAGAGAGTTTAGAGAGAGGTACTTCAAGGAGTGATAGTGTCAAAGATGATGTAGTTATAAAGGATTATGTACTAGATATGAAAGCTATTTGTGATAAACTCGAGAAAGCTGATGGGAAGGTCCTTGTTGTCTCGGATATCAAGCAGGCTCTCGGATACCGTAAAACTCAAGGCCATAGAGCATGGAGAAATATCCGTAACAGGTTACGGGAGGCCCGCCTTGTTGAAGAGTTCTGTGCGGAGGTGGACAAGAAGGAGGTCCGCTGCATACGTTTAATTAAAAAGCTTGATCCACAGTGTGTGCAACCAAAACATATGGGATTTCCGTCCGACGATGTTGCTACTGAGCAACCTACGAACTTCGGAAAACAAGGACAAAACACCGACCAACTTCTAGAGCTTCCCGTTGAGCATCAGATTTTCGATTTGATTGATGCTGAAGGGTCAAAGGGCATGACTGTTACTGAGGTGTGCAAGAGACTCGGACTTGATAATAAGAGAAATTATAGTAGGCTTCTCAGTATTATCTCAAGATTCGGAATGCATCTACAGGCAGAGAGTTGTAACAGAACTGTGCATTACAGGGTTTGGACATCTGGGAATTTCCCTGGTGAGACTTCTAATAATGTTCATGGTAAATCCGAATATCCGCAATCTCATCAAAAGGTGGATCAAGCCATCCCACTTATAGAATCGTCAACCTCAAAAGATGAATTCAAATCTCCTGACGAAGTTGAATGTGGGCAAAATGAATTCGAGCGTCACTGCAGTTCCCCTCATGACAACGGGAACACCCAGATGCTTATTTGTGGAAGCAGCCTGCATGATTCAGTTAATGGCATTTTAGGTACAGATTGGGACGAGGAACATGAAGTAGGGATTATGGTCTCTGAACCAGTTAGTGCTCCATCAGAAGTACCACCTCCTACACCGTCAACTCCATCAAAGCGCCGATCCTATCCGAGGCACCCATGTCTCATCTCGACTGTAGAAAGTGCTCAAAGGAAGCAGAGGATTATTGAACGACTACAGGCGGAGAAGTTTATTCTTGCCGTTGAACTTCACAAGTGGCTAGTGAGTCTTGAGAAGGACAAACACACAACCATGGACGTGAAGACTATGATGCGCATTTTGGATAAACTTAAACAAGAAGGTGTCTGTAGATGTATTAATATCAGTGTCCCTCTTGTCACCAACTTTGATCGAAAACGCGAAACAGTAGTTGTGCTGCACAATTCTCTTCAGAACCTATCGCCAGAATTGATGGTTAAAATTCAAGAAAAACTGAGAGCATTCGATATGCGAAGTCGTGGGCAAGGATTGGCTCGTCTGAAGAGTGATAAGGAAGTGCCTGTACTGACCAATATCAATAGGAAAGTGTCCCCTGACAGCTCAGATACCCAACCTGTAAGAGCAGACGCCATGCGTGGTAATGTATTTGTCTTGGCAAAGATGGTCCGTGTGAAGCTTTTCCATTGCTTTCTGTGGAGCTACCTCAGTAGTTCACCTGATTGGAGTGATGCTGTATATTCTGGTAGGCATGGGTACGATCTCAAGAATCCTCACAGCTGTTGCAAACTGTTTCCACTGGCTTTGGCTATGAAGACAATGCCACTTGAGCTTTTTCTACAAGTTGTTGGCTCCACGCAAGTGTTTGAAAATTTAGTTGAGAATTGCAAGCTTGGTTTGTGTCTTGCTGATCTTCCTAAGCAAGAGTATGCTGGCCTAATGAATATTCAGGCGACTATGCGCATCTCGTCGCTTGTCGATACTTTATGCCGGTTAAAGTTGATGCGACTGGTTACAGATAAAGACGTAGAAGATACAGAAGAAAGTTCACTTACTATTTTTACATATGCAATGGAGCTTAAACCCTACATTGAGGAACCATTGTCAAAAGTTTCAGTGTCACTGGGCCTTAGTTCTTTTGATCTTCGCCCAAGGATTAGGCATGACTTTATACTTTCAAACAAAGATGCTCTGGACATATACTGGAATATGCTGGAGTATTGCTATGCAACAGCCGACTCAAAGGCTGCAGTACATGCTTTCCCCGGATCATCTGTTCACGAGGTGTTTTCTTACCGTTCTTGGGCTTCTGTTCGAGTTGTGACTGCGGATCAGCGCACTGAGCTTCTCAAGCGTATAGTACGTGATAATCCAGACAAAAAGATTTCTTTCTCTGACTGTCTCAAAATTGCCAATGATCTAAATCTTACCATAGAACAGGTGCTCCGCATCTCTAATGATAAATGTCAGTCACGTCTAAATAGACACCAAAAGGATTTAAACCCTATCACAAATGATTTTGCATCAGCTTCTCGGAAAAGAAAAAGATCAGAAGAAACACCCTCAAAGCATGCTCAACCTGAAAGAATGGATGTTTGCTCTATTGATCAAGAGGACCATGATGTTTCCTTCCAACATCGGGATGAGGACGTACAACAGACAATCGCAGATGATCTTGGGTTCATAAAAGAAGGTAGAGAGGAGGATATTAATTTCATTAGCCGTTGTGCACTTTCAGGGTTGAAGCCATCACGACAGGGAAGGTTTATGTGGACAGAAACATCTGATAGGCAATTGGTGATCCAGTACGTAAGAAACCGTGCCGTCCTTGGAACCAGGTTTCATCGCACTGAATGGGTCTCACGTCCTGACCTACCGACCTCTCCAGATGCTTGCCGTAGACGAATGGACATAATAATAAAAAGGCCTATGGTTAGGAAAGCAATGATGCGCCTTTGTACTTTGCTTGGCGGTCGATATGCCCGCCACCTGGCCGAAACTCAAGAGAAGGAACTATTGATCTGTAATAGTTCCAAGCATATTCTTCAGAAATGTTCAACAGTAGCAGCTCCCCAGAGAGAAATTCTAGAGGAGAATTTGAAGGAGCAACAATGGGATGATTTTGAGGATGAGAACATAAAAGCGGCTCTCAACGAGGTGCTCCGATGCAAACAGTTGGCCGGACTAAAATTCAGTAGGAGAAGTGGCTCTTCCCCTAAAAAAGAATGGGGATCTAATGTTTTGGCTGCTCAACCATATGACTCACATGAAGACCATTTCCAGGAAGAACTTGGATTAATTCCAGGAACCCTTAAAGGGGAGGAACCAGTAAAAGTGAGCCTGCATAGATCAAGAGGCCACCGCCTTCCCGGAAAGTTCTTTAAACTTCTTAATGAAGGTACCAGTGTCAGTAGACGAGCCTGTGAGTCATTGGCTGTTGCCAATGCTGTGGAGCTTCTTAAGCTCGTATTCTTATGCACCTCATTAGCACCCGAAGCACCAAATTTGTTCGCAGAGACTTTAAGACGTTACTCAGAGCACGATCTTTTCACGGCCTTTAAGTACCTCCGCGAGAAAAGATTTTTGGTTGGTGGTAATGAAAGTCAACCTTTCGTGCTTTCCCAACAATTTTTACACAATGTTTCATCTTCTCCTTTTCCTACTAACACCGGAAAGAGAGCTGCTAAATTTGTTAACTGGCTCAAAGAGCGAGAACAAGATCTTATGGAAGGCGAAGTCTATCTTAATGAGGTTTTACAATGTGGTGACATCTTTCATTTACTCGCTCTAGTTTCTTCCGGCGAATTATTCATTACCCCAACCTTACCAGATAAAGGTATTGGAGAGGCGGATGAGGAT---------AATTATACAGTAAAGCGCAAAAGAGATAATAATTCTGATGATAATGTTAAGAGGTTCAAGACAGTATCAACAAGAGAAGGTGAGGTTGTATATCGCCGAGCAAAAGGTTTTCCAGGTATTAATTTATCTGTTAGCCGGATCACCATTACACAAGCTGATGCTGCTGGA------------------TCTTTAATTGATACTGCTCCCATAGGAGCAACTAGTAGTCAGTCTCTTTCTGATCTGTTTGGGTCTATGGCAGCAGTGGAGAAAAGGGTTGATGGATCAACTTGGCAAGCTATGACTAACTATGGAAATCAGTTATTAGGGTCCTCTTGTGCTAATTTTTTGGATTCGGATCTATTTAAAACGGCTTATTCCTCTATTTACAGAGCTGGTGATCAAGGGCTGAGATTTGAACAAATCTCGGAAGTATTGTCTTTAGAGGGACACATGGTTGAGTCTATTGTTGATGTGTTTCAAGTGTTTGGAGTGGCAGTCAAGGTGAATTCTTATGATGACATCCGTGTTGTAGATGCATCTTTTGTTCACAAGTATTTTTTA---ACAATTGAAGGTCGTTACGAAGGTCTTAAACTGGCTCCATTACCCGAAAAACCTTCAACCAAAAGCGATGAGAATACTCCAAGAATCCCACAA------------------GAAACTATTGTCGGTTGCGATGATGTTCATAAAGTGACCCTTGTGAATCTTACAGAGGATGTTTCGGTTTCTCATCCC---------------------------CATGATGAGGTTGAGACAGTCAAAAGAAGAAAGAATACTACTAGTGACTCGTCACATTCCTTTACGCCAATTTTGCCATGGATTAGTATAGATGGTACAACGAACCCTATTGTTTATAAAGGACTTGTTCGTCGTGTCCTTGGGATAGTCATGCAAAACCCTGGTATACTAGAGGAAGATGTGATATGCCGTATGGACGTGTTGAATCCTCAGAGCTGTAAAAAATTGTTGGAGCTCATGATTTTGGATAACCAGCTACTTGTGAGGAAGATGAATCAAAGTATATGCAGTGGACCACCATCCATTCTGGGAAGTATTTGCGGTATGAGCAGATCAGTATGCCGAGATCATCTATTTGGTAATCCCATGAGTTCGATGCACTTA

4767 Unigene13467_R-bungei Unigene21433_R-chinensis

------------------------------------------------------------------------------------------------------------------------------------------------------------------------CCACCTCAATATGATGCAGCTCCTGATGATTCAATCAGGCCTTTTATATCAACATTTTCGATGCCCTTAGACACCAATTCCATCACGTCCCAACACTTGGACTCTGGCTCTGCCACACATAAAGTGTCTGATAGAGTTCTTCCGGCTTCTGCCTCCGATGACATTCCTGTAAGCAGAGGTTATTCAAGTATTGATGGAACTGTATATGATACGCCTAAGGTGGAAGATAGTCTTTCATCCGAAAAACAACCAGACCTTCCT---------------------------------------------------CCTCCTAAGCCAGCTGCTATAGAAACTGTTCGTCAAATCGAAACTTTATGCCAGTATATTGCTAAAAATGGTTCCGGTTTTGAAGTCACAGTCCGGGCAAAGCAACATGGGAACCCAAAGTTTGCTTTTTTGTTTGGCGGTGAGCCTGGCAGCGAAGCTGCAGTTGCACATGAATATTTTCAGTGGACGAAAAGAAATTGTCTTAAGGAGGAAGTTAAAATGCCTAATGGATGCGAGCCTTTAAAAGTTGAATCTTCTACATGTCCAAGTGGTTACTCAAATGAGGATGCATGCCATTCAGCTGCAGAATCTGACATGGATATGGAAGATGACAGTCACCAGTTCAACAAAAAGCAGATTATTGTTGCGTCTGCTAAAGTTTCAGAAGAGCTCGAGGTTCTTAATGTGAAAGATCTGCAACCTGGAAGTCAATGTTCTTCAAATCCAACAGGAAATGTTTCATCTGAATTTCAGCCAAGAATTGACATTCCAATATTGGCTAGAGAAGAAAAAGAATCTGTTCACACTCCTGTTCGGAACTCATCTGGAGCTTCTGAGGGTACATTAGATGACATCACTCACAAACCTGTCAGACCAGTTGACAAATTAACTCTGCCTAAAGCTTCTCCATCTGTTGCGGTACGTAGCAGCTCCAAGGAAGTCTCAGAATCAGTTAGCAATATGGGAAGCCCGTTCCGACTTATACAAGACTATGCTTCTGATGAGAGTGTTGAAGCTGATGTCAGCACTGAAAGTATTTCCCCCCCAATTCCAGTTGAGGAGACATGTTTGTCCAAGCAGTTGCTAAAGGAGGTGACCAGTGTGGAGACTAATTCAGGTTCCTTGAGTGTTCTTCTGCATGAAACAGAA---------TTCACAGAGTCATATCCTACTCGCAAGTCAAGTGTATCGATTAAAGCAGATAAGGTGGTAGATGCGACTCATGTAGCATCCCCTATATTAGATACATTTGCTAAAACTAATGAGCTTCATAATGACAATCACGACTACCAACCATCTAATGATCCGGATCATGGGGATTCAATGCAAGGTGATGATGGTGTTGATTCTCAAAGTGGAAAGCATAACATGAAAAACGAAAGTCAAGGTTCACCTGCACTGAAAGTTGACGAATTTGGGAGAATGGTGCGAAAAAATGCGAGTGACAGTGACTCTGATAGTGATAAGGAGCATTACAGCGGGAGACGTCATAGAAGAGGCCGCAGTCGATCTCCCTTGGAAAGGAGGAGGAGTCGCAGTCCACGAAGAAGAAACGAGAAACGAAACCGATCTCGCAGTTGGTCTCCCAGAAAGCGAAGAAGCACGAGCAAAAGTAGGTCTCCACCTTCTGTTAGGCACAAGGGTGAATTTTCTGGGGAGAAATCGAGACGCGACAGAGATCAGACTCCTTTTTGTTTTGACTTCCAGATAGGAAGATGCTACCGCGGAGCTTCGTGTCGTTATTCACATCATGGACAGGGAGATTCAGTCAGGCGCTATAGAGGTAGACAAGATTGG

CCACCACCACCGCCACCACCACCATCTAGTTTTGCTCCTGTCCCACCAGCATCATCTGTTACTCCACCAGAGGCTCCATCTGAACACGCTCACCCTCCTTCTATGCCTCCTCCTCCACCACCTCCACCTCCCCCCTCTTCCCCTCCTCCACCACCTCCACCCCCGTCTCCACCTCAATATGCTGCGCCTCCTGATGATTCAAATAGGCCTTCTGTAATGACATTTTCGATGCCCTTGGACTCTGGCTCTGCCACATGTAAAGTGTCTGACACTGTAACAAGTGTTAGTGATCCCGTTGATAGAGCTCTTCCAGCTTGTGCCCCCGATGACACTCCTGTAAGCAGAAATTATACGAGTACTGATGGAACAGTATCTGCTATGCCTAAGGTGGAAGATAGTCTTTCATCGGAAAAAAACCCAGACCATCCTCCTAAGCCAAAGGTGGGAGTAAGTCTTTCATCCGACAAAAATCCAGACCATCCTCCTAAGCCAGCTGCTATAGAAACTGTTTGTCAAATCGAAACTTTATGCCAATACATTGCTAAAAATGGTCCCGGGTTTGAAGTCACAGTAAGGGCAAAGCAACATGGAAACCCAAAATTTGCTTTCCTGTTTGGTGGTGAGCCTGGCAGTGAAGCTGCAGTGTCGCATGAATATTTCCAGTGGATGAAAAGAAATTGTCTAAAGGAGGAAATAAAACTTCCTAAAGGATGCGAGCCTTCAAAAATTGAATCTTCTACATGTCCAAGTGGTTACTCAAATGAGGATATATGTCATTCAGCTGCAGAATCCGACATGGATATGGAAGACGACAGTCAC---TTCAACAAAAAACAGATCATTGTTGCATCTCCTAAAGTTTCAAAAGAGCCCGATGATCTTAATTTGAAAGAGCTGCAACCTGAAAGT------------------------------TCATCTGAATTTCAGCCAAGATTTGATTATCCAGTATTGGCCAGAGAAGAAAAAGAT------CACAGTCCTGTTGGGAACTCATCTGGAGCTTCAGAACGCACATTAGAGGTCATCACTCACAAACCTATCAGACCAGTTGATATATCAACTCTGCCTAAAGCTTCTCCATCTGCTGCTGTGCCCAGCTGCTCTAAAGAAGTCCCAGATTCAGTTAGTAATATGGGAAGCCCGTTCCGACTTATACAAGACTATGCTTCTGATGACAGTCTTGAAGCTGGTATCATCCCAGAGAGGATCTCCCCACCAATTCCAGCTGAGCAGACATGTTTGTCCAAGCAGTTCCCAAAGGAGGTGACCAGTGTGGAGATTAATTCAGGTTTCTCGAGTGTTCCTTTGCATGAAACAAAACTATCTTCTTTCAAAGAGTCATGTACCACTCACAAGTCAAGTGTATCGAAAGTAGCAACTAATGTGGTAATTGGGACT------------------------------------------------------------------------------------------------------------------------------------------------------------------------------------------------------------------------------------------------------------------------------------------------------------------------------------------------------------------------------------------------------------------------------------------------------------------------------------------------------------------------------------------------------------------------------------------------

9427 Unigene39957_R-chinensis Unigene38659_R-bungei

TTCTGTCTTCCTCGACATAAGACGACGACGACTATATTGGATGTCAACATTGACGCCTTTCCCCTCCAGCCATGGAATCATCCTGGAGTTGACCCTTCTCTTTTCTTCAATTTTGGCTTCCATCACCACTCCTGGAAACATTACTGCAACACCTTCCAACTATACACCCAACAACAAACTTTTACCACCAGTAATCAGACCTCTCGACTCAACGAAAAGGAAGATCAGCAGCCCAAGGGAAGGCCAATACAAGTTGATGCTGGCATTGGAGAGCGTTGGCCATCCATCGATATAAGGCGTCCACAAATTCGTGACTCTGACGTCGTTATACAGATTTCAGTTCACGACCATATCCAAACTTCTTCCACTTCAACTCACCAGGACTCCCCCCATTCTGCTCAAACCACCCACCAACATACAAGGTGTTCCCCACAAAAAATTGCATCTAGTCCGTTACATGTGGAAAATGAGGATGACAGCGACTGTTGTGATCATCATAATCTCAAAAATCGTGCTTGCATGAAAGACTCCAAGAATATGGAAGCAAGTAAGGATGCAATGGAAAAACTTCAGAAAACTCCCGTTAAAGAAGACCTGGGTATGGTGGAAGCGTTACACGGCGTT------CATGCTTCCAAAATGTCATTTTCTGATAGCGATAATGAAGCATCTACTGATGGGGATGCTCCTGTTAAGGAGAAACGACACAAGCATGCAAGAAAGCAGTTTTCAAATTCTGCTAGTGGGTTACTTGCACCTGTCACTCTAAGATGTGATAGATCTAAGGAGCCTGAACGTAATAAGTACAATACTGAACGAAGAAATAATAAAGATGAGTTAAAAAATCAAAGCCACGTGAAAAGAGAGCGATGTAATAGA------------------------------------------------------------------------------------------

------------------------------------------------------------------------------------------------------------------------------------------------------------------------------------------------------------------------------------------------------------------------------------------------------------------------------------------------------------------------------------------------------------------------------------------------------------------------------------------------XXCAGTTGTGATCTTAATAATGTTAATAAACATGCTTGCATGAAAGACTCCAAGAATATGGAAGCAATTAAGGATGCAAAGGAAAAACTTCCAAAAACTTTCTCTGAAGAAGACCTGGATGTGGTGGAAGCGTTACACGGTGTTCTTAATCATGAATCCAAAATGTCATTTTCTGATAGACATAATGACGCATCTACTGATGAGGATGCACCTGTTAAGGAAAGACGACACAAGCATGCAAGAAATCAATTATCAAGTTCTGCGAGTGGGTTACTTGAACCTGCCACTCTAAGATGTGATAGATCTAAGGAGTCTGAAAGTAATAAAAATAGTACTGAATGGAGAAATGATAAGGATGAGTTAAAAAATCAAAGCCACATGAGAAGGGAGCGGTGTAATAGAGCAAGACTACAGGGTGTTGCCGACTCTAAAAATGTGGATGATGACAAGGCCATTCCAATGCTTAGTAGAAATAATTGGGATGCTGGGAAT

6514 Unigene19490_R-bungei Unigene35142_R-chinensis

AAGTCTCTACGAGGAGCAGAGCTCTTTGGAACAGATCCCCACGGGCGGCAATATTTTGGTGCTTGTGGCTACCTCTTGGTGTTGGATTCATGTGAACCAGATTCTTCTTATCACTTCTACAACAGCAGTGACGTGAATAATGTTATCGAAGTTTTGAAGTCATCCAATATACTTTATAGTAATATAACAAATGCGATTATTAAGAACTGGGGCATTCTTGGAGGGAATGATTATCAGAACTCTGAGATGGATATGATATGCAGAGTCTTGGATCTGAATAATCAAATACCTACTAATATGTCGTCTTCAGTTGCTATGCCGCTCTTAGAAATATCCGAAGGTAAATCTGCAGACAACCCTTACGAAACATCTGTTAGAAATGAAGATTTTGATCTTCAGGGCTATATGGGCTCAGAATCAGAAATCAATCCCGATTCTGGATTGGGGAACGAAACAACAGAAACTAGTATTTTGGCCAGAAAACCCGAGGACTTGGGCGAAATCGAGAAAATTGCTGCAGTTGTTCAAAATTGTGTGGAATCTAATGAAGACAATCTAAATGGA---CCTAAAACTTCAAGCAAGTCTAAAATTCTAGAAGAAACTCATTCCACAGCTGCAGACTGCTCTATGCAATCAAGGGTATCTTCTGTGAGATCAGAAAAGGAAACAGAGGCCGGGCTTTCTTCTTATGAGTCAGCAGGGACAAAGCAGGAAAAACGAGCATCACCTCAAGTTTGTTCTGAACCTGATCCTTATGTCAACTATTATGCTTTTGGTCAGAATGCTGCATCTGTTGTGCGAAAGTTACTAAGCAAACCTCCAAATAATGTAAAAGAGGATGATACGAGATCACTGGAGGAGATAATTTCACCAGAGACGAGGTCCATTTTTCAGAAGTCAATAGGTTTTTCATGGTCAAACATCCAGGGGATCAATCAGAATGCCCGACAAGAGGATTGCGGGTGGTGCTTGTGTTGCCAAAATCCCGGTGAAAGCGAGACTTGCTTATTTTGTCCGACTGGCAAAAAACCTTCTCCTGATGTTTCAGCAAGTGGTCCATTTAGTTTACGTTCTAAGAATAGCAGGCAAACTCATCTTTCTGCTGTCATTTGTCACATCCTCTCCATTGAAGAACGCTTATGTGGACTCCTATCTGGTCCATGGAAAAATGCAAGCTATAGCAAGAACTGGCGCAAAACTGTTCTGAAAGCCTCTGATGTTGCATCAGTGAAATACCACATACTTACACTGGAGTTAAATATGCGCCGTCTTGTACTTCTGCCAGAGTGGCTGAAACATGTGGATTCTGTAGTTACGATGGGTTCGGCTTCTCATATCATAAAAGCACCAGTTCAGGTACCTTCCTCAAAAAATGCGTCTGCAAGAAAACGGGCGAAAAATGCTGCTGTAGAGGCCATTTCTGCTTCAACTCCTGCTTCCAAATTAAGGACTCTCTGGTGGAGGGGTGGAAAACTTTCTCGCTCCATGTTTAACTTTAAAGTCCTGCCTCACTCATTGGCTTCCAAGGCTGCCCGACAAGGTGGGAGAAAAAAGATACCAGGCATCTTATATTCTGATAGTTCAGATTACCCTAAACGAAGTAAAAACATAGCTTGGAGAGCTTCTGTTGAGATGTCAATGAATGTGGCCCATCTTGCTTACCTGGTTAGAGAGCTTGATTCGCATATTAGGTGGGATGACCTTGAAAATACTCAAACTTTCTCTCACTTGTCCAAGGAATCTAAAAAAGCAATGAAACTGTTCAAGAAAGTGACCATCCGCAGGAAGAGTACCGCAGGAACGAATATTAAGTATCTTCTTGATTTTGGAAAGAGAAGGGGTATTCCTGATATCGTTATTAGAAATGGTGTTATGCTTGAAATATCTGCCAGTGAGAGAAAGAAATATTGGCTAGAAGAATCTTATGTTCCTTTGCACATCTTAAAGGCATATGAAGAGAAGAAACATGTTTTAGCTTCTTGTAAGATGAAGCCTGTACTCCTTAGTGAGGCTGCAAAATACTTGAAAAAGTCCACCAGGAGAAATGGACTTCAGCTTCTTATATCCAGAGGGCAAAAGTCCGAATACTACAAGTGTGGTCATTGTAACAAAGATGTTTTGATCAGGGATGCAGTAAATTGCAAGGATTGCGAAGGTTTTTTCCACAAAAGACATGTGCGGAAGCTTGGATATTTCAGTGCAGCTGAACCTACGTATCAATGTCAACAATGCCGGGAGTCGTCTCCTGTAAAGAAATCTGCTAAA

---------------------------------------------------------------------------------------------------------------------------------------------------------------------------------------------------------------------------------------------------------------------------------------------------------------------------------------------------------------------------------------------------------------------------TCAGAATCAGAAATCAATCTGGATTCTGGACTGGCGAACCAAACAATAGAGACAAATAAGTTGGCCAGAAAATCTGAGGACTTTGACGAAATTGAGAAAGTTGCTGCAGCTGTTCAAAAATGTGTGGAACCTAATGAAGATTGTCTAATTGTACTTTCTAAAACTTCAAGCAAGTCTCAGATTCTAGAAGAAACTCATTCTACAGTTGCAGCCTGTTCTGTGCAATCAAGGGTATCTTGTGTGAGATTAGAAAAGGAAACAGAGGCCGCACTTTCTTCTCATCCATCAGCAGGGACAAATCAGGAAAGAAAATCATTACCTCGAGCGTATTCTGAGCCTGATCATTATGTCAACTATTACACTCTTGGTCAGAATGCTGCATCTGTTGTGCGAAAGTTACTAAGCAAACCTCCAAATAACGTAAAAGAGGACGATACAAGACCACTCGAGGAGATAATTTCACCAGAGACGAGATCCATTTTCCAGAAGTCAATAGGTTTTTCATGGTCAAACATCCAGGGGATTAATCAGAGTGCCCGGAAAGAGGATTGCGGGTGGTGCTTGTGTTGCCAAAATCCCAGTGAAGGCGAGACTTGCTTATTTATTCCAACTAACAAAAAACCTTCTTCTGATGTTTCAACAAGTGGTCCGGTTAGTTTACGTTCTAAGAGTACCATGCAAAGTCATCTTTCTGCTGTCATTTGTCATATCCTCTCCATTGAAGAACGCTTATGTGGACTCCTATCTGGTCCATGGAAAAATGCAAGCTATAGCAAGAACTGGCGTAAAAATGTCCAGAAGGCGTCTGATGTTGCATCAGTGAAATACCACATACTTTCACTGGAGTTAAACATGCGCCGTCTTGTACTTCTACCGGAGTGGTTGAAACATGTGGATTCTGTAGTTACGATGGGTTCGGCTTCTCATATCATAAAAGCAGCAGTTCAGGTACCTTCCTCAAAAAATGCATCCGCAAGAAAACGGGCGAAAAATGCTGTTGTAGAGGCCATTTCTACTCCAACTCCTGCTTCTAAATTAAGGGCTCTCTGGTGGAGGGGTGGAAGACTTTCTCGCTCTTTGTTCAACTTTAAGGTCTTGCCACACTCATTGGTTTCCAAGGGTGCTCGACAAGGTGGG------------------------------------------------------------------------------------------------------------------------------------------------------------------------------------------------------------------------------------------------------------------------------------------------------------------------------------------------------------------------------------------------------------------------------------------------------------------------------------------------------------------------------------------------------------------------------------------------------------------------------------------------------------------------------------------------------------TGC---------------------------------

2558 CL1950.Contig7_R-bungei CL8687.Contig1_R-chinensis

AACAACAAATTTTCAGGTAAAGAGCTTTTGTCATTGTATGGTAGAAGCAAAAATGAAATTGGACTGGCTCGCTACACGAGCATGGATTGCTTTGATCGTTTCAGGTCCACATTAGAATGTTCTGCACCATTTTCTGATGAAAATGGAAGCTCTGCGAAACCAATCTCAGGTATTAATTCATCATTGGAGTCTCTGATTGGAGGTTCCCTCAGAAACGAGTTACCAAATTCTGCCATATTTTCCACTAGCACATCTTTTTCTGATAGACCATCGGTGGGCTCACAGGAAACCAATACTTTATCTATCCCAAGGACTGATATTTCGACAGAAGACTTGAGAAAGGGGCCAACTGTTCCCGTATTGTCTGCTGACAGCACGAGGCTAAGGATTGATGAAAGCCTGGGGTTGCATGATAATAGCATAGTACCTTGTCAGGATATTGAACATTCACGTTTGAATCAAGACAAAAATGATGGTGTCGGTGACAATGAAGGAGTTGTTGGTATTAAAAGGAAGAGGTTGTCAGATGAAGTTGAATCATTTAAGTGTTTGCCCACTGAGGTCACAAACTTTCCCACAGAGATAGAAGAAAACCAAATTAATAGGAATTTGAATGACTCCGGAGATGGAAGATGTTCAACTGCTCATTTGCTGGATTATGCACACGCAAGGAATTACATTTCCAACAAGAAGGCCAAGGTGTCTATGACATCGAGACAAATTCCAGAGCCATTTACTCTGGGCTTTGAAGTCACTGGAGATGTCGGGGTATTCAACCAGACGTTATCACATGATAATTATCTCGTGGACACCATCCAGAGTCGTAGGGGTGATACTGTTGAAATGGACCTGTCAACTGAGGTATCCTTTGATAACATGACTGGTGAAGCCATACTGAAATTGCTGGAGTTGGATGATCCGGATGATGAGGAGAGGTTTAAGATGGCAATGGAGATGCCTCTGTCACCTACTCTGCCAGAAATTAGGATGCATACTATGGATGCATTTCAAGCTGATGACATATTTAATGTCATGATAAATGAGAGGGATAGTAGTAAAGTCGTGTCTTGTGGCCTCAGTGTGGAAATGGAGACAGGGAAGCAACCTCTTAGAAATGAAATGATGAAAGTTTCTAAGTATTTTGTTGCATTTCCAAACATG---GACAAAACCAGTATTTCTAGGATAATATCTGCTTGTGAGACCTATATATCTAGTAGCCCTATTGTCTCTCCAGAAATGTGGACGGTGGAGGAATTTTTGGTTGCCATTGCGAAGGAAGAGGATCTGATATCTGAGGAGAAAGCTTGTGTGTTCTTTTCATTGTTGCTGCACAAGTTCTTGGGCAATATGCCAGCAGATTTTACAAGTATTTTTGCAGACGATATCCGTTCCTGCTGGAATTCCTTCGAGACACATATGAAAACAGTGATGTCGAATGCAGAGTCAAGAAACAGACTTCTCAAATTATTCCATTTGGACACCTTGCTTAGTCTTATCGAGAGTTTCATTCTACATAAAAGAATTACGTCATATCAACCATCGATTTCCTCTGAAACAGCTACCACTGATACACTGGTAGCTGCCAGCTTTATTTTGGCATCGATATGTTTGACAACTGGCCGGATTGGTTATATTTGCGAGGCGTCATATGAGCTCCTTCGAATGTGCAGATTTGATACTTACTCGACGCTTACGATACTTCATGTTTTTGCTTTGGTTTGTGGAAGCGCTTATCTTAATTTGGGAAGCCATAGCTTAGTTATATCTGTGATTAGATGTATAGTTACACTTCTGGAGAGAGATATTAGAACTGAGAGTCTAACTACACCTCGGTTTCATCCATGTGTTGAGTGCGCATTCTCCAAAGGTGTAGAGGTTTCTGTTGACACAATTTCGAGTCTGCTTCTTGAAAAGCTCCAAGAATGTGTTGCTGTAGTAAGGACAGGAAATAAGCCTTTGAATGATGTTAGCGATGTTATGTCATTGGTGGAGCTCCTTGCATCCTACATGAGTTGGGAGTGGACATGTAGAAGTATTATTCCCCATCTTCTAAAGATAGCGGATTCATGCGTCCAACAGGAAACTTCAGCTGCTGTTGTACTTCTTATTGGTCAACTTGGAAGATTCGGAATTGATGATAGCAGTGGAAGTGAACAGTTAGGAGTGGAAGAGCTGAGATGTAGTTTGGCCTCCTTTCTTGATCAGAGAAGTACAAGGAATTGGACTCTACCTACACAATTTGCCATTATTCACGTTTTAACAGGTCTATCTCCCAATAAATTCAAAGAAGTTGTAGAGAACAAAGGGCTT---GTAAGCCAACATGCCAATTTTGTACGAGCATGG------------------------------------------------

---------------------------------------------------------------AAGGCTAGCTACATTAGCATGGATTGCTTTGATCGTGTCAGATCCACATTAGAATGTTCTGCACCATTTTCTGATGAAAATGGAAGCTCTACAAAGCCAATCTCAGGTATTAATTCATCGTTGGAGTCTCTGATTGGAGGTTCCCTCAGAAACGAGTTACCAAATTCTGCCATATTTTCCACTAGCACATCTTTTTCTGATAGACCATCGCTGGGCTCACAGGAAACCAATACTTTATCTGTCCCAAGGACTGATAGTACGATAAAAGACTTGAGAAAGGGGTCAACTGTTGCTATATTGTCTACTGGACGTACGAGGGTAAGGATTGATGAAAATCTGGGGTTGCATGAAAATAGTTTAGCACCTTGTCAGATTATCGAACATTTACGTTTAAATCAAGACAGAAATGATGACACGAGTGAAGATGACAGAGTTATTGGTGTTAAAAGGAAGAGGTCCCCAGATGTAGTTGAATCATTTAAGAGTTTACCCACTGAGCTTACACACGTTTCCACAGAGATGGAAGAAAACCAAAATGATAGGAATTCTTTTGAGTCAGGAGATGGAAGATGTTCAACTGCTTGTTTGATGGACTATGCACATACAAGGAGTAACATATCAAACAAGAAGGGCAAGGTGTCCCTGACACCGAGACAATTTTCCGAGCCATTTAATCCAGGGTTTGAAGTCGCTGGAGATGTTAGGGTATTCAACCAGACTGTATCACATGATAAGTATCTTACAGACACTATCAAGAGTCGCAATGGTGAGACTGTTGAAATGGACCTATCAACTGAAACATCCTTAGATAATATGACTGGTGAAGCCATACTGAAGTTACTGGAGTTAGATGATCCGGATGACGAGGAGAGATTTAAGATGGCAATGGAGAGGCCTCTGTCGCCTACTCTGCCGGAACTTGATATGCCTACTATGGATGCATTTAAACTGGATGACGTATCTAATGTCATGATAAATGAAAGCAATAATAGCAGATTTTTGTCTTGTGGCTTAAGTGTGGAAATGGAGACAGGGAAACAACCTCTTAGAAATGAAATGATGGAAGTTTCTAAGTATTTTGTTGCATTTCCGAACATGGAGGACAGAACCAGTATTTCTAAGATAATATCTGCTTGTCAGGCCTGTATATCTAGCAGCTCTATTGTCTCTTCTGAAGTATGGCTGATGGAGGAGATTTTGGTCGCCGTTGGGAAGCAAGAGGATCTGATATCTGAGGAGAAAGCTTGTGTGTTCTTTTCATTGTTGCTGCACAACTTCTTGAGCAATATGCCAGCAAATTTTACAAGTGTTTTTGCAGACGATATCCACTCTTGCTGGAATTCCTTTTTGAGACGCATGAAAACAGTGATGTCTGATGCAGAGTTAAGAAACAAACTTCTCAAATTATTCCATTTGGACATTTTGCTTAATCTAATAGAGAGTTTCATTCTACATAAAAGAATTACGTCATATCAACCAACGATCTCCTCTAAAACAGCTTCAATTGATACATTGGTAGCTGCCAGTTTCATTTTGGCATCTATATGTGTGACGACTGGTCGGATTGGTTATCTTTGTGAGGCATCATATGAGCTCCTTGGAATGTGCAGATCTGATACTTACTCGTCGCTTACAATGCTCCATGTGTTTGCTTTGGTCTGTGGAAGCGCATATCTTAATCTGAGGAGTCACGGCTTACTTATGTCTGTGATTGGATGTATAGTCACACTTGCGGAGAGAGATATTGGTTTG---------------------TTTCATCCATGTGCCGAGTGCGTATTCTCCAAAGATGTAGAGGTTTCTGTTGACAAAATTTTGAGTTTGCTTCTTGGAAAGCTC------------CCTGTGGCAGGGACAGGAAACAAACCTCTGAATGATGTTAGTGATGTTATTTCGTTGGTGGAGCTCCTTGCATCCTACATGGGTTGGGAGTGGACACATACAAGTATGATTCCCCATCTTCTTAAGATAGCGAATTCATGCCTTCAAGAGGAAACTAGAGCTGCTGTTGTACTTCTTATTGGTCAACTTGGAAGATTTGGAATTGCCGAGAGCAGTGGAAGTGAACAGTTAGGAGTGGAAGAGTTGAGACGTAGTTTGGCCGCCTTACTTGATCAGAGAAATATAAAGAATTGGACCCTACCGACACAATTTGCCATTATTGACGCTTTGACAGGTCTATCCCCCCTTAAATTCCAAGAAGTTGTAAGGAACGAAGGGCTTGAGGTGAGTCAACATGCCAATCTTATACGAGCCTGGTTTACAGAGCTTAATCAAGAAGAGAAGGCATTGTCACTTGGCATTCTC

4890 Unigene22642_R-chinensis CL2735.Contig1_R-bungei

CCAATGGCGCGATTTTTCCCAGTTTTGTTCCTC---ATCTTGCTCCTCACTGCTACTGAATTTGGACCTAGGGTTACTGAGGCAAGACAATGTAGTTCCGCAAGCCATAGGTTCAAGGGGGGATGCGCATCAAATCATAACTGTGCTGCTATTTGCAACACCGAAGGCTTTCATGGTGGTAGCTGTGCTGGATTTCGAAGGAGATGTATTTGCACTAAACCTTGC

---------CGATGCTTCCCAGTTCTGTTCCTCATCATCTTGCTTCTTACTGCTACTGAATTTGGACCAAGGGTTACAGAGGCAAGACAATGTGATTCCGCAAGCCACAAGTTCAAAGGGACATGCTTGGCAGATCGTAACTGTGCTGCTGTTTGCCAGGGAGAAGGATTTCATAGTGGTTACTGTGGTGGACTTCGAAGAAGATGTCTTTGCGCTAGACCTTGC

1040 Unigene29574_R-bungei CL4018.Contig1_R-chinensis

GCACCA---------------------------------------------------------------------------------------------AAGATATCTGGT---------------------------------------------------------------------------------------------------------------------------------------------------------------------------------------------------------------------------------------------------------------------------------------------------------------------------------------------------------------------------------------------------------------------------------------------------------------------------------------------------------------------------------------------------------------------------------------------------------------------------------------------------------------------------------------------------------------------------------------------------------------------------------------------------GGTGGCTTAAACACTTCAAGTTTTCATTCTGATCATGTTGCTAAAGATGAACCAAAAAGTAATGTGTCAGTTCGAAAGGTTAATGACTTGGACACTCAGAATATTTCGAAGGCAGGTTTAGAGAGGTCAAGAACACCTATATTGCCACATACCATTAGAACGGTAAGTAGTTTTATCACATCTTTTCCGGCTGCCACACCTCCAACGCTCCCCCACCAGTTTCTAGATGGTTGCTCTGAAATAATTCCAGACCCAGCACATCCAAAAGCAGAAATTCATGCTGGGTCTGCCGAAAATCAAAATGATGTGGGTATGAACTTCGAAACCCAAAGCGAATTAGCTAATGTGAGACCACAATCATCTGAGATGAAGTTGGTTACATATGTGAAGCGAAAATCCAATCAGTTGGTTGCTTCATGTGATACTGAAGTTCATGATTGCTCCAACGCCGCAGAGAATACTCAGTGTTTACCTTCATCCACACCATCTGATCAGTACTACAAAAAGAGTAAGAATCAGCTTATTCGTAATGATCCATCTCTGAGAAGCCACTTGATTCAAGCAGTTTCCGTTCCAGATGGTTGTACAAATTCAGAGAGTCAAAGAGCTTCCACAGTTTCCTCCCTGAAGTGCATTAGAAATATGAATAAGCGAAGGATCAATAAAGCTCTTTTAAAGACACGCAAGCCTTGGTCTTTGGTGTGGACCTTACATGGGGCACAAACACAAAATGGAGATGCAAGTTCACTGCAGCGGCGTAAGGTTTATCCTTCTCTTCTCCCATGGAAAAGGACGACACAATGGATATCCAGTAAGAGGCCGTTCTCCCGGATCAGTAAGAAGCTGTATCTATCACGCAACCGAGATACCGTTTACACAAGATCAACAGGTGGATTTTCCCTTCGGAAGTCCAAAGTTTTGAGTATTGGTGGATCCAATCTGAAATGGTCAAAATCCATTGCAAGCCGCTCCAAGAAAGCTAACGAGGAAGCTACATTAGCTGTTGTGGCAGTAGAAAGAAAGAAAAGAGAGCAGAAAAGTGCTGGTTGTGCAGTCCCCTCTGCGAAGCATAGAAGTCAATCTTCCAGAGAACGCATATTCCGGATTGGCTCAGTTCGTTACAAAATGGACGCATCAAAGCTTACGCTTCAG

AGTCCTGCAAGTGCTTCTGCTGGTGTGATAAAGAGAAATGGGATAAGAAAGACGAAAGACATGGACCATACTTCAGATATATCAAGCACACAGATTTCGGAAATTGATGGAGACCCATTGCCTGGCATTGATTCCTCCCACAGTGCCGATTATGCAGCTGGAGAGATTGGTGTACTTGGTAGAGAAAAAGCTGAAGAGATTGCATTGCCTTCCCTGGTAGAAGAGAAAATCGACTGCTTACCTATAGAACTTCAAACTGCAGTGCGTAATGATATACCACCAAGCGAAACCATGGAAGATGTTGTTTGTGCTCTCCCTGTCAAAGCCGAGTCATCATCTCTCTCAACTTATGTGCCACCAGGAGAGCAGTCCTCAGGCACCTCTATTCCTAATACCAATGATGAAGTGGCGGACTCTGGTATTGGAGGACATGCTGGCATTGATGATCCGCCATGCACAGTTCAGGATTTGCTCATAATGGATTCCAACCACCTACAGGCGTCGAGCAAAAAGCGTTCTAGAGATAATGGACCATCATCAGACCCTGAAGAAGATCAAACAACACACAAGAAGACATTGCTATCGCCACCGTTGCAGAACCTGAAGCCAACATCTAACTTGAAATCCAAGGTAGGAGAAATTAGTGAGAGGAAAACTTTTCAAAGTCCTCCAACACACAAGGTTTTACCCGGCCGGGTTTCCTTTCCTTCACCCTATAGCAAGAAAACTGATCCCGTGACTCCAATTGGAAGGGCTCGAACATGGCATCGAACAAATAATCCATCCTTGTCTCAAAAAATGACACAGGGAGCCTCATATATTCGTAAAGGTAACAGTCTTCTTAGAAGTTGTCTTCCAGGTACCACGGCATCAAAGGTATCTGGTGGCTTAAACACTTCAAGTTCTCATTCTGATTTTGTTGCTAAAGATGAACCAAAAAGTAGCGTGTCAGTTCGAAAAGTTAATGACTCAGACTCTCAGAATTTGATAAAGACAGGTCCAGAGAGGCTAAGAGTGCCTATACTGCCCCATAGTACCAGAATGGTAAGTAGTTTTATCACATCTTCTCAGGTTGCCACACCTTCCATGCTCCCCCACCAGTTGTTAGATGGTTGCTCTGAAACAATACTGGACCCAGTACATCCAAAAGCAGATACACATGCTGGGCCCGCTGAGAATGAAAAGGATGTG---TGCAAGAGCAAAACCCAAAGTGAAATAGCCAATTTGAGATCACAATCATCTGAGATGAAGGCGGTTACATATGTGAAGCGCAAATCCAATCAGTTGGTTGCTTCGTGTGATACTGAGGTTCATGATTGCTCCAACTCTACAGTGAATACTCAGAGTTTACCTTCATCCACACCCTATGATCAGTATTACAAAAAGAGTAAGAATCAGCTTGTTCGTAATGATCCATCACTGAGAAGCCACTCGATTCAAGCAGTTTCCATTCCAGATGGTAATACAAATTCAGAGACTCAAAGAGCTTCTACAGTTTCCTCCCTGAAGAGCGTTAGAAATATAAGTAAGCGAAGGATCAATAAAGCTCTTTCAAAGACACGCAAGCCTTGGTCTTTGGTGTGGACCTTACATGGGGAACAAACACAAAATGAAGATGCCAGTTCACTGCAGCGACATAAGGTTTATCCTTTCCTTTTCCCATGGAAAAGGACGACGCAATGGATATCCAGTAAGAGGCCATTCTCCCGGACCAGTAAGAAGCTGTATTTGTCACGCAACCGAGATACCATTTACACAAGATCAACAGGTGGATTTTCCCTCCGGAAGTCCAAGGTTTTGAGTATTGGTGGATCCAATCTGAAGTGGTCAAAATCCATTGCAACCCGCTCCAAGAAAGCTAACGAGGAAGCTACATTAGCTGTTGCAGCAGTAGAAAGAAAGAAAAGGGAGCAGAAAAGTTCTGGTTGTGCAGTTCCCTCTGCAAAGCATAGA------------------------------------------------------------------------------

1271 CL4704.Contig2_R-chinensis Unigene10533_R-bungei

------------------------------------------------------------------------------------------------------------------------------------------------------------------------------------------------------------------------------------------------------------------------------------------------------------------GAAGTTGACATAGCAAAATCAAGCATAACAAGGTTGAGGGAAGAGGAGGATTCCTTTTCTGAACAGATAGCAGCTGCTAAGAGCAGAGTTGACATTATTTCTTCTGAGATAAACGATAACGAAAGAAAACTTAAGGATATCAAGTATCAAATTCGCAAACTCGAACAACATCAGACTAATCAGGTAACTGCTTTTGGAGGAGATGAGGTGTTGCGTCTTTTACAAAGAATTGAGGAGAATCATAGAAGGTTTACAATTCCCCCTATAGGCCCCATCGGATCTCATGTGAAATTAAAAGATGATGCATGGGGCACAGCTGCTGAAAGCGCCATCGGGAAGTTACTAAATGCATTTATTGTGACAAATCACAAAGATTCTCTTCTTTTGCGAGATTGTGCTAAGAGGGCGGGCTACTCTTACCTGCAGATAATTATCTATGACTTCAACAGACCAAAGTTGAATATCCCCAATCACATGCTGCCAAATACACCACATCCAACTACATTTTCCCAGTTAATTATTGACAACCCAACTGTGTCAAATGTTTTGGTGGATGTGGTTGGCGCAGAGAGGCAAGTGCTTGTTAAGGATTATGATGTGGGTGCAGCAGTTGCATTTCGTCAAAGAATTCAAAACCTTAAAGAAGTTTATACATCAGATGGGTATAAAATGTTTTCTCGTGGATCTGTCCAGACGACTCTTCCGCCACTAAAATGGCAGAAAGCTAGCCGTTTGTGTGGTTCCTATGATGATCAAATTAAGAAGTTTCAGAATGATGCTTCGGTGCTTGAAGAACAAGTACGTCATTGCCATAGCAGGAAAAGGGATGCGAGTGATGCTCTTCAAGATCTTAATATCAGATATGACAGCGCA---------------------------------------------------------------------------------------------

CAACTGGTTAAGGTGGAGAAACTAAAGGAAGACCTTTCCAAAAAGAAAGCTCAAGTTTCTGGTATGATGGAGATAACCTCTGAACTGAAGAAAAAAAAGGAAGAGTTGCGGCAAAATCTTTCTTCGGCAACGAAAAAAAAGCTTGAGCTTGAAGAGGAAAAGTTCCGTAAACGTAACCAAATCCAGAAGATGGTTGATCGTGTGAAGTTGCTTGAAAAGCAAGTTACTGATATTCAAGAGCAACATTTAAAAGATACACAGGCTGAAGAATGTCAGATGGAGGAAGTACTGAAGGGGCTACAGGATGAAGTTGACATAGTAAAATCAAGCATAACAAGGTTGAAAGAAGAGGAAAATTTCTTGTCTGAACAGCTAGCAGCTGCTAGGAGCAGGGTTGAAAGTATTGATTCTGAGATAAAAGATAATGAAAGAAGACTTTGGGATGTTGATAATCAAATTCGCAAACTCCGGCAGCATCAGACTAATCAGGTAACGGCCTTTGGAGGAGATAAAGTGTTGCAGCTTTTACAGAAAATTGAGGAGAATTATAGAAGGTTTACAATGCCCCCTATCGGTCCCATCGGGTCTCATGTGAAATTAAAAGATGATGCATGGGGCATTGCTGCTGAGAGCGCCATCGGGAAGTATCTAAATGCATTTATTGTGACAAATCACAAAGATTCTCTTGTTTTGCGAGAATGTGCTAAGATGGCACACTACAATTACCTCCAGATTGTTATCTATGACTTCAACAGACCAAAGTTGAATATCCCCAATCACATGCTGCCAAATACACGGCATCCAACTACATATTCCCAAATAATTACTGAAAATCCAACCGTGTCAAATGTTTTGGTGGATATGGTTGGTGCTGAGAGGCAAGTGCTTGTTAAGGATTATGATGCGGGCAAAGAGGTTGCATTTCATCAAAGAATTCAAAATCTTAAAGAAGTTTATACATCAGATGGGTCTAAAATGTTTTCTCGTGGATCTGTCCAGACGACTCTTCCGCCAATGAAACGGAATAGAGCTAGCCGTTTATGCGGTTCCTATGATGATCAAATTCAGAGATTTCAGAATGATGCTTCGAAGTTTAAAGAACAAGTGAGTCATGGCCAAGGCAGGAAAAGGGATGCAGAACAAGCTTTTAAGGATCTTAATACGAGATATAACAACGTAAAGAAACAACGAGAGTCTAATGAAAAGGATTTGATTCGTAAGGAGCTAGATGTACAAGATCTGAGGAATACACAGTCTGCTCAAGCTAGTTCT

2527 CL8602.Contig1_R-chinensis Unigene22114_R-bungei

ATAGCAATTGCTCTGTTATTGCTCTGTATTTCATATTCCGGTGAATCACAGTCTCCATCTCCTGCACCAAAGTCTTCTTCTCCAGTGATTTCTCCCTCATCTTCCCCCAAATCCTCTCCCTCATCCCCGAAAGCCTCTCCTCAATTA------------------------------------TCCCCTCAAGCATCAACACCCAAACCCTCTCCGGTGGCACCAGTGATAAGCCCTCCTCCTGCAAAAACCCCATCAGTTTCTCCAGCGAAATCTCCTGCAAGTACACCGGTCGCAACACCTCCTGCACCTGTTCCAACTAGTTCAGCGACGCCTTCAGAAGCACCCATGATTCCCTCTGTTCCATCAACATCTGGTACTCCTGCAGGCTCACCAGAAATGTTCCCAGAA------AGTCCTCCTAGTCCTTCTCCTTTTGCTGCTTCGTCTCCTGACATGGCTAGAGGTCCAGCAGGTGCTGACTCGGGATCTATTTCGAAGTATCAAGTTGGAGTTTTCTTGAGCGGATTTGTGGTCATTGCTGCCATGGTTCTT

GTCGCAATTGCTCTGTTATTGCTCTGTATGTCAAATTCTGGTAATTCACAGTCTCCATCTCCGGCACCAAAGTCCTCTTCTCCGGTGGTTTCTCCTGCATCTACCCCCAAACCCTCTCCCCCAACCCCAACATCATCCCCTCCAACGACAACCCCAAAAGCCTCTCCTCAACCCCCTACATCATCCCCACCAGCATCAACCCCCGAACCTTCTCCGATGGCACCAGTAATCTCCCCTCCTCCTGCTATCACCCCATCACTTTCTCCAACAACATCTCCTGCAACTACACCGGCCGCAACC---------------CCTACTAGTTCAGAGACGCCTTCGGGGTCACCCATGATACCCTCTGTTCCATCGACTTCTGGTACTCCTGCTGGCTCACCAGAAATGTTCCCGGGGAGTATTAGCCCTCCAAGTCCTTCTCCAGTTGCTGCTTCGTCTCCTGACATGGCTAGAGGTCCAGCGGGAGATGACTCGGGTTCGAATTCCAAGTATCAACTCAGAGTTTTCTTGACCGGATTCGTGGTCATTGCTGCTTTGTCTCTT

1669 CL5886.Contig1_R-chinensis Unigene7436_R-bungei

GTGGATTTTAATACAAGTTCTGGAGCCAATGAATCTATGGATGCGGAATGTAGGGAATTTGATAGGCTTCCGGAACATATTAAGTTTCTGGAGATCACTCCACCAGATCCTCGAAAACTCAGCAGTCCCACTGAGGAAGTGGCTGCAGCCGATCAAGAGTCCAGTATTCCCTATGATTCTGGCGCTCCTGAATTAATGGTCATTCACACCCCTTCCAAGAAGGAACCGCCTCGATTTATACGGAAAAGAAAATGTTTATACGATGAAACCGTTGTTTTGTCAAACAAGGTAATGAGACAGAGTATACACGATGCAAGTGATTTAGTAGCTAAACGAACAAGTAGTCCAGTAACTTCTTTGGATGTTTGGAAAGCATACAAGCTTCCAAAGTTTTATCAGGACTTCTTGGAGCCTTTGCTTCCTTGTGCACCACAGATTGGATCACTCTTCCGTAAAAGGTTTATGAGTTCTCCTCAGATAGTCACTAGTTTAAGAGAAAACAGGTCTAAGACAAGAAAAAGGCGTGCAGCAGAGAGAGCACAATCACTTGAAGCTCAAGAAGTAGCCATGAAGTCAGGAGAGGATAGACGTGAGCCACAAAGCAAATTCTCACAAGAGAGAGCTCATTCAATCGAATCCCCAGAA------------------------------------------------------------------------------------------------------------------------------------------------------------------------------------------------------------------------------------------------------------------------------------------------------------------------------------------------------------------------------------------------------------------------------------------------------------------------------------------------------------------------------------------------------------------------------------------------------------------------

---------------------------------------------------------------------------------------------------------------------------------------------------------------------------------------------------------------------------AAGGAGCCGCCTCGATTTTCAAGGAAGAGAAAACCTTTATTTGATGATACCGTTGTTTTGCCTAACAAGGTAATGAGACAGAGTCTACATGATTCAAGTGACTTAGTAGCTAAACGAACAAGTAATCCAGTAACTTCTCTAGATGTTTGGAAAGCATACAAACTTCCAAAGCTTTATCAGGACTTTTTGGAGCCTTTGATTCCTTGTGCACCACAGATTGAGATACTCTTCAATAAAAGATTTATGGGGTCTCCTCAGAGACTCACTTGTTTGACAAAAAACAGGTCTAGGAAAAGAAAAGGGCATGCAGCAGAGAGAGCACAGTCACTTGAACCTCCAGAAGGGACCACGAAGTCGGCAGAGGCTGGATGTGAGCCGCAGAGAGAACTCTCACAAGAGAGAGCTCATTCTATTGAATCCCCAGGGGAAGGTAGACGTGCGACACATAGGGAAGCTTCAGAAGAGAGAGATTACTGTGTAGAAACCCCGGGAAGTCCCAGTAGGCCAAAAGACAGCAGACACGAGGAAGATAGGGAATTTACACTTGAGGCAGTTGACTCACGTTATCCTTCAGAAGGTCGTCCTGATGGGTCAATAGAGCATGGGTATAAAGCACAGTCACAAGAGAGAGCATACTCTGAGAAAAATACGTTGGATGATACAACATGGTGTCCTTCTGACATTCCGATTCCGACAACGAGTAATGTTGATATGGCGGTAGTGCCCATGACTTCGCTCCCTAGAAGGGATGATGATATTGATCTCATAGATGAGGATTTACACCACGAAGATGTTCTCAGTGAGAATCAGAATGGGTGGTCAGCAAGAACAAGTACGGTGGCACGTTTTCTATCTAAAAGCTTTTTAAAACAAAAAGAAAGCAATGAGGGTGCTGAAGTTCTGAATATGAGCCCGATTATGAAAGGCAAAACACGAAAAGAAAATGCCAGAATATTCTACGAGATACTGGTTCTGAAATCTGGAGGATTTGTGGATCTGAAGCAAGACAAACCTTACGGTGATATA

1077 CL4126.Contig1_R-chinensis CL6929.Contig2_R-bungei

ATGGCGGAAGAGGAAGAAGAAGAAGAAGTACACGTAGACACTGGTTCTAATCCAGTACACTCTCATTATTTGGAGATTGTTTGCAAAAGTTCTGGTAAGATTAGAAGATTTGCTGTGGGAACAAAAGCTGGATTTGCATTACAATTGATCAATCGGAAATCCGGTGTTGGGAAGGCTTTAGGTGTTTATATTGAAGCTGGTAAAGAAGGAGAGGAGCCTATAGTGTTTGGTCCTAGTGCTTTTCTTGTAGATTTTGGTGATGGTTGGACTCTGCAGACAGTTTTAGATCAAGGTCTTGAAAAGGAGAAAGAGGAGTTGTTAGTGAAAAAGAAGGAGATTTTAATGGTTAAGCCTTCTCCGGATGTCATTAAAACAGCAATACTGAACAGATCAACTAGCAGCAACGAAGGCAGCTTCCGAACAACTAGGAACATCAGTTATGAGTATATTGGTAGAATTTTGGTGGTTTTGATCTTCATTATTATGCTTGGCTCAATCTGCACATTAGCCTTGGAAAATCTTCCTAGGTTAATCCTTTTCATCAGCTCATCAATG

------ATGGCGGAGAAAGAAGAAGACGTACACATAGCTACTGATTCTTACCCTGTACAACCTCAGTTTTTGGAGATTATTTGCAAAAATTCCGGTAAAATTCGTCGATTTGCTGTGGGAACAAAAGCTGGATTTGCATTGCAATTGATCAATCGGAAATTGGGATCTAAAAAGTCTGTAGCTGCATATATTGAAGCTGATAAAGATGGTGAGGAGCCTATAATCTTTGGGCCTACTGCTGTTCTTGAAAATTTCGGTGATGGTTGGACTTTGCAGACTGTTTTAGATGAAGGGTTTGAAAAAGTGAAAGAGGTGCAGTTTGCGAATCAGCAGGAGGATTTAATGGCTAAGCGTGTTCCGGATGTCATTCAGAAAACAGTAGTGAAAAAATCGACAAGCAGCAACAGCGGCAGCTTCGCAGCAACGAGGAACATCAGTTACGAGTATATTGCTAGAATTTTGGCGGTTTTAATTTTCATTATTATGCTTGGCTCAATTTGCACCTTAGCCTTGGAAAATCTTCCTAGGTTAATCCTTTTAATCAGCTCATCAATG

4479 Unigene19401_R-chinensis Unigene17923_R-bungei

---------------------------------------------------------------------------------------------------------------------------------------------------------------------------------------------------------------------------------------------------------------------------------------------------------------------------------------------------------------------------------------------------------------------------------------------------------------------------------------------------------------------------------------------------------------------------------------------------------------------------------------------------------------------------------------------------------------------------------------------------------------------------------------------------------------------------------------------------------------------------------------------------------------------------------------------------------------------------------------------------------------------------------------------------------------------------------------------------------------------------------------------TCATCAGTGGGTAGTGGACGATCTACTCACGAGCCATCAATTCAAGATCAAAGTATGCGGAGGAATGAAGAACAGCATGTACATGGTGCCTCTGATAGCTTGTTGGAAAGTGACGATATTACGAGAAGAAGAAATCAGCATTCTGCTTCATCGATTGGTAGTGGAAAATATTCCCGCGAGTCATCCATGCAGGAAGAAAAGACACTCAGAGATGAAAGACAGAGAACACGTGACACGTTCGACAACACAGTGAAAAATGATGATCTTACGAGGAGAAAAAATAGGCCTACCACTTCATCATTA---------AGTGATGATGAAGAAGATCTTGAAAGAAGAGATGTATACCATAACAAACATTCTTCCGAGGAAAGCGAAATTCAGGCGAGCCACTTCGATGAACAGATCAACATACCTGGGCATATTGACTCCATGGAAGAAGGTATTACAAGACAACCGAGCAGGACTTCCTCATCTTTGTCAGATGATAATGTTGATGTCATGCAGAGTACCACCAAGGATAGTGCTCCTATTGTTTTTGATGAATATGTATGGGATGATGAGAGTACTGGGCATAAAGAAGTTTTCTACGAT------------TCACCACCACGTACCAAGTCGCCCCCTAGATTTTCAGAAAGTTCGAGTATTTGGAGCCCTAGAAGGGACAAGGCCGAGTCTAACTTCCCTTCGGAGTTTTCTACAAGAAAGAATGGAACTTCATCGCCTACGTGGAGCCCTGATCGGAATAGTGGCGGCTCACTCGAGAAATCATCCAGTAGTAACTCAAATCTGTTCACAGATTCTAAAACTACTTCTGTGTTTTCTGCAAGTGAAAATAAGGCATCTTCTCCTTCACAATCAATTGACTTGCCACCAGTAACTTTTGATGAATCTGATGGGGTGGAATCTGAGATTGAAGAGGAAGTGGAAAATGCCAAAAGCCCAGAACCAACTAGGCATGCCTCCTCTTTTCCGATAAAGGGGGCCCAAGTTCCCTTGAGGAAGCCTTCCTCATATGTCTCATCTGATGATTCAGACTCTGACAATACATACCCTAAGAGAAACCAAAGAAAGCAAGATAATGTTCGAACTGAATCTCTGAAAGAGGATAGGCCATATAGTCCAAAGGTTGAAGAGAAACAACCATCTATTAGATCATCAAGGTACTCCCTGAATCAAGATCGTAAAAACAGTGATGATTTTGATGCACCAGTTTCACAGAATAAAGTAGAAGATTCTGAATTAGCAGTGGACTTTAGCTCTGAAAGTGAAAAGGAGTTTGATATAGGAGGACTAAGATTGAGAGGCGGTTTACGAAACAAGGGTTACAAACGGCCACCTTATCACAAGGATGCACCCCAACAA---------GACTCGATTGAGAAATCAAAGGGCCATCGTCAGCCTTCCATTCATGGAGAAAGGAGCTCCAGATATTCTGGAACAACATTTGCGTCA---GACAGTGATGATACCGAAGAGATCAAACGCCAAAGCAGTGGGAGCAGGGTTAATAAAGATACAAGCTCAAGAACTTCTAGAACAACTTTTGCATCAGAGAATGTAGATACTGGAAGGTTAGAACGCGGAAGTAGTGGGAGCAGGGTCCATAAAGATACAAGCTCAAGAGCTTCTAGAACGACTTTTGCGTCAGAGGGTGTTGATACTGAAAGGTTAGAACTTGAAACTAGTGGGAGCAGGGTTCATAAGGATACAAGCTCAAAAACTTCTAGAACAACTTTTGCGTCAGAGAATGATGATAACGAAAGGTTAGAACGGCAAAGTAGTGGGAGCAGAGTTAGACCACTTTCTCGTAGGACCACCAGGGTACCTTCAACACTAGCAGGTGTATCTGGTCCTTCAACGTCTGTAAGTGTCTCTAAAGCAGAGGCATTGGAAGAAACCAAAACTGTACAGGTCGATCCTCAACCTGAGAAAAGCAAGAGATCATCATTCAGGACTAGTGGCTCTGCAGAACAAAAGCCCCTCCTCTCTTTTCAATCAAGACAAGAAGAGATACCCATGTCACCAACACGGGCTGAGCGCCCTCTCCGTGAAGAGAAGTCACAGCCATCACGATATTCAGAA

GTTCTTCACAGAAGCTTCAAGCCTGCAAAATGCAAAACATCGTTGAAACTTGCGATGGCTCGATTGAAACTATTGAAGAACAAGAGAGAAGTACAAGTGAGATCGATGAAGAAGGATTTGGCTCATCTTCTCGAAACCGGTCAAGAGAAAACCGCTATGATCCGGGTTGAGCATGTTGTGAGAGAGGAAAAAACAATGGCTGCTTATGATTTCATTGATATATATTGTGAGCTTATTGTAGCCCGCTTGCCGATCATTGAATCGCAGAAAACCTGCCCAATCGACTTGAAAGAGGCCGTGTCTAGCTTAGTGTTTGTATCACCTAGATGTGCAGACATACCAGAACTGGAGGATGTTGGCAAGCAATTCACTGCAAAATACGGAAAAGAGTTTATCACTTCAGCTCTTGAGTTGCGACCTAATTGTGGTGTCAACCGTACGGTGGTCGAAAATTTATCTGCTAGAACACCTGATGGTCAGACCAAACTTAAAATCTTGTCCGAGATTGCCAAAGAACATAATATTGAGTGGGACCCTAAAGCATTTGGAGATAAAGAACTGAAGCCTCCAGATGACCTACTGAATGGTCCAAAAGTAAATGAAGCCACTGGTGGGGGAAAGGCAGAGACCCTTCCAACACATCTTCTTTCGTATGGAGGGACACCTGGACCAACTAATGCAGCAGATACCACAGGGTCTTCATGGGGTTCCCACACAGCTGATCCTACTAATGTTAAATACTCTAACGTGACAAATAAAACTTCAGGTAATGTGGTAGAAGGTAGAGATTCAGCACATCAAGATTCCCAGAATACCAACCAATATTATCCTAACAGACAGAACTGGAACATGGAATTTAAGGATGCAACATCTGCTGCACAGGCAGCTGCTGAATCTGCTGAAAGAGCAAGCTTTGCAGCTAGGGCTGCAGCAGAACTTTCGACCTATGGGAATTTTTCAGGGCAGTATTCCACAGAATCACATGATCAAATGATGCAGGATCAACAGAGATACAGAAATGAGAGAGAGAACGTACAAGGAGTCTCTGGCAGCTCTCTGGAAAAGGAAGATAGTATGAGGAGAAGAAATAAACACTCTGCTTCATCAGTGGGTAGTGGGCACTTTTCTCATGAGCCATTGGTTCAAGAACAAAGGACACAGAGGAATGAGAATAAGAACGTGCAAGGTGTCTCTGACAGCAAGTTGGAAACTGAAGATATTACG---------AGTAACCATTTTGTTTCATCAGTTGGTAGTGGGAAGTATTCCCGTGAGCCATCCATGCAGGAGCAAAAGTTACACAGGGATGAAAGACAGAGCACGCAAGGCACGTTTGACAATGCAGTGAAAAGTGAAGATATTACGAGGAGAAAAAATAGGCGTTCTTCTTCTTCATTGAGTCATAGATCTGATATTGAAGATGATCTCGAAAGAAAAGATATATACCACAACAAACATTCTTCGGAGGAAAGCAAAATTCAGTTAAACCGCTTTGATGAACAGATCAACAGACCTGAGCGTACTGACTCTGTGAAAGAACGTATTACAAGGCAACCGAGC---AGCTCTTCTTCGTTTTCAGATGATAATGGAGATATCACGCGGAGTAGCAGTAACGATAACCCTCCTACTGTTTTTGATGAATATGTATGGGATGATGAGGGTACTGCGCAGAAAGAAGTTCTCTACGATTCGCCACCACATTCACCACCACGTGTCAAATCACCCCCTCATTTTTCAGAAAATTCAAGTATTCGGAGTTCTAGAAGGGACAAGGCTGAGGCTAATTTCCCTTCTGAATTTTCTACAGGAAGGAATGAAACTTCATCACATACGTGGAGCCCTGGCCGGAATAGTGGCGGCTCACTCGAGAAATCATCCAGTGGTAACTCAAATTCATTCACAAACTCCAAAACCGCTTCTGTGTTATTTGAAAGAGAAAATAAGGCATCTTCTCCTTCACAATCAATTGACTTGCCACCTGTAACGTTTGACGAGTCTGATGGGGTGGAATCTGAGATTGAAGAGGAAGTGGACAATGCCAAAAGCCCAGAATCAACTAGGCATGCC---TCTTTTCCGATGAAGGAGGCCCAATTTCCATCGAGGATGCCCTCATCGAATGTCTCATCTGATGATTCAGACTCTGACTATACATATCCTAAGAGAAATCAAGGAAGGCAAGGTAGTGTTCGAACTGAATCTCTGAAAAAGGATAAGCCATATAGTCCAGTGATTGAAGAGAAACAACCATCTATTAGATCATCGAGGTACTCACTGAATCGGGACCGTAAAAACAGTGATGATTTTGATGTACCAGTTTCACAAAATAAAGTTGAAGATTCTGAATTAGCAGGGGACTTCAGCTCTGGAAGTGAAAAGGAGTTGGATTTAGGAGGACTGAGATTGAGAGGTGGCTTACGAAATAAGGGTTACAAACGGCCATCTTATCGCAAGGATGCATCCCAACAGACTAAGGAGGACTCAAGCGAGCAATCAAAGGTCCATCATCAGCCTTCCATTCATGAAGATACAAGCTCAAGATATTCTAGAACAATATTTGCGTCAGATGATGATGATGATACCGAAGGGATCAAACGCCAAAGCAGTGGGAGCAGGCTTCACAAAGATACGAGCTCAAGAGCTTCTGGAACAACTTTTGCGCCAGAGAATGTCGATACTGACAGTTTAGAACGCCGAAGTAGTGGGAGCAGGGTTCATAAAGATACGAGCTCAAGAACTACTAGAACAACTTTTGCGCCAAAGAATGTTGATACTGAT---------------------------------------------------------------------------------------------------------------------------------------------------------------------------------------------------------------------------------------------------------------------------------------------------------------------------------------------------------------------------------------------------------------------------------

3759 Unigene15554_R-chinensis Unigene19931_R-bungei

---------------------------------------------------------------------------AGTTATAAGATGCCCTCTTTTGTGGATATTGTCTCAAGAGATGACGTTGATCATAAATCTTCTATCCTTGGTGAAGGATTCCTTAACAAATGTGAATATGGAGATCGGCTTGCAAGTGAAAATGAGTTTGAGGACCTAAGGTCAGAGAGAACAGTTGGCATTTCATTTTTCCCTGAATTATTTGAATATGGCCACTCAACAAGGCAGCAGAGTATAGAAGATCAATTGTGTTCACCATCTTTTGATAATCTACCCCGAAAACGTGTTTCCGTAGGTCCAGATTATCAAGCTGTTGTTCCAGAACAGTGTACAATGATGGAAACTGTCGAAAATGAAAGAATGTTGGGCACTTGTGTTAATCCAATGTCTGAAGAATCCTACACATCTATCATTGTTGGAAGAGGCCGAGGGGATTGCAGCTGTCATGATAAAGACTCCTCTAGATGCGTGACACAACATATTGAGGAATCTCGAGAAAACTTGATAGCAACTCTTGGCAAAACAGCTTTTATGGAGTTGGGCTTTTACGACATGGGAGAAAATGTGGCAAGTAAATGGAATGAAGAGGAAGAAGACGTATTTAACGATGTTGTAGTCTCGAACCCTGCCTCTTCGGGTAGAAACTTTTGGGATCATCTCCCCGTCGTCTTCCCTTCTCGAACC---------------------------------------------------------------------------------------------------------------------------------------------------------------------------------------------------------------------------------------

ATGATACAGAAACGTCCTTTTGGTGATGGAGAATCCCACGACCAACCTTGGAAGCAGCCTAGACATTTTGAGTGCAGTTACCAGCTGCCCTCTTTTGTGGATGTTGTTTCAAGAAATGATGTTGCTCATAAATCTTCCATC------CATGGATTCGTTGACAAATGTCAATTTGGAGATCGACTTGCGAGTGAAAATGAGTTTGAGGACCTAAGGTCAGAGGCAGCGGTTGGCTTTTCATTTTCCCCTGAATTCTTTGAATATGGTCACCCAACAAGGCAGCAGAGTATAAAAGAGCAATTATATTCACCATCTACCGATTTTCTGCCCCGAAAACGTGTTTCCGTTGGTCCAGATTATCAAGCTGTTGTTCCAGAACAGGGTACAAAGATGGAAGATGTCGACAATGAAAGAATGTTGGGTACTTGTGTTAATCCAATGCCTGAAGAATCTTACACATCTATTAATGTTGGAAGAGGCAGAGGGGTTTGCTGTTGCCAGGATGAAGGCTCCTTGAGATGCGTGATGCAACATATTGGGGAATCTCAAGAAAACTTGATAGCAACTCTTGGGGAAACAACTTTTATGGAGTTGGGCTTTTGTGACATGGGAGAGAATGTGGCTAGTAAATGGAATGAAGAGGAAGAAGACGTCTTTAACGAGGTCGTAATCTCAAACCCTGCCTCTCTGGGTAAAAGCTTTTGGGATCATCTCCCCGTCGTCTTCCCTTCCCGAACCAAGAAGGAGATTGTCAGCTATTATTTCAACGTTTTTATACTACGAAAACGTGGTGAACAGAATAGATTTGATCCTTCCAATATTGACAGCGACGTTGACGAATGGCATGATTCAGTGGTAGTGTCCCCCCATCATCATTTCGATGATGATCTTGAGATCAGTGAAGATGAGATGAGTGAAGATCTCGAGATCAGTTATGACAACAATCCATCTGCTGCTAAGGTTGATGAT

3357 Unigene5762_R-bungei Unigene11922_R-chinensis

------------------------TTGTTTCTGGGAGCTCTGCTTCAACTATTATGTTCCCTTGTACACCAAACCAGTTCTTTTAATGAGCATCCAGTTTTTGGGAAAATCGTCAGTCTTGTTCCAGAGCTCCTATGTTGGTGTTTCGACAAGAAGGGTGATGATACTCGCACAACCTCTTATTCATATTTGAATCACAAAATGTTGATGCTCATGACCAGGCTCAGCGTCCAAGTGCATCAAGAGTCTTGCAACTTTGTTTTGTGGTTGCAACTCTTACACAAATATTATCAAGATCTTCTCCATCTACCTATATCTCAAAGTCCCGCCGTTGTTGATGTTTGCCTGGAGGGGTCACCGTTTCTGACATGTTTTTCGGATGGACAGAAAGTTGATTCCCATCTGCAAAGACAGGCCATCTTTCTTTTTCTAAAATGTTCCATCAGTTTGATTAGAGTTGATGATGAAACTGATACAAAACAATCATGTACTACTTCAGACTCTTGCACATTGGTACCTGCCCAGGAAAGTTGTTCTAGGAAGATCGGTTTATTAGAAATGTCTGCATGGCTTAATAAGCATGTGCCTCTGGATGTGATTATCAGCTACGAGTTGTATTTGGATGAATGTAGCCGTTTCACTTTGTCCTTCCTTAGGCTTTACAGGGACGAGGACGATTTTTTATTTGAAGTTTTACTACTGCTCTTTTCTTTACCA

TTTGATTCTCCAGTCTCGAAGTTTCTGTTTCTGGGATCTCTGCTTCAACTGTTATGTTCCATTGTTCACGAAAGCAGTTCCAGTAATGAGCATCCACTTCTTGGAAAAATCAGCGATCTTGTTCCAGAGCTCCTAAGTTGGTGTTTTGACAAGAAGGGCGACAATACTTGCACGACTTGTTATCCATATTTGAATCACAAAATGTTGATGCTCATGACCAGGCTCAGCCTCCGAGTGCCTCAGCAGTCCTGTAACTCTGTTTTGTGGTTGCAACTCTTACACAAACATTATCACGATCTTCTCTACCAGCCAATATCTCAAAGTTCCGCTATTGTTGATGTATGCCTGGAGGGCTCACCATTTCTGACATGTATTTCTGATAGACGGAAAGTTGATTCCCACCTGCAAAGACAGGCCATCTTTCTTTTTCTAAAATGTTCCATCAGTTTGATTAGACTTGGTGATGAAACT------------------------------------------------------------------------------------------------------------------------------------------------------------------------------------------------------------------------------------------------------

4635 Unigene33218_R-bungei Unigene20204_R-chinensis

XGTGTTTCAATTGGCGCGGTTGATAACTTGAAATCGGATAATAAATGTCTTGAAACTTCTCCAATTGAACAGTCTGAGGTGACGGTGGCGGATTTAAATCAGGTCTATGCCGCAGATGTAACAGTTAGAGATAATGTTGCGATTGAGAACGCGAAAGATCTAGTGCCAATTACAGCTCCGGAATCCTTGCTGCTAGACACCCTGAAACAATTGCACAAGGTATCAGATCAATCCCCAGAATCAGATCATCTTGGAGCTGTAGGAAATAATGTGCGGAAGAAATATCCTCCTCGACGAAGAATTTCAGCTTCACGAGATATTCCTTTACGTTGCACCACCAATGCTTCATATTCTATCCAAGAAGTTAATAACTCAATAAATATCGGCACAAGTGATGACAAGGTTTGTGGGGAGAACATATTAGXX---------------------------------------------------------------------------

------------------------------------------------------------------------------------------------------------------------------------------------------------------------------------------TTGCCGCTTGACACCTCGAAACAAATGCATAAGGTATCAGATCAATCCTCAGAACTAGATCATCATGTAGCTGTAGAAACTAATGTGCGCAAGAAATATCCTCCCCGACGAAGAATTTCAGCTTCCCGTGATATGCCATCATGTTGTACCACGAATGCTTCATGTTCTACCCAAGAAATTCATTCCTTGATGGATATTGGCATGCGTGATGATAAGATTTGTGGGGAGAACATACTAGAGGTGCTTGATAAAAGTGAATCAAAGAAAACAAGAGAGGGAATTGTTGAAATCACACACGATAGCATGCTACAGGGG

9444 Unigene7375_R-bungei Unigene40018_R-chinensis

AATCGACAGCTACAGATGGGTTTCTGCAGAGGCAAGCGTGTTAAGGTAAGCAGTAGGATGAGTGAATTGCCCGACGAGATCCTTTCGTTCACTCTATCCTTTTTACCCGTCAGGGAAGCAGCCAGAACAAGCATCCTCTCATGCAGATGGCGATACCTATGGAAGACATCCAGGTCATTCTGTTCCACTCTCACTCTCGACCCTTTAAATATGAGAGGTTGTGACTACCCAGAGGGCATGTTAGGTGCTGTTGATGATAAAACCTACATGTACAAGGTTAATCCTAGTGTGCTCAAAATAGAAAGATCTAAGTTTGTAGAATGGGTTGACATGATAATGGACCTCCACTACCTTCCTGTTTTACACTCCTTTCGCCTTCGATATCATTTCCGCAATAAACATGCTGCCAATATTGATCGCTTTATAAAGAGGGCAATACAAAAGGGAGCCCAGAATTTCGATATAGATATGTCTTGGTTTCATAAGGATGATATTTATCAGACTAGAGCCTTCTATACGTTTCCTGATGGACTGTTTACAAAGGAAACAGGGATTCAAGTCAAGTCTTTGTGGCTCAAATCATGCATTTTTGGGCCATTCGATTTCAACAGCTTTGGCTCTCTTTTGGATCTCAATTTGGAGAATGTTCGTCTAGATCAGGATATATTTAATAGCTTTCTGCTCACTTGTCCCACTGTGGAGAACCTATGTTTGACCAATTGCAGTGGTCTGTTCAAGTTTAAACTTTCTGGCCCGCACACTCGACTCAAATCTCTGGCTGTACTTCACTGTGATGGAATGATTGAAATTGAGATTCATGATGCCAAGTACCTTGCAATATTTGAGTATCGAGGGAGGCAAACAGTTTTTCGTAGTTTTAAGGTTCCTCGACTTCTGAATGTGGTTTTCGATGTCCTTGCCTCTAAGCATTCCCCCATGGTAGAATCCATTTCCAGTGGAATTCCCAATTTAGAATCACTGCTGCTATCGTTTGGACCTCATCACGAATACATAGTGCCTAATAACCAGTTTGCAAATCTGAAGAATTTGGAACTGACACAACACATGTTTCGCGGATCTCTCTGGCAATATATTCTCCTGATTCGCTCAGCCCCTTACTTGGCTACTCTTGTGTTTAATTGGTTCTTCTTCCCACATTTCGAGCCTAAAATGGACCCGGAGCCAGAG------------GCTTTTGACTCTCCTCACTGCCACCTCAAGAAGATGACGCTAACTGGTTTTGGTGCAAGTGATCATGAATTTGAGTTTGTTAGATACATGCTCAATAATACCGCATCTCTTGAGGAGATTGCAATTAGTGTCGACTTTAAAGGA---------

------------------------------------------------------------------------------------------------------------------------------------------------------------------------------------------------------------------------------------------------------------------------------------------------------------------------------------------------------------------------------------------------------------------------------------------------------------------------------------------------------------------------------------------------------------------------------------------------------------------------------AACTCTTTCGCCTCTCTTTCGGATCTCAAATTGGATAATGTTCTTCTTGATCAAAATAAATTTCATAGCTTTCTGCTCAGTTGTTCCAGTGTGCAAAACTTGTGTTTGACCGATTGCAGCGGTTTGCACAAGTTAAATATTTCTGGACCACACATTAAACTGAAGTCTCTGAGTCTACGTGACTGTGATGGAATTATGGAAATTGAGATT---AATGCCAATCACCTGGCAAGATTCGAGTTTAATGGGAGACGAACAGTGTTTCGTATTTGTAAGGTTCCCCGACTTGTGCATGTGATTTTCGGTGTCCTTGACTCTAATCCTTCCCCCATGGTACAATCCATTACCCGTGAAGTTTCCAATTTAGAATCATTGATGCTATCGTTTGGACCTCATAAGGAATATATAGTCCCTAATAACCAGTTTTCAAATCTGAAGAATTTGGAGGTTATACAAGACCTGTTTCGCGGATCTCTTTGGCAATATATTCCACTGATCCTCTCAGCCGCTTACTTGTCTAATTTAAAGTTGAATTGGAACTTCTTCCCCCATTTGGCATCTGAAATGGAGCTGGAGCCAGAGCAGGAGCCAAATGGTTTTGACTCCCCTCACCGCCACCTAAAGAAGATGACGCTGACTGGTTTTGGTGCAAGCAACCATGAATTTGAGTTTGTCAGATACATGCTCAATAATACTGCTCATCTTGAGGAGATTGCTATTAGTTTCAACTTTAAAGGATACGATTAT

5825 Unigene46897_R-bungei Unigene28993_R-chinensis

---------------------------------------------------------------------------------------ATGCTAGGCAAATTTTCAACAAGGGAATCATTTAAAGGCGGAGAGATAGAGAGAAATTCTCGTTTTCTGCCTTACATGATACAGATGGGTAGGTACCTTGTCGAACAAGAAAGCTTAACTCAGAGACAGTCCATGTCCAATGCTATTTCATCATGTCTAACATCATGCTCAACTCAACCAAATTACCTCCAACTCATGATGGTGAACTCACTTCTCTTGGAATCGTACAATTCCTGGTTGACTCACCGC---------------------------------------------------------------------------------------------------------------------------------------

TGTGTTAGTGAGTATTGGGGTAACCTTAAAAGGGTTGGGCGTGGTGGTGGAAGTCGTCTTCAGTTACTGTCTTATGATCTTCTTCTGATGCTGGGCAGATTTTCAACAATGAAATCATTGAAATGCGAAGAGACAGAGAAAAATACTCGTTTAATCCCTTTCATGATACAAATGGGTAGGTACCTTGTTGAACAAGAAAGGTCACCTCAGAGACGGATTAAGCCCAAAGCTATCACCTCTTGTCTGCAGTCTTGTTCGACTCAACCAAATCAACTCCAACTCCTGATGGTGACCTCGCTTCTCGTGTTATCCTATGAGTCCTGGTTGACTCATCGTCCAGCCTTTCTGGAATGCGGAATTGAACTTGCATATATGCAGCATAAGAATAAACAGCCATTATTCTCTGAACCAGTAGCTGCAAGTGAAAATTTTTGTATAAAGCCGATACTTGTGTACATTGGATTGATCGAG

2072 CL7141.Contig10_R-chinensis Unigene27798_R-bungei

GTTTGTAGATTGTGTCATCGGAAGACTAATCACCCTCGTGGGGAGTGTCCCTCCGGAGATTTATGTGTGGAACCTAATAGAAACAGTGGTCCCTGCAGGCGGATACCCATTGAGTGCAAGCCCCTTTGCAAAGAGAATGCAATAGAAAATGGACTTCGTTTTGGTTGGGCCCCTGAGAGTGAAGATGAAGATGAGTTGCAGGCTAAATTTGGGGAATATGGGGAAGTGAAAAAGCTCGTTCTTCTTAGAGACAGACGGACTGGTTTGTCTGATCGCTGTGGTGCGGTATTTTTTGTCAACATAGACGATGCTGAAAGAGCCTTGAATGCGCTA

GTTTGTAGAATGTGTCTTCAGAAGACTAATCACACTAGTGAGGAATGCCCCGCGGGTGATCTTGGTTTTCTGTCTGATGAAAACAGGGGTCCTTACCAGCGGATACCTATTATTTGCAAGCCCCTGTGCAAAGAGAATGCAATTGAGAATGGGGTTCGTTTTAATCTTGCCCCTGAGAAAGCCGATGAAAATGAGTTGCGTGCTATATTTGAGGAATATGGGAAAGTGAAAAAGCTCTATCTTATGAGAGACGAAGTGACTGGTGTATCTCAACGCGCGGGTTTTGTAAATTTTGTCAATATTGACGATGCTGAAAGAGCTTTGGATGCGCTA

9699 Unigene24005_R-bungei Unigene41836_R-chinensis

XXGCCTTTGAAGATTCTAGACCCGGTTGATGGATTGAGTGATGATAGTGATATTGATTATGAAGATGATTACGAGACTTGGTCTGCGATTGAGAGGCGGTTCTCGAGTTATACGCCGGCAAGCTTCCCAGAGAAAAACGCAGATATCATTTCAAATGAAATTGGGGTTAAAGGTACTTGCACACCCTTGGGCTCTGAAACTCCCTATGCCTCTGTAAATAAAAATGTTTCAGCGGTTCTTGGTGATGATTATCTACAAACGGAAGAACCTTGCAACAACTCCCTTTCTCTGGTATCTTGTAATGATACAGATAACTGGAACCAGCCTTTTCGGTCTGTAGACGGGCACCAA------TCTCCAAAGTCGCCAATACTGCCATCAAATTATTCTGGCTTCCGTCACTCCAAATCCTCACATTATTTCATTGATGCTATCAAGAAGAACCGGTCTTTX------------------------------------------------------------------------------------------------------------------------------------------------------------------------------------------------------------------------------------------------------------------

---------------------------------------------------------------------------------------------------------AGTTATTTGCCAACAAGTGTCCCAGAGAAAGATAGAGATACCATTTCAAATGAAGCTGTGGTTCAAGGCACCTGCGCAACCTTGGGGCATGAAACTCCCCATACTTCCGTAGATAAAGATGTTCCCTTCATTCTTGGTGATGATTATCTACTAACGGAAGAACCTTGCAGCAGCTCCCTCTCTCTGGTATTGATTAACGATACAGATAACTGGAACCAGCCTTTTGGTTCTGCGGATGAGCACCAACCTGAAGCATCGAAGTCGCCAATACTGCCATCAAAGTTAACTGGCTTCCGCCATTCAAAATCCTCACATCATTTCATTGATGCTATTAAGAAGAATAGGTCATTCCAGAAACTCATTAGGAGTAAAATGATACATATTGAAGCAAAGATTGAGGAGAACAAGAAATTAAAGGAGCGTATCAAATTATTAAAGGATTTCCACTTTTCATGCAAATTAAAAGCAGGGAAAGCTTTAACCCAGAAGAAAGATGCTAGGGTTCAGTTAGTATCACTGTGCAATTCCAGGAGCTCACGCTCTTCAAAGGGTAATGATAAGAAGGTTTCTCCTTTAAATTTTGGCCCAGTTGAAAATTCTCACGTTGCC

10224 Unigene7913_R-chinensis Unigene28210_R-bungei

---------------------------------------------------------------------------------------------------------------------------------------------------------------------------------------------------------------------------------------------------------------------------------------------------CAAAGTGAGCGGAACATACTCAAGAGTGAAAGTGATCACTTAAACCAGCAGTTTGGAGTTTGGGAGGAAACTCTGAGCCGGAAAGGAACGGAGCTGATAGAAACAGAACGGAATCTCGAAGCTACTAAAACTGAGAATGCGGAGCTATACAGAAAACTGGAGAATACAAATAGAGAATGCGATGAAGCTAGACCAAGAATATCAGTCTTAGAATTGCACAATTTGAGAATTGGAGAAACTAACCTTCATCAGCACGAGGAAATCAACCGTCTTCATAAAGTGATCGAAAAGCTTGAGTTAGAAATGGGGAAATTACTCAAAGAAATTCAGGAAGGAAGAGTTAGAGAAGATTTTTTGAGCTCTGAGGTGCAACAGATAAAAAGCAAGCTCAAACTATGGGAGCATGAGGCTGAAGCACTATATTCTAATTGGCAGATTGCCGACACGCATGCAGTTCTGTTTGAACAGAAGTTACGTGAGCTTGCTGGAGTATGTGTGAGTGTTGAAGATGAAAGAATTTTAAACGTGCAGGCCATTGCACAGATGAAAGAACGAGTTCGGGTGCTGGAGTTGGAAAATAAAGGAACAAAATCTGATTTGGCTGCATATCTTCCTGCTGTAACTTCTCTGAATGACAGTGTAACGTTACTTGAAGATCATGTATTTTCAGCAACGGAGGAA---CAAGCTGACAACCATGAAATTGAGGCTGCTAACTCATCAAGTAATCTCCAGGACAACACCAACAAAGAAAGATCAGGGGATCAAAGCCTTATGGCGGTAGATGGAGTTTCAAATTTACTTGCATTGCAGACCCGGATCAAAAAAATCGAAAATTCAGTGATTGAACAGAAAAAGTTGCAAATGAAGGAAAACTTTGATAGGAACATAAAACTTGATGCTGCAATTAGAGAGATCCAGAAATTCGAGCAAAGTGGCCGAACTCTTCACAATACTGATCCCGAATCTTCGGTGAAAGATATCCCACTTGATCAAGTATCACATTCCTCCTCCTACGATCGACACACAATGAGTAAGAGACAAGATTTCAACAGAAGCAGCGAGATTCTTGAAATATGGGAGACTGAAGAAAAAGATTTTGAGCTCGATTTAACATTTGAACAAAAATCAGAACCCGTGGCATTAGAAGGAAACAGCGAATATTGTTCCTCCGATATTCAGTCAGAGGATTGGAGTTCTAAATCAATGACAATTCCTATGACTGGAGGTGGCAAGAAGAAGATCTTGGAAAGGTTAGCTTCTGATGCTCAGAAGTTGATGAACCTTTTATTGGCAGTCCAAGATTTGAAGAAAACTGCACAAGGGTTGGAGAAATTCAAGACACCAAAGAATGATACTGAGTTAGATAGAGTGAAAGCAGAGATAGATGAACTGGAGGAAGCCATTATGCAGTTGTCTGGTACTAATGACAAGTTATCAAAGAGAGCTGAAACCAATTATGAATTGGAAGAGACCGAAAAGGCTCGAAAAAACAGGGTTTCGGAGAGGGCGAGGAGGTCATCGGAGAAGATTGGAAGATTGCAAATTGAAGTCCAGAGGATTCAGTTCGTATTGGCGCGACTTGACAACAAGTACAAGGGAAAGGATAGAAAAGTTTTGCTGAAAGACTATCTATATGGAAACTTGAGAAGCCCGCTAAATATGAAGCAGCGTAAAAAGCCTCAATTGTTTTCTTGTGTTAAACTTAAAACT

CTAAGGTCTGGGATCTGTCACATAGCATCATCACTCAAGGTTAACCAAGCGCACGAGTGTCAAGTTGAAACTGAAGAAGACAAACTTCTTGTTCAGCTGACATTGAGGAAGATTGAAGATCTGGATACTTCTTTGTGGTTAACCCAGGACGAGAAGTACCAGTTGCTATGTGAGAATTCAGTTTTGCTGACTCTGTTGGGTCAGCTAAGGTTAACTGCAGCAAGTATCAAGTCCGAGAGGATTGCTTTGGATAGAGATTTGAAGCTGAAGACAGAGGATCTAGTCCTTTTGCAAAGTGAGCGAAACATACTCAAGAGTGAAAGTGACCACTTAAACCAGCAGATTGGGGTTTGGGAGGAAACTCTGAGCAGGAAAGGGATGGAGCTGGTAGAAACAGAACGGAAACTCGAAGCTGTCATAAGTGAGAATGCAGAGCTATACACAAAAATGGAGAATAAGAATAGAGAGTGCGACGAAGCTAGGCTAAGAATAGCGGTCTTAGAGTTGCACAATTTGAGCAGTGGAGAAACCAACCTTCATCAACATGAGGAAATTGTTCGTCTTCGTGAAGTGGCTGGAAAGCTTGAGTTAGAGATGAGGAAATTACACGAAGAAATTCAGGAAGGAAGAGTTAGAGAAGATTATTTGAGCTCTGAGGTGCAACAGATAACTAGCGAGCTCAAACTCTGGGAGTATGAAGTTGAAGCACTATATTCTAATTGGCAGATTGCCGACATGCATGCAGTTTTGTTTGAAGAGAAGTTACGTGAGCTTGCTGGAGTATGTGTGAGCGTTGAAGACGAAAGAAATTTAAACATCGAGGCTATTGAACATATGAAAGAAAGAGTTCGGGTGCTGGAGTTGGAAAATAAAGGAATGAAATCTGAATTGGCTGCATATCTTCCTGCTGTTGCATCTCTAGATGATAGTGTAACATTACTTGAAGATCATGTACTTCTGGATAGAGATAGTTTTGCAGCTGATGATCAAGAAACAGAGGTTGCTAACTCATCAAGTAATCCCCATGATTACGCCAACAAAGGA------------------------------------------------------------------------------------------------------------------------------------------------------------------------------------------------------------------------------------------------------------------------------------------------------------------------------------------------------------------------------------------------------------------------------------------------------------------------------------------------------------------------------------------------------------------------------------------------------------------------------------------------------------------------------------------------------------------------------------------------------------------------------------------------------------------------------------------------------------------------------------------------------------------------------------------------------------------------------------------------

1019 CL1818.Contig1_R-bungei CL3964.Contig2_R-chinensis

AAAGAGGCAGAAACAGAGCAAATATATGCAGGTGAAAAGGTTGAAACTATAGAAGAGATGAAACAAAGTGATTTAGTTGATGAAATAAATGATCAAGATTTCATCAGTATCGGAGAAAACACTAGT---CTTGAACAGGAGGATTTCACAGAGGATATCAAGGAAACAAGACTAGAAAATGAGGGAGAAGAAAAACTCGCAAATCAAAATGTTGAAGTTGTGAAAATAGAAGAAACGGCCAAAAAACATGCAGGTATGATAAAGAAACCAAAGGCGGTACTAAAGAAAACAGCTATTCAGCCT---TCTTCACAAACTGATAAAAAGGTGGTGACGAAAGAGAAACCCAGCGGCTCTTTGCAGAATTCAAAATTGTCAAAATTACCCAGCTTCAACTCCAAAGTCGATCCTCAAAACGTGAATGGGGCAAAGAAAATACCAGAGCAGAGGCGTGGAATGAGTAATGCTACAAAGTTCACAGTTGTCAGAAATAGCACTAGCACCATCGAGAAGGCGCAAGCAAAGGATATACACAGTAAGAATACACAAGCAAAGGCTAAGGAGGGAGAGGAAACACAACCAAATAAGGTAAAGAAAAGTAATACTTTCAAGGCTTCACCATTACCCAGCTTTTATCTTAGGAAGGCTCCAACTACAAAACCTGAGCCAAAGAAGGATGGAGAGGAG---GCAAAGCCAGATAAGATTAAGAAAAGTAACACTTTCAAGGCTTCACCACTCCCGAGTTTTTATCATAGAAAGGATCCTCCTCTGATAGTGCCTGAGTCAAAGAAGATCACCCCAACACATCCTAAATCTTCAGAACTTGGACATCAAAGCAACTTGTTCGCTGAAGGGATAAAAATCGACACAGAGGACAGAAATAACAAGACTGCTTCAAGAACAATTAGCACCACTGCAAAAGAGACCATACACAAACTACTAGAAGGTGCTTGGGAGGATCCAAGCACTCAAAGAGCAAAG---------

------------------------------GATGAAAAGATTGAAACTCTAGATGAGATGAAACAAATTGATTTGGTTGATGAAATAAATGTTGAAGATTACATCAGTAACGAAGAAAACACTAGTCTTCTTGAACAGGAGCAATTCACAGAGGATATCAAGGAAACAGGACTAGAAAATGAGGTGCAAGAA---CTTGTGCATCAAAACATTGAAGTTGAGAAAGTAGAAGAAACTGCAAAAAAACATGCAGGTTTGATAAAGAAACCAAAGGCGGTAGTAAAGAAAACAGTTGCGCAGCCCTTTTCTTCAAAAACTGATAAAAAGGTGGTGGTGAGAGTGAAACCAAGCAGTTCTTTGCAAAATTCAAAATTGTCAAAATTACCCAGCTTCAACACCAAGGTTGATACTCAAAACGCGAATAGGGCAAAGAAAATACCAGAGTTGAAGCGTGTAATGAGTAACACTTCAAGACCCTCAGTTGTGCGAAATAGCACTAGCACCATCGAGAAGGCTCAAGCGAAGGACATACACAGTAAGAATACGCAGGCAAAGTCTAAGGAGGGAGAGGAGATACAGCCAGATAAGGTAAAGAAAAGTAATACTTTCAAGGCTTCGCCATTACCCAGCTTTTATCTTAGGAAGGATCCAACTACAAAACCTGAGGCAAAGAAGGATGGAGAGGAGGCGGCAAAGCTAGATAAGCTCAAGAAAAGTAATACCTTCAAGGCTTCACCACTCCCGAGATTTTATCATAGAAAGGATCCTCCTTCAGTAGTGGCTGAGTCGAAGAAGGTCACCACAACGCGATCTAATTCTTCAGAACTTGTACAGCAAAACAACTTGTTTGCTGAAGGGATCAAGATCGACAAAGAGGACAGAAATAACAAGGCTGCTTCAAGAACAATTAGCACGACAGCAAAAGAGACTATACACAAACTACTAGAAGGTGCTTGGGAGGATCCAAGCACTCAAAAGACGAAAGTGCCAAGT

1157 CL4380.Contig1_R-chinensis Unigene28957_R-bungei

---------------------------------------------------------------------------------------------------------------------------------------------------------------------------------------------------------------------------------------------------------------------------------------------------------------------------------------------------------------------------------------------------------------------------------------------------------------------------------------------------------------------------------------------------------------------------------------------------------------------------------------------------------------------------------------------------------------------------------------------------------------------------------------AATAATGACTTAATTGGTATTGTCATTCATTTGGAAGAGCTGCAGCAGAAAGTGTTTGCAAAGATGCAACAACTCGCTCTCGAGAAGAAGGATATGGAAGAACAAAGGGATACTGCTCAAGGAGTATTGAGTAGCATTGAGTCAGAAAATCTGTTAATAAAGAAGAGGTATGAAATGGATGTCAAAAGAATGGAAAACCAGCTAGAAACGTCTACTGGCCTTGTGGACAAGCTTCAGCTAGAACTTGAAACTATTGCCGATAAACTTAAGATCAGCTTGGAGTTGGAAGAAAGTTATGCAGATCGATTGGAAGAGCTATCATCTAAACTTTCAGTTTTCGAAGTTGAGATGCAGAATGTTACTAACGAGAACAGAGATCTTGCTAAAAAAATCACGGGTTTAGAGTTTATCAGTGAGGAGCTTGACAGGACCAAGTTAACTGTCATTGAATCAGCTCGTGAGAACCAAGCATTATTGCGGTCTTTTCAGTCTGGTAATGAAGAATCTGTCCTTTTATCAAATGAACTTGGCATTTTAAGTGAGAAGTTAAGGTGCATGACTGATGAAGTGAATTCTGAGAAAATTTTGAGAGTTAAACTGGAGGCCAACATTGCAGATCTTTCGTCTGAGCTGAAGATGAAAAATGACATTTTGATCTCCTGTGATGATGTGAAAACTGAGGTGGTCCATCTCAAGCAGACGGTGTCAGATTTAGAACTAGAGAAATCAAGAGTGTGCAATCTTTTATATGAAAGTGAAGAGTCCCTGAGAAAGGCTGATGAGGATGCTTCATTCTATCGTCTCCAGGTTACTGATCTGGAAGCTCATTTAAACGTCTCACACGAACATTTCCTATCCACAGATGTCGAACTCATTTGCATGAGAAATCAGTACGAGATTAGGATACATGAGCTTGTTCAGCAACTTGAAGCTATAGACGGGTGCTACAGGGAGCTTCATTTGACACATCTTCATGTATTAACGACACTGAATGGCCGTATTTCTAGTGAAGCACAACATGTTGATGAAAAAGCTCGGTTATTGACGGCCTTGGACTCTCTTAAGGCTGAGCTGGAAGTTTTTGCTAGTAATAACAGTGTATTGGTGAATAAA

TGTCAGAACCAAAATGCAGAGCTGAAGAAAGAACTCTTGAGCGTGAAAAAACTCTTAGAGGGTTCCCAAATATCTCTTCACGCGCAGGAGGGGCTTTATCAAAAAGCAGAAACAGAACTTAAGGAAATGCATTTAATAAACATAAATTTGAATGTTTTCTCAAAGGTACTGCAGGAATCTTTGGGTGAGGCAAGCAATGGCATTTGTATCATGAAGGAACAACTGGATGCACTTGGACTGAAGCTATCTCATTCAACTGAATCAGAGGAGTTGCTGAATCTCAGACTGCAAACCGCACTGGAGGATGTAAATACACTCAGGGTGTACAACAACAACTGGAGCACCAAATGTGATGAGCTAACATTGCAGAATCAAATTTTGGAGGAGAAAGTTAATAGCATGTCGAATGAAAGTGACTCTCTTACTCAAAAGATAACAAAAATGGGAAGGATGGCAGATGAATATAGAGGATTTGAAAGGAAATACAAAGACTGCACTGAAGAAAAAAATGAACTGCTAAATTTACTAAAGAAGGAAACGTCGAGAACATGTAGTTTTCAAGATGAAGTTAGCTCCTTGCACGAGGAGTTGAAAACAGTGAAGGCCGCTGCCGATGAGCAGTTCTCAGTCAACAGGAACCTGAAAGAAACCCTCTCCCTTCTGCAGGATAAGTTGGTGCATCTGCGGTCAGCAATGTTATTAATTGACGACCACATCAGGGAACAAAGTTCAAGTGTGACTTCTCTTCATGATACAGAAAATAAGGACTTAATTGGTATTGTCGTTCATTTGGAAGAGCTGCAGCAGATAGCGTGTGCAAAGATACAACAACTCACTCTCGAGAAGAAGGATATGGAAGAACAACGGGATACTGCTCAAGGAGTATTGACAGGCATTGAATCAGAAAATCTGTCAATAAAGAAGATGTGTGAAACAGATGTAAAAAGAATGGCAAGCCAGCTAGAAACGTCTACCGGCCTTGTGGTTAAGCTTCAGGTAGAACTTGAAACTATTGCTAATAAACTTAAGATCAGCTTGGAGTCAGAAGAAAATTATGCAGATCAATTGGAAGAGCTATCATCAAAACTTTCAGTTTTCGAAGTTGAGATGCAGAATGTTACTAACCAGAACAGAGATCTTGCTAAAAAAATCACGGGTTTAGAATGTATCAGCGAGGAGCTTGACAGGACCAAGATAACTGTCATTGAATCTGCGCGTGAGAACCAAGCGTTGTTGCTGTCATTTCAGTCTGGTAATGAAGAAACTGTTCTTCTATCAAATGAACTTGGCATTTTGAGTGAGAAGTTAAGGCACGTGACTGATGAAGTTAATTTTGAGAAGATCTTGAGAGTTGAACTGGAGGGCACCATTGCAGATCTTACGTCTGAGCTGAAGATGAAAAATGACATTCTGATCTCCTTGGATGATGTGAAAACTGAGGTGGTCCATCTCAAGCAGCTGGTGTCAGATTTAGAACTAGAGAAATCAAGAGTATGCAGTCTTTTATTAGAAAGTGAAGATTCCCTTAGAAAGGCTGATGAAGATGCTTCATTCTATCGTCTCCAAATTACTGATCTGGAAGCTCATTTAAGTGCCTCACAAGAAGATTTCTTATCCACAGATGTTGAACTCATTTGCACGAGAAATCAGTACCAGAGTAGGATACATGAGCTTGTTCAGCAACTTGAATCAATAGATGGGTGCTACAGGGAGCTTCATTTGAAACATCTTCGCGTATTAACGACACTGAATGGCCGTATTTCTAGTGAAGCACAATATGTTGATGAAAATGCTCGGTTACTGACGGCCTTAAACTCTCTTAAGGCTGAGTTGCAAGTTTTTGCTAGTGAT---------------------

3794 Unigene15743_R-chinensis Unigene7332_R-bungei

ATGAAGACTACTCTCCTAGTCTCCCTTGTTCTATTTCTCTTCCTCGCTCGAATCTCTGAAGGTTTACGCTTCGATCATAGTCTAAAAGTACTTCTTCATCATCAAAAATTCCATGAGAAGAAGAATAAAGTGATTGCAGACGACGCAGAAATCATATCCGAAGATGAGAACACTCTATCAGGA------AGGAAACTTATCACCAATACTCCCTCTACAACCTCTACAACTTCATCAAAGGTT------GAAGGGTCCAAAGTTGAACCAAAACCAAAACACCAACATGAAGAGAACTTCTCCATTAAATCATCACCATCTTCTTCTTCCTCTTCT---TCGGAGCAGCACGGGGAAGCCACAAACGGGAAGTACCCGGATATTATTGATATAGCTGGAATGGACTACTCTCCTGCAAGAAGAAAACCTCCAATTCACAAC

ATGAAGACTGCTCTACTAGTCACTCTTATTCTTTTTATCTCCTTTGCTTCGATCTCCGAAGGTTTACGCTTCGATCATGGTCTAAAAGGACTTCTT---CATCAAGATATCCATGAAAAGATGAATATAGTAACCGAAGACGACGCAGAAATCATATCCGAAGACAAGCATATTTCATCAGGAAGGAGTAGGAAACTTATCACCAACATTCCCTCTCCAACCTCTACAACTTCAAAGGTTGTCAACACTGAAGAGTCCAAAGTTGTACCCAAACCAAAACACCAACATGAAGAGAACTTTTCCGTTAAATCGTCACCAATTTCTTCTTCCTCCTCCCTTTCGGGGCACCGCGGAGAAGCCACCAACGGGAAGTACCCAGATATTATTGATATAGCTGGAATGGACTACTCTCCTGCAAGAAGAAAACCTCCAATTCACAAC

3400 Unigene12178_R-chinensis Unigene31395_R-bungei

---------------------------------------------------------------------------------------------------------------------------------------------------------------------------------------------------------------------------------------------------------------------------------------------------------------------------------------------------------GAACTGCTTATGTCACATGATGTTGCTGCAATCGATGAGTTGAAGGAGCAAATTTCACAGCTCTCTGTTTTATTTTCAAATGAAATTGTGGGCAGTCCTCTTTCAAAAGCAAAAATGGTTAATGATGGCTTTGTAGTTCATAGAGGAATTTCATCTCTAAAGACAGGACCTTCATCCTTTGAGCCAGTGGTTCATTTACTTGACTCTCCTGCTCTAGTTAAGAAGAAGAGTTTAGCCCCTGCAGTGATACAGTTACAAGACTCTCCTGTGGTTGTAAAAAGTACGGTAAAGGATCCGGTATTTGTTCTTTCTGATGATGAGGCAGAAGAGGAATTATCCTCTAAATTGACCAATCTATCCAGCAATGCAATAATTGATCTGATGGGTGAGAGAACCGTCAAACCGTCTGCAGATAAAGGTAGTCTAGTCAATGATATACCCATAAAAGAGTTAAAACAAGACACTGAAAATGAACCGTGGGAGGTGGCATTTAAAGCGGCAAGGCATTCACAGTCCTTGCAAAGAAATTCAGATCCATCATCTGATAATTTTGGACCTACCGGAGCTAGTGGCAGAGTGAATACTGTCGTAAAAGATTTAGAACAGGATATTGAAAATGAGCCATGGGAGGTGGCGCTCAAAGCTGCAAATCGTTCACAGTCCTTGCAAAAAATTTCAGAACCATCATCTGATAATTTTGGAGCTCCT------------------------------------------------------------------------------------------------------------

TCGTTAGAAAGATTCAGTCATTCACTGTCTGAACTTTCATGGCCTTCTTTAGTTAAATGTTTGGCAGAAGGGAAGCGGTTTATTAATAGCAAAAGCTCCCAGATGGCATGTGTCCGGTTACTTGAGATTCTCCCAATTGTTGCTGAGAGACTCTGCTCACCTTTGCATAATGTTCCTGGGACCGAAGTCAGCATTTTTGAATTGAAGTGGCTTCATGATTTTGTGGATTGGGGAAAATCATCGCAGGCCACGGTGTCCAGATACTGGAAGCAATCTTTTCTTCCATTGCTGGATGTACTACGAAGTTTGTGTCCTAATGATCCTGCAAGTACATTTGGGGCCATCGAACTGCTTATGTCGCGTGATGTTGCTGCAATAGATGAGTTGAAGGAGCAAATTTCACAGCTCTCTGTTTTATTTTCAAATGAAGTTGTGGGCAGTTCTCTTTCAAAAGCAAAAACGGTTTACGATGGCTTTGCAGTTCATAGCGGAATTTCATCTCCAAAGACAGGTCCTTCATCACTTGAGCCAGTGGTGCATTTACTCGACTCTCCTGCTCTAGTTAAGAAGAAGAGTTTAGCGCCTGCAGTGGCACAGTTACGAGACTTTCCTGCAGTTGTAAGAAGTACAAAAAAGGAACCGGTACTTGTTCTTTCAGATGATGAGGCAGAGGAGAAATTATCCTCAAAATTGATCAATCTATCCAGCAATGCGATAATTGATCTTATGAATGAGAGCACAGTCGAACCGGCTGCAGATAAAGGTAGTCAAGTGAATGATACACCCGCAAAAGAGTTAAAACCGGACATAGAAAATGAACCATGGGAGGTGGCACTTAAAGCTGCAAGGCATTCACAGTCCTTGCAAAAAAAATCAGAACCATCATCTGGAGCTGTCGGCGCTAGTAGAAATCTTGGAACGGCGAATGCTGTCTTAAAAGATTTAAAACAGGACATTGGAAATGAACCGTGGGAGGTGGCGCTTAAAGCTGCAAATCGTTCGCAGTCCTTGCAAAGAAAATCAGAACCATCTTCTGAAAATTTTAGAGCTTCTGATGGTAGTAGAAATCTTGGAATAACGAATACCGTCATCAAAGATTTAAAACATGACATCGAAGATGAACCGTGGGAGGTGGCACTTAAAGCCGCAAAGCATTCACAA

5791 Unigene11782_R-bungei Unigene2865_R-chinensis

GTTACCTATACCACAGTCATGAATGGTTTATGCAAGTTAAGACGGCTAGAGTCTGCAATTGAGATATGGCATATAATGGTGGGAAGAGGGTTTACTCCCGATGGTGTAGCATGTGGAGCTCTGGTTTTCAGTCTTTGTAAACAACAGAAGGTTGATTTGGCTTATGAACTAACAGTTGGATTAATGAAGCGCCGGGTTCTGCTGAGCAAATCAGTTTATAACGCTCTTATTAGTGGGTTTTGTCAAGCTAGTCGAGTAGACAAAGCGCAAGTCATAAAGTCTTTCATGCAGAAGAATGGAGTTGAGCCAGATTTGGTCACATACAATATACTCTTAAATTATTGCTGCGATGAGACTATGTTAGACGAGGCCACAAATTTGATGGAAAAGATGGAGAAACATGGAATGAATCTAGATAGTTACAGCTACAACCAACTTCTGAAGGGACTCTGCAAAGCCAACCGTATAAATAAAGCATACCTCTTGATGGTGGATAAGATGGAGATGAAAGGCCTCTGTGATGTTGTATCATTCAACACTCTCATTGTAGCACTCTGCAAGAGTAGATACACTAAAAAAGCGTAC

---------------------------------------------------------------------------ATGGTGCGAAGAGGGTTTACTCCTGATGGTGTAGCATGTGGAGCTCTGGTCTTCAGTCTTTGTAAGCAAAAGAAGGTTGATAAGGCTTATGAACTAATAGTTGAATTGATGAAACGCCGGATCTTGCTGAGCAAGTCAGTTTATAACGCTCTTATTAATGGGTTTTGTCAAGCTGGCCGAGTAAAAAAAGCGCGAGCTATAAAGTCTTTCATGGAGGGGAATGGAATTGAGCCAGATTTGATAACATATAATATATTCTTAAATTATTGCTGTGAAGAGACTATGTTAGACGAGGCAAAAACTCTGATGGAAAAGATGGAGAACCGTGGAATGAATCTAGATAGTTACAGCTACAACCAACTTCTGAAGGGACTCTGC------------------------------------------------------------------------------------------------------------------------------------

5232 Unigene25684_R-chinensis Unigene32170_R-bungei

AGTAAGGAGGAATTGTACATGTTTTGGAATCAGTTGAAGTTATCTAACAAACTCTATAATACAACATATAGATCCATGATAAGCTCTCTTCTAAATTTGGATGACATTACGGGAGCAGAGAAGATTCTGGAAGAGTGGGACTCTGAACACAAAACCTTTGACTTCCGTGTTCCACTATTGCTGCTTTCCTTTTATTGCAAATATGGTCATATGGGAAAAGCTGAATTGTTGGTAAGTAAAGCAATAGAGAAAAGGAAGAAGCCTTTTGCAGATACATGGGAGATCTTGGCAACTGGTTACATTAATAGTGATCAACTTCCAAAGGCGGTGCATGCTGTAAAACAAGGGTACTTGGCAAAACTACCACGGTGGAAACCAAAATATAAAACCCTAGCTACATGTTTGGGTTACCTGAAAGAGCAGGGAGATGCTAAAAATGCGGAGGAGTTTGTGAGATTGCTTGGGACCCCAGATTATATGTCAATAGAAGATTGTGAAGGACTGCTGGAGTATATATATAATCGTAAAGGAGAAGCAGGTTCCAGCAAAGTGAATAAGATTTATGAAGATGGTTCAGATGATGATGAAACAGAA------------

---------------------------------------------------------------------------------------------------------------------------------------------TCTGAACACAAAACCTTTGACTTCCATGTTCCAAAATTGCTGCTTGCTGTTTACTGCAAATGTGGTCATATGGGAAAAGCCGAAATCTTGGTAAACAGAGCAATGGAGAAAAGG---AAGCCCTTTGCAGATACATGGGAGATGTTGGCATCTGGTTATATTGAGAGTGATCAAATTCCAAAGGCGGTGGATTCTGTAAAACAAGGATATTTGGCAAAACGACCACAGTGGAAACCAAATCATAAGATCCTAGCTACGTGTTTGGGGTACTTGAAAGAGCAGGGAAACACTGAGAAGGCAGAGGAGTTTGTGAGATTGCTTGGGGCCCCTGATAATATGTCAATAGACGATTGTGAAGGACTGTTGGATTATATATATAATCGTAATGGAAAGGCAGATTCCAGCAAAGTGAACAAGATTGATGAAGATGGTTCAGATGATGGAACAGATGAACTTGCCCAAGAT

6080 CL7691.Contig1_R-bungei Unigene31022_R-chinensis

GATTTTAGTGAGAAAACGCTCACAAAGAAGATAATAGCATGCTTGACTGGAACAGAAAGCACGCTTGCAAATTTGGAACCTCTGAAAAATCACCTCAAACAGTTACTGAACGGAAAGAAGTTTTTACTAGTTCTAGACGATGTCTGGAATGAAAATCCTGAAATTTGGCAAAGGTTGAGGTCTTCGCTAAACGCTAACGGAAGTTCAGTTGTTGTCACGACTCGTCTACCCACTGTTGCAAAGATAATGGGTACACACTCTGTGTACGACTTAACAACTCTATCTGATGATGACTGCTGGGCACTGTTCAGAGGATGCGCGTTTGGAATGGACGACGAAGCTGTTGAAAATCTGGAAGCAATTGGCAAGCAAATTGTTAAGAAATGTGGAGGTGTACCTCTTGTAGCAAAAGCACTAGGCGGCTCGTTGCTTTTTGAACGCGGTGCAAATATGTGGAAGTCTGTTAGGGATAACGATATCTGGAAGCTAGAACGTGAAAAGGGTACTATTTTACCATGTCTGAGATTAAGCTATAACCGCTTACCGTCATATTTGAGAGAATGTTTTGTGTATTGTTGTGTATTCCCAAAAGATTATGTAATGGTCGTAGAAGAACTGATTCAGTTGTGGATGGCAAACGGGCTTCTTCAGTCAGATGGAAGAATGGAGTTGGAGGAAGTCGGTAACCGTATATTCAACCAACTAGTATTGCGATCCTTCCTCCAAGATGTCAGTGTAAATAGTGATGGGAACCAAGTGTGCAAAATGCACGACCTTATGCATGAACTCGCATGTTCTGTTGCACGAAAAGAATGCCATGCTTGGGAGGTGCACGAAGAATTAGTTACAATTCCCAAAAGCGTTCGGCATCTATGGATTGATCGGATTCCGTTCATACTTGTTAAGGCTCAACTTAAGTCACCCCTTCCCTTGCGTACATGTATCTTTTCTCGTGGTTCTTACATGTATGACCAT---CCTTCATTTGAAAAAACCTTAAAAAACCTCACTTGTTTGCGGGTCTTGCAGTTCCAAGCC---CCCCTAACCCTACCACAGTCAATAGAGAAGATGTTACACTTAAGGTATCTCAACCTTTCATCGTCCCTTATTGAACATTTACCCAAATCCATTTGTAGCCAAAAAAACTTGCAAACGTTAATTCTGAATTCCTGTCGTCACCTTAATAAATTGCCGGAATCTCTGAACAAGTTGATCAACCTGAGACACTTGGACTTGAGTAAGTGTGGGAGCTTAACGCAATTACCAGATGGGATTGGAGGTTTGATCAATCTT

------------------------------------------------------------------------------------------------------------------------------------------------------------------------------------------------------------------------------------------------------------------------------------------------------------------------------------------------------------------------------------------------------------------------------------------------------------------------------------------------------------------------------TTACCCAGTCTGAGATTGAGCTATAACCATTTGCCGTCTAATTTGAGAGAATGTTTTGTGTACTGCTGTGTATTTCCAAAAGATCATGAAATGGTGGTAGAAGAATTGATTCAATTATGGATGGCAAACGGGTTTCTTCAGTCAGATGGAAGAATAGGGTTGGAAGAAGTTGGGAACCATATATTCAACCAACTAGTATTGCGATCCTTTCTCCAAGATGTGAGAGATCATGATGATGGGACACATGGATGCAAAATGCACGACCTTATGCATGATCTTGCATGTTCTGTTGCTCGTAAAGAATGTGATGCTTTTGAGGAGGGTGACAAATTAGTTACTATTCCTAAAAGCATTCGGCATTTAAGCATT------CTTTTAGCTAAATCATGTGAGACTCTATGGAAGTCACCCCCACCCTTGCGTACATGTCTTCTTTTCTGTTGGGATTGGGAGGAGGAGTATTGTCCCCAAGTTGAAATAATTTTCAAAAACCTTACTTGTGTGCGAGCCCTTCGTCTAGAATCCCTCTCCTCATGTCTACCAGAGTCAATAGGGAAGATGATACACTTGAGGTATCTCAACCTCAGGAAGTCCCAGTTTGAGCATTTACCCAAATCCATTTGTAGCCTAAAAAACTTGCAAACTCTAGATTTGCACTTATGTGAGTATCTTCAAAAATTG------------------------------------------------------------------------------------------------------

6130 Unigene31399_R-chinensis Unigene38143_R-bungei

ATGCCAGAAGATTCACACTCAATGATTCCTCATGCCGAGCCTGAAGTAGGACCCGATTTATTTGCGTACTACACCCGTGAAATTGAGGAACTGTTCTCCCGAAAGGTAGATCGTACTCCTGTCTCGTCCAGACCTGCAACATCTACAGCAACATCAGCAATTGTTAAAGATAATGGAATTGGTGATAATAACTCCAAGGATCATGACATTTCTGGCAAATGTCATGCTAGAGAATTATTTTTTAATGGTGTAGGAGAGAGTTTATCTGCTTTGGAGGAAGAGAGAGTGAAGGAATCATTGAGGCAGAGCGTTAAGGCCTTTGCTAAAAAAACTGATGAGACACTAGCCTCTGTACTGGGTGTGATAAAAACGAAGAAACAGTTATATGCCAAAGAGTGTGTTACTAAAACACCCAGTCCAACAAATTCCATGTCAAAGCTGTCTCCCTGTAAGAAGCAGAAGATATCATCTTCTCTATCTTCAGCTGGTGTTGCCATACAATCTGGAACTCCTGAGACATTATCTACTGAAGGGGTGATAAAGAGTTTGCGGTCTCTTGTAGACGTTGACAAGTCAGTAATATCAGATGAACAATTACTGAAAAATGCTGATTTTTTACTCAAGACATCTTCTGACGAGTTACATGCAAAGATGGAGATTATGGAACAGCAGCTTGAAAATTATCTGAATGTAATTATGACAAATTGCAGAAAAATGACTGTTCAAGAAAAAACAGAACTTAGGAATCTCATTCAGAAGCTCCCATCGAAGAATCTTGATCGGGTGGTGGATATAATTTTACAGCACAGGAAATTACCGGAGAGAGAGTCTTGTAATATGCTCCATGTTGACTTGGAAAAGGAGGACGATGTTACCCTTTGGAGATTGTATTATTATGTTGAAGCTGTGGAAAGGGCTAAACACTTGGCTCAG

---------------------------------------------------------------------------------------------------------------------------------------------------------------GTTGTTGAGGATAATGGCATTGGTTATAATAACTCCAAGAGTAATGAAATTCCTGGCACATGTCTCGCTAGGAAATTATTTTGTAATGGTGTAGGAGACCGTCTATCTGTTTCTAACGAAGAGAGAGTGAAGGCGTCACTAAAGCAGAGCGTCAAGGCCTTTGGTCGAAAAACTGATGAGACATTAAAGTCTGTACTGGGCGTGATAAAGACGAAGAAACTGTTACATACGGCAGAGTGTGTTACTAGAACACCAAGCCCAACAAGTTCCATGTCAAATCAGTCTCCCTTTAAGAAGCAAAAGATATCATCTTCTCTATCTTCAGCTGGTGTCAGCACACAATCTGGAACTCCTGGGACATTATCCGGTGAAGAGGTGATAAAGAGTTTGCGATCTCTTGTAGACATTGACGAGTCAGTAATATCAAATGAAAAGTTGATGGAAAATGCTGATTTCTTGCTCAAGACATCTTCTGACGAGTTACATGCTAAGATGGAG------------------------------------------------------------------------------------------------------------------------------------------------------------------------------------------------------------------------------------------------------------------------------------

9481 Unigene12568_R-bungei Unigene40189_R-chinensis

ATGGGTCCAACGGTTCAAATTTATGGGTTTCCGGCTAAGGTGACAGCAGAAAATGTGAAGATATTTCTTGAAACTCATACAGGCGATAAAACTATTCGGGCACTTAGAATTTTGCAATCTCAAAAAAACCAGGGGTCAAATCCAAGAGCATATGCCATTGTCCAATACACGGATATAGATTATGTTACAGTTATATCAAATAAGATCAATGCCAATGAGAGATTATACTTTGGTAATTTTTATCTCAAAATTTACCCAGTGGAGCGTGATATTATACCAAAGCCAAATGTCTCG

ATGGGTCCGACAGTTCAAATTTATGGGTTTCCGACTAAGGTGACAGCAGAAGACGTGAAGACATTTCTTGAAACTCATACAGGCGACAAAACTATTCGCGCACTAAAAATTAAGCAATCTCAAAAA---CAGGGGTCAAATCCAAGAGCATATGCCATTGTCCAATACACAGATACGGATTGTGTTACAATTATATCAGATAAGATCAATGCGCGATTATGCTATGGCTATAGTAATTTTTTTCTCAAAATCTATCCAATGGAGCGTGATATTATACCAAAG------------

8049 Unigene28031_R-bungei Unigene37459_R-chinensis

ATGGACAAGTCTCACCAAACTCTGGCAGTGATGTTGGCAATAATGGTTCTTCTCTTTGGAATATCATCAGCAATTAGCCTTTGCAATTTGAGTCAAAATGAACTTGAGGCTTGCAAACCAGCTGTGGTCAAGTCCGGCGCAGTTGATCCTAGTGCTGAGTGTTGCACTGTGGCGGGTAGGGCAGATTGGTCTTGTTTGTGTTCGCATAGGAATAGTTTTTTGTTGCGCATGTTTGGGATAGACGCAGATCGTGTTCTGCAACTCCCTGTTAAGTGTGGCCTTTCTTCATCACCTCCAAATTGC

------------AGTAAAATTCTAGTTTTAGTGGTGGCAGTAATGGTTTTCTTATTTGAAGCATCATCAGCATTTAGCCTCTGTAATTTGAGTGAAAGTGAGCTCAATTCTTGCAAACCAGCTGTGGTCAGGTCTAGCACAGTTGATCCTAGTGCTGAGTGTTGTGCTGCAGCGGGTAAGGCAGACTGGCCTTGCTTGTGTTCAAAGAAGAATGATTTTTGGTTAGTTGTGTATGGGATAGACACAGGCCGTGTTCTGCAACTCCCAGTTAAGTGCGGGATTTCCTCAACACCTCCTAATTGC

2118 CL7254.Contig2_R-chinensis CL7965.Contig1_R-bungei

GATGGAGAGATCGATTTCTCGAACCAAGCTTTTTTCTCAAATACAGATATTGATGGACAATACCTTAATAACTGTTCTCTTGTTGATAGCTTCTTCAACGTGAATGACGCTCATGCATGTACTCATACCCACACCTGTAACCCACATGGTCCAGACCTTGCTCACTCACACACGTGTGTCCATGTTCACACTAAAATTCTCCCTGCTGTATCTGACAATAAAACAACTAGTGCTGATGACTCTGGCGAATCTGTTGATAAGAAATCAAAGAAACGGCCAGTTGGCAATAGGGAAGCAGTTCGTAAATATCGTGAGAAGAAGAAGGCACGAGCTGCTTCTTTAGAGGATGAAGTTATTAGATTGAGGACTCTCAATCAGCAGTTGGTGAAAAAGCTACAGGGTCAAGCTGCACTGGAGGCGGAGGTTGGTAGGCTGAAGTGTTTGCTTGTGGATATCAGGGGAAGGATTGAAGGGGAAATTGGATCGTTTCCTTATCAAAGGTCCGTAAAGCGTGCAGACGGGATCCCATCCCATCCGAACATACCTTGTGGTTATGCATTGAATTCTTGTGAGTTGCAGTGTGATGATCAAGTTTACTGCATGCAGCCTGGTTTGGAGAACAGAGGAGGAGAAACTGGTGCATTAGGCGGACAAGAAATTGGGGCATGTGATACTGGAAATTATCAATGTGTGGGAGGTTTTGACTCTGCGTTCACAAACCTCTCAGATTGTGGACGTGGAAATGTAGTGCCACCAGTTGGACCGCCTGCTCCTAATAAGAGGAAAGGAGGAAATCGTGCACAAGCTGCGAGT

GATGGAGAGATTGATTTCTCGAACCAAGACTTTTTCTCAAACACAGATATAGATGGACAATACCTTCATAATTGTCCTCTTGATGATAGCTTCTTCAACCTGAATGACGCTCATGCATGTACTCATACACACACCTGCAACCCAAATGGTCCAGACCTTTCCCACTCACACACGTGTGTCCATGTTCACACCAAAATTCTCCCTACTCTATCTGATGATAAAACAACTAGTAATGATGACTCTGGTGAATCTGTTGATAAGAAATCAAAGAAACGCCCAGTTGGCAATAGGGAAGCAGTTCGTAAATATCGTGAGAAGAAGAAGGCACGGGCTGCTTGTTTAGAGGATGAAGTTGTTAGATTGAGGACTCTCAATCAGCAGTTGGTGAGGAAACTACAGGGTCAAGGTGCACTGGAGGCAGAGGTTGCTAGGCTGAAGTGTTTGCTTGTGGATATCAGGGGAAGGATTGAAGGGGAAATTGGATCGTTTCCTTATCAAAAGTCTGTAAAGTGTGCAGATGGGATCCTGTCCCATCAGAACATGCCTTGTGGTTATGCAATGAATTCTTGTGAGTTGCAGTGTGATGAT------CACTGCACGCAGTCCGGTTTGGAGGTCAGA---GGAGAAATTGGTGCATTAGGCAGACAAGGACTTGGGGCTAGTGACACTAGGAATTATCAGTGTATGGGAAATCTTGGCTCTGTG---ACGAACCTCTCAGATTGTGGACGTGGAAATGTGCTGCCACCAGTTGAACCTACTGCTTCTAATAAGAGGAAAGGTGGAAGTCGTGCAAAAACTGCG---

6115 Unigene19495_R-bungei Unigene31310_R-chinensis

GCGGAATTAAACCCTAATGTTCAATCTGCTGTGGAT------GGAGTATTCTGCACTTCATCTTCTCCATCAATAGATCCATCTCAAGTTTTACTTCATAGAAATACAGGGTACATGGTTTCACTCGGAACATGCTCAAAGCTTTGTGCAGTGACCTTCATTACTGGTGTCATTGTAGGCTACACATTGAAGCGACGTGTTCGGAACTGGGCTTCCAAGATTCTCCGGCGACTAAGGGATGAT

GCGGAATTGAGGCGCAGTGTTGAATCTCTTGCTAAGGAGTCCAATAGTGTTGTGGATGCTTCATCCCCAATTGGAGATCCATCTGCACTTTTGCTTCGCAGAAATACCGGGTACATGGTTTCACTCGGAACATGCTCAAAGATTTGTTCAGTCAGTTTCATTGCTGGTGTCATTGTCGGTTACACATTGAAGCGGCGTGTTCGGAAATGGGCTTCCAAGATTCTCCGGCGGCTAAGGGATGAT

9467 Unigene40117_R-chinensis Unigene33607_R-bungei

AAAGAAAGATTCAGATTGTTTCCTCAGCAGGGTAATATTAAGAAATCTGACATGCCAACGGAAATAGGTTTGACAGAAGCTAGAGATGCCATAAGAAGAGAAGGAAAAGGAAATATTCAGTCTGTCCATCAATTGGGTCTGGACTTGAATCAGATGAATATATCTTCTTGCAGGTGCAGTGAGTATACCCAAAAGCAGCTCGAAAAGGAAAAATTCATATGGGTAGAGAACGATGATGGTAGCTTTATACCAACTAAACAACCGACAATGCGTCTAATGGGAAAAGATGTTACAGTGGGTAGAAGCAACAAAGAGATCACTGGCTCCGAGGATGGTAAGGTATGGACTGATAAGAATGTTATTAAAGAACACAGTCCAACAATGGCGGTAGAGAACTCGTCAGTGAGCAGAAATTTTCAAATGGAAAGAAGTGCACTGAAAGCAACAACAGTCCAGTCACAAGAATCTCAAGGAAATCCATACTTTCTGCAGAGAAACACT---GACCCTATGTATGCTCAAAGCTACTATAATTGGCAGCCAAATCTAATGCCACAATTGGCTAGTGACAGATACCCTTTCTCCTTCCCTTTTTCTCCTGATGAGTCGTTGACTAATACTTTCAGACTGCCAGAAACCAACATCCATGGAGTTGAATCTCATTGCCAATGCATGCCTTTGAGCTCTACCCAGCTCCTATATAACCAGGGTCTGTCTCGTGGTTCTTCAAGCTTTCAGTTTCCTTTCGCCACTCAGGTCTGTGTGGAGTGTTTCCCACCATCTCAGGTCCAAAGTTACCCAAATGTCCTACCTCATTGGATGCTGAATGGATCACAACAGCACGGAAGTCCAGTCATTAGTTCTCAATATGATGCAGATATTAATAGCAATCCACACAACTTCTCCAGATTTAGTTTTCCATCCATCTCTTCTCCTCCATATCAAGCACCCCTGAGTTCTCCGCCTAGTCAACAAGCCAGATTTGTACCTCGCCCACATGATTCATCTGGTCCTTATTCGGTTCAGCGTCCACTC---ATCCCAGTTCCTGCTGGCTTCAAAAACAACTCCTCAATCAATACAAGCTATAGAAACATGATCAATGCTAAGAACAAAATGAAGCCACTTGGAATCAAGAGAAACAGGAAAAGGTATGGCAAGCAACCAGATTCTTCTATTACATTCTCAAAGAAGCCCACTCTTGAAATACCGATG------------------------GAGAAAGAAGGGAGTTCAATGGAACATCAA------------------AGAGATGGGTCAGAAACATCGCCTGCGGTTTGCTTTTCTAACAGGGACACTTTGTCAAGATTAGGTCCTGTAAAGTTAAGTGCAGGAGCAAAACATATCTTAAATCCCAGCCTGAATACTGATCAGCACGGATCAAGGTCAATCTGGTCGAGTATACCTATTGACTCAGTGAGAAACTCTCGTGATGATGTCCAGAAGAAACCATTAAAGATATACAGG

---------------------------------------------------------------------------------------------------------AAAGGAAATATTGAGTCTGTTCATCAATTGGGTCTGGACTTGAATCAAATGAATATATCTTCTTGCAGGTGCAGTGAGTATACCCAAAAGCAGCTCGAAAAGGAAAAAGTTATATGGGTAGAGAATGACGATGGTAGCTTTATGCCAACTAAACAACCAACAATGCGGCTAATGGGAAAAGATGTTACAGTAGGTAGAAGCAACAAAGTGATCATTGGCTCCGAGGATGGTAAGGTATGGACTGATAAGAATGTTATCAAAGAACACAGTCCAACAGTGGTGGTTGAGAATTTATCAGTAAGCAGAAATTTTCTAATGGAAGGAAGTACACTGAAAGCAACCACGGCAAAGTCTCGAGAATCTCAAGGAAATCCATGCTTTTTGCAGAGAAACACTGTTGACCCTATGCATGCTCAAAGCTACCTTAATTGGCAGCCAAATCTAATGCCACAATTGGCTAGTGAGACATTCCCTTTCTCCTTCCCTCTTGCTCCTGATGGGTTGATGAATAATACTTTGAGACTGCCAGGAACCGATATCTATGGAGCAGAATCTCATTGTCCTTGTATGCCTTTGAGTTCTACCCAACTCCTATATAACCAGGATCTGTCTCGTGGTTGTTCAAGCTTTCAGTTTCCTTTCGGCAGTCAGGTCTGCGGGGAGTGTTTCCCACCATCTCAGGCCCAAAGTTACTCAAATGTCCTACCTCATTGGATGCTGAATGGATCACAACAACAAGGAACTCCCGTCATTAGTTCTCAATATGATGCAGATATTAATAGGAATCCACACAGCTTCTCCAGATTTGGTTTTCCATCTATCTCGTCTCCTCCGTATCAAACAGTATTGACTTCTCGGCCTAGTCAACGTGCCAGATTTCTACCTCGTGCACATGATTCATCTGGTTCGTATTCAGTTATGCGTCCACTCATCATCCCAGTTCCTCCGGGCTTCAAAAACAACTCCTCAATCAATGCGAGCTACAGAAGCATGATCAATCTTAAGAACAAACTGAAGCCACATGGAAGCAAGAGAAACAGGAAAAGGCACGGTGAGCAAACAGATTCTTCGACTACATTCTCAAAGAGGCCCACTCTTGAAATACCAATGGAACCAAGGGATGTAATAAGAGCGGAGAAACAAGGGAGTTCAATGGAACTTAATATACTCGAAGACACCGGAAGAGATGGGTCAGAAACCTCACCTGTGGTTTGCTCTTTTAACCGCGACACTTTGTCAAGATTAGGTCCTGTAAAGTTAAGTGCAGGAGCAAAACATATCTTAAAACCCAGTCTGAATAATGATCAGCACGAATCAAGGTCAGTTCGTTCAAGTATACCTAATGAATCAGTGAGAAGCCCTGGTAACGATGTCCAGAAGAGCCCCGTAAAGATATACAGG

11118 Unigene9468_R-chinensis CL6031.Contig2_R-bungei

---------------------------------------------------------------------------------------------------------------------AGAGCGTGGCAAAGGATTGAAGAACACATTGGAACTAAAACGGCTGTTCAGATCAGAAGTCACGCACAAAAGTTTTTCTCTAAGTTGGAAAAGGAGGCTCTCCTTAAAGGTATGCCGCTTGGACAAGCTTGTGAAATAGATATCCCTCCTCCCCGTCCTAAAAGAAAACCAAACAATCCATATCCTCGGAAGACCAGCTCAGGCTCTCCTAGCTCTGTACCTGTGGGACTGAAGGATGGGAGTTTTTTGATATCAACT---TCTTCACACTCCAGTAAACAGTCACAGGACCTAGAGAACGACTTACATCTTGAGAATCCTTGTAAGAAGACAAGTGGAAAAGATTTTTCTGACATGGACAATAATAGCTCAGGCATCCTTTCCCTTTCTGAAGAGACTTCGTGTACATCCTTATCACCATCAGTTAAAATTCCACCAAAACTTCCGGAACAGAAAGGGCAATCATTTTTCAGAGATTCTGTCTCCTCGGTGAAAGAAGAAAGTTGTGATGGTGGCAGGGCAGATAAATATCTTCTTGACACAGACGCCGAGGAAAGTCAACGAGTCGGTGTCACCTGTACAGACCTAGATGATGGAATGGGTGGAACCCCAATCCGGAATAACTCCCTC---AAATATTTGGAT---GAGGTGAAGACAGGAGATAGATTAGCGCAGCAAGACGATCTCTGTCAATCAAATCAGAATTATCCAAGGCATATACCCATCCATGTCTTGGAAAGCAGCACAGAAGCATGCACTGAGAGTCCGTCCCCCACCATGCCTCAACTCAAAGGTACTGAGACTTCAAAGCCATCTACGTGCGCCAATACTGGAAGTCACAGCAACATATCCTCTCAGCAG---------------CCATCCATCACCAACAATCAAGAAAGCTACAACATGTCAAATTTTTCGAATCTTATTGTAACTACTCTTCTGCAGAACCCAGCTGCCCATGCTGCAGCAAGCTTTGCAGCTTCTCTGTGGCCTTTTACGAATGTCAGAAGTACAACTGATGGTGCAGATCCGTGTACTGCAGCAATCGCTGCCGCTACTGTAATGGCAGCTTCTGCATGGTGGGCTGCTCATGGGTTGCTGCCTTTGTGCCCTCCTCTTCAATCTGCTGCTTTTGGGTGTGTCCCTCCACCGCTTGTCCCATGCACAACAGATCCCAGTCTTCCATGGGTTGGTCCAGGAGAACCCGGGAAAGTTATTGAACCAAAACTGTCGAATAATAAATCATCGGTTGTGACGCCATCTTACTGTAATAGTAAGAACGACAGTGCAGGGTCAGACAACGCTGAACCAACCAAATCCCACAGCCAAGAACAGAAACTTGTATCATCTACCAAGGGGAAAACCAAAGAACAGGTGGATCGTTCTTCGTGCGGATCCAACACGCCATCAGGTAGTGAGGCAGAGCTGGATGTGCACGCAGACGGAAAGGTTGAATCCAATGAGCCAAATAATAATGATAATGTAAATGAATGGATAGGTGAACCAAACAATTACCGCAGTAGTTGTAGAAGCACAGGTAGCAACATGAATGACTCTTGGAAGGAGGTCTCCCAAGAGGGGAAACTGGCCTTTAAAGCACTCTTCTCAAGACAGGTACTACCACAAAGTTTTTCACCACAAAAAATCTGCATAGCAAACAGGGAGGAGCAG------CAACCGAATGATAAATTACGGATAGATCTCAATAGCAAGACACCATTCACTTGTATAGATGATGATGATTGCGAAGAAAAC---------------------------ATTGAGAAACCTGAAGTTCGTGGAGCAGGATTTAAACCATACAAACGGTGCTCAACGGAGAGCTGTTCAGTGACTAACACTAGTATTCAAGGTGATGAAAGAGATCCAAAAAGGTTATGTTTACAAGGAAAAGGTTCAACC

TCTAAAGAAGATGACTCCATTACAAAGACACGGAAACCATACACGATTACAAAACAAAGAGAACGCTGGTCAATAGAGGAGCATAATAGGTTCCTAGAAGCTATTAAGCTATACGGCAGAGCATGGCAGAAGATAGAAGAACACATTGGGACAAAAACTGCTGTTCAGATCAGAAGTCACGCACAGAAATTTTTCTCAAAGTTGGAAAAGGAGGCTCTCCTTAAAGGTATGCCGCTTGGACAAGCTTGTGAAATAGATATCCCTCCTCCCCGTCCTAAAAGAAAACCAAACAATCCATATCCTCGGAAGACCAGCTCAGACTCTCCTGGCTCTGAATCTGTAGGACTGAAGGATGGGAGTTTTTCATTATCAACTTCTTCTTCACACTCCACTAAACATACTCAGGACCTGGAGAATGACTTGCATCTTGAGAAACCTTGTAAGAAAACAAGTGGAAAAGAATTTTTTGACTTGGACAGTAATAGCTCGGGCATCCTTAACCTTTCCCAAGAGACTTCATGTATATCCTTGTCTCCATCAATTAAA---AGTTCAAAAATTGTGGAACTCAAAGCCCAATCAATTTTCAGAGATTCTGCCCTCTTGGTGAAAGAAGAAAGTAGTGAAGGTGGTAGGGCAGATAAATACCTTCTTGACACGGATGCTGAGGAAACTCACAGAATCAATGCCATCTGTACAGACCTCGATGATGGATTGGGTGGAACCCCAATCTCAAGTAACTCCTCCCGCAAATATTTGGATGGGGAAGTGAATAGAGGAAATAGACTAAAGCAGCAAGAAGATCTCTGTCAATCAAATCAGAATTATCCAAGGCACGTACCCATCCATGTATTGGAAAGCAGCACAGAAGTG---------AGTCCCGCCCCCACCATTCATCAACTCGAAGGTACTGAGAATTCAGAGCCATCTACGTGTGTCATTACTGGAAGTCACAGCAACATATCCACTCAGCAGCCGTCGCCTCTTAATCCATCCATCACCAACAATCAAGAAACCTACAACTTGTCAAATTTTTCAAATCTTATTATATCTGCTCTTCTGCAGAACCCAGCTGCCCATGCTGCAGCAAGTTTTGCAGCTTCTTTGTGGCCTTTTACGAATGTCAAAACTACAACTGAAGGTGCAGACCCGAGTACATCAGCAATTGCTGCAGCTACAGTAATGGCAGCTTCTGCATGGTGGGCTGCTCATGGGTTGCTGCCTTTGTGCCCTCCTCTTCAGTCTCCTGCTTTTACTTGCGTCCCTCCACCGCTTGTCCCATGCCCAACAGATCCCAGTCTCCCATGGGTTGATCCAGGAGAACCTGCGAAAGTTATTGAACCAAAACTCTCGGATAATAAATCGTCGGTTGTGTCGTTATCTTACGATAATAACAAGGATGATAGTGCAGGGTCAGACAACGCTGAACCTAACAAACCCCACAATCACGAACAAAAACTTTTATTATCTACCAAGGGGAAAACCAAAGAACAAGTGGATCGTTCTTCGTGTGGATCCAACACGCCATCAGGTAGTGAGGCAGAGATGGATATGCGTGCAGACGAGAAGGATGAATCCAAAGAGCCTAATGATAAT---AATGTAAACGAATGGATAGGTGAACCAAACAATTACTTCAGTAGTTGTAGAAGCACAGGAGGCAACATAACCGAGTCTTGGAAGGAGGTCTCACAAGAGGGGAAACTGGCCTTTAAAGCGCTCTTCTCAAGACAAGTACTACCACAAAGCTTTTCACCACCGATGAGCTGCATAGACAACAGGGAGCAGCAGCTGATGAGTGACACTGATAAATTACGGATAGATCTCAATAGCAAGACACCATTGACTTGTATAGATGATGATGATCGCAGAGAGAGCATTGAGAAAGTTGAATTGATGATGGGCATTGAGAAACCTAAGGCTCGTCGAGCAGGATTTAAACCATACAAACGGTGCGCAATGGAGAGCAGCTCAGTGACTAACACTAGTAATCAAGGTGAAGAAAGAGATCCAAAAAGGTTATGTTTACAAGGA------------

9604 Unigene40986_R-chinensis Unigene31731_R-bungei

------GCGTTGATAAAAAGCTTGTCATCCTCA---------GTAGCATCGGAAGCAATTCCATCAGTGTTGGATTGTATTCTGAGTTGTTCAACCGCTCTCGATCCCCGCTCTCTCTTTGATTCCTTGCTCCTAGCTTTCTCCAACTTGCTTCTGCTGCTCTTACAGGCTGGGGATTGCTTGCAATCA---ACTACATCTATATCATCTTTCTCAACCGCTCTCTGTTACCTCTTAAGCAAATCAGGAACTGATCACAATGCTTTGCAAGCATTTATTTGGGAAGCTTATATTCCAACAATGAGGATTATAAATGTGAATCACCACCAATTACTTAACCTGATGGCACAACAACTATGCGATGTTGTAGTCGAATCTAATACATGGAAGGTGCTAGATGCTAGCTTGGTGCCTTTTTGTCTACGATCCATTGGGATATCTATAGGAATGCGTCAGAATGAGGAATCATCTGTTTACCAATGGAGTGAC---------ATTCACAAGCAAAGTGGTTTTCTAGATGTTCCCTTTGCAAATAAACAACCCATCTCACCTTTGATCGACTCTTTACCACTGCCTGTATCCTGCCATATATTAACTTCTTTCCTACTTACTTCATTAACAAGTTCTCAAGCTCAGCATAATCCACCAGAAACAATGTCGCTAAATGGGCCATCTGCACATGTTTTTTCTAGAAATCTACTGTGGGATATCTCTGCCATGGCCATACAGATGCTTTCACTAACTCCAGATCACAGGTCATGTGCAACTCATCTTCTC------------------------

GTTTCATCGCTGGTAAAAAGCTTGTCATCATCGCTGGTAGTAGTATCACCAGCAGCGATTCCATCAATTTTGGAATGTATTTTGACTTCAATCCAATCTCTCTCTTCTACTTCCCTCTTTCATTCACTGCTCCAAGCTTTCTCCAATCTCCTCTCTTCAATG------------GATTGCGGGCAATCAAATACTAGCTCCATATCATCTTTCACAACTGCACTTTGTTACCTCTTAAAGAAATCAGGAACTGATCACAATGCTTTGCAAGCATTTATTTGGGAAGGTTATCTTCCAACAATGAAGATTATAAATGTGAATCACCACGAACTACTTAACCTGATGGCAGAACAACTATCCGATGTTGTAACCGAATCTAATACATGGAAAGTGCTAGAAGCTAGCTTGGTGCCTTTTTGTCTCCGGTCCATTGGTATATCTATAGGAATGCGTCAGAATGAAGAATCATCTCGTTATCAATGGAGTGAACAGTCAGTCATGCACGCGCAAAGTAGTCTTCTAAATGATTCCGTTGTAGATAAACAACCCATCCCA------CTCGAGTCTTTACCACTGGCCATATCCTGCCATATTTTAACTTCTTTCCTCCTTACTTCACTAAGAAGCTCTCAAGCTCAATATAATCCACCAGAAAGAATGTTGCTAAAAGGGTCATCTGCAGATGTTTTTTCTAGAAATTTACTGTGGGATATCTGTATCATGGCCATACAGATGCTTTCACAAAGTCCAGATCACAGGTCATGTGCAACTCACCTTCTCCTTCCACATATTTTGAGGATATTT

9877 Unigene40010_R-bungei Unigene44923_R-chinensis

AAAATTATCAATCCTAACATTTCTTTGGTAGAATTGATGGAGAAACTGTGTCAGTACTTCTTGCAACTAACTTACAATCCTACTATGAATGATATCAACATCACTCCCATTGGGCGTTACATGAAGAATTCCCGTGCTACTGCTTCTGATGGCAGTGATTGTGGTCATAATCACATGGCAAGTAGTTCTTCTAATGGGCAGCAGAGTACTGTGGTGAATACTCAGTCCCATAATGTTGAAGGGAAGAGACAACCTCACAATCTGAATGACATTTCTAACGGCCAAGAAAGAGTAAGAATCCCTGTGGTCAGTAAAATTAACAGTGAACAGTATCCTCCTTGTTTCCGATACATTTCGCGAAATGCACATTATCAGGGGGCTCATGTAAATTTCTCACTTGCTCGTATTGGGGATGAAGATAGTTGCGCTGAATGTTTTGGTGATTGCTTGACAGCACCAATACCTTGTGCATGCACAAAAGAGACAGGAGGTGTGTTTGCTTACACTGTTGAAGGGAATGTCAAGAAAGAATTATTAGACAAGTGTATTTCGTTGAAACGTAATCCGAAAACTCACTATTGTGAAGATTGCCCTTTAGAAAGATCGAAAAGTGGCTGGGGGAAATGTAAGGGC

---------XXCCCGAACGTTTCCTTGTTAGAATTGATGGAGAAACTGTGTCAGTACTTTTTGCAACTAAATTCCAATCCTACTATTAGTGACATCAACGTCACTCCGGTTGGGCGGTACTTGAAGAACTCCTGTTCTATTGCGTCTGAAGGCAGTGATTATGGTAATAATCACATGGCAAGTAGTTCTTCAAATGGGCAGCAGAATACTGAGATGCATGGTCCTCCCCATAATGTTGACAGAAAAAGACGACCTCTCGATCTGAATGACATTTCTAAAGGCGAAGAAAGAGTAAGAATCCCC------------------------------------------------------------------------------------------------------------------------------------------------------------------------------------------------------------------------------------------------------------------------------------------------------------------------------------------

2652 CL9008.Contig2_R-chinensis Unigene6848_R-bungei

GATATCATCTTCTTAATCCTGATTTCTCTCCCTGCAGTGGAAGCTTACAAGTTTAGACAAGTTTGCAAATCATGGTATTACATGATCAAGTCGCCTGCTTTCGTACAGGCCCATCTTCGTAAGGCAAAGCATGGGTTAATCTGTAACAAAGAAAATGGGATGGACAATACTGTCTAC---AATGACAAGATCCAGTTACGGGAATTTGACGAAAAATCTAGGAATATTTGGGGCAGTTGTAATGGTCTAGTATTACTTGATCAGATTGTTCAAACGGCTAGGAGACGCTTTTTGTTTATTGCAAATCCTGTTACTCAAGAGGTGATCATAAAACTTCCTTCTCCATATAGCCACAACGTTAAAGCATTTTCTCATGTGTTTGGGATCACATACGTGGCTTCAGTTAAAAGGTATGTCGTGGTGCATTACTTCATTATGCAAAAGGGAAATCAACAATTAGGTAATTTGACATCAGATTTTGGATGTGAAACACTAAGTGTTGGTGATGATGAATGGGAACATGTGTTTTACTTTCGTACAAATACAGGGATGGAATTCTTTTGGTCTGCAAAAGTTATTTTCATCAATGATGTGGCATATATGGTTGTTTTGGAGTGGATAGACTCATATAGGTCCCATCTCATATCTTTGAATATGATTGACAGGAAAGTATATAAAACGAGTATGCCTGATAATCATAAAGATCTATTTGAAATAGGAGGTGTATTATCTTATACACGTCTATGTAATGATGCTGATTTTGAGATATGGAGTTTGGGAGATTTTCGTACTGGGGACTGGGTTATGCATACAAGGATTGAAACTAGAGTAAATGGTTTTGCTGGTTCTGTAGTTGCAACTCTTGAAAATGGAAGAATAATAATTGGCACGTACTATGATTTTGCTGCGACACGAAAGGATGATGTTGGCTACTTTGCTTATGACATTCAACTCGAGCAAACAACCTATTTAGATTTTAAACCACAGCAAACAGGATGTAGGTTGCTACATCATGTCGACAGTCTTGTTTCGAGCATAGAAGTA

---------------------------------------------------------------------------------------------------------------------------------------------------------------ATTGACAATATGGTATACAATAATGACAAGATCTCCTTACGTGAATTCGACGAAATGTCGAAGACTATTTGGAGTAGTTGTAATGGTTTAGTATTACTTGATCAGATTGTTCAAACCGTTAGGAAACGCTATTTGTTTATTGCAAATCCCGTTACTCAAGAGGTGATCACGAGACTTCCTCCTCCGTATCGCCACAACGATACAAAATTTTCTCATGTGTTTGGGATGACATATGTGGCTTCAATTAGAAAGTATGCCGTGGTGCATTACTTCATTCTAGAAGAGGGAAGTGAACAGTTAGGTAATTTGTCGTCAATTTTCGGATGTGAAACACTAAGTGTTGGCGACGATGAATGGAAACGTGTGTATTCTGTTCATACAAATACAGGGATGCAGTTCTTTTGGTCTGGAAAAGTTATATTCATCAATGATGTGGCACACATGGTGGTTTTGGAGCAGATAGACTCATTTTTGTCCTATCTTATTTCTTTGAATATGATTGACATGAAATATTACAGAAGGAGGATGCCTGATAATCATAAAGATCTTTTCGAAATAGGAGGCCTATTATCTTATGCGCGTCTATGTGATGATACTGATTTTGAGATATGGAGTTTGGAAAATTTTCGTACGGGGGACTGGGTTATGCATACAAGGATCGAAACTGGAGTAAATGGTTTTGCCGGTTCCGTAGTTGCAACTCTTGAAAATGGAAAAATAATAATTGCCACGTACTATGATTTTACCGCGACACGAAAGGATGATGTTGGCTACTTTGCTTATGACCTTCAACTCACGCGAACAACCTGTTTAGATTTTAGACCACAGCAAGCGGGATGTAGATTGCTACCTCATGTCGACAGTCTTGTTTCG------------

7885 Unigene37240_R-chinensis Unigene28117_R-bungei

ATGGTGTTCTCCACAATCCTCCGCAGAACAGCCTCATCTCTCCTCCCCCGTACCATCACCTCCGTTACACAAAGAACCTATCACTATGCTGCAATTACTACCCTGAAAAACAATTTT---TCCTTTGAGGAATTTCGTCAAACACCTTCTTGCAATCACAGTAATAATTGCAATTGCAAATACTGTCTCCCAGTTCGTCATTTCTCTTCTGTTCCTGAGAAAGCAAATAATGATCAGAAACTTCTTTCAACTCTTGAATCTGAGATTGATGATGCTGTGGAGTCTGGCGATCATGAACATGAGATCAACATTCCAGAGGATTTTCCATTCAAGATTGAAGATGAACCTGGAAAGAGCTATATATCACTCAAAAGACAATACCAGGGAGAAATCATCAGGATAGAAGTGCAAATGCCAACACTAGCTGAAGATGAGGACAATCGAGATGTAGATGAGGACAATCAAGATGAAGGCAAGGATGGCGAAGGCGAGGGTGATGAAGATAGTGATTCTCCATCGAGCCTTCCAATAGTTGTGAGTGTTTCTAAAGGGAAGAATGAACCGCACATGGAGTTCTGCTGTACTGCTTATTCTGATGAGATTGTAATCGATAGCTTGTTTGTGAAGAATCCAGATTCTTCTGACGAGGGATTTGATTACGAGGGACCTGATTTTTCGGATTTGAACGAGGAGTTGCAGGAGTCTTTTAATGTGTATTTGCAAGACCGAGGGATTAAACTAAGCACCACCAACTTCTTGTTTGAGTACATGTTCAACAAAGAGCAAAAAGAGTACTTTAGATGGTTGAAGAATCTGAAGAGTTTCGTAAAG

ATGGCATTCTCCGCAATCCTCCGCAGAGCGGCCTCATCTCTACTTTCCCGTACCATCAACGCCACCGGACAAAGAACCTACTATGCTGCTGCAATTACTGCCTTGAAGAACAATATTTCTTCCTCTGAGGAATTTCGTCAAAGACCTTGTTGCAATCACAGT---AATTGCAATTGCAAGATTTGTCTCCCAGTTCGTCATTTCTCTTCTACTTCTAAGAAATCTAATAGTGATCAGAATCTTCTTGCGATTGTTAAATCTGAGATAGATGATGCTAAGGAATCTGGAGATCATGAACAGGAGATCAAAATTCCAGAGGGATTTCCATTCAAAATTGAAGATGAACCTGGAAAGAGTTCTATATCACTCGAAAGACATCACCAAGGCGAAATCATCAGGGTAGAAGTGCAAATGCCAACACTAGCTGAAGATGAGGATGACCAAGATGAAGACAAGGACGACCAAGATGAAGAGAAA---------------GGCGATGAAGAAAGTGATCCTCCATCGAGCCTTGCAATGGTTGTGAGTGTTTCTAAAGGGAAGAATGAACCATGCCTGGAGTTTTGCTGTACTGCTTATTCTGACGAGATCGTAATTGATAGCTTGTTTGTGAAGAATCCGGATTCTTCTGAGGATGGTTTTGATTACGAGGGGCCTGATTTTTCGGATTTGGACGAAGAGTTGCAGGAGGCTTTTAATGTGTATTTACAAGACCGAGGGATTAAACCAAGCACCACCAACTTCTTGTTTGAGTACATGTTCAACAAAGAGCACAAAGAGTACTTGAGATGGTTGAAGAATGTGAAGAGTTTCGTAAAG

1769 CL6188.Contig1_R-chinensis Unigene16921_R-bungei

------------------------------------------------------------------------GATAAAATGGAAGTAAATTCCCAAATCGAAGTTGCACTCTTATCAGAAGAGGATTCATGGATCCTCTTTCGCCAGAAAGCTGACACCGTGGCAGACTTACCACTTGCTCAAGAGCTTTTAAATGAATGCAAGTGCTTGCCGTTGGCAATCATTACGCTGGGATTGGCTTTGAGAAACAAGAATGAACGTGTTTGTGTTGTTGCACTTGCACAATTGCGAAATTCCATCTTCAAAGGTATGAGCCCCGTTGTTCCTTCAATAAAGTTGAGCTATAAATTCTTAGAGAGTGAGTCAATCAGAATTTGTTTTTTATTTTGTTGTTTATTTCCGGAG------------------------------------------------------------------------------------------------------------------------------------------------------------------------------------------------------------------------------------------------------------

GTTGGGATACCTTACAAAAACAAAGGCAAATGCTGCAAGGTTGTATTCACTACAAGAGAACAAGATGTTTGTGGTGGAATGCAAGCAAATGCCCAAATCAAAGTTGCCCTCTTATCAGAAGAGGATTCATGGGAACTGTTTTGCCAGAAATGTGGCACTGTGGCGAAATTACCAATTGCTCGAGAGCTTTTAAATGAATGCGACCGCTTGCCCTTGGCAATCATCACGTTGGGATCAGCTTTGTGTGACAAGGATGAAGAAGTCTGTGCTAATGCACTTCGGCAATTGCAGAAGTCAATTTATGAAAAAATGAGTCCCGTAGATTCCTCTATAAAGTTGAGCTATGATTTTTTGACAAAGGATCAG---AAGGTATGTTTTTTGTTTTGTTGCTTATTTCCCGAGGACCATGTAATTGAATTAGATGTATTATTAAGTTATGTGATGGGAGAGAAGCTACTTGAAGATGTGGACACATATGAAGATGAAAGGGGTCAGTTGTACACTATCCTAGACAAACTTGTATCTTCTGGTTTGCTGTTGAGAGACGAGGACGGAGACATAAGAATGCACGATGTGGTTCGTGACATGGCTATCTCAATTTCAAAGGAAGAGGAAGGGCATATTGTGAAAGCTGGAAGGAATTGGAGTTACTGG

4492 Unigene19440_R-chinensis Unigene12552_R-bungei

------------------------------------------------------------------------------------------------------------------------------------------------------------------------------------------------------------------------------------------------------------------------------------------------------------------------------------------------------------------------------------------------------------------------------------------------------------------------------------------------------------AAAAAGAAGAAGAAGAAGAGTAGGGAGAAGAGTGAGAAGAAAAAAGGACATAAGAAGCATAGTCGTACACATGTGGAGGATCAAAGCTCCGAGGAGGAGGAGGATGAATGTAAGAGAGAATCTTCAGGGAAGAAGAGTCATTCACGCAGTAGGGATATACGTGATCAGTATTTTTCTGACGATAGGACAGACGACTCCGAAACTGAACAGAGGAAAAGGAGGAGAGATGACAAGTACACTTCGTCCAAGGGCCGAGTATCAGAAAGGCATGTCAGTAACAAGGAGAAAGAAAGAAGCAGGAGAAGCCACCACAGTAAT---AGTGAGAAGCTTGACCGGCATTCCAGAGATATATCTGATTCAGAAACACGAGAAAAGAGAGAGGATAGATATGACAAGTATTCATCATCTAGAGATCTAGGATCAAAAAGATATGTCAGTAGCAGGGACGAAGAACGAAGTAGGAGATCTCGACATGACAATTATGATAAGCGTGGAAGATATTCATCTGATGACAGGTCTGATTCAGAAAATGAAGAGAAGAGAGATAGGCAGTACGAGAGGTACTCTTTATCCAAGGAGCATGGATCAGAAAGATATGCTACTAGGAATGACAGAGAAAGAAACGAGAGAACTCACCACCACAGTGATAGAAAGCTCGAGGTGTATTCATCTAATGGCAGGCCAAAATCTGATGTTGAAGATGACAGAACGAGGATCAATGAGAAGTCAATAGATGAGAGGCATTCACCCAAGTTAATGGAAGCTGCAAGCAAAAACATGGAAAGACCACACCAGAAGCAGCGGCGAGTTGTCACGAAGCTTTCTGAGGAGGAGAAAGCTGCTAAGTTAAAGGAGATGCAAATGGATGCTGTGTTGCATGAAGAACAGAGGTGGAGTCGGTTGAAAAAGGCTTCAGAGAATGATGCTCGAGAGGCTAGTCAGGCCACCTCTTCGCGTGGTCCTAACTTTTTAACTGCTGCTCAA---------------------------------------------------------------------------------------------------------------------

ATGGCGCTTAAGTTTTTGAATAAGAAGGGATGGCATACAGGAAGTTTAAGGAACATAGAGAATGTGTGGAAGGCAGAGCAGAAACACAATCAAGAGCAAGTGAAATTAGAGGAGTTGCGAAAGCAGATAGCAGAAGAGAGAGAGAAAGCGGAGTTTCGATTACTTCAAGAACAAGCTGGACTTGTTCCGAGACAAGAAAGATTGGATTTTTTGTATGATTCGGGATTGGCTTGTGGGAAAGGGAGTTCTGATGGGTTTAAGGCGCTTGAGCAAGTGCCGAAAGTGGAGCCGGAGGTGGCTCCGTCTTCTTCTTCGGCTAAACCGCAGTCGGTTACGCCTGGTGCGTTGTTTGTGGATAAGCCTCAATCGGCTAATGATACGTGGAGGAAACTGCATTCTGATCCTTTACTTATGATTCGGCAACGAGAGCAGGATGCGCTTGCGAGGATTAAGAATAATCCGGTTCAGATGGCGAATATAAGGAAATCTGTTGAAGAGAAGAAAAAGAAGCATAAGGAGAAGAGTGAGAAGAAAAAGGGACACAAGAAGCGTAGTCATAAACATGTGCACGATCAAAGTTCC---GAGGAAAATGATGAAAGTAAGAGAGAA---ACTGGAAAGAAGAGTCATTCCCGCAGTAGGGATACACGTGATAAGTCTTCTTCTGATGATAGTACA---GACTCCGAAACTGAACAGAGGAAAAGGAGGAGGGATGACAAGTACTGTTCATCCAAGGGTCAAGGATCCGAAAGACATGTTGATGGTAAGGAGGAAGAAAGAAGCGGGAGAAGTCACCACCACAATAGTAGTGAGAAGCTTGATCGGCATTCCGATGACAGATCTGATTCAGAAAAACGAGAAAAGAGAAGGATTAGACACGACAAGTATTCCTCTTCCAAAGATCAAGGATCAAAACACTATGTCAGTAGCAGGGACAAAGAACGAAGTAGGAGAACTCGACACGAAAATTATGATAAGCGTGGAAGGTATTCATCTGATGAGAGGTCTGATTCAGAAAATGAAGAGAAGAGAGAGAGGAGGTACGAAAGGCACTCTTCGTCCAAGGGACATGGATCAGAAAAATATCCTACTAGGATTGATGAAGAAAGAAATGATAGAAGTCACCATCACAGTGATAAAAAGCTCAAGGCATATTCATCTAGTGGAAGGTCAAACTCAGACGTTGAAGCTGACAGAACGAGAATCCATGAGAAACCAACAGATGAGAGGCATTCACCCAAGTTAATGGAAGCACCAAGCAAAAACATTGAAAGGCCATACCAAAAGCAGCGACGAGTTGTCCCAAAGCTTTCTGAGGAGGAGAAAGCTGCTAAGTTAAAGGAGATGCAAATGGATGCTGTGTTGCATGAAGAACAGAGGTGGAGTCGGCTGAAAAAGGCATCGGAGAGTGAGGATCGAGAAGCTACTCTGGCCAACTCTTTGCGTGGTCCTAACTTTTTGAATGCTGCTCAGAAGAGTGTATACGGTGCCGAAAAGGGTGGAAGTTCAACAATTGAGGAGAGTCTTCGTCGTAGAAAATATTACTCTCAAGGACGATCAGAAGCCAGTGAAGTCAATGCGTTTCGGCGA

3637 Unigene29133_R-bungei Unigene14507_R-chinensis

ATGGATCACATCAAG---GGAGAAAAGCTTCAGTCCATGAAGAAGAACAAGAGAAACCAGTTTGTTGACAACCTCATCCTTTTCTCCTTTATAACACTATCATGTATCTTGTTTTGTTCTAGCCCTCTCTGGATGCCTCCTATATACTCTTTCATGAAAACCTTACTTTTTGTCTATCTTCCATACATAAAAACTTACATATCTAGTCCCAAATGTTTGTTTATACTCTGCAACATCATTGTTGTGATCCTCGTTGGAGAGTCCAAGCTAGTGGGTTCAAAATCATCAGCTCCAGCAACAACTGATATTTACAATGAATACATAAATAGGAGTGAAATTGTTCGAAAAGCCCGATTTCTTGAGGAGAAA------GAAAATGAAATGGAAGTTGATGTATATGAGATCGAAGAAAGTGTGCAGGGAGATGATGAAATGGTGGAAAAAACAGTTGAAGGAGAAAAGGAGTCTGATGTAGAAGAAGATAGGCTTTGTTTGCCTACTGAGGAACTGAACAAAAGGGTTGAAGATTTCATTGCCAGGGTGAACAAACAAAGGATGCTTGAAGCTAGATCACTC

ATGGATTACATCAAGGAAGGAGAGAAGCTTCAGTCTATGAAGAAGCAAAAGAGAAACCAGTTTGTTGATAACCTCATCCTTTCTTCCTTCATAGCATTATCATGTATCTTGTTTTGCTCTAGCCCTCTTTGGTTGCCTCCTATATACTCTTTCATGAAAACCCTAGTTTTTGTCTATCTCCCATCCATAAAAACTTACATCTCTAGTCCCAAATGTTTGTTTATACTCTGCAACATCATTGTTGTGATCCTCATTGGAGAGTCCAAGCTATTGAGTTCAAACTCATCAGCTCCAGCAACAACTGATATTTACAATGAGTATGTAAATAGAAGTGAAACTGTTCAAAAAGCCCGGTTTCTTAAAGAGAAGAAGATAGAAAATGAAATGAAGATTGATGCATTTGAGATGGAAGAAAATGTGCAGAATGAAGATGAAATGGAGGAAAAAACAGTTGAA---------------------------------------------------------------------------------------------------------------------------

4334 Unigene28390_R-bungei Unigene18601_R-chinensis

ATGGGAAATTGTTGTGGGTGCGAAGAATCATCATCGGCTATATGGGGTGGTGAAGATTGGAGCTCAGTTATATCCAAGAAGCCGGCCAAGAGAGAT------ATCTTGCACAAGAAGGGAAGAAATGAGAATCCTGATATGACGAAGTCTCCATCAGGGAACACCAGAACCACGGAATCTTCAACTTCAATTAAAATCAGGATCACAAAGAAGGAGTTAGAAGATTTACTCCAGAAAGTAGAGATACAAGGTATTTCATTCGAACAAGCCTTGACTCAATCCATGGATGTGAAAGACCAGTGTCGTAGACAACATGTGTCTTTACAACCAGCCTTGCCATGCATCAGCGAG

ATGGGAAATTGCTGTGGATGTGAACAACAATCATCCGCCATGTGGGGTGGCGAAGATTGGAGCGATCTGATATCCGACAAGCCAGCCAAGAGTAAAAGTAGTATCTTGCATCACAAGGAAAAGCATGAGAAGGTTGAGAGCAAAAAGTCTCTGTCAGGGGATATGAGAACCATGGCGCCTTCAACTACGGTTAAAATCAGGATCACAAAGAGGGAGCTCGACAATTTACTCGAGAAAGTGGAGATACAAGGTGTTTCATTCGAACAAGCCTTGAGTCAATCCATGGATGTGAGACAGCAGTGTCATAGACAGCATATCTCTTTGCAACCGGCCTTGCCATGTATCAACGAG

1485 Unigene28302_R-bungei CL5314.Contig3_R-chinensis

---------------------------------AAACCTGGTGGCGAAATCGGTTCGTGGCAAGAGAGAATTGATTGCTATATAAGAACTCTTGCTGCTGTTATTGGAAGTGTGGAAGCAGCAAAGGAGAAAATTTATAGGGTTATATGTGAGGATCAGTTTTTTGGCTTTGGTGCTGAGATTGACGAGGTTTCCGTAAACAAGCTTAAAGGTTTGTCTGGTGTTCTGGACGTTTTTCCTGATTACTTATTTAACAAGGATCACAAAGGTGTTGATTTATCAGATGGAGAGATATTCAGAAGCACCCTTGAA

CCTGCTTGCCAGCGTTGGTTTGTAGTTGTGGAGAAACCTGGTGGTGAAATCAGTTCGTGGCAAGAGACGATTGATTCCTGTATAAAAACTCTTGCTGCTGTTATTGGAAGCGTGGAAGCAGCAAAGGAGAAAATCTACAGGGTGATATGTGAGGATGATATTTCTGGGTTTGGTGCAGAGATTGACGAGGCTTTGTCAAATAAGCTTAAAGGTTTGCCTGGTATCCTGGACGCCTTTCCTGATTACTTATTTTACAAGGATTACAAAGGTGTTGATTTTTCTGATGGAAAACCATTTAGAAACACCCTTGAA

5548 Unigene27415_R-chinensis Unigene39912_R-bungei

TTGGTGGGCAAGTTCCAGAATCGAGTATCATTTGGAGTGCGAGCGGAAATCGTAGAGCTCACAAATATTCCATACGTAAAGGGTTCTCGAGCAAGGTCACTCTATAAATCCGGTTTGCGTACTCCGCTTGCCATTGCTGAGGCATCTATTCCCGAAATAGCCAAAGCACTTTTTGAA---TCATCATCATCATCATGGGCGGCTCAAGAAGGAACAGCACAGCGACGCATACAATTGGGTTTAGCTAAAAAGATAAAGAATGGCGCACGCAAAATCGTTCTTGATAAAGCAGAGGAGGCGAGATTAGTTGCATTTACAGCTTTTCAATCACTTGGTCTTGATGTTCCCCAGTTTTCTCGACCCTTGGCATCCATAGTCTCCGAGAACCCCATTTCACATGGTGCTGGAGATTCTCTAGGTAAAGATTATTTGGTAGGCGAAGAGTCCAAGAAGGATGTATCAGCTAAAGCAAATGCAGAAAGAAATGAGAATCCTAATTATTCTGTCGCACAGCTTGAAGTAGAGAACAAAAGTAATGTACAAGAATATGGTCTCAACACTTCC

------------------------------------------------------------------------------------------------------------------------------CTTGCCATTGCTGAGGCATCTATTCCCGAAATAGCCAAAGCGCTTTTTGATTCATCATCGTCATCATCATGGGCTGCTCAAGAGGGAACTGCGCAGCGACGCATACAATTGGGTTTAGCAAAAAAGATAAAGAATGGCGCACGCAAAATCGTTCTTGATAAAGCAGAAGAGGCGAGATTAGTTGCATTTACAGCTTTTCAATCACTTGGTCTTGATGTTCCACAGTTTTCTCGACCCTTGGTATCCATAGTCTCTGAGGACCCCATTTCACCTGGTGTCGGAGCTCCTAAAGGTGAAGATTGTTTGGTAGACGCAGAACCCAAGAAGGATGTATCAGCTAAACCAAACACAGAAAGAAATGCGAATCCTAATTATCCTGCTGCACGGCTTGAAGTAGAGAACAGAAGCAATGTACAAGAAAATTGTGTTGACACTTCC

4653 Unigene659_R-bungei Unigene20364_R-chinensis

AAAATTCGAATAGTAAGATGTCCTAAATGCTTGAAGCTTCTTACTGAATTGGAAAATATTCCGGTGTATCGATGTGGTGGATGTGATACAACTCTTAAAGCAAAAAAAAGAAAAGAAGTTCAGAAAATTGAAAGCTCAAAAGCGCTTAATCAATTCCCAGTGCAGAAAATTGGGCAAAAGTATGACTCTGAAGAAAATGATTCGGCCAACTCAAGTCAAAGGGCGCTCACGTCTTCAACAGATGGATATGAGCAAGATGATCTTGCAGATTTTCCTAAAGAAAAATCTGCAAATAGGAGGGCTTTGAATGGATCTGGTTCTTCTGATAAGATTCATTGGCATGAGAAAGGAGAGTCATCAATGGTAGCTGGAGGGAGTAGAAAAGCAAATGGAAACCATGAAAGGGGTCGTGACTTGGGATATCCAATTCATGACAGGCGAGGGATT------------------------TCTGTTCAGAAACTGACGGTTGAGAGTTCTCCATCAAAACCCAGTTCTTCGTCTTTAAGCCAAGAGTTAGATATATCTGAAAAGATTGTTCGCCGCTATGCTGATCTTATAGGGTCAAGGGGTCCAGTCAAAAGTCCTGATGCTAGCATCACCTCAAAACCACAAGCAAGCAAAACCTATCATGCCAGTGGGGCGAGTGCTTCTGCAACTGTTGTGCGGGAGAATCAGGCATCAGATCACACATCTAAGGAACATGGTCCTGAATATCAAAGAGTAGAAGAAGTCAGCAGTAGTAACAAAGGGGCTATAATGGAGGACATCTCTGTAGATGACAGGAACGATACCGTGTCAAAAGCACAAAATCAAACAAAGAAGTTATCAGTCATGTCTGTGAATGTGAATGAGAATCATGATACTAGTGTCCAGAGAAGTGATAGTCTGCATCAGAATCACGTAACAGAAAGAAATGTCATTCTGCAAGTACAAAATAACAAGGATATGTTGGGAAAATCGCTCAAGGATGGACTCCGATTTCCAGAATCATCAGGCACCAAAATCTTTCATGAGTATCATAGTAGTGTCTCTTCTGATGATGGAATGCTGGTGTCCGACCTTCAATCAAGTAAATCAGAGGAATCTATCTCTAGCTCTCAAAGGGTAGATGAAGTCAGTGTTTCAAACGAGGAAACTAAAATGAACGATGCTTCTATTAATAACAGGACTGATATCAAATCAGAAATATTTCCCGTGCTAATCACTGGGAATCATGTCCCAGTTGTTCTGGATAGTGTCAGTCTACATCAAGATGAGTTACCTCAATATACAGAATTGAGAAATCAAGTTCAAGATGATAACATCACATCAGAGGTTGATATGGATCATGATGGGATTGGGTCTGGCCTTGACGTTTCAAAATCATCAAATACCAAGATCTTTCATGAGTATGATCGTAGTGTTTCTTCTGATGCTGCCTGGGAAGCGCATGTTTCTGACATTCATTCTTTCTCTGGGTCTCAAAGGGTAGAAGAAGAACTTGTGGATACACACAAGGCTATAATGGAGGATGTGTCCGTTAATAGCAGAATTGATACTGAATCAGATGTCCCTCCAATGTTGATGAGTGGGAAGCACAGTCCAGTTGTTTTAGACAATGTGAGTCTGCAAGAAGACGAGTTACCTCAAAATATAGAATATGGCATTCAAGTTCAAGATGATAGCATCACTTCAGAAGATGAGATGGTTTGCAATAAGATTGGAGGTGACATTGGAGCTTCAAGTATACCAGCAATCAAAAGCGTTCATGCTTATGATGCCAGTGTGTCTTCTAACGATGGCTGGGACGATCAAGTTTCTGATTTTCATTTACACCAGTCTAAGGAATCATTCTCTGAAGCTCAAAAGGTAGCAGACAATGAGGCAATAAGGGGGAAAGACAGTTCAGCAGATGACATGATTGATTACGAACTGGATATGCGACGTCCATCAAGGAAGTTCTCATTAATGTCAGTGATTGAGAATCATAGTATGCATGATCAGGATGAGTTTCCTCAGTACAGCAGATACCAAATTCAAGTCGAAGATCAAGTGCATTCAAACTTCTCTAGCTTCAGGTCAACCCAAACTAGCTTTAAATCAACACAGAGCTGGCTTGGAGTTGACAGAGAAAGGTTGCAGACTCAGAAATACTCAACAGAAAGGTTTCCACGTGTGGATTACGAGGTAAGTTCCCCTCCCCCTTCCGCACGCAACTACCCACCACATACTCTTGACAGTGTTGAACGCAAGCAAATAGAACTCTTGAGGAAAGTGGAGGAATTGAGTGCACAACTCAATAGATCATATGGACACAACACGAGAGTAAATGAACGAATACAACCAAGTCCATATGAATCATATCTCCCGAGTAGAGTGACCTCTTTCCAATGCAGTTATCATCATATGCTCAATTGCTCCTGTTCAAATGCGTATAGTGGACAACAACAATTACTACCAAGGTCGCCACTGCCTAAACGTTACTACAGTGAAGATCTAGTAAGGAGATACTCTGATCAGGCATCCTACAACATATATAGCTCCGACCACTCTACTCCAGAGCCTCATGTACTGTCCCGCTGCCCATTGCGTGAAGGTTGCATACTTCCTTACAATCAGATAAACAAAAATCAAAGTGTGGAGCCTAGAGAGATACGTCCAAAGAGACACTACCGTCCTATAGCTGGAGGAGCTCCATTTATTACCTGCTACAGTTGCAAAAAGCTTCTTTTCCTCCCAGCTGATTCGTTCCTCTCGAACAAGAAACTTCACCGGCTACAATGTGGTTCGTGTTCCAAGGTACTCACGTTTTCATTTCACGGAGGATCTCATGTTTCTCCTTATTCTCAAAATGATGTC

AAATTCCGATTAGTCAAATGTCCTAAATGTTTGAAGCTTCTTGCTGAATATGAAAATGTGCCGGTGTATCGATGTGGTGGATGTGAGACAACTCTTAAAGCGAAAAAGAGAAAAGAAGTTCAGAAAGGTGAAAGCTCAAAAGCGCCTAATCAAATCCCAGTGCAGAAAATTGAACAAAAGTATGACTCTGAAGAAAATGATTCGGCCAACTCAAGTGAAAGGGCGCTCACGTGTTCCGCAGATGGATATGAACAAGATGATCATGCAGAA---------GAAAAATCTGCAAATAGGAGGGCCTTGGATGGATCTGGCTCTTCTGACGAGGCAAATTGGCATGAGAAAGGAGAGTCATCGTTGGTAGCTGGAGGGAGTAGAAAAGCAAATGGAAACCATGAAAGGGGTCGTGACTTGGGATATCCAACTCATGACAGGCGAGCGATTTCAGAGCGTAGTAACTTCACCTCTTCTGCTCAGAAACTAGCCGTTGAGAGTGTCCAATCAAAACCCAGTTCTCTGCCTGTCAGCGAAGAGTTAGATATATCTGAAAAGATTGTTCGCCGCTATGCCAATCTTATAGGGTCAAGGGGTCCAGTCAAAGTTCCTGACGCTAGCATCGTCTCAAAATCACAAGGAAGCAAAACCTATCCAGCTAATGAGGCCAGTGGTTCTTCAAATGTTGTCCTGGAGAATCAGGTTTCAAATCCCACATCTAAGGAACATGGTTCAGAATATCAAAGTGAAGAAGAACTCAGCAGTAGTAACAAGGGGTCTATAATGGAGGAC---------------------------------------------------------------------------------------------------------------------------------------------------------------------------------------------------------------------------------------------------------------------------------------------------------------------------------------------------------------------------------------------------------------------------------------------------------------------------------------------------------------------------------------------------------------------------------------------------------------------------------------------------------------------------------------------------------------------------------------------------------------------------------------------------------------------------------------------------------------------------------------------------------------------------------------------------------------------------------------------------------------------------------------------------------------------------------------------------------------------------------------------------------------------------------------------------------------------------------------------------------------------------------------------------------------------------------------------------------------------------------------------------------------------------------------------------------------------------------------------------------------------------------------------------------------------------------------------------------------------------------------------------------------------------------------------------------------------------------------------------------------------------------------------------------------------------------------------------------------------------------------------------------------------------------------------------------------------------------------------------------------------------------------------------------------------------------------------------------------------------------------------------------

9953 Unigene48395_R-chinensis Unigene19318_R-bungei

ATGGCTTCAATCTGCAGAGGTAAGGAGACCTGGCCAGAACTAGTTGGGGCGAGAGGGGACGTCGCAGAGAGAACAATTGAACGTGAAAACCCTAACGTCAATGCCATAATTAGACCTCCAGGATCTATTGGAACTACTGATTATCGGTGTGACAGGGTTTGGGTCTACGTTGATCAGAATTGGATTGTCGATCAGGTG------------

ATGGCTTCAATCTGTCATGGTAAGGATTCGTGGCCAGAACTAGTTGGGGCGACAGGGATTGTGGCAGAGAGGACAATTGAAAGTGAAAACCCTAATGTCGATGCAATCATTGTACCTCCAGGAGATGCTATAACTACAGAGTACCGGTGTGACAGGGTTGCGGTATGGGTTGATGAAAATGGGTTTGTTGTGGAGGTGCCCATAATTGGC

5233 Unigene25685_R-chinensis Unigene9491_R-bungei

------------------------------------------------AAGGAGACTCTGTACAGTAGAATTCATCCTTTGCGTGATCCAAGGGTTCTAATTACTCCGGTACTAGATCGGTGGATTCAGCAAGGAAATTCTATCAAGGATGAAGAAATCATCTCAATCATAAAGACTCTCAAATTCTCCAAAAGATTCAATCATGCTCTCCAGATTTCTCAGTGGATGGGCAACCAAAGTAACTTTCTCCTCTCAGGAAGCAACATTGCTGGTAAGCTGGACTTGATCTCAAGGGTTCATGGCGTTGAGAAAGCGGAAGAGTACTTTGATACCATTCCGAAGGAATCCAGAGTATTCCCAGTCTACCTTTCTCTGCTTAATTGCTATGTCCGTGGAAAATGCATTGAAAAAGCCGAGACCCTTATGCCACAGATGCTAGAGTCGGGGTTTACTGGGACAACACCAGTTTACAATAGTATGCTAAACCTTTATAGCCAAGTGAACGAGTATGAGAAGTTTGACAGCCTAGTCAAAGAAATGAAAAGTAGGGAAGTACGTCCAGACAAGTTTACATTTAGCCTCCAGTTACATGCCTACGCAGCCGTATCCAACTTTGATGGGATGGAGAATTATTTGCAGATGATGGAGGCAGACACCAACTTCAGTATGGATTGGAGGTGTTATGCTATTGCTGCAAATATATACATCAAATCTTACGTAATGGACAAGGCTCTGGTATTGCTGAAGAAGTCAGAAGAACTCGTCAACGGAAGAAATGCATATGAACATATTCTTACCTTGTATGCAGCCACAAGGAGTAAGGAG

GTTTCATTCTACTCAACAAAAAAATCCTCTTTACCTAAATCCTCGAAGAAGGAGAGTCTGTACAGTAGGATACATTCGTTCCATCATCCAAAGGCTGCAATTGCTCCGATAATAGATGAATGGATTGAGGAAGGCAATTCAATCAAGGATGATGAACTTATCTCAATTATAAGGAGTCTCAAATTCTCCAAAAGATTCAATCATGCTCTCCAGATTTCTCAGTGGATGAGCAACCAAAGTAATTTTCCCCACTCAGGAAGT------GCTGGTAGGCTTGACTTGATTACAAGGGTTCATGGCATTGAGAAAGCGGAAGAGTACTTTGATACCATACCAAAGGAATTCCGAACATTCCCAGTCTACCTTTCTCTGCTTCATTGCTATGCCCGTGGGAAATGCGTTGAAAAAGCCGAGACCCTTATGCAACAGATGCTGGAGTTGGGGTTTGCAGGGACAATACCAATATACAATGGTATGTTAAACCTTTAT---------------------------------------------------------------------------------------------------------------------------------------------------------------------------------------------------------------------------------------------------------------------------------------------------------------------------------------------

**Sub-file 2. BBH_ML_bun-tan.cds_Selected**

1045 CL3351.Contig3_R-tanguticus CL861.Contig1_R-bungei

---------------------GAGATTCTTCTTCGTCTTCCCGTAAAATCATTGGCAAGGTGCAAGACCTTGTCCAAAGATTGGCAGGAGTTTATCTCTCGCAATCCGTCTTTCCGTAAGCGTTTCATAGAACGAAATAGAGACTGCTCATTCATGCTAGGTCTTTACGTAACCAAACACATCACTGTAGTGGAAGAGCAA------CTTCACTTCCTTCCTACATATAATCAGGGAATATGCATGTTGAATAAAAGGAAGAGA---GATAATGATGGCAACTCTCTGCAGTTGGATGCACGCCCCGCAAAGAAAAAAAAT------------TTGAGTCCTCCTTTGTTGTTGAAGAAGTCACCCAGTTTTTTTAGTGACCCTGCAGTGCGAGTCCTGGGGTCATCTAATGGTTTTCTACTATGTTCGCTGGATAAGAAGTGTCCTTTGAATTATATAATTATTAATCCAATCAATAAGTTATGCATGCCTCTTCCAGTTGCCAACACCTCAAGTGATAATTACAGTCATGGATTCATCTGCCATTCATTGTCTCCTCTGCTTGATACTGTTGACTATTACAAAGTGGTCCGCGGAATATCTCCCCAAGATTGGCACGCAGAGAGTTTTACGACGCTCGACATCATCTGTTCAGACTCGAGTCAGTGGAGTCAATACCATGTAAGAGGCCAGTCACCTTTTTATTTGGATTCTTTAAATCCTTCGAAAATCATAATCTCCGAAGCAGGGTTAGTTTATGTGCCAGGTATCGCATTCAAAGAGGAA------TCAACAACACCTTGGTATGGTGAGAATAATAGTGTCCTCATCTTTGATCAAAATAAAGAAGAATCTGTACATGTGATAGAA---CTGCCGCCAGCAGAAAATAAAGTC---ATGCATGAATGCTTTGGTCAGTCCGAGGGAATGATTTCCTACGCTCGCAATGAAGTAGGCCAGCTCAAGGTATGGAGGTATAATGAGCAGAAG---GGTGGCGGATGGTCCTTGATACACAATGTAAGCCTGAATAATTGG---------------------------------------------------------------------------------------------

ATGAGAGACGATATTGCAGAAGAGATTCTTCTTCGCCTTCCCGTGAAATCATTGGTCAGGTGCAAGTTGTTATCCAAGGAGTGGCAGGAGTTCATCTCTCGCAATCCGGCTTTCCGTAAACGTTTCATTGAACGAAATAAAGGATGCTCATTCATGATAGGTTTCCACTTAACCAAACAAATTTATTTAATACATTTGGATTGGCTAGTTCACTTCCTTCCTACATATAATAAAGGAATAAGCTTGTTTAATAAAAGGAAGAGGGGCAACAATGGAGGCAGCTCTTTGCAGTTTGATGAGCCACTGAAGAAGAAAAGAGGACTTGAACGAAGATTAAGTCCTGTGTTGCTTTTGAAGAAATCACCCAACTTCTTTGGTGATCCTGCCGTACGCGTTTTGGGTTCATCTAATGGGTTTTTACTTTGTTCGTTGGATCCAAAGTGTCCCTTGAATTATGTTATTATTAACCCAATCAATAAGTTATGCGCGTCTCTACCGGTTGCAAATGCCTCGAGCGATTCTTACAGTCATGGATTCATCTGTTGTTCGACATCTCCTCAGCTTGATACTATTGACTACTATAAAGTGGTCCGTGGAATATCTCCCAAGAAATATCATAAAAAGAATCCTCTAATGCTTGAAATCATCTCTTCAGACTCAAGTCAGTGGAGCTCATTCAAAATGACAAGCTTGCCACCTTTTCTTTTGGATGATACAACGCCTTCGACAATCATAATTTCTGAAACAGGGGTGGTTTATGTGCCGGGTAGGGTATACAAAGAGACTCGCCGTAATGGTGAGGGTCTTCGTAATGGAGACAAAGGTGTCATCATCTTCGATGAAAGTAAAGTGGAACCTGTACTACGGGTGGAAAAATTCCCGCCAACGGAAGATTACTTGAACTGTAATGATCTCCTTGGTGAGTCTGAGAATTTGATTTTATATGCTCGGCATGAACCAGGGCGACTCAAGATATGGACGACGAATGACGACGAGGGTGGTGGAGGATGGACCTTGATACGCAATGTAAGCCTAGAAAGTTGGTTGCAAATCTACCCTGAACATTATAAATGTATCGAGGATGTGAGACTTTATATTCGAGGGTTCCACCCAACGAATAAGAATGTCATTTTCGTT

295 Unigene25660_R-bungei CL166.Contig2_R-tanguticus

ATTTCGAAAGGAATTGATGACGATGATGATAATGATGAAGTGTCGTCTGATTTTGATTCGGAGTCGGATAGGGCGGAGAGTTCGTCACCTGATGCTAGTATGGCAGATATCATGCCAATGCTTGATGAGCTCCACCCGTTATTGGATTTGGAGATTTCACAGCCTGCTCTTGTGTCAGTGGAGGATGATGATGATGTTTCCGAGGAGTCAGTTAGCTCCACTGATGAGTCGGAGGAGTCAGAGGAGGAGACTGAAAATGTGGAGATAGAAGATGAAGATGTGGAAGAAGAAGAAGAGGAGGAGCAACAACAAGAAAATGATGATGGAACGGAAGCTGTTGTAACGTGGACAAAGGAGGACGAAAAGAATCTAATGGATCTTGGTAATTCTGAGCTGGAAAGAAACCGGCGGTTGGAGAGTTTGATTGCTAGGAGAAGGGCAAGGAATAGCTTTAGAATGATGATGACTGAGAAGAATTTGATAGATTTCGAGGGTATGGATCCCCCATTTTCCATGGTGCCAATATCAACTGCAAGGCGGAATCCTTTTGATCTTCACTATGATTCTAATGAGGGAATTGATTTGCCACCGATTCCTGGATCAGCTCCATCTGTTTTGGCACCAAGGAGAAATCCTTTCGATATTCCTTATGACCCACTTGAAGAGAAACCAAATCTTCAAGGGGGAAGCTTTGATGAAGAGATCTTTTCAAACCTCGAGAAGGATATGCACTTCAGGAGGTATGAGAGCTTTAATGTTGGACCTTCTTCCCTAGGAGAATCCATGCAAGATAAGTCGAGCAGAAAGTTGAGACCTTATTTTGTACCAGAACCATTGCACTCAGAAGGGATTGGTTATTCCACTTTTCAAAGACAAATGAGTGAATTAAGTGAGTCCAAAATGAGTTCTGTTACGGAGGTTGAGTCAGTTTCTTCAGTGTCAGATCACGATAATCACAAGAATATCGCCGAAGACGAATCATTCCAATCTATTTCCCATGTTGAACAAGAAGTGTCTACCTCGATGTCCCATGTTGAGCATGATCGCGATCGTATTGAACTTGAAAGCTCATCCTCAGAAGAAATAGACATGTCTGAGGTTGAAGGAGATCAAGAATATCATAGAGGTGCTACTGTAGGTGAAATTGAAGGAGAATTATCCCAACCAATCCCTCGATCTGAACAAGAATTGTTTCATCCGATATCCCATCTTGAAGATGCTCACGACCATGTAGAAACAGAGGGCTCAATTTCTGAAGAAGTAAACATAGCCGAGGTTGATCAAGAATATCAGAGAGATGTTATTGTAGACGAGGAAAATGCAACAGAATATACTTCCGTCGAGATGCAATCCATTCCTTCACCTGTGGGGTCTGATACTGGTGATGTGACAGCTGATGTAGTCGAAGAGAAGCACACAGAATTGAGCTCTTCCTCTTCATCTGAAGCGAACACATGTGAAGGATTAGACAGCTCAGAGCAGAAAAGCGATGATAATATACTTAATAATTCAGCGGAACCATCTATTGAGGGTTCTGGTCTCCGGAGTGAACTTGTAGAAGTAGAAGATATTCATGCAAAGGGACCTCTATATGATTGGAATTGGAACCCGTCAGAAGTAGAGAAGACACTTTCTGGCATATCAATTATTGAAGAAGCTTTAAATTATGTGGATAGAAGAGCTCTAACTTCTACTTCATCGGCAGCATCAGACATAGCTGAAGAAGCTGGGGAAGTAGGTCCAATTCCAGTTCAAGTTGAAAGAACCATTTCATTTATGGACGAGGAACCACTGCTGCATAATGGGAGTATGGAAGAGGTTTCGCATTCACATGCAGTTGATGTGAACAATTTAAGCTCTACCGAAGTAACTGAGACTAATGAGCATGATGTTATTGAAGCTGCAATTTCTGAAGATATCCAGAATGAGTCCAACTTAACTGCTCCTGTGACGCCGTTAGTAACTGAGCAAGTCATATGTGGCTCAGGTTCATCTTCATCAGAAACAGAATTTGAAGATGGAAGTTCATTGAGCAAGGAAGAAAATGAGCACCAACAAGTCCATATAACTGCCACTGACCTCAATCCAGTCCATATTGATTTTTCTAAGTCAAATGTGGATGTGGCAGACCGCAAAGCTACAGGAGTGTCATCTGAAGGTTCAGTTCGTATCATGTCAGAGGTTCCACCACCCTTGTTGGAGGAAACAATGGCTCATTCGTCATCTGGTGGTGATCATGAAAGCTCTCAGGAACCATATAGTCTGTTGGCTGTTTCTGCCGTTATGTCCAAAGCTAATGATGATTTCTCTGAGGTGGTGCATGTGGATGGTGGAGCGGATTCCTCGAGTGACACTGTTATGGTTTCTTCCCCTGCTTTGGTGACATCAGAAATTTCTGACAATGACCCTAATGATGAACACCAGAGAAGTCTATACTTTGAGGATTTCGAGCACGCAAATGTTTCACAGAGAGCATCTGGGATTGATCTCACTGACAGAGAGTTAGAT---------GGAATCACAGAGATTGATGAAGGATTCCTGACAGAGTTGGATGGAGTTGGCGACTTTGCTGTTGAAGAAGAGAGCCTTACGAGGCTACACGCCGTTCATGGTGAACCATCCCAATATGTTGAAAGCCACTCCCATAGTTCCCAATATTCAAGAGAGGCCCTCGCACACGCTCATGAACATGATTTTACCCGAGAGCAAAATGAATCCGTGGATGCTTCTACAGTACTTTCACCAAAGTTGGAGTCAGCCATTGAAATCACATTAGATTCCAAAGAATTGATGGTTGAAAGTGCCGAAGAAGAAAATTCCAAAGACAAAGAAACTGGTGAAACTTTAAGTTCAAGCAACATAGATGACAGTAAGACAACCACAGAACTCAAGGCAGTTGATGATGTGAATGTTGAATCTCTGGAGACAAACATAAACCAAACTGAAAACATTTCTAATGAAGTTGAGAGCCTCGCAGAGTTACAAATTGTCAAAGACACCTCTTCCAAAGATTTTGAAGATCCTGAAGCAAGATCAGAGGAAGAGGTCCATTTGAGCTCCCCACAACTCTCAAAATCAAGAGATATCTTAGCAGAAGTAGTCGATTCAGATATCAAATCACCTTCTGAAACAAGTGAATCAAAGGAGGCCATGAACCAAGCCAGTGTTACAGGGATACAAGTTCTTGAAGATAATGTTTCCAAAGATAATACCCACAAAATAGAGGATTTGCATTCCGAAATTTCCACAGGGACAGTTGAGTCATCCCTAGACGTGGTATCACACCCCGAAATAACTGGACCCCAGAAGTGTGTGGTACAACCAAGTCGCGCAGAGGTACAAGTTCTTGAAGACAATGTTCCCAAAGATGTTGAAAACAACTCCCAGAAAACAACAAATTCGCAGGAAGTTGGA------------ACAGAGATCCATGCAACTGAGAGGGAACCGCCAGTTGAAGAAGATCAGCCAAAACCAAGTGAATCAGTGGACGCACCGGCAGAAATTTTGCCCAAGATAGCCAAAGTACATCCCGAAGGATTCCCTGCCGAAGAAATTGAATCAGTCTTTAGTGTTAAAGAAACAGAGGAGGCTTCAAGCTCGAGCGCAACAGTTGTAAAGAAGAAGAAGAAAAGCTCTGGTAAGTCTGGCTCTAGCTCAAGTTCTAGTTCTAGTTCTAGCTCC------------

------------------------------------------------------------------------------------------------------------------------------------------------------------------------------------------------------------------------------------------------------------------------------------------------------------------------------------------------------------------------------------------------------------------------------------------------------------------------------------------------------------------------------------------------------------------------------------------------------------------------------------------------------------------------------------------------------------------------------------------------------------------------------------------------------------------------------------------------------------------------------------------------------------------------------------------------------------------------------------------------AATATCGCTGAAGAAGAATCATTCCAATCTATTTCCCATGTTGAACAAGAAGCGTCTACATCGATATCCCATGTTGAGCATGATCGTGATCATATTGAACTTGAAAGCTCATCCTCAGAAGATATAGACATGTCCGAGGTTGAAGGAGATCAAGAATATCATAAAGGTGCTATTGTAGGTGAAGTTGAAGGAGAATTATCTCAACCAATCTTTGAATCTGAACAAGAATTGCTTCATCCAATATCCCATCTTGAAGATGCTCACGACCATGTTGAAATAGAGAGCTCAACCTCTGAAGAAATAGACGTAGCTGAGATTGATCAAGAATATCAGAGAGATGTTATTGTCGACGAGGAAAATGCAACAGAATCTACTTCCGTCGAGATGCAATCCATACCTTCACCTGTGGGATCTGATACTGCTGATGTGACACCTGATGTAGTTGAAGAGAAGCACACAGAATTGAGCTCTTCCTCTTCATCTGAAGTGAACACAAGTGAAGGATTAGAAAGCTCAGAACAGAAAAGAGATGATAATATCCTTAACAATTCAGAGGAAGCATCTATTGAGGGTTCCAGTCTCCGTAGTGAACTCGTAGAAGTAGAAGATATTCATGCAAAGGAACCTCTATACGATTGGAATTGGAACCCGTCAGAAGTAGAGAAGACGCTTTCTGGCATATCAATTATAGAAGAAGCTTTAAATTATGTGGATAGAAGAGGTCTAACTTCTACA---TCGGCAGCGTCAGACATGGCTGAAGAAGCTCGGGAAGTAGGTTCAGTTCCAGTTCAAGTGGAAAGAACCATTTCATCCATGGATGTGGAACCACAGCGGCATAACGGGAGTATGGAAGAGGCTTTGCATTCACATGCAGTTGATGTGAACGATTTAAGCTCTACGGAAGTAACTGAGAGTAATGAGCATGATGTTATTGAAGCTGCAATTTCTGAAGATGTCCAGAATGAGTCCAACTTAACTGCTCCTATGATGCCGTTAGTAACTGAGCAAGTCGTATGTGGCTCAGGTTCATCTTCATCAGAAACAGAATTTGAAGATGCGAGTTCACTGAGCAAGGAAGAAAATGAGCACCAACAAGTCCATATAACTACCACCGACCTCAATCCAGTCCATATTGATGTTTTACAGTCTAATGTGGATGAGGCTGACCCCAAAGCTACAGGAGTGTCATCTGAAGGTTCAACTCATATCATGTCAGAGGCTCCACCCCCCTTGTTGGAGGAAACGATGCCTCATTCGTCATCTGGTGGTGATCATGAAAGCTCTCAGGTACCATCTAGTCCGGTGGCTGTTTCTATCGATATGTCCAAAGCTAATGATGATTCCTCTGAGGTGGTGCATGGGGATGGTGGAGTGGATTCCTCGAGTGACACTGTTATGGTTTCTTCCCCTGTTTTGGCGGCGTCAGAAGTATCTGAGAATGACCGTAGTGATGAAATGCAGAGAAGTGTATACTTCCAGGAGTTAGAGCACGCAAATGTTTCGCAGAGAGCAACTGGGGTTGATCTCACTGACAGAGAGTTGGATGAGGTTGAAGGAATCACAGAGATTGACGAAGGATTCCTGACAGAGTTGGATGGAGTTGGTGATTTTGCTGTT---GAGGAGAGCTTCACGGGGCTACACGCCGTTGATGGTGAGCCATCCCAAGATGTTGAAAGCCACTCCCGTAGTTCCCAGGATTCAAGAGAGGCCCTCACGCACACGCATGAACAGGATTTTACTCCAGAGCAAAATGAG------------TCAGAAGTACTTCCACCAAAGCTGGAGTCAGCCATAGAAGTCACATTAGATTCCCAAGAACTGATGGTTGAAAGTGCCGAAGAAGAGAACCCCAAAGATGTAGAAACAGTTGAAACTTCAATCTCAAACAACATAGATGAAAGTAAGAAGACAACAGAACTCGAGGCAGTTGGCGATGTGACTGTTGAGTCACTCGAGACAAACATAAACCAAACTGAAAACATTTCTAAAGATGTTGAGAGCCTTACAGGTTTACAAATTGTTGAAGACGACTCTTCCAAAGATGTTCAAGATCCTGAACCAAGATCA---GAAGAGGTTCATTTGAGCTCCCCACAACTTTCATTTTCAAGAGAAATCCCAGCAGACATAGTTGATTCAGATATCAAATCACCTTCTGAAACAAATGAATCTGAGGAGGCCATGAACCAAGCCAGTGTTACAGGGATACAAGTTCTTGAAGACAAT------------AATACCAACAAAATAGAGGATTTGCATTCCGAAATTTCAACAGCGGCAGTTGATTCAGCTGTAGATGTGACATCACACCCTGAAATAACTGAACCTGAGAACGCTGTGGTACAGCCAAGTCTCACAGAAGTACAAGTTCTAGAA---AACGTTTCCACGGATTTGGAAAACAACTCCGAGAAAACAGAGGATCTGCAGGAAGTTGCACCAGTATTAAAAGAAGAAGTGCATGTAACTGAGAGGGAACTGCCAGTT---GAAGGGCAGCCGCAACCAAGTGAATCTGTGGATAAGCCAGCTGGAATTCTGCCGAAGGTAGCCAAAGTACATCCCGAAGGAATCGCAGCTGAAGAAAGTGAAACAGTCTCTAACCCTGAAGAAACAGGGGAGCCTTCAAGCTCGAGCGCAACAGTTGTAAAGAAGAAGAAGAAAAGCTCTGGTAAGTCTGGCTCTAGCTCAAGTTCTAGTTCTAGCTCCAGCTCAAGTGATTCTGAT

8331 CL7691.Contig1_R-bungei Unigene26373_R-tanguticus

------------GATTTTAGTGAGAAAACGCTCACAAAGAAGATAATAGCATGCTTGACTGGAACAGAAAGCACGCTTGCAAATTTGGAACCTCTGAAAAATCACCTCAAACAGTTACTGAACGGAAAGAAGTTTTTACTAGTTCTAGACGATGTCTGGAATGAAAATCCTGAAATTTGGCAAAGGTTGAGGTCTTCGCTAAAC------GCTAACGGAAGTTCAGTTGTTGTCACGACTCGTCTACCCACTGTTGCAAAGATAATGGGTACACACTCTGTGTACGACTTAACAACTCTATCTGATGATGACTGCTGGGCACTGTTCAGAGGATGCGCGTTTGGAATGGACGACGAAGCTGTTGAAAATCTGGAAGCAATTGGCAAGCAAATTGTTAAGAAATGTGGAGGTGTACCTCTTGTAGCAAAAGCACTAGGCGGCTCGTTGCTTTTTGAACGCGGTGCAAATATGTGGAAGTCTGTTAGGGATAACGATATCTGGAAGCTAGAACGTGAAAAGGGTACTATTTTACCATGTCTGAGATTAAGCTATAACCGCTTACCGTCATATTTGAGAGAATGTTTTGTGTATTGTTGTGTATTCCCAAAAGATTATGTAATGGTCGTAGAAGAACTGATTCAGTTGTGGATGGCAAACGGGCTTCTTCAGTCAGATGGAAGAATGGAGTTGGAGGAAGTCGGTAACCGTATATTCAACCAACTAGTATTGCGATCCTTCCTCCAAGATGTCAGTGTAAATAGTGATGGGAACCAAGTGTGCAAAATGCACGACCTTATGCATGAACTCGCATGTTCTGTTGCACGAAAAGAATGCCATGCTTGGGAGGTGCACGAAGAATTAGTTACAATTCCCAAAAGCGTTCGGCATCTATGGATTGATCGGATTCCGTTCATACTTGTTAAGGCTCAACTTAAGTCACCCCTTCCCTTGCGTACATGTATCTTTTCTCGTGGTTCTTACATGTATGACCATCCTTCATTTGAAAAAACCTTAAAAAACCTCACTTGTTTGCGGGTCTTGCAGTTCCAAGCCCCCCTAACCCTACCACAGTCAATAGAGAAGATGTTACACTTAAGGTATCTCAACCTTTCATCGTCCCTTATTGAACATTTACCCAAATCCATTTGTAGCCAAAAAAACTTGCAAACGTTAATTCTGAATTCCTGTCGTCACCTTAATAAATTGCCGGAATCTCTGAACAAGTTGATCAACCTGAGACACTTGGACTTGAGTAAGTGTGGGAGCTTAACGCAATTACCAGATGGGATTGGAGGTTTGATCAATCTT

TGTGTATCTGAAGATTTTAGTGTGAAAAGACTCACAAAGAAGATAATAGCATCTTTATCTGGAAAAGAATGCAAGCTTGAGGATTTGGATCCTCTGCAAAACCGCCTCAAGGAGTTACTGAATGGAAAGAAGTTTTTACTAGTTCTAGACGATGTCTGGAATGAAGATCCTGAAATTTGGGAAAGGTTGAGGTCTTCGCTAAAATGTGGGGCTAATGGAAGTTCAGTTGTTGTCACTACTCGTCTACCTACTGTCGCAAAGATCATGGGTACACACTCTGCGTACGACTTAACAACTCTATCTGATGAAGACTGTTGGGCATTGTTCAGAGGGCGCGCTTTTGGAATGGAGGATGAAGCTAATGAAAATCTTGAAGCAATCGGCAAGGAAATTGTTGGAAAATGTGGAGGTATACCTCTTGTAGCAAAAGCACTAGGCGGCTCGTTGCGGTTTGAACGTGATCCAAGTATGTGGAAGTCTGTTAGGGATAATGAGATTTGGAAGTTAGAACGAGAAAAGGGTGCCATTTTACCATGTCTGAGATTGAGCTATAACCACTTACCGACATATTTGAGAGAATGTTTTGTGTATTGTTGTGTATTCCCAAAAGATCATGAAATAGAAGTTGAAGAATTGATTCAATTGTGGATGGCAAACGGGTTTCTTCAATCAGATGGAAGAGTGGAGCTGGAGGAAGTTGGTAACCGTATATTCAACCAGCTAGTATTGCGATCCTTCCTCCAAGATGTTAATGAAGATCGACGCGGGAAACAGGTATGTAAAATGCACGACCTTATGCATGACCTCGCATGCTCTGTTGTACGAAAAGAATGTTATGCTTTGGAGGAGGGTGACAAGTTAGATACACCTCTTAAAAGCGTTCGCCATTTATGGAGTCATTCAGGGAAATCT------GTTGAAACTCTATTTAAATCACCCATGCCCTTACGTACATGTCTCTTTCCTATTTGGGAGACTATGTATGGC---CCTTTGGTTGAAACAATATTTAAATACCTTACTTGTTTGCGAGCCTTGCATCTCAGAGGATCCCCAAGCCTACCACCGTCAATAGGGAAGATGATACATTTAAGGTATCTCAACCTCTCATCGTCCCATTTTGAACATTTACCCAAATCCATTTGTAGCCTAAAAAACTTGCAAGCCTTAGATCTGCATAGTTGT------------------------------------------------------------------------------------------------------------------------

10498 Unigene31235_R-tanguticus Unigene659_R-bungei

AAAATTCGAATAGTAAGATGTCCTAAATGTTTGAAGCTTCTTACTGAATTGGAAAATGTTCCGGTGTATCGATGTGGTGGATGTGATACAACTCTTAAAGCAAAAAAGAGAAAAGAAGTTCAGAAAAGTGAAAGCTCAAAACAGCTTAATCAAATTCCAGCACAGAAAATTGAAGAAAGGTATGACTCTGAAGAGAATGATTCTGCCAACTCAAGTCAAAGGGCGCTCACGTGTTCCACAGATGGATATGAACAAGATGATCATGCAGATTTTCATAAAGAGAAATCTGCAAATAGGAGGCCATTTAATGGATCTGGTTCTTCTGATGAGGTTAATTGGCATGAGAAAGGAGAGTCATCGTTGGTAGCTGGAAGGAGTAGAAAAGCAAATAGAAACCATGAAAGGGGACGTGACTTGGGATATCCAACTCCTGACAGACAAGGGATTTCTGAACATAGTAACTTTACCTCTCCTGTTCAGAAACCAACCGTTGAGAGTTCCCCATCAAAACCCAGTTCTTCGTCTTCAAGCAAAGAGTTAGATATATCTGAAGAGATTGTTCGCCGCTATGCTGATCTTATAGGGTCAAGGGGTCCAGTCAAAAGTCGTGGTGCTAGTATCATCTCAAAACCACAAGCAAGCGAAACCTATCATGCTAATGGGGCCAGCGGTTCTTCAAATGTTGTGCGGGAGAATCAGGTTTCAAATCACACATCTAAAGAATATGGTTCTGAATATCAAAAAGTAGATGAACTCAGCAGTAGTAACAAGGGGGGTATACTGGAAGACATCTCTGTAGATGTCAGGAACGATGCCGAGTCAAAAGTACTAAATCAAACAAAGAAGTTGTCAGTCATGTCT------GTGAATGAGAATCATGATACTAGTGTTCAGAGAAGTGTTAGTCTGCGTCAGGATCAGTTAGCAGAAAGAAATGTCATTCTGCAAGTACAAGATGACAAAGATATGGTGGGAAAATCGCTTGAGGATGGACTTCAATTTTCAGAATCACCAGGCACCAAAATCTTTCATGAGTATAATAGTAGTGTCTCTTCTGATGATGGAGTGCTGGTGTCCGACCTTCAATCAAATAAATCGGAGGATTCTATCTCTATCTCTAGCTCTCAAAGGGTAGACGAAGTCATTGTTACAAACGAGGAAACTACAATGGAGGATGCTTCTATTAATAACATGACTGATACCAAATCAGAAATATTTTCCATGCCAATCATTGGGAATCATGTCCCAGTTGTTCTGGATAGTGTCAGTCTACATCAAGATGAGTTACCTCCAAATACAGAATTGAGAAATCAAGTTCAAGATGGTAACTTCACATCAGAGGATGATATGCTTCATGATGGGATTGGGTCTGTCCTTAACGTTTCAAAATCATCAAATACCAAGATCTTTCATGAGTACGATCATAGTGTTTCTTCTGATGCTACCTGGGAAGCGCATGGTTCTGACATTCCTTCTTTGTCTGGGTCTCAAAGGGTAGAAGAAGAACCCGTGGATACACACAAGGCTATAGTGGAGGATGTCTCCGTTGATAGCGAAATTGATACTGAATTAGATGTCCCTCCAATGTCAATGAATGGGAATCACAGTCCAGTTGTTTTGGGCAATGTGAGTCTGCAAGAAGATGAGTTACCTCAAAATACAGAATGTGGCATTCAAGTTCAAGATGATAGCATCACTTCAGAAGATGAGATGGTTTGCAATAAGATTGGAGGTGACATTGGAGTTTCAAGTATACCAGCAATCAAAAGCTTTCATGCTTATGATGCCAGTGTTTCTTCTAACGATGGCTGGGGCGATCAAGTTTCTGATTTTCATTCACACCAATCTAAGGAATCATTCTCTGAATCTCAAAAGGTAGCAGACAATGAGGCAATAAGGGGGAAAGAAAGTTCAGTAAATGACATGATGGATACGGAACCGGATATGCGACGTCCATCAAGGAAGTTCTCATTAACTTCTGTGATTGAGAATCATAGTATGCATCATCAGGATGAGTTTCCTCAGTACAGCAGATACCAAATTCAAGTTCAAGATCAAGTGCATTCAAACTTCTCTAGCTTCAAATCAACCCAAACTAGCTTCAAATCAACACAAAGCTGGCTGGGAGTTGATAGAGAAAGGTTGCAGACTCAGAAATACTCAACAGAAAGGTTTCCACATGCTGATTACGAGGTAACTGCCCTTCCC------ACACGCAACTACCCACCACATACTCTTGACAGCGTTGAACGTAAGCAAATAGAACTCTTGAGGAGAGTGGAGGAATTGAGTGCACAACTCAATAGATCATATGGACACAACACAAGAGCAAATGAACGGTTACAACCAAGTCCATATGAAACATATCCCCCAAGTAGAGTGACATCATTCCAATGCAGTTATCATCAAATGCCCAATTGCTCCTGTTCAAATGCGTATAGCGGACAACAGCAATCACTACCAAGGTCGCCACTGCCTAAACGTTACTACAGTGAAGATCTAGGCAGGGGATACTCTGATCAGGCATCCTACAGCGTATATAGCTCCGATCCCTCCACTCCAGAGCCTCATGTAGTGTCCCGCTGCCCACTGCGTGAGGGTTGCATACTGCCTTACGACCAGATAAACAAAAATCAAAGTGGGGAGCCTAGAGAGAAACGTCCAAAGCGACATTACCGACCCATAGCTGGAGGAGCTCCGTTTATCACCTGCTACAGTTGCAAAAAGCTTCTTTTCCTCCCAGCTGATTCCTTCCTTTCCAACAAGAGGCTTCACCGGCTACAATGTGGTTCGTGTTCCAAGGTACTTACATTTTCATTTCACGAAGGATCTCTTGTTTCTCCTTATACTCAAAATGACGTC

AAAATTCGAATAGTAAGATGTCCTAAATGCTTGAAGCTTCTTACTGAATTGGAAAATATTCCGGTGTATCGATGTGGTGGATGTGATACAACTCTTAAAGCAAAAAAAAGAAAAGAAGTTCAGAAAATTGAAAGCTCAAAAGCGCTTAATCAATTCCCAGTGCAGAAAATTGGGCAAAAGTATGACTCTGAAGAAAATGATTCGGCCAACTCAAGTCAAAGGGCGCTCACGTCTTCAACAGATGGATATGAGCAAGATGATCTTGCAGATTTTCCTAAAGAAAAATCTGCAAATAGGAGGGCTTTGAATGGATCTGGTTCTTCTGATAAGATTCATTGGCATGAGAAAGGAGAGTCATCAATGGTAGCTGGAGGGAGTAGAAAAGCAAATGGAAACCATGAAAGGGGTCGTGACTTGGGATATCCAATTCATGACAGGCGAGGGATTTCT------------------------GTTCAGAAACTGACGGTTGAGAGTTCTCCATCAAAACCCAGTTCTTCGTCTTTAAGCCAAGAGTTAGATATATCTGAAAAGATTGTTCGCCGCTATGCTGATCTTATAGGGTCAAGGGGTCCAGTCAAAAGTCCTGATGCTAGCATCACCTCAAAACCACAAGCAAGCAAAACCTATCATGCCAGTGGGGCGAGTGCTTCTGCAACTGTTGTGCGGGAGAATCAGGCATCAGATCACACATCTAAGGAACATGGTCCTGAATATCAAAGAGTAGAAGAAGTCAGCAGTAGTAACAAAGGGGCTATAATGGAGGACATCTCTGTAGATGACAGGAACGATACCGTGTCAAAAGCACAAAATCAAACAAAGAAGTTATCAGTCATGTCTGTGAATGTGAATGAGAATCATGATACTAGTGTCCAGAGAAGTGATAGTCTGCATCAGAATCACGTAACAGAAAGAAATGTCATTCTGCAAGTACAAAATAACAAGGATATGTTGGGAAAATCGCTCAAGGATGGACTCCGATTTCCAGAATCATCAGGCACCAAAATCTTTCATGAGTATCATAGTAGTGTCTCTTCTGATGATGGAATGCTGGTGTCCGACCTTCAATCAAGTAAATCAGAGGAA------TCTATCTCTAGCTCTCAAAGGGTAGATGAAGTCAGTGTTTCAAACGAGGAAACTAAAATGAACGATGCTTCTATTAATAACAGGACTGATATCAAATCAGAAATATTTCCCGTGCTAATCACTGGGAATCATGTCCCAGTTGTTCTGGATAGTGTCAGTCTACATCAAGATGAGTTACCTCAATATACAGAATTGAGAAATCAAGTTCAAGATGATAACATCACATCAGAGGTTGATATGGATCATGATGGGATTGGGTCTGGCCTTGACGTTTCAAAATCATCAAATACCAAGATCTTTCATGAGTATGATCGTAGTGTTTCTTCTGATGCTGCCTGGGAAGCGCATGTTTCTGACATTCATTCTTTCTCTGGGTCTCAAAGGGTAGAAGAAGAACTTGTGGATACACACAAGGCTATAATGGAGGATGTGTCCGTTAATAGCAGAATTGATACTGAATCAGATGTCCCTCCAATGTTGATGAGTGGGAAGCACAGTCCAGTTGTTTTAGACAATGTGAGTCTGCAAGAAGACGAGTTACCTCAAAATATAGAATATGGCATTCAAGTTCAAGATGATAGCATCACTTCAGAAGATGAGATGGTTTGCAATAAGATTGGAGGTGACATTGGAGCTTCAAGTATACCAGCAATCAAAAGCGTTCATGCTTATGATGCCAGTGTGTCTTCTAACGATGGCTGGGACGATCAAGTTTCTGATTTTCATTTACACCAGTCTAAGGAATCATTCTCTGAAGCTCAAAAGGTAGCAGACAATGAGGCAATAAGGGGGAAAGACAGTTCAGCAGATGACATGATTGATTACGAACTGGATATGCGACGTCCATCAAGGAAGTTCTCATTAATGTCAGTGATTGAGAATCATAGTATGCATGATCAGGATGAGTTTCCTCAGTACAGCAGATACCAAATTCAAGTCGAAGATCAAGTGCATTCAAACTTCTCTAGCTTCAGGTCAACCCAAACTAGCTTTAAATCAACACAGAGCTGGCTTGGAGTTGACAGAGAAAGGTTGCAGACTCAGAAATACTCAACAGAAAGGTTTCCACGTGTGGATTACGAGGTAAGTTCCCCTCCCCCTTCCGCACGCAACTACCCACCACATACTCTTGACAGTGTTGAACGCAAGCAAATAGAACTCTTGAGGAAAGTGGAGGAATTGAGTGCACAACTCAATAGATCATATGGACACAACACGAGAGTAAATGAACGAATACAACCAAGTCCATATGAATCATATCTCCCGAGTAGAGTGACCTCTTTCCAATGCAGTTATCATCATATGCTCAATTGCTCCTGTTCAAATGCGTATAGTGGACAACAACAATTACTACCAAGGTCGCCACTGCCTAAACGTTACTACAGTGAAGATCTAGTAAGGAGATACTCTGATCAGGCATCCTACAACATATATAGCTCCGACCACTCTACTCCAGAGCCTCATGTACTGTCCCGCTGCCCATTGCGTGAAGGTTGCATACTTCCTTACAATCAGATAAACAAAAATCAAAGTGTGGAGCCTAGAGAGATACGTCCAAAGAGACACTACCGTCCTATAGCTGGAGGAGCTCCATTTATTACCTGCTACAGTTGCAAAAAGCTTCTTTTCCTCCCAGCTGATTCGTTCCTCTCGAACAAGAAACTTCACCGGCTACAATGTGGTTCGTGTTCCAAGGTACTCACGTTTTCATTTCACGGAGGATCTCATGTTTCTCCTTATTCTCAAAATGATGTC

8873 Unigene17884_R-bungei Unigene27426_R-tanguticus

GTTGTTGCTGCGAATGTTCTTTCTAGTATGTGCATCATAGCAGAGGATACAGAACCATATCTTTTTAGCAATGTATCCCTCGTTTCAGATGATATACAGATTACAGATTTGAGATATTCAATCAATGCAATTTTGTCTGAGGAAGTACCTAAAAATGAAGCTTTGTTGGTTGCTATTTTGAAACTTCTCACCTCTGCAGCACTCCACCAGCCTGCTTTTCTTATATCTTTGATTGAGCTGGAGGAGCATATGGATTCTAAACACCAATCAGTGCAAACTTCGGTGCAACCACTGAAGCCTACGGGAAAAAATATCTTAGAGGCATTACTGCAATTTGTCAGAAGATCAGGGGATCTTATTGAGAGCGGTCCAAATATACTTCTGAATGTGCTTATGTTTTTAAAAGCTTTGTGGCAAGGAAGTATGCAGTATATTCAGATGTTGAAGTGGTTCAGAGCTTCTGAGGTTTTCTGGATGCAATTGTCCACCATTCTAGATGTTGTGATGACTGAGGATCCTCCTGCTTTGCTGGGAGATGGCACACTTAGCCTCGCCTGCAAGTACCAGTGTTGTTCAGTGATACTAGAAATTTTGGCATATGACTTGTTTCTGGAGAAAAAATTAGTAAACGCCGAGTCTCCAATAAAACCGACTTCTGAAAAATCTCAAGCTAGTAGTCCAGCTTCTGTGCAGAATATATTGTTGAAATGGTGTAAATTTCCAGCCTTAAAAAGTCTGATAAATTTATATGCATCTTGTGAATATGACTGCGAACTACTTCTCCGTGCAAAGGTCGCTTTAAGCACGTTTCTTGTGCATGTGATGGGGAAATTAACTGCTGATGATGCTGGGAGTTTGTCTTTGTCATTTTGTCGGAAGATTCAAGACATTTTAAAGAAGTTAATCACTCAACCTGCATTTTCCGATCTTCTAGCTCAATATACCCAGCAGGGATACAGTGGCGGCAAGGAACTGAAAACTCTAGTACTTAATGATCTTTACTACCATATGGAAGGCGAGCTAGAAGGTCGTAAAATTAACTCTACACCATTCAAAGAGCTCGCTCAGTTTATGTTACAGTTAAGGTTTTTGCACACTGAGCTGAAATACAGAAGAGACTGTCCTGAACACGCCAGTGATGTGTATGTATTTGATCTTGTACGCTTGCAAAAAGATTTGGGATACGAGTATTGGGAGTACTCAAACTGGAAGGCATCTAAAGTAATTGCTGAAAAGATGATGTTTTACATGCGAGATGCAAACATGATGTCTTATCTTGCAAGTTCCAAGCTTTGCGCGTTGGACGCTCTGACAACCATATTATCTGTTAATGGAGAAGAGTTAACTGGAACAAAGAACATTTCAGATGGTAAAGTGATTGAGTCGTGCATTGAACTAGTCTGTGAAAAATTGGAGACAATTGTGGAATCATTAATTGCACCTATCATTCCTTGTGTTGAAGTTATTGATCATGTGGAAGCGCAAACAAGATTGCTTCTCTGTCTGGTCAGATTACTAGTAGCTAACGGGGACAAGGCAAATGTTTGTAGTCGTGTCTTGAACACTGCAGGTAGCAGTCTCAGAGTGATT---AGCAGCAGCAGCATCGTGATGACATCTAGGAGGGTTGAGGGAGTCATGAAACTCTTACTTGCAGTGCTTCTGGTATCAGTTGAAAATAGTATCCTACATGAAAAACAAGGTGAAAGTGTGATGAGGGTGTTGCCAGTTTTGTGCAGCTGTGCCCAGGTTGGAGAGCAGTGCTTTACTATCTCGGTGGCCACAATGAACTTGATACTGAATGGTTTCTTGACGTGCGATACGTGGATACCCATCATACAAAGGCATCTAAAACTGGATGTTATAATTCTCAAGCTCCGAGAGAGAGGAGCGTCTTATACGTGTGTTTCGGTGGTAATGAAATTTCTCTTGAGCCTTGCACGAGCAAGAGGAGGTGCAGAGATGCTTGAGAAAGCAAATTTGTTCCCATCACTTAAAGTGTTAAATAGCTTGTTGTTGGACGAAAAGCCTTTGTTTGACCAAGACGAGAAGCGCCAGAGAGTTTGGGGACTTGGCATGACTGTTGTGACGGCAATGATAGAGTCTCTGGGGGTTTTTTCAGATTTTGTAGACAGCAGTGTGATACCATATTATTTTGGTGAGAAGGCGTTTATACTGTTTCATAATCTTGATTTGGTAAAGAGCAAAACGTCCCTGACGTCTCTGAGGGAAGTAGAGAATACTCTGATGCTGATATGTGCAGTGGCAAAAAGGAAGTGCAAGTGGAATGTGCAACTGAGGGAGACGTGTATCCATATATTGGCACTAATAGGCAACAGTCATCAACAGCAGATTGTATGTCCTCCGATCTTGAAAGAGGAGCTTGAATTGAACGCAAGAGCTTCTTCATTTGTGAATTGCAGCAGCAAGCGTTGTTGTCTTGGCGGCACGTGTTTTTCAGATGCAGTGGCAGTAGAGATATACAGGATTGCTCATCTTATACTGAAGTACCTGTGTATGGAAGCAATGGAAGCGACAAACAGAGCAGTAGAAATGGGATTCATTAATCTAGCACAGTTTCCACAACTTCCAGTGCCTGAGATTCTGCATGGCTTGCAGGACCAAGCTATGCAAATTGTATGTTCCTTGTCAAAGGAGACGCAGCAGGGTGAAATT---------------------------------------------------------------------------------------------------------------------------------------------------------------------------------------------------------------------------------------

---------------------------------------------------------------------------------------------------------------------------------------------------------------------------------------------------------------------------------------------------------------------------------------------------------------------------------------------------------------------------------------------------------------------------------GGAAGTACGCAGTATATTCAGATAGTGGAGTGGTTCAGAACTTCTGAAGTTTTCTGGAAACAAATGTCCACCATTCTAGATGTTGTGATTACTGAGGATCCTCCTACTTTGCCTGGAGACGGCGCACTTAGCCTCGCCTGCAAGTACCAGTGTTGTTCACTTGTGCTAGAGATGTTGGCATATGACTTGTTTCTAGAAAAAAAATTAGTAAACGCGGAGTCTCCCATAAAACAGAATTCTGAAAAATCTCGAGCATCTAGTCCAGCTGTTGTGCAGAATATATTGTTGAAATGGTGTAAATCTCCAGCCTTAAAAAGTCTGATAAATTTATATGCATCTTGTGAATACGACTGCGAACTACTTCTCCGTGCAAAGGTCGCTTCAAACATGTTTCTTGTGCATGTGATGGGGAAATTAACTGCTGATGACACTGGGAGTTTGTCTTTGTCATTTTGTCGGAATATTCAGGACATTTCAAAGAAGTTAATTACTCAACCTGCATTTTCCGATCTTCTAGCTCAATATACCCAGCGGGGATACAGTGGCGGCAAGGAACTGAAAACCCTAGTACTTAATGATCTCTACTACCATATGCAAGGCGAGCTAGAAGGTCGTAAAATTAACTCTACACCATTCAAAGAGCTCACTCAGTTTATGTTACAGTTAAGGTTTTTGCACACTGAGCTGAAGTACAGAAGAGACTTTCCTGAACACGCCAGTGATGTATATATATTTGATCTTGTACGCTTGCAAAAAGATTTGGGATACGAGTATTGGGAGCACTCAAACTGGAAGGAATGTACAGTAATTGTTGAAAAGATGATGTCGTACATGCGAGGTGCAAATATGATGTCCTTTCTTGCAAGTTGCAAGCTTTGCGCTTTGGACGCGCTGACAACCATATTATCTGTCCATGGAGAAAATTTAACTGGAACAAAGAACACTTCAGATGGTAAAGTGATTGAGTCGTGCATCGAACTAGTCTGTGAAAAGTTGGAGATAAGTGTGGAGTCATTAATTGCATCTATTGTTCCTTGTGTTGAAGTTATTGATCATGTGGAAGCGCAAACAAGATTGCTTCTCCGTCTGGTCAGATTACTAGTATCTGATAAGGACGAGGCAAATGTTTGTAGTCGTGTCTTAAACACTGTAGGTAGCAGTCTCAGAGTGATTAGCAGCAGCAGCAGCATCGTCATAGCATCTGGGAGGGTTGAGGGAGTCGTGAAACTCTTACTTGCAGTGCTTCTCGTATCAGTTGAAAATAGTATTCTACATGAAAAACAAGGTGAAAGTGTGATGAGGGTGTTGCCTGTTCTGTGCAGCTGTGTCCAGGTTGGAGAGGATTGCTTTAATATTTCGCTGGCCACAATGGACTTGATACTGAATGGTTTCATGACGTGCGATACGTGGTTACCCATCATACAACGGCATCTAAAACTGGATGTTATAATTCACAAGCTCCGAGAGAGAGGAGCTTCTTATACGTGTGTTTCAGTAGTAATGAAATTTCTCTTGAGCCTTGCACGAGCAAGAGGAGGTGCAGAGATGCTCGAGAAAGCCAATTTTTTCCCATCACTTAAAGTGTTGAATAGCTTGTTGTTGGACGAAAAGCCTTTGTTTGACCAAGATGAGAAGCGCCAGAGAGTTTGGGGACTTGGCATGACTGTCGTGACGGCAATGATAGAGTCTCTTGGGCCGTTTTCAGATTTTGTGGACAGCAGTGTGATACCATATTATTTTGGTGAGAAGGCGTTTATACTGTTTCATAATCTTGATTTGGTAAAGTGCAAAACGTCCCTGACGTCTCTGAGGGAAGTAGAGAATACTCTGATGCTGATATGTGCGGTGGCAAAAAGGAAGCGCAAGTTGAATGTGCAACTGAGGGGGACGTGCATCCATATTTTGGCACTAATAAGTAACTGTCATCAACAGCAGATTGTATGTCCTCCGATATTGAAAGAGGAGCTTGAATTGAACGCAAGAGCTTCTTCATTTGTGAATTGCAGCAGCAAGCGTTGTTGTCTTGGCGGCACGAGTTTTTCAGATGCAGTGGCAGTAGAGATGTACAGGATTGCTCATCTTATACTGAAGTATCTGTGTATGGAAGCAAAGGAGGCGGCAAACAGAGCAGAGGAAATGGGATTCATTAATCTAGCACAATTTCCACAACTTCCAGTGCCTGAGATTCTGCATGGCTTGCAGGATCAAGCTATGCAAATTGTATGTTCCATGTCAAAAGAGACGCAGCAGGGTGAAATTAGAGACGTATGCATATTGTTGCTACAAATGTTGGACAAGAGTTTGTATTTGGAATTCTGTGTGGCACAGACTTGTGGAATTAGGCCGGTGCTGGGACGCATTGAGGATTTCACCAAGGAATTCAAAATGTTGATGCGAGTGGCAGAAGAGCATGCGTACTTGAAAGGCTGGATGAAGTCGTCAAGGCAAATAATATCGTATCTACACCCTGCCTTGTTGGAGACACAGGGA

9833 Unigene29333_R-tanguticus Unigene17923_R-bungei

---------------------------------------------------------------------------------------------------------------------------------------------------------------------------------------------------------------------------------------------------------------------------------------------------------------------------------------------------------------------------------------------------------------------------------------------------------------------------------------------------------------------------------------------------------------------------------------------------------------------------------------------------------------------------------------------------AATGCAGCAGAAACCACAGGGTCTTCATGGAGTTCCCACACACCTGATCCTACTCATGTTAAAAACCCTAACATGATGAATAAAACTTCAGGTAATGCTGTCGAAGGTAGAGATTCAGCACATCAAGGTTCCCAGAATACCGACCAATATTATCCTAACAGACAGAACTGGAACATGGAATTTAAGGATGCAACATCTGCTGCACAGGCAGCTGCTGAATCTGCTGAAAGAGCAAGCTTTGCAGCTAGGGCTGCAGCTGAACTT------------------------------TCCAGAGAATCACATGATCAAATGATGCAGGATCAACAGAGATACAGAAATGAAAGAGAGAACGCA---GGAGTGTCTGGCAGCTCACTGGAAAAGGAAGATGGTAGAAGA---------------------------------------------------------------------------------------------------------------------------------------------------AACCATTCTGCTTCATCAGCTGGTAGTGGGAAATTTTCCAGCGAGCCATCCATGCAGGAGCAAAAGTTACACAGGGATGAAAGACACAGTACACAAGGCACATTTGACAGTGCAGTGGAAACTGAAGATAGTACGAGGAGAAAAAATAAGCGTTCTACTTCATCATTGAGTCATAGGTCTGATATTGAAGATGATCTCGAAAGAAAAGATATATACCACAACAAACATTCTTCAGAGGAAAGCAAAATTCAGTCAAGCCACTTTGATGAACAGATCAACAGACCTGGGCATACTGACTCCATGAAAGAACGTATTACAAGGCAACCGAGCAGATCTTCCTCTTCTTTTTCAGATGATAATGGTGATATCATGCAGAGTAGCACTAACGATAACCCTCCTATTGTTTTTGATGAATATGTATGGGATGATGAAAGTACTGGGCATAAAGAAGTTTCTTACGAT------------TCACCACCACGTACCAAATCACCCCCTCAATTTTCGGAAAGTTCGAGTATTTGGAGCCCTAGAAGGGACAAGGCTGAGACTAGTTTCCCATCTGAATTTTCTACAAGAAAGAATGAAACATCATCACATACGTGGAGCCCTGAGCGGAATAGTGGCGGCTCACTCGAGAAATCATCCAGTGGTAACTCAAATCTCTTCACAGACTCAAAAACTGCTTCTGTGTTATTTGAAAGAGAAAATAAGGCATCTTCTCCTTCACAATCAATTGACTTGCCACCTGTAACATTTGATGAATCTGATGGGGTGGAATCTGAGATTGAAGAGGAAGTGGACAATGCCAAAAGCCCAGAACCAACTAGGCATGCCTCATCTTTTCCGATAAAGGATGCCTACGTTCCCTTGAGGAAGCCCTCGTCGTATGTCTCATCTGATGATTCAGACTCGGACAATACATATCCTAATAGAAATCAAGGAAGGCAAGGTAGTGTTCGAGTTGAATCTTTGAAAAAGGATAAGCCATATAGTCCAGTGACTGAAGAGAAACAACCA---ATTAGATCATCAAGGTACTCACTGAATCGGGATCGTAAAAACAGTGATGATTTCGATACACCAGTTTCACAGAATAAAGTAGAAGATTCTGAATTAGCAAGGGACTTTAGCTCTGGAAGTGAAGAGGAGCTTGATTTAGGAGGACTAAGATTGAGAGGTGGTTTACGAAATAAGGGTTACAAACGGCCACCTTATCGCAAGGATACACCCCAACAGACTAAGGAGGACACGAGTGAGCAATCAAAGGTCCCTCATCAGCCTTCCATTTATGAAGATACGAGCTCAAGATATTCTAGAACAACATTTGCGTCA---GACAATGATGATACCAAAGGGATCAAACGCCAA------------------------------------------------------------------------------------------------AGCAGTGGGAGCAGGGTTCATAAAGCTACGAGCTCAAGGACTTCTAGAACAACTTTTGCGCCTGAGAATGTTGATACTGAA

GTTCTTCACAGAAGCTTCAAGCCTGCAAAATGCAAAACATCGTTGAAACTTGCGATGGCTCGATTGAAACTATTGAAGAACAAGAGAGAAGTACAAGTGAGATCGATGAAGAAGGATTTGGCTCATCTTCTCGAAACCGGTCAAGAGAAAACCGCTATGATCCGGGTTGAGCATGTTGTGAGAGAGGAAAAAACAATGGCTGCTTATGATTTCATTGATATATATTGTGAGCTTATTGTAGCCCGCTTGCCGATCATTGAATCGCAGAAAACCTGCCCAATCGACTTGAAAGAGGCCGTGTCTAGCTTAGTGTTTGTATCACCTAGATGTGCAGACATACCAGAACTGGAGGATGTTGGCAAGCAATTCACTGCAAAATACGGAAAAGAGTTTATCACTTCAGCTCTTGAGTTGCGACCTAATTGTGGTGTCAACCGTACGGTGGTCGAAAATTTATCTGCTAGAACACCTGATGGTCAGACCAAACTTAAAATCTTGTCCGAGATTGCCAAAGAACATAATATTGAGTGGGACCCTAAAGCATTTGGAGATAAAGAACTGAAGCCTCCAGATGACCTACTGAATGGTCCAAAAGTAAATGAAGCCACTGGTGGGGGAAAGGCAGAGACCCTTCCAACACATCTTCTTTCGTATGGAGGGACACCTGGACCAACTAATGCAGCAGATACCACAGGGTCTTCATGGGGTTCCCACACAGCTGATCCTACTAATGTTAAATACTCTAACGTGACAAATAAAACTTCAGGTAATGTGGTAGAAGGTAGAGATTCAGCACATCAAGATTCCCAGAATACCAACCAATATTATCCTAACAGACAGAACTGGAACATGGAATTTAAGGATGCAACATCTGCTGCACAGGCAGCTGCTGAATCTGCTGAAAGAGCAAGCTTTGCAGCTAGGGCTGCAGCAGAACTTTCGACCTATGGGAATTTTTCAGGGCAGTATTCCACAGAATCACATGATCAAATGATGCAGGATCAACAGAGATACAGAAATGAGAGAGAGAACGTACAAGGAGTCTCTGGCAGCTCTCTGGAAAAGGAAGATAGTATGAGGAGAAGAAATAAACACTCTGCTTCATCAGTGGGTAGTGGGCACTTTTCTCATGAGCCATTGGTTCAAGAACAAAGGACACAGAGGAATGAGAATAAGAACGTGCAAGGTGTCTCTGACAGCAAGTTGGAAACTGAAGATATTACGAGTAACCATTTTGTTTCATCAGTTGGTAGTGGGAAGTATTCCCGTGAGCCATCCATGCAGGAGCAAAAGTTACACAGGGATGAAAGACAGAGCACGCAAGGCACGTTTGACAATGCAGTGAAAAGTGAAGATATTACGAGGAGAAAAAATAGGCGTTCTTCTTCTTCATTGAGTCATAGATCTGATATTGAAGATGATCTCGAAAGAAAAGATATATACCACAACAAACATTCTTCGGAGGAAAGCAAAATTCAGTTAAACCGCTTTGATGAACAGATCAACAGACCTGAGCGTACTGACTCTGTGAAAGAACGTATTACAAGGCAACCGAGC---AGCTCTTCTTCGTTTTCAGATGATAATGGAGATATCACGCGGAGTAGCAGTAACGATAACCCTCCTACTGTTTTTGATGAATATGTATGGGATGATGAGGGTACTGCGCAGAAAGAAGTTCTCTACGATTCGCCACCACATTCACCACCACGTGTCAAATCACCCCCTCATTTTTCAGAAAATTCAAGTATTCGGAGTTCTAGAAGGGACAAGGCTGAGGCTAATTTCCCTTCTGAATTTTCTACAGGAAGGAATGAAACTTCATCACATACGTGGAGCCCTGGCCGGAATAGTGGCGGCTCACTCGAGAAATCATCCAGTGGTAACTCAAATTCATTCACAAACTCCAAAACCGCTTCTGTGTTATTTGAAAGAGAAAATAAGGCATCTTCTCCTTCACAATCAATTGACTTGCCACCTGTAACGTTTGACGAGTCTGATGGGGTGGAATCTGAGATTGAAGAGGAAGTGGACAATGCCAAAAGCCCAGAATCAACTAGGCATGCC---TCTTTTCCGATGAAGGAGGCCCAATTTCCATCGAGGATGCCCTCATCGAATGTCTCATCTGATGATTCAGACTCTGACTATACATATCCTAAGAGAAATCAAGGAAGGCAAGGTAGTGTTCGAACTGAATCTCTGAAAAAGGATAAGCCATATAGTCCAGTGATTGAAGAGAAACAACCATCTATTAGATCATCGAGGTACTCACTGAATCGGGACCGTAAAAACAGTGATGATTTTGATGTACCAGTTTCACAAAATAAAGTTGAAGATTCTGAATTAGCAGGGGACTTCAGCTCTGGAAGTGAAAAGGAGTTGGATTTAGGAGGACTGAGATTGAGAGGTGGCTTACGAAATAAGGGTTACAAACGGCCATCTTATCGCAAGGATGCATCCCAACAGACTAAGGAGGACTCAAGCGAGCAATCAAAGGTCCATCATCAGCCTTCCATTCATGAAGATACAAGCTCAAGATATTCTAGAACAATATTTGCGTCAGATGATGATGATGATACCGAAGGGATCAAACGCCAAAGCAGTGGGAGCAGGCTTCACAAAGATACGAGCTCAAGAGCTTCTGGAACAACTTTTGCGCCAGAGAATGTCGATACTGACAGTTTAGAACGCCGAAGTAGTGGGAGCAGGGTTCATAAAGATACGAGCTCAAGAACTACTAGAACAACTTTTGCGCCAAAGAATGTTGATACTGAT

9238 Unigene28161_R-tanguticus Unigene7041_R-bungei

GATTGCAGCGCTGGTAGTATCGTTTGGGTTCGCCGCAGAAACGGATCGTGGTGGCCTGGTAAAATAGTCGGCAACCAAGAGCTCTCAGCTTTACATCTCATGTCTCCCAGATCTGGTACTCCGGTCAAGCTTCTTGGCAGAGAAGATGCCAGCGTGGACTGGTACAATCTAGAGAAATCCAAGCGCGTAAAGCCATTTAGGTGCGGCGAGTTTGATGATTGCATTGACAAGGCAGAGCAATCATTGGGAATTCCCATAAAGAAAAGAGAAAAATATGCACGCCGAGAAGATGCTATTCTACACGCCCTTGAGCTTGAAAGGGAGAACGTGGAGAAGAAAGAGGAGAAACAAATGAATGCAAACTCTGCAAGCAGTAAAAAGCTTTCTGTTCTTAAGGAGTTGAGTGTTTCTTCAAAGCACGCAGGGAAAGGTAACTACAAAATTATAAATTCGAAGTCGCATCTGGATTCTTCTATAAAGGAGGAAGTTATCATTCCTGTATATCCTCAAAGGGCTAAACCTGGTAAGCAGTCAAGTGGGAAAGAAGATAAACTTCGGATTAGAGGTTTGCAGGACTTTGATCCCAACAGAAAGCCACCTGTTATTCCAGTGTATAATCATGTTCCAAGTGGGATTCAAAGCTTGGAACGTACAGGTGCATTTAGTAGCAGTAAAAGCCAGGTCACATTCAAGAGGAAAAGAAGTCATGGTGGCTTAGTTGAGGAATCTCTCAGCAAAAGACGTGATAGACGCCGGCCTCTTGTCCAAGTATTACAAACTAGTGCGATACTGGAAGCTGCCCAAAATTTGCAGTTTGTTGAAGACGTGCAAGGAGAAAAGGAGCAGGTAGGATTCATTTGTCGTGCAAAACGGAGCCGATGTGTCTATCTTCCAGCCGAGGCAAATGAGGGGTTGGATCATACAGCAGAACCGCAGAGATCCCCACCCCAGTTCGGGATGGATAACTGCAATATCATCAACCAAGGCTCTTTAACTGAGGAAAATACTACATCTGGATCGACGAGGGACAATGCAGAGTCTGATTCTTCTAGCAGCGACGAAGAGACGGATGATGACCCTGCTTTTCTCTCAGATTGCATTGAAGCACAACCCATGATAGTAGGAACAAGCAATGGTCCCATTATAGGGGGAATAGAAAGCTCGTCAAGTGAAGAAGAGGGCTATCTGTCTCGCTATCATTCTAATAACCAAGCTGCTGATATGGAAGTGTCTAAATGGAAATTGAAGGGGAAACGGAATATTCGCAATAACAGTTACTTGGAAGGAAAGGGGAACTGGTCAAACCAGCTGACGAACCTGAGACCTAGTGTTAGTAGGTTTTATGGTAGTGATTATTTAAGCTATGGTTATGATGAGGATGAAGAAGAGATTGAGAAGAACCCCCCAATGCATAACAACAGTAAGAGAATGACAGACGGGTTTGGATATCTAGATATTATTGATTCA---GAAGAAGAAGATGATCCGCTTTGGGAGGCTGATATAGGTGAGTGCTATGATCCGATATACAGCGGGAAAGGGAAGAAGAATTCTTGCGATTTGGTAGATGTGGAGGTGAAGGTTCAAGCCAGTAGTTATCAAGGAGAGCATGTCCCTTTAGTTTCCTTGATGAGTAGGTTGAATGGGAAAGCAATAATAGGGCATCCCATCCAGATTGAAGTTTTACAAGAAGAGGAAGAAGTTTTTCTATCTGATTTTAGGGAAGAAGCAACTGCCAACGATACGATAGTAGCAGCAGCACCCATGTGGCAGACTGCCAGAAGAACAGCAATGCACAGGATCCCTCGTCCTCATCCTTGTATAACTTTGACTCCAGATGACAACCCCCCTTTACAATTATATCAACGCCCCGTAGAAAGGAAAATCCAGAGAAGACCAAGGAAGAAAGTGAGCAGCAGCGGATTGTCTAGCCAGAAAACAAGAACATTATCGTCTATTGCTATTGAGCAGAGACTATATGGAAAGGTGAGTAGTCCTGGGGCGTTGATAAAACCAGAGAATACTGCTGTCACTTGCATTCCTGTGAAGCTAGTGTTCAGTAGGTTACTTGCAGCAGTGGGTAAGCCGCCATCTAGACCCACCCCAAATCATCCTAGTGTT

------------------------------------------------------------------------------------------------------------------------------------------------------------------------------------------------------------------------------------------------------------------------------------------------------------------------------------------------------------------------------------------------TCTGTTCTGAAGGAGTTGAGTGTTTCTTCAAAGCATGAAGGGAAAGGTAATTACAAAGTTATAAATTCGAAATCACATCTGGATTCTTCTATCAAGGAGGAAGTTATCATTCCTGTATATGCTCAAAGGACTAAACCTGGTAAGCAGTCGAGCGGGAAAGAAGATAAACTTTGGATGAGAGGTTTGCAGGACTTTGATCCCAACAGAAAGCCACCTGTTATTCAAGTGGATAATCATGTCCCGAGGGGGGGTCAAAGATTGGAAGGTACAGGTGCATTTAGTAGCAGTAAAAGCCTGTTCACATTGAAAAGGAAAAGAAGTCACAGCGGCTTAGTTGAGGAATCTCTCAGCAAAAGACGTGATAGACGCCGGCCTCTTGTCCAAGTTTTACAAACTAGTGCAATGCAGGAAGCTGCCCATAATTTGCAGTTTGTTGAAGACGTGCAAGGAGAAAAGGAGCAGGCAGGATTCATTTGTCGTGCAAAACGGAGCCGATGTGTCTATCTTCCGGCTGAGGCAAATGAGGGGTCAGATCATACAGCAGAACTGCAA---TCCCCACCCCGGTTTGGGATCGATAACTGCATAATGATCAACCAAGGCTCTTTAACTGGGGAGAATACTACATCTGGATCGACGAGGGACAATGCAGAGTCTGATTCTTCTAGCAGCGATGAAGAGACGGATGATGACTGTGCTTTCCTCTCAGATTGCATTGAGGCACAGCCCATGATAGTAGGACCAAGCAATGGTCACATTGTAGGGAGAATAGAAAGCTTGTCAAGTGAAGAAGAGGGCTATCTTCCTCGCCATTATTCTAACAACCAAGCTGTTGATATGGAAGTGTCTAAATGGAAATTGAAGGGGAAACGGAATGTTCGCAACAACAGTTACTTGGAAGGAAAGTGGAACTGGTCAAACCAACTGACGAACCTGAGACCTAGTACTAGTAGGTTGCATGGTACTGATTATTTAGGCTATGGATATGATGAGGATGAAGAAGAGATTGAGAAGAACCCCCTAATGCATAGCAACAGTAAGAGAATGACAGACAGATTTGGATATCCAGATATTATTGATTCGGAAGAAGAAGAAGATGATCCGCTTTGGGAGGCTGATGTAGGTGAGTGCTATGATCCGATATACAGCTGGAAAGGGAAGAAGAATTCTTGTTGCTTGGTAGATGTGGAGGTGAAGGTTCAAGCCAGTAGTTATCAAGGAGAGCATGTCCCTTTAGTTTCATTGATGAGTAGGTTGAATGGGAAAGCAATAATAGGGCATCCCATCCAGATTGAAGTTTTGCAA---GAGGAAGAAGTTTTTCTATCCTATTATAGGGAAGAAGCAACTGCCAACGATGCGATAGTAGAAGCAGCACCCATGTGGAGGACTGCCAGAAGAACAGCAATGCTCAGGATCCCTCGTCCTCATTCTTGTACAACTTTGACTCCAGACAACAAACCCCCTTTACAATTATATCGACACCCCGTGGAAAGAAAAATCCAGAGAAGACCAACCAAGAAAGTGAGCAGCGGCGGATTGTCTAACCAGAAAACAAGGAAATTATCATCTATTGCTATTGAGCAGAGACTGTACCGAAAGGTGAGTAATCCTGGAGCATTGAAAAAACCAGAGAATACTGCTGACACTTGCATCCCTGTGAAGCTAGTGTTCAGTAGGTTACTTGCAGCAGTGGGTAAGACGCCCTCTAGACCCACTCCAAATCATCTTAGTGTT

1480 CL4274.Contig1_R-tanguticus Unigene25757_R-bungei

------------------------------------------------------------------------------------------------------------------------------------------------------------------------------------------------------------------------------------------------------------------------------------------------------------------------------------------------------------------------------------------------------------------------------------------------------------------------------------------------------------------------------------------------------------------------------------------------------------------------------------------------------------------------------------------------------------------------------------------------------------------------------------GTTAAGTTAAGCTACGACTTCTTGCATGATGATAAAGCTGCTCAAATGTGCTTCCTATTCTGTTCTCTGTTTCCGGAAGACTATCTGATCAAAACTGATGTATTACTTAGTTACATGCTTGGAGAAGAGTTGTTGGAAAAGGGGGATAACATGGAAGAAGCTAGAAACGGGTTACACACCGTAGTCGACAAGCTGAGGTCGTCCGGAATATTGTTAGAAAGCGAGAAAGGTGATATAATGATGCATGACATTATTCGTGATGCGGCTATATGTATTGCATCTAATTCTGAGGAAGGTGAGGGATTCATTGTGAGATCGGGGTTAGGATTGAAAGAGTGGCCACCTATAGATAGAGAGATGGAAAAGTGCAGGCGATTATCTTTAATGTGCAACGACCTCAGTTTTTGTCCCCTAAACCAAATCACTGCTTCACTCCTCTTAACCCTTTCTATGTTTCAAAACCCAAATTTAGAGGAAATTCCTAGTGATTTTTTCAAGGAAATGAAACACCTCAAGACTCTAGATCTTAGTGAGACTGCTATTAGATCACTCCCTCCATCTTTTCAATGCCTTGAGAGTCTCCACACACTTTATATG---------------------------------------------------------------------------------------------------------------------------------------------------------------------------------------------------------------------------------------------------------------------------------------------------------------------------------------------------------------------------------------------------------------------------------------------------------------------------------------------------------------------------------------------------------------------------------------------------------------------------------------------------------------------------------------------------------------------------------------------------------------------------------------------

TTTGAAGCCTTTGCATCAAGGAGAGATACTACAAAAAGGGTCATGGATGCATTAAAAAATGACCATCTCAATATGATTGGGGTTTTTGGGAAGCCAGGTGTGGGCAAGACCTTTTTAACAGAAAATATTATGAAACAAGTGAGAGATGAGGAATTATTTGATCGAGTTGTGATGGTCATTGTTTCTCGAAATCCCAACATCAGAAGGATTCAGGAAGATATAGCAAAACAATTGGATCTGGATCTTAACATCGGAAGGTTTCAGGAAGATATGGCAAGACATTCGGATCTGCATCCTAAAATCAGAAGGATTCAGGAAGATATGTCTCCTAGAAGATATGCTGCAGAATTGTCACATCACTTAAGTGGGGATAGAAATGTACTTATTGTGTTGGATGACATTTGGGAAAGATTGGACCTCCATGAAGTGGGAATACCATATAATAGCAAAGTTTTGTTCACGACACGAAGTCAAGATGTATGTGATGAAATGAAAACAGAGGAAACGATTGAAGTTGGAGTCTTATCAGAAAATGATTCTTGGGAACTTTTTCAACAAAAAGCAGGTGATGTAGTGGATTCTCCTTCTTACCGAGAAGTAGGTCACGATGTCCTAAACGAATGCAAGGGTTTGCCTTTTTCTATAGTTACACTGGGATTGGCATTGAGAAGCAAAGAGGTGAAAGTGTGGCATGATGCTTACACACAATTGAAGACATCAATATACAGCAATGGTACCGATCCTGCCACCGCTGTAGTTAAGTTAAGCTACGGATTCTTGCATGGTGATAAAGCTGCTCGGATGTGCCTCTTATATTGTTTGCTATTTCCGCAAGGCTTTCGGATCAAAATTAATGTATTACTTACTTACTTGCTCGGAGAAAAGCTACTGGAAGATGCTGAAACCATTGGTAAAGCGAGACAGAGGTTGCACGACATAGTCGACAAGCTGACATCGTCCGGAATATTGTTGAAAAGCAAGGAAGGCGACATAATGATGCATGATATTATTCGCGACACGGCCATATGTATTGCATCTGATCCTAAGGAAGGTGAGGGGTTCATTGTGAAATCTGGGTTAGGACTAGAGGAGTGGCTACCCATAGATCAAGAGATAGAAAACTGCAAACGATTATCATTAATGAGCAACGACCTCAAGTTTATGCCC------AATTTCACTGCATCAAGTCTCTTAATCCTTTCACTGTCTCAAAACAAAAATTTACAAAGAATCCCTGATGACTTTTTCGAGGAGATGAAACAGCTCAGAACTTTAGATCTTAGTAGTACTGGCATTATGTCATTGCCGCAGTCTATCCAGTGCCTTCAGAGTCTCCATACACTTCTTATGGATGAGTGTTTTGATTTAACTGACATTACTCTGGTTGGAGAGTTGCAAAGGCTTGAAATACTCAGCTTTCGTGATTCCAAGGAGGTCCTAATACCAGAAAGCATGCAGAAGCTGGCGAATTTAGCAATACTAAATGCGTCTAACACCAAGAATCTTTTTCCGCCACGAGTAATCTCAAGCCTGTATCGATTAGAGGAATTGTATTTGGGAACTGGTATTGTCAATGATTTTTCAGAGGTTGAATTCTTGGATCGGTTGACTTGCCTGGAAATTCAGTTACCAGTTGAATGTTTGTCGAAAGATTCTCCTAGGCACTTGACGAACTTGTCCAGCTTTTCTATTTCGGTCGGCAAACAAGAGTGGTTCTTTCCTACAGGGCATCAATTGGAGGCAAGAGAAATGTATCTCGATTTGAGTTTATCAACTCAAAATCCCTCAATTCGAATTCCCAATTGGGTTGGGACGTTGTTAGAGAAAACTGAGCATTTGGAAGTAGCCGGAATTCATCATCTTACAACTGTTGCCGAACTGAATGAAAATTGGATGTACCACAGCAGCTTGAGGTTCCTTCGCGTTTCTCAATGTAAACAGCTGAGGCATCTCCTGAGTACAAAAAAAGGTCTATTCAGTACTCTGTGGCATTTCATTAATACAACGGTAAGAAAACTTACTTCTGCAACGACAACGGTAAGAAAACGTCTATTCAGTGACCTTGAAGAATTACATCTCTCAGGCGTGCTGCACTTTATCTCAATATGTGATGGA

2661 CL6998.Contig1_R-tanguticus CL8147.Contig5_R-bungei

---------------------------------------------------------------------------------------------------------------------------------------------------------------------------------------------------------------------------------------------------------------------------------------------------------------------------------------------------------------------------------------------------------------------------------------------------------------------------------------------------------------------------------------------------------------------------------------------------------------------------------------------------------------------------------------------------------------------------------------------------GAGAGTGATGATCTTCAAGGTGATGAAATGGTCAACTTGGGAGCAGATTCTGTAGAGAGGGATGACTCTGCAGATGATTCGTCAGATACAGATATGGAGGATAACCACTCAACACAGGAAGACCATTTTGTTGTAGCTCCAATAGGTAAGGAACACTCTGGTGGAGAGAAGGACATGGTTCATGAAGATGAAACCGAAGATGAAGAGGAAGATGCTCGCCAGTTAGCCCTAGTGAAGGACTGGGTCAATTCTCATCATAACAATTCAATTGGGCTTATTGATGTTCTTTCAAATTTTCCTGACATTTCCATGGCACTGACTGAAGGAATCATGGCCAGACTGGTTGATGACGGAGTAATATCAAGAGCTGGTGGGGACACTTACACCATCAGAAAGAAACAGGATGTTGAGTCACACTTGAGGGATGTGAAGGAAGAGAGGAATGCCCAAGAGAGA---CATGATGACAACATCATAAAGAAGACAGACGAAGATTACATGTATATGAAGGCTTTGTATCATGCTCTTCCAATGGATTATGTGACTGTACCAAAGCTTCATGCGAAACTCAATGGAGAAGCAAAACAGGCAACAATACGAAAATTGGTTGATCAGATGGCCCTGGATGGATATCTCGAGGACAAGAATAACCGCAGACTAGGCAAGCGCGTTGTTCACTCAGATTTGACTGCCAAAAAGCTAGCTGAGGTTCAGAAAGCTTTAGGAAACAACTCTTCGGAAATGGAAATTCAGGAACCACAGAATAGACCTAGTGGTTTAGACCACCTCAAAGAG---------GGGAGCAATGGTAGAGATACATCAACCTGTGGTGGTCTTCACTCAATTGGTTCTGATCTTACACGCACACAAGGAAGATCCCGCATCCAGCAAAATGACTCACTCAGAAGCGAGCAGACTACAGGAATAAAGCAAGAGCAGGGAAACACACCCAATAGCAAACGCGAGCCTGCTCCTTCACGAGAGAGCACTGTACCTGGAATTGAAAGAACTTACTCAGGCGATGGCGGCAATTCTAGGTGTTCCACTCAAGATAAGCGAGCTAGGAGAGCAAGCATGGTCAAGGAACCTATTCTTCAAAACTTGAAGCGCCAGAAGTCTCAAGCT

CTTATGTTTCAGGTTGTCGCACAGAAATTGAAGGAAGCTGAGATTACCGAACAGGATTCGCTGCTTCTGACACGGAATCTGCTTCGAATAGCAATTTTTAACATTAGCTATATCAGAGGGCTATTTCCAGAAAAATACTTCAATGACAAGGCAGTTCCTGCTCTAGACATGAAGATTAAGAAACTTATGCCAATCGATGCTGAGTCAAGAAGATTGATTGACTGGATGGAGAAAGGTGTTTATGATGCCTTGCAAAAGAAGTACCTGAAGACACTTTTATTCTGCATTTGTGAAACCATTGACGGACCAATGATTGAGGAATATTCATTTTCTTTTAGCTATTCCAATTCGGATAGCCAAGAGGTTGCAATGAACAAAAACTGCAGTGGAAACAAAAAGGGAACAACATTCAAGTCTAATGGTGCAACTGATGCTACCCCAAATCAGATGAGGAGTTCTGCTTGTAAAATGGTTCGAACACTGATCCAGCTGATGAGAACTCTTGATAGAATGCCAGAAGAGCGCACTGTTTTGATGAAGCTCCAGTACTACGATGATGTTACGCCAACCGACTATGAACCACCATACTTCAGAAGCTGTTCTGAACAAGAAGCTATTAATCAGTGGACTAAAAATCCCCTTAAAATGGATGTTGGGAATGTAAACAGCAAGCACTTGATTTTGGCTCTGAAGGTCAAGAGCATCTTAGACCCCTGTGGCGATGAGAGTGATGATCTTCATGGTGATGACATGGTTAACTTGAGAGAAGATTATGTAGAGAGGGATGACTCTTCAGATGATTCTTCAGATACAGATATGGAGGTTAACCACTCATCACAGGAAGACCATTTTGTTGTAGCTCCAGTAGGCAAGGAACACTCTAGTGGAGAAAAGGACATGGTTCATGAAGATGAGACTGAAGATGAAGAGGAAGATGCACGCCAGTTAGCGCTAGTGAAGGACTGGATCCATTCTCATCATAATGATTCTTTTGAGCTTACTGACATTCTTTTAAACTTCCCTGACATTTCCATGATATTGATTGAAGAAATCATGGCCAAACTGGTCGATGAAGGGGTAATATCAAGACCTGGTGGGGACACTTACACCATTAGAAAAATACAGGATGTTGAGTCACACTTGAGGGATGTGAAGGAAGAAACGGATGCCCAAGAAGAAGCACATGAGTTCAAAACTGAGAAGAGGACAGATGAAGATTACATGTATATGAAGGCTTTGTATCATGCCCTTCCGATGGATTATGTGACTGTTCCAAAGCTTCATGCGAAACTCGATGGAGAAGCAAAACAGGCAACAGTACGAAAACTTATTGATCAGATGGCCCGGGATGGATATCTTGAGGAAAAGTATACCCGCCGATTAGGCAAGCGCGTTGTTCACTCGGATTTGACTGCCAAAAAGCTAGTCGAGGTTCGGGTTGCTTTGGAAAACAACTGTTCGGACATGGAAATTGAGGAACCACAGAATAGACCTAGTGGTTTAGACCACCTCAAAAAGAGTATTACAGGGAGCAATAATAGAGATACATCAACCTGTGGTGGTCTTCACTCTATTGGTCCGGATCTTACACGCACACAAGAAAGATCGCGCATCCAGAAAAATGGCTCACTCAGAAGCGAACAGACTATAGGAATGCAGCAAGAGCAGGGAAACACGCCTAACAGCAAACGCGAGCCTGCTCCTTCACGAGAGAGCATTGTACCTGGAAATATAAGAACCTACTCA---GATGGCAGCAATACTAGGTGTTCCACTCAAGATAAGAGAGCCAGGAAAGCAAGCACGGTCAAGGAACCTATTCTCCAAAACACGAAACGCCAGAAGTCTCAAGCT

1861 CL6941.Contig2_R-bungei CL5143.Contig4_R-tanguticus

------------------------------------------------------------------------------------------------------------------------------------------------------------------------------------------------------------------------------------------------------------------------------------------------------------------------------------------------------------------------------------------------------------------------------TTTGAGATGATGCACAGAGGTCCAAAGCCAATAAAGGTTGTGGATTCTTTTGTAACTCAGGCGATATCTCCAGGACCAGAGGTATCTCAAAACTTAAGCAATAATGACCGGGAGGATATATCAACACAATATTTAGAGTTTTTGGATCATTCTGGTAGTATCGATGATTTCATGAAAGCTTGGAATCGCCATAGAAGGTTGTTTCCCCATTTGGTGAGGCCTACTTCATTTGATTACAGAACTGTACAGGATGTCATCAGCCTATTGCCTCGTGCATCGGATAATGAAAAGGTCCAGGAGTCACTCCAGGCATTACCGTTAGACCAGAAATCATCACTGCCTGAAGATCCAGACAAAGAACTTGATCAT------ACTACCCACACCCAGATTGTAGATACTGATAATACAAATGAAAGACTGCAAGTGGTATCATCTCCCATGGTTGTGGAACAATCCAGGCAG------------------GTAGAGGCCAATGAAATGGTGGATGAAATTTCTCTGGTTACTCCACAAGAATATACGGAAGGGGCTAGCAAAGTTCACAGAAAATCGGATCCAAATTGCAATGATGATTCCAGGCTACCATCACTGGCGTCCCTTTCATTAAATCCTCCAAACATTGAAATTCAGGATTCTATCCCAACCACCTCTCACGGCAGCGGAATTCTTCAAGAAACGCCTAGTTCGAACAAAAACAGACAGCATGATGAAATATCTTTGTCAAGTCTGACATGCCCAAAGTGTGGAAGCGAAGTTCGCGTGCATCCAGATTATGGTCGTGGACACATGCCCAGTAATAATCAAAGTTCACAAGCAGTGAATCGACATTCACAACCATTACCACCTACAAATCGTGCGCCGACTCGTTATGTCAATACAAAAAATAGATATAGGCCGCCGAATTATTCTGAATCTCAGCATCCTAAACGATTGCAACAGCATCCACCGTCTTCAGTGCATCCCCAGGCCCTTGGTAATCAAAATTTTCCTCATCAGCCTCAGACATGGCAAAACCCTCCATTGCAACCAAGCAACCCTGCAGCAAACCAGTATCAAATGCCTGCTGTGCAGATGCCGCAAATGAGTTCCAATCAGTGGACAGGCCAAACCATGCAGCCACAATATTTTGCACCTAAT---------CCTCAGCTTCAACCTGTTGTGTCCCAACCTGTTGTTTATCCTCAGGTGCAAGCTTATCAATATCCAGTGCAAAATAATGTCCAGTATGCATATATGCAGGGCAACCAGGTTCTTACTCCTGAGATGTGGCATTATTATTATGGACAACAGCAGCAGCTGCAGCAGCAGCAACCACAACTAGTACAGCAATTTGCCACAGTACAAACACAACCGTGGGACAATAATTACTAT---CAGCAGGCTGTTGTGCAACACTATGCTACCAATCTGTATGGGCAGGAGTATGTTCAACAGGCAGTTCAATCCCAGGGACAAGGAACCACTCAACAAAAGATTTCCATTGTGCAGGGACAAGGAACCACTCCACAAAACATCGAAGTGGCTGGACCACCATTGCCATCGTATCCGGAAATCCCGCAGCCACCTGCGCCACATGATGAAAGCACTCAGTATGGGCAGGAG------------------------------------------------------------------------------------------------------------------------------------------------------------------------------------------------------------------------------------------------------------------------------------------------------------------------------------------------------------------------------------------------------------------

GAGATAGCAAACTCAGCACTCATTCGAGCAACACAAATTTTTCTCAAGAGAGTACCAGCTATCCATACGTTCTGTGCTAGGCTCAAGGAGAAAATAGGTGATGCATTTGGCAGCCGTGCTGCATATCGTCAATGTGACAACTCAGAGTTTGATTCTGATATCATTGAAAATGTTAAAAGTGAAGCTAACATGGAGAAACGTCTGGGGAATTTTGAAGCAGGTATGCACATATATGAAGAAGCACTTCAAAGAGCAATAGAGAAGCGAAAGTATCATATCTTTTCCATGCTTTCGGTTCATTTTTTTCGATATAAATACATGGTCACTGGGGATGTAGAAGCCAGTAGAGAGATCTTGATCAAGGGCATTCAACATTCCCCTCAGAGCAAACTACTCTTACAGGAGATATTGACCTTTGAGATGATGCACAGAGGTCCAAAGCCAATAAAGGTAGTAGATTCTTTTGTAACTCAGGCAATATGTCCAGGACCAGAGGTATCTCAAAGCTTAAGCACTAATGACCGAGAGGATATATCAACGCAATATTTAGAGTTTTTGGATCATTGTGGTACTATCAATGATTTCATGAAAGCTTGGAATCGCCATAGAAGGATGTTTCCCCATTTGGTGAGGCCTACTTCATTTGATCACAGAACAGGACAAGATATCATCAGCCAGTTGCCTCGTGCGTCTGATAGTGAAAAGGTTCAGGATTCACTCCAGGCATTACAGTTAGACAAGAAATCATCACTGCCTGAAGATCCAGATAGAGAGCCTGATCATGTTCCTACTACCAAGACCCAGATTGTAGATACTCATAGTACAAATGAAAGACTGCAAGTGGTATCATCTCCCATGGTTGTGGAACAGTCCGGGCAGGCTACGAAAAAACATAATGCAGAGGCCAATGAAATGGTGGATGAAATTTCTCTGGTTGCTTCACAAGAATATTCGGAAGGGGCAAACAAAGTTCGCAGAAAATCGGATCCAAATTGCAATGATGATTCGAGGCTACCATCACTGGCGTCCCTTTCATTAAATCCCCGAAACATTGAAACTCACGATTCTATTCCAACCACCTCTCGCGGCCGCGAAACTCTTCAAGAAACGGCTAGTTCGAACAGAAACAGACAGCACGATGAAATATCTTTGTCAAGTCTGACATGCCCAAAGTGTGGCTGTGAAGTTCGTGTGCATCCAGATTATGGCCGTGGACAGATGCCCAGTAATAATCAAAGTTCGCTAGAAGTGAATCGACATTCGCAACCACTACCACACACAAATCGTGCACCAAATCGTTTTGTCAATACAAAAAATAGATATAGGCCGCCGAACTATTCTGAATCTCAGCATCCTAAACGATTGCAACAGTATCCACCGTCTTCAGTGCATCCCCAGGCCCTTGGTAATCAAAATTTTCCTCATCAACCTCAGACATGGCAAACCCCGCCATTGCAACCAAACAACCCTGCTGCAAACCAGTATCAAGTGCCTGCTGTGCAGATGCCGCAAATGAATTCCAATCAGTGGGCGGGCCAAACCATGCAGCCACAATATTTTGCACCTAATCCTCAGCCTCCTCAGCTTCAACCTGTTGTGTCCCAACCTGTTGCTTATCCTCAGGCGCAAGCTTATCAATATCCAGTGCAAAATAATGTCCAGTATGCATATATGCAGGGCAACCAGATTCTTACGCCTGAGATGTGGCATTATTATTACGGACAA------------CAGCAGCAGCAACCACAAGTAGTGCAGCAATTTGCCACAGTACAAACACAACCGTGGGACAACAGTTACTATCAGCAGCAGGCTGTTGTGCAACACTATGCTATCAATCCGTATGGGCAGGAGTATGTTCAACAGGCAGTTCAATCCCAGGGACAAGGTACCACTCCACAAAACACGTCCATTGTGCAGGGACACGAAACCACTCCACAAAACACTGAAGCATCTGGACCACCATTACCCTCGTATCCAGAGATCCCACAGCCTCCTGCACCACATGATGAAAGCACTCAGTATGGACAGGAGTATGGTCAACATACACGTCAATCCCATGAAGAAGGAACTAGTCAACAAACTATTGAAGCTTCTGGATCAGCGGTGCCCTTGTATCCAGAAACTCAGCAGCCTCCTGTGCCACACGATGATAGCAGTCAGTATGGGCAAGAGTATGGTCAACATGCACTTCACTTGCATGAACAAGAAACCACTCCACAGAAGATAGAACCATCTATACCATCGGCCCCCTCATATCCAGAAAACCAGCAGCCTCCTGCACCACAACTTGAAAGCAGTCAGTGTCGACCGAAGGAGTATGGTGAACATGCTCTTCAATCTCAAGGGGGAGAAACCACTGAACAAAACATCAATTTATCTGGACTGTCCTTGTCTCCATGTCCACAACAACAGCAGTCTCCCATGCAAATGGAA

8227 Unigene26216_R-tanguticus Unigene28957_R-bungei

TGTCAGAACCAAAATGCAGAGCTGAAGAAAGAACTCTTGAGTGTGAAAACACTCCTAGAGGGTTCCCAAATATCTCTTCACGCGCAGGAGAAGCTATATCAAAAGGCAGAAACAGATATTAACGAAATGCATTCAATAAACATAAATTTGAATGTGCTCTCGTCGGTACTGCAGGAATCTTTGGGTGAGGCAAGCAAAGGCATTGGTATCATGAAGGAACAGATGGATGCACTTGGCCTGAAGCTATTTCATTCAACTAACTCTGAGGAGTTGCTGAAGCTCAGTCTGCAAACCGCACTGGAAGATGTAAATACACTGAGAGTGTAC---AACAACTGGAGCACCAAATGTGATGAGCTAACATTGATGAATCAGATTTTGGAGGAGAAAGCTTGTAGCGTGTCGAATGAAAATGACTCTCTTACTCAAAAGATAACAGAAATGGAAAGGAGGGCAGATGAATATAGAGGATTCGAAAGGAGATACAAAGACTGCACTGAAGAAAAAAATGAACTGCTAAATTTACTAAAGAAGGAAACATCGAGAACATGTAGTTTTCAAGATGAAGTTAGCTCCTTGCACGAGGAGTTGATAACAGTGAAGGCCGCTGCCGATGAGCAGTTCTCAGTTAACAGGAACCTGAAAGAAACCCTCTCTCTTCTGCAGGATAAGTTGGTGAATCTGCGGTCAGCAATGTTATTCTTTGACGACCACATCAGTGAACAAAGCTCAAGCGTTCCTTCTCTTCATGATACAGAAAATCAGGACTTAGTTGGTATTGTCGGTCATTTGGAAGAGCTGCAGCATAAAGCGTGTGCAAAGATACAACAACTCACTCTCGAGAAGAAGGATGTGGAAGAACAAAGGGATACTGCTCAAGGAGTATTGACTGACATTGAATCAGAAAATCTGTCAATAAAGAAGAGGTGTGAAATTGATGTAAAAAGAATGGCAAACCGGCTAGAAATGACTACTGGCCTTGTGGATAAGCTTCAGCTAGAACTTGAAACTATTGCTAATAAACTTAAGATCAGCTTGGAGTCAGAAGAAAGTTATTCAAATCAATTGGAAGAGCTATCATCAAAACTTTCAGTTTTCGAAGTTGAGATGCAGAATGTTACTAACGAGAACAGAGATCTTGCTAAAAAAATCACAGGTTTAGAGTGTATCAGTGAGGAGCTTGACAGGACCAAGTTAGCTGTCATTGAATCTGCGCGTGAGAACCAAGCACTGTTGCTGTCGTTTCAGTCTGGTAATGAAGAATCTGTCCTTCTATCAAATGAACTTGGCATTTTGAGTGAAAAGTTAAGGCACATGACTGATGAAGTGAATTCTGAGAAGATCTTGAGAGTTGAGCTGGAGGGAACTATTGCAGATCTTACGTCTGAGCTGAAGATGAAAAATGACATTCTGATCTCCTTTGATGATGTGAAAATTGAGTTGGTCCATCTCAAGCAGCTGGTGTCAGATTTAGAACTAGAGAAATCAAGAGTGTGCGATCTTCTATTAGAAAGTGAAGAGTCCCAGAGAAAGGCTGATGAAGATGCTTCATTCTATCGTCACCAAGTTACTGATCTGGAAGCTCATTTAAGTGTCTCACTAGAACATCTTCTTTCCACGGATGTTGAACTCATTTGCACGAGAAATCAGTACCAGAGTAGGATACATGAGCTTGTTCAGCAACTTGAATCTATAGACGGGTGCTACAGGGAGCTTCATTTGAAACATCTTCGTGTATTAACGACACTGAATGGCCGTATTTCTAGTGAAGCACATTATGTTAATGAAAAT------------------------------------------------------------

TGTCAGAACCAAAATGCAGAGCTGAAGAAAGAACTCTTGAGCGTGAAAAAACTCTTAGAGGGTTCCCAAATATCTCTTCACGCGCAGGAGGGGCTTTATCAAAAAGCAGAAACAGAACTTAAGGAAATGCATTTAATAAACATAAATTTGAATGTTTTCTCAAAGGTACTGCAGGAATCTTTGGGTGAGGCAAGCAATGGCATTTGTATCATGAAGGAACAACTGGATGCACTTGGACTGAAGCTATCTCATTCAACTGAATCAGAGGAGTTGCTGAATCTCAGACTGCAAACCGCACTGGAGGATGTAAATACACTCAGGGTGTACAACAACAACTGGAGCACCAAATGTGATGAGCTAACATTGCAGAATCAAATTTTGGAGGAGAAAGTTAATAGCATGTCGAATGAAAGTGACTCTCTTACTCAAAAGATAACAAAAATGGGAAGGATGGCAGATGAATATAGAGGATTTGAAAGGAAATACAAAGACTGCACTGAAGAAAAAAATGAACTGCTAAATTTACTAAAGAAGGAAACGTCGAGAACATGTAGTTTTCAAGATGAAGTTAGCTCCTTGCACGAGGAGTTGAAAACAGTGAAGGCCGCTGCCGATGAGCAGTTCTCAGTCAACAGGAACCTGAAAGAAACCCTCTCCCTTCTGCAGGATAAGTTGGTGCATCTGCGGTCAGCAATGTTATTAATTGACGACCACATCAGGGAACAAAGTTCAAGTGTGACTTCTCTTCATGATACAGAAAATAAGGACTTAATTGGTATTGTCGTTCATTTGGAAGAGCTGCAGCAGATAGCGTGTGCAAAGATACAACAACTCACTCTCGAGAAGAAGGATATGGAAGAACAACGGGATACTGCTCAAGGAGTATTGACAGGCATTGAATCAGAAAATCTGTCAATAAAGAAGATGTGTGAAACAGATGTAAAAAGAATGGCAAGCCAGCTAGAAACGTCTACCGGCCTTGTGGTTAAGCTTCAGGTAGAACTTGAAACTATTGCTAATAAACTTAAGATCAGCTTGGAGTCAGAAGAAAATTATGCAGATCAATTGGAAGAGCTATCATCAAAACTTTCAGTTTTCGAAGTTGAGATGCAGAATGTTACTAACCAGAACAGAGATCTTGCTAAAAAAATCACGGGTTTAGAATGTATCAGCGAGGAGCTTGACAGGACCAAGATAACTGTCATTGAATCTGCGCGTGAGAACCAAGCGTTGTTGCTGTCATTTCAGTCTGGTAATGAAGAAACTGTTCTTCTATCAAATGAACTTGGCATTTTGAGTGAGAAGTTAAGGCACGTGACTGATGAAGTTAATTTTGAGAAGATCTTGAGAGTTGAACTGGAGGGCACCATTGCAGATCTTACGTCTGAGCTGAAGATGAAAAATGACATTCTGATCTCCTTGGATGATGTGAAAACTGAGGTGGTCCATCTCAAGCAGCTGGTGTCAGATTTAGAACTAGAGAAATCAAGAGTATGCAGTCTTTTATTAGAAAGTGAAGATTCCCTTAGAAAGGCTGATGAAGATGCTTCATTCTATCGTCTCCAAATTACTGATCTGGAAGCTCATTTAAGTGCCTCACAAGAAGATTTCTTATCCACAGATGTTGAACTCATTTGCACGAGAAATCAGTACCAGAGTAGGATACATGAGCTTGTTCAGCAACTTGAATCAATAGATGGGTGCTACAGGGAGCTTCATTTGAAACATCTTCGCGTATTAACGACACTGAATGGCCGTATTTCTAGTGAAGCACAATATGTTGATGAAAATGCTCGGTTACTGACGGCCTTAAACTCTCTTAAGGCTGAGTTGCAAGTTTTTGCTAGTGAT

1739 CL4873.Contig3_R-tanguticus CL2092.Contig2_R-bungei

AAGAGATCTAAGGTTTTAAAAGGTAGGATATGTGAGTTGCCGGATGATGTTCTAGCGCTTACTCTATCCTTCTTGCCTATAAGAGAAGCTTCGAGGACAAGTCTCCTCTCATGCAGATGGAGAGATCTTTGGAAGGATTCAATGAAGTTTTCTTCTAGTCTAACTCTTGACGAATTCAGCATGACTGGCAATACATATACCCATGGAGAATTTGGCGAAGAAGGTAGCCCTCTGGAAACTGAACAGTATAAATTTGTAGGATGGGTTGACCTGATGTTGCAGCTGGGTTATGCTCCCGTAATAGACTTGTTCAGACTCCGTTTTCCTATGACAAAGGACTTTGCTCATCATATTGATAAATGGATAAAGGTGGCGGTAACGAAGAGAGCACAAAGATATCACATTGATTTGTCTGGGTTCAATACAATTATATATATGTTCGATTTTCCGTTTGAAAATCTCTATACTTTTCCTGATTGGCTATTCACCCAGGAATCTGGACCCTCGGTAGTGTCATTACATTTGACATCTTGCGCG---TTACGGTGTCTTGGTTTCAATTTCTTTAGCTCCTTGGCAGATCTCCAGTTGCATAGTGTGCTTTTAGACCAGGGCGTTATTGAAAAATTCTTGTTGGAGTGCCCTAATATCGAAAAACTGGTCCTCCTCAATTGCTGGAAGCTTCATAAGTTATCAATTTCCGGACCATCCCTCAAACTAAAGCAATTGAAAATAGTCAGATGTCAAACACTTTGTGACATTGAGATTAAAGCCAGAAATCTCACCAAATTTGAGTATTGTGGTAGACAAGTGCAGATTTCATTTGTTGATGCTCGCGTGCTTTCCGATTTTGTTATATGCGCCTTCACTCGC---------CAAGATGAAGATTTTCCAATAAATTATGCTCTCAGTAAACTATCAAGCGATCTTCTGCAATTAGAAATGTTGTTTCTGGAAGCGGCACCGCTGCGGGAAAATAAGATTCCCAGGCGGTTGCCCATGTTTGAGAAACTCAAGAAGTTGGGGGTGCTGATTACAGGTTCAGAAGTAGAAGAAAGTCTTTGGGGATTCATTCACCTTCTCCACGCTTCTCCGTGCTTACATATGCTTGAGTTACATTTGTCTACTCCTGAGGAGTACAAAAAACAAAAGAGGCCTCTCGATGTTGCTCATCTCCACTTGAAGGAAATTGTGGTTTCTGGGTTTAAAGGTGATTCACACGAGATAGAGTTGATGAGATACTTACTTTCGAATTGTGTGGCTCTGAAAAAAGTAACTCTCGATGAATGTGATAAGATATATGACTACATG

------------------------------------------------------------------------------------------------------------------------------------------------------------------------------------------------------------------------------------------------------------------------------------------------------------------------------------------------------------------------------------------------------CAAAGATACGACATTGATTTGTCTGGGTTCCATACAATTAGATATGTATTTGATTTTCCGCTTGAAAGTTTTTATACTTTTCCTGATTGGCTATTTACCCAGGAAACTGGACCCTCAGTAATGTGCTTACATCTGAAATCTTGCGCATTATTAGGGTGTTCTGGTTTCAATTTCTTTAGCTCCTTGACAGATCTCCAGTTGCAAAGTGTTCTTTTAGACCCGGGCAGTATTGAAAAATTCTTGTTGGATTGCCCTAACATCGAAAATCTGGCCCTTGTCCATTGCTGGAAGCTTCATAAATTATCAATTTCTGGCCCATCGCTCAAACTAAAGCAGTTGAAAATAGTCAGATGTCGAACGCTTCGTTACATTGAGATTAAAGCCAGAAATCTCACCAAATTTGAGTATTATGGTGGACAAGTGCAGTTTTCATTTGTTGATGCTCGCATGCTTTCTAATTTTGTTATATGTGCGCGCTCTGAAGGCCAAGATAATGATGATGATTTTGCTATAAATTATGCTGTCACAAAATTATCAAGCGATCTTCCGCAATTACAAATGCTGTTTCTGGATGGGGAATCGCTGCGGGAAAATAAGATCCCCAGACAGTTGCCCATGTTTGAGAATCTCAAGAAGTTGGGGATGCATGTTTCAGGCTCTAGA---GAAGAAAGTCTTTGGGGATTCATTCGCCTTCTCCAGGCTTCTCCGTGCTTACAGATGCTTGAGTTACATTTGTCTACTCCTGAAGAGTACAAAAAGCAAAAGAGGCCTCACGATGTTGCTCATCTCCACTTGAAGGAAATTGTGGTTTCTGGGTTCCAATGTGATTCTCACGAGACAGAGGTGATGAGATACTTGCTTTCGAATTGTGTGGCTTTGCAGAAAGTAACTCTCGATGAAGGGGATAAG---------------

1568 CL4496.Contig5_R-tanguticus CL6892.Contig2_R-bungei

ACTACTCTGAAGATTATGAGCTATAATGTTTGGTCTCAAGAAAAATGTGAGTTGACCAGGAGGATGGAAGCAATTGGTGAACTTGTTGATGAGCATTCTCCACACTTCATTTGCTTCCAGGAAATTACATCATCTATCTACAAATATTTTAGCCAATCAAGCTGGTGGGAAAGTTACCACTGTTCCATGTCACTTGAAGAAGCCAACAACAAGCAATATTTTTGCATCTTGTTGAGCATATTCCCTCCTGTCAATATCATCAAGGCACCATTGGGCTATATTTCAAAAGTAGGGAGAGAAATTAGTTTAATGGAAACCAGAGTTTGGGGCAACAAGTCACTGGTAGTTGCAACTAGCCATCTCAAAAGCCGCTACACTGACCCAAGTGTTCCTAATGGCCAATATCATCAGAATAGGAAGGAACGTATCGATCAGGCGAAAGAGGCGCTTACCATTCTCAACAAATACCCAAATGTCATTTTCTGTGGTGACATGAATTGGGATGAGAATAAGGATGGTGAGTTCCCCCTGCCAGTGGGATGGGTCGATGCATGGAGTGAATTGAGGCCATATGAGAAAGGGTTCACTTATGATACAAAGACGAACGCCATGTTGTTGGGTCATCCACGAGTGAGGAAGAGATTTGATCGAAGCTTGTGCAATTTGACTAGTTTCAGATTGGATCAAATCGAAATTATAGGTAAGGATGCCATACCAGAAGTCATTTACAAATCGAAGAAAAAGGAACTTCCGGTTCTACCTAGTGATCACTATGGATTGATCTTAACAATCTGTAAC

ACTACTCTGAAGATTATGAGCTATAATGTTTGGTCGCAAGAAAATTGTGAGTTGGACAGAAGGATGGAAGCAATTGGTAAACTTGTTGATGAGCATTCTCCACACTTCATTTGCTTCCAGGAAATCACACCAGCTATCTTCAACTATTTCAGCCAATCAAACTGGTGGAAAAATTACTATTGTTCCATGTCACATGAAGACGCCAAGAACAAGCAATATTTTTGCATCTTGTTAAGCAAATTCCACCCTGAGAATACCACGAGGAAACCATTGGGCTATATATCAAGAGTTGGGAGAGAAATTAGCTTAATTGAAGTCATAGTG---GGCAACGAGTTACTGGTAGTAGCGACTAGCCATCTCAAAAGCCGCTGTGCTGACCCAAGTGTTCCTAATGGCCAATATCATCAGAATGGGAAGGAACGTATGGAACAGGCAAAACAGGCGCTTATCATTCTCGAAAACTATCCAAATGTTATTTTCTGTGGTGACATGAATTGGGATGAGGATAAGGACGGCAAGTTTCCCCTGCCAAGGGGATGGGTCGATGCATGGAATCAATTGAGGCCAGATGAGAAAGGGTTCACTTATGATACAAAGACGAATCCAATGTTGTTGGGTCGTCTACCAGTGAGGAAGAGATTTGATCGAAGCTTGTGCAAATCGAATAGTTTCATACCGAATCGAATCGAAATAATAGGTAAGGATGCAATACCTGAAGCAACATTCAAATCGAAGAAGAAGGAGCTCCCTGTTTTACCTAGCGATCACTATGGATTGATCTTAACAATCTGT---

2767 CL7234.Contig2_R-tanguticus Unigene22726_R-bungei

AAGGGATTGAAGAAAAAAGCGGTGCAAGCCTGTAAAGAATTAGTGGGTGCATCTGAAACAACAGATCAAGAGGAAGTTGGTGTGACTCATTCAAAAAAGAGAAGCAAGGAAAAGAGGGATGATGGTCAGGAAAACAAGAATTTGTTGGACAACGGCCGTGTTTCAGATATAGGAATTACTAAAAGTCTCGAACCGGATAACTCCTTTCCAGAAAATTCAGAACATTCTTTGATTAGCAATATGTCGAGTAAGGAAGCTCTTTCAGATGATAAGCATACTGTACAGCATTCACTGCACCCGTCCGACTGTGAAAATGATAATCTAACTCATATACCCAATGTATGTAATGAGACTAAGTCACCCGAAGTTCTCAAAACATACCGGAGGAAGGATAAAAATCTCTTCCCCTTGGCTAATAACTCGGAATACATAATATTTAAGGATCGGCTTGAGGTTTCTTCTAACTGTCTGATTACTTTGGAAAAGAAAGATGAAATTAACTCTAAGAACAAGAATGAGTTATATATAAGTCCAAATGTTCCAGATCCATCGTATAACAAGCACCCCGAGGAAGATAAATTGGGTCAGACATTAAAGATCTCCCCGGATGCTAATATTGAACAGGAACATCAACCAGTTCCAGATCAAGAAAATGATTCTGCTACGTGCTCCAATCAGTTACCCCAGGAG---GTAGGTTATTCAAGCAAGTCTTGTGAAGATTGTCCTAACAAGAGATCTGCATCAAAGAAGACGAGTAGAAAAGATAAGACGAGGTCTACTTCTGATCATTCTTCCGAGCGTCCGAATTCTCCTTCTCGTGAAGGAACTACCACATCCTCTTTAAATGAATGCCAAATGGAGGCAATAGATCCTTCAGTTGAAGGTGTTAGCAAAGATGCTGCGACGGTAGGGAATGAGATCGTAAAGGATGTACTTCAAAAGAGATCTGCTTCTCCTGATATAACACATACGACTCAGTTGCGCAGGAAGCTTCTTATTCTTGATCTTAATGGCTTGCTTGTTGATATAGTTTCTTCTCTTCCTGAGGGCTATAAAGCTGACACGTGGATAGCAGGCAAAGCACTTGTAAAAAGGCCATTCTGTGATGACTTCTTGAAGTTCTGCTTTGAGACATTCGATGTTGGTGTTTGGTCATCAAGGACTAGGAGAAATGTAGACACTGTTGTTGATTTTATCATGGGCGATCTAAGACATAAACTGCTATTTTGTTGGGACCAATACTTCTGCACTGACACGGGGTTCAGTACAGTTGAGGAATATCACAAACCTATGGTCCTAAAGGAGCTAAGGAAAGTCTGGCGTAAGTTTGGGGAGTATAATGAATCTAATACGGTTTTGTTAGATGATTCTCCATACAAAGCACTGATTAACCCTCCACACACTGCGATCTTTCCTTGTTCATATGAGTTTCGGCAAAAAAATGATAATGCATTAGGACCTGGTGGCAATATCCGAACATATTTGGAAAATTTAGCAGCGGCCCCTCATGTTCAGAAATTTATAGAGCAAAACCCATTTGGGCACCCTTTTGGGCAGCGTGCCGTGACAAAGAAGAATACATCTTGGGGTTACTACCTCAGGGTTATC

------------------------------------------ATTGGTGCATCCGAAACAACAGATAAAGAGGAAGTGGGTGTGACTCATTCAAAAAAGAGAAGCAAGGAAAAGAGGCGTGATGGTCCGAAATATGATAATTTCTTGGACAACGGCTGTGTTTCAGACATAACAACTGCCAAACAACTCGAACCAGATAGCTCATTTCCAGAAAATACAGAACATTCCTTGATTAGCAATATGTCAAGTAAGGAAGCCCTTTCAGATGATAAGCATGCTGTACGGCAGTCACTGCACCCACCCAACTGTGAAAATGATAATTTAACTCGTACACCCAATGTATGTAATGAGACTAAGTCACCCGTAGGCCTCAAAACATACCGGAGGAAGGATAAAAACTCCTGCCCCTTGGCTAATAACTCGGAATCCAGTATATCTAAGGACCAGCTTGAGGATTCTGCCAACGGTCCGATTACTTTGGAAAAGAAAGATAAAATTAACTCAAGGAACAAGAATGAGTTAAATATAAGTCCAAATGTTCAACATTCACCGTATAACAAGTATCCTGAGGAAGATAGATTGGGTCAGACATTAAACATCTCCCCAGATGCTAATATTGAACGGGAACACGAACCAGTTCCAGATCAAGAGAATGATTCTGCTACTTGTTCCAATCAGTTACCACAGGAGAAGGTGAGCTCTTCAAGCAAGTCTTGTGAAGATTGTCCTAACAAGAGATCTGCATCAAAGAAGATACCTAGAAAAGACGAGAGGAGATCTATGTCTGATCCTTCTTCCAAGTGTCCATTTTCTCCTTCCCGTGAAGGAACCACCACATCCCCTTTAAATGAATTCCAAATGGAGGCAGTAGATCCTTCAGTTGAAGGTCTTAGCAAAGATGCTGCAGCGGTAGGGAATGAAATCGAAAATGATGTACATCAAAAGAGATCTGCTTCTCCTGAAATAATACATACGACTCAGTTGCGCAGGAAGCTTCTTATTCTTGATCTTAATGGTTTGCTTGTTGATATAGTTTCTTCTCTTCCTGAGGGCTATCAAGCTGACACGTGGATAGCAAGAAAAGCACTTATAAAAAGGCCATTCTGTGATGACTTCTTGAAGTTCTGCTTTGAGACATTCGATGTTGGGGTTTGGTCATCAAGGACTAGGAGAAATGTAGACACTGTTATAGATTTTATCATGGGTGATCTAAGACATAAACTGCTATTTTGTTGGGACCAATACCACTGCACTGACACGGGCTTTAGCACAGTTGAGGAATATCACAAGCCTATGGTCCTAAAGGAGTTAAGGAAAGTCTGGCAAAAG------------------------------------------------------------------------------------------------------------------------------------------------------------------------------------------------------------------------------------------------------------------------------------------

9539 CL6031.Contig2_R-bungei Unigene28706_R-tanguticus

TCTAAAGAAGATGACTCCATTACAAAGACACGGAAACCATACACGATTACAAAACAAAGAGAACGCTGGTCAATAGAGGAGCATAATAGGTTCCTAGAAGCTATTAAGCTATACGGCAGAGCATGGCAGAAGATAGAAGAACACATTGGGACAAAAACTGCTGTTCAGATCAGAAGTCACGCACAGAAATTTTTCTCAAAGTTGGAAAAGGAGGCTCTCCTTAAAGGTATGCCGCTTGGACAAGCTTGTGAAATAGATATCCCTCCTCCCCGTCCTAAAAGAAAACCAAACAATCCATATCCTCGGAAGACCAGCTCAGACTCTCCTGGCTCTGAATCTGTAGGACTGAAGGATGGGAGTTTTTCATTATCAACTTCTTCTTCACACTCCACTAAACATACTCAGGACCTGGAGAATGACTTGCATCTTGAGAAACCTTGTAAGAAAACAAGTGGAAAAGAATTTTTTGACTTGGACAGTAATAGCTCGGGCATCCTTAACCTTTCCCAAGAGACTTCATGTATATCCTTGTCTCCATCAATTAAAAGT---TCAAAAATTGTGGAACTCAAAGCCCAATCAATTTTCAGAGATTCTGCCCTCTTGGTGAAAGAAGAAAGTAGTGAAGGTGGTAGGGCAGATAAATACCTTCTTGACACGGATGCTGAGGAAACTCACAGAATCAATGCCATCTGTACAGACCTCGATGATGGATTGGGTGGAACCCCAATCTCAAGTAACTCCTCCCGCAAATATTTGGATGGGGAAGTGAATAGAGGAAATAGACTAAAGCAGCAAGAAGATCTCTGTCAATCAAATCAGAATTATCCAAGGCACGTACCCATCCATGTATTGGAAAGCAGCACAGAAGTG---------AGTCCCGCCCCCACCATTCATCAACTCGAAGGTACTGAGAATTCAGAGCCATCTACGTGTGTCATTACTGGAAGTCACAGCAACATATCCACTCAGCAGCCGTCGCCTCTTAATCCATCCATCACCAACAATCAAGAAACCTACAACTTGTCAAATTTTTCAAATCTTATTATATCTGCTCTTCTGCAGAACCCAGCTGCCCATGCTGCAGCAAGTTTTGCAGCTTCTTTGTGGCCTTTTACGAATGTCAAAACTACAACTGAAGGTGCAGACCCGAGTACATCAGCAATTGCTGCAGCTACAGTAATGGCAGCTTCTGCATGGTGGGCTGCTCATGGGTTGCTGCCTTTGTGCCCTCCTCTTCAGTCTCCTGCTTTTACTTGCGTCCCTCCACCGCTTGTCCCATGCCCAACAGATCCCAGTCTCCCATGGGTTGATCCAGGAGAACCTGCGAAAGTTATTGAACCAAAACTCTCGGATAATAAATCGTCGGTTGTGTCGTTATCTTACGATAATAACAAGGATGATAGTGCAGGGTCAGACAACGCTGAACCTAACAAACCCCACAATCACGAACAAAAACTTTTATTATCTACCAAGGGGAAAACCAAAGAACAAGTGGATCGTTCTTCGTGTGGATCCAACACGCCATCAGGTAGTGAGGCAGAGATGGATATGCGTGCAGACGAGAAGGATGAATCCAAAGAGCCTAAT---GATAATAATGTAAACGAATGGATAGGTGAACCAAACAATTACTTCAGTAGTTGTAGAAGCACAGGAGGCAACATAACCGAGTCTTGGAAGGAGGTCTCACAAGAGGGGAAACTGGCCTTTAAAGCGCTCTTCTCAAGACAAGTACTACCACAAAGCTTTTCACCACCGATGAGCTGCATAGACAACAGGGAGCAGCAGCTGATGAGTGACACTGATAAATTACGGATAGATCTCAATAGCAAGACACCATTGACTTGTATAGATGATGATGATCGCAGAGAGAGCATTGAGAAAGTTGAATTGATGATGGGCATTGAGAAACCTAAGGCTCGTCGAGCAGGATTTAAACCATACAAACGGTGCGCAATGGAGAGCAGCTCAGTGACTAACACTAGTAATCAAGGTGAAGAAAGAGATCCAAAAAGGTTATGTTTACAAGGA

------------------------------------------------------------------------------------------------------------------------------------------GAACACATTGGGACTAAAACTGCTGTTCAGATCAGAAGTCACGCACAAAAGTTTTTCTCAAAGTTGGAAAAGGAGGCTCTCCTTAAAGGTATGCCGCTTGGACAAGCTTGTGAAATAGATATTCCTCCTCCCCGTCCTAAAAGAAAACCAAACAATCCATATCCTCGGAAGACCAGCTCAGACTCTCCTAGCTCTGTATCTGTGGGACTGAAGGATGGGAGTTTCTTAGTATCAACTTCTTCTTCACACTCCAGTAAACAGATACAGGACCTGGAGAATGACTTGCATCTCGAG---CCTTGTAAGAAGACAAGTGGAAAAGAATTGTTTGACTTGGACAGTAATAGCTCAGGCGTTCTTACCCTTTCCCAAGAGACTTCATGTACATCCTTGTCTCCATCAATTAAAAGTCCACCAAAAATTGTGGAACTCAAAGCCCACTCAATTTTCAGAGATTCTGCCCCCTTGGTGAAAGAAGAAAGTAGCGAATGTGGTAGGGTAGATAAATACCTTCTTGACACAGATGTTGAGGAAACTCACAGAATTGACGCCACCTGTATAGACCTGGATGATGGATTGAGTGGAACCCCAATCTCGAGTAAC---TCCCGCAAATATTTGGACGAGGAAGTGATCAAAGGAAATAGACTGAAGCAGCAAGAAGATCTCTGTCAATCAAATCAGAATTATCCAAGGCACATACCCATCCAGGTCTTGGAAAGCAGCACAAAAGCATGCACTGAGAGTCCGCCCCCCACCATTCCTCGACTTGTAGGTACCGAGAATTCAGAGCCATCTACGTGTGTCAATACTGGAAGTCACAGCACCATATCCACTCAGCAGCCATTGCCACTTAACCCATCCACCACCAACAATCAAGAAAGCTACAACTTGTCAAATTTTTCGAATCTTATTGTATCTACTCTTCTGCAGAACCCAGCTGCCCATGCTGCAGCAAGTTTTACAGCTTCTTTGTGGCCTTTTACGAATGTCAAAACTACTGCTGATGGTGCAGACCCGAATACATCAGCAATTGCTGCTGCTACAGTAATGGCAGCTTCTGCGTGGTGGGCTGCTCATGGGTTGCTGCCTTTATGCCCTCCTCTTCAGTCTGCTGCTTTTACTTGCGTCCCTCCACCGATTGTCCCATGCCCAACAGAATCCAGTCTCCCATGGGTCGATCCAGGAGAACCTGCGAAAGTTATTGAACCAAAACTCTCGGATAATAAATCGTCGGTTGTGTCGCTATCTTACCATAATAACAAGAATGACAGTGCAGGATCAGACAACGCTGAACCGAACAAACCCCACAGTCACGAACAGAAATTTGTATCATCTACCAAGGGGAAAACCAAAGAACAAATGGATCGTTCTTCGTGCGGATCCAACACGCCGTCAGGTAGTGAGGCAGAGATGGATATGCGCGCAGACGGGAAGGACGAATCCAAAGAGCCTAATAGTAATAATAATGTAAACGAATGGATAGGTGAGCCAAACAATTACCGCAGTTGTTGTAGAAGCACAGGAGGCAACATAAACGAGTCTTGGAAGGAGGTCTCACAAGAGGGGAAACTGGCCTTTAAAGCGCTCTTCTCAAGGCAAGTACTACCACAAAGCTTTTCACCACCAAAAAGCGGCATAGACAACTGGGAGCAGCAGCTGATGAGAGACGATGATAAATTACGGATAGATCTCAATAGCAAGACACCATTGACTTGTATAGATGATGATGATTGCGAAGAGAACATTGACAAAGTTGAATTGATGATGGGCATTGAGAAACCCAAGGCTCGTCGAGCAGGATTTAAACCATACAAACGATGCTCAATGGAGAGCAGCTCAGTGACTAACGGTTGTAATCAAGTTGAAGAAAGAGATCCAAAAAGGTTATGTTTACAAGGA

5567 Unigene27730_R-bungei Unigene20529_R-tanguticus

AAGATCTTCAACTGGGTGCACCGCAAATTCCATTGCACTGGCGTCGGCTACGTTATGTCTCCTAAGAAAGATGACATTGCCATTACTATCACCAAATCCGAAAACACGGTCACAAATGAATTCGACACGAATTCCTTACTAGGTCCGGTGACTTTCATGGAAGCATGGAAAGATGGCATACTAACCATAGGGACGTTTGGGTTCGATGAATATTCAGTCATCCATGAAGAAGAAGTGGAGGAGGACGACGAAGAAAAGCAAGAGCAAGTGGAGAAAGAAAAAGATTATTCCTCTAGGGAGACTATTGAGGAAAAGGTGACGACCACTAAAGTTGCAAATTTGAAACCGGAAATTGCTACTATCAAGAGCAGTAACACGATACAAAATGTCGTTGAAGAAACCCCGGAAACCACACCTCTCCTTGAACTGGTTGAATCCGAAATAAGCATTGTGAAAAAGAGAGTTACACTAGCTGATTTACTCTCAGTGGCAGCAGATGCAGTGGACATACCAGACTCAACTCAAATATCGGTAGCGACTACCAAAAGATCGGCCGATATTGATAAGAAAGGTAAGTTGTCATTTGGCAAGAAGCCCAAAGATGAGGATTCTCGCCCAAAAAGAAAACTACATAGGATGATATCGAAAATGCTGAAAAAGAAGATTTACCCTGATGTTGAAGGAAAAAGTCCTGGGAGAGATGGATTGAATCGCGCATCAGGACCAATTGAAATGGTTTCACTTATTCAA

------------------XXAGAAAAGTACCAC------------------------------------------------------------------------------------------------------------------------------------------------------------------------------------------------------------CAAGAGGAAGAGGAGAAAGAAAGAGATTTTTCCTCTAGCGAGAAAGAAAATGAGGAAAAGGCCACATTCATTAAAGTTATAAATCTTGAGAAGGTCACAAATTTGAAACCTGTAATCGCTATTATGGAAAGCAATAACATGATACAAAATGTCGTTGAAGAAACCCCGGAAACCATACCTCTCCTTGAAGTGGTTAAATCCGAAATAAGCATTGTGAAGAAGAGAGTGACCCTAGCTGATTTACTCTCAGTGGCAGCAGATGCAGTTGAAATACCAGACTCGACTCAAATACCGGTAGCTACTACTAAAAAATCGGCCGATATTGATAAGAAAGGTATGTTGTCGTTTGGCAAGAAGCAC---------------------------------------------------------------------------------------------------------------------------------------------------------

257 CL1583.Contig1_R-tanguticus Unigene16921_R-bungei

TTTGCATCGAGAGATTCTACTGCCGAGGAAATACGCAAGGCACTGCTCGATGATGCAATAAATTTGGTAGGAGTACATGGGATGCCAGGGGTGGGAAAGACCACCTTGATCAAAGCAATAGCAAAACAAGTGAAGGAGGAACAGCTGTTCCAGGAGGTTGTGGTGGTAATTGTATCCCAAAACCCCAGTTTGAAAACACTTCAAGGAGATATCGCAGAGGGATTGGACTTCTCACTCGTTGGGGACAATGTGTTGAGGAGAGCTCAGCAATTGTCTGAGAGACTAAATCAAGATGCTAAGATGACCCTTGTAATTTTAGATGATGTATGGGATAGGATTGAACTTTCAGAAGTTGGAATACCTTACAAAAACAATGGAAATTGCTGCAAGGTTGTATTTAGTACACGGGATCAAGGCGTGTGTGATAGAATGGAAGCAAATGCCAAGATCGAAGTTGCACTCTTATCAGAAGAGGATTCATGGATCCTCTTTCGCCAGAAAGCTGGCACTGTGGCGGACTTATCACTTGCTCAAGAGCTTTCAAGTGAATGCAAGTGCTTGCCTTTGGCAATCGTTACACTGGGATTGGCTTTGAGAAACAAGAATGAAAGTTTTTGTGCTGATGCACTTCAGCAATTGCGGAAGTCGATCTTCAAAGGTATGAGTCCCGTGGTTTCTTCTATAAAGTTGAGCTACAACTTTTTAGAAAGTGAATCAATCAGACTTTGTTTTTTATTTTGTTGTTTATTTCCCGAGGACCATAGAATCAAATCAGAGGTATTTTTAATTTATGTGATGGGAGAAAAGCTACTTGAAGATGTGGACACATATGATGAAGCAAGGGGTAGATTA------------------------------------------------------------------------------------------------------------------------------------------------------------

---------------------------------------------------------------------------------------------------------------------------------------------------------------------------------------------------------------------------------------------------------------------------------------------------------------------------------------------------------------GTTGGGATACCTTACAAAAACAAAGGCAAATGCTGCAAGGTTGTATTCACTACAAGAGAACAAGATGTTTGTGGTGGAATGCAAGCAAATGCCCAAATCAAAGTTGCCCTCTTATCAGAAGAGGATTCATGGGAACTGTTTTGCCAGAAATGTGGCACTGTGGCGAAATTACCAATTGCTCGAGAGCTTTTAAATGAATGCGACCGCTTGCCCTTGGCAATCATCACGTTGGGATCAGCTTTGTGTGACAAGGATGAAGAAGTCTGTGCTAATGCACTTCGGCAATTGCAGAAGTCAATTTATGAAAAAATGAGTCCCGTAGATTCCTCTATAAAGTTGAGCTATGATTTTTTGACAAAGGATCAG---AAGGTATGTTTTTTGTTTTGTTGCTTATTTCCCGAGGACCATGTAATTGAATTAGATGTATTATTAAGTTATGTGATGGGAGAGAAGCTACTTGAAGATGTGGACACATATGAAGATGAAAGGGGTCAGTTGTACACTATCCTAGACAAACTTGTATCTTCTGGTTTGCTGTTGAGAGACGAGGACGGAGACATAAGAATGCACGATGTGGTTCGTGACATGGCTATCTCAATTTCAAAGGAAGAGGAAGGGCATATTGTGAAAGCTGGAAGGAATTGGAGTTACTGG

749 CL2674.Contig2_R-tanguticus Unigene12471_R-bungei

------------------ACTGAGAATGCTAAGCAAGGTGAGAAGGATGGTGGAACCACAGAGGTTGAGAATTGTGAGAACGAACAGATACAGTCAACACCCCCAGATAATGATATCTTCTGCAAACCTGGGCCACACAAGGGTAAGAATGATTCACTGGAGTGCCAACGGAGCAACGTAACTCATTGTGAAAAGAAAAGGCTTGTTGGTGATGTCGAGAAAGATACACATTCAAACACAAGGGCTAAATTGGGAACTCTTACACCAAGTTCATGCATGAAAATATATAGGACGCCAGGTTCTTTCAGCTACAGAAGATTGTTACCATACCTTATGGATCTCAAGAAAAATGATACAAGTACTTTGGGAACTCAACCATGTAAAATTATTGACAAAACCGTATATGAAGAGCCTTCTTCGTTATCAGTGCCAAAAGCGACGTTAGAAGATGTGTGTGGTCCAGACACTTTGCCAATCCTCAAATCAACAGAAACAAATCCTCCTGCTGGTTCTGATTGCATAAACTTGGGATCAGATGATGACCACCTTGTTCCTGAATTAAGTGCCCATACAGATGTTGAAAGTCCCTCACAAGTTAAAGAGCAGCCAATGGACGAACCCATCGATACTGAACCAACTCGCAATGAAACAAATCTTTTGATCAAGTCACCACCTTCTCTTGCTATACCTGAACCTGACATGAACTCCCCTGAAGTGTTGAGCTTGAATCAAGAGCCATCCAGTATTGCCCCCTTTTCTTCTCCAAGGGAACTCATATCAACTCCATCACCCAGTTTACTAAAATCAACTCCATCGCCCGGTATACTAAAGAGATATCCACGAGGATGCAGAGGCTTATGCACTTGTCTTAAGTGTACTTCTTTCCGTCTCCATGCAGAAAGAGCATTTGAGTTTTCAAGAAATCAAATGGAAGACAGTCAAGATGTAGCAATGGCGCTTGTAAAGGAACTCTCATCTATTCGAGCTCTTTTAGAAAACTCAGCTAATAGCGCCGGATGTGATGTTGTTCTTCCTGTCGCCAAGGTGAAAGAAGCTTGCATGAAAGCCTTAAGAACAGAAGAAGTAGCAAAAAGTCACTTGAAGGAAATGAATCAAGAACTCCATTTTCATTGTAAAATCCCAGGTGTGCCTCGACCAAGAGTGAGGTTTGCT

GAAACAAATCTACCTGTCAAGTCATCATTGTCACTTGCTACCCCGCAGTCATTAACCCTAAAAGTGGGCACTGAGTACAACGAACAGATACAGTCAACACCCCCAGATAATGATATCTTCCGCAAACCTGGGGCACACATGTATAAGAATGATTCACTGGAGTGCCAACGAAGAAATGTAACTGATTGTGACAAGAAAAGACTTGGTGGTGATATTGAGAAAGATACACATTCAAACTCAAGAGCTAAATTGGGAACTCTTACACCAAATTCCCGCATGAAAATATATAGCACGCAAGGTTCTTTCAGCTACAGAAGATTGCTACCGTACCTTATGGACCTCAAGAAAAATGATACAAGTACTTTGGGAACTCAACCATGTAAATTTATTGACAAGACCATATATGAAGAGCCTTCTTCGTTTCCAGCGCTGAAAGAGAAATTAGAAGATGTGTGTGGTCCAGACACTTTGCCAATCCTCGAATCTACAGAAACAAATGCTCCTGCTGGTTCTGTTTGCATAAACTTGGGATCAAATGAAGACCACCATGTTTCTGAATTAAGTGCCCATACGGTTGTTGAAAGCCCTTCACAAGTTATAGAGCAGCCAATGAACGAATCCATTGATACTGAACCAACTCATAATGAAACAAATCTTTTGATCAAGTCGCCACCTTTTCTTGCTATACCCGAACCTGACATGAACTCCCCTGAAGAGTTGAGCTTGAGTCAAGAGCCATCCAGTAGTGTCCCCTTATCTTCTCCTAAGGAACTCATATCAACTCTATCACCTGGTTTACTAAAATCAACTCCATCACCCGGTATACTAAAGAGATATCCAAGAGGATGCAGAGGCATATGCACTTGTCTTAAGTGTGCTTCTTTCCGTCTCCAGGCTGAAAGATCGTTTGAGTTTTCAAGAAATCAAATGGAAGATAGTCAAGATGTAGCAATGGAGCTTATAAAAGAACTCTCGTCTATTCGAGCTCTTTTAGAGAAATCAGCTAATGGTGCCGAATGTCATGTTGTTCTTCCTGTCTCCAAGGTGAAAGAAGCTTGCATGAAAGCTTTAAGAGCAGAAGAAGCAGCGAAAAGTCACTTGAAGGAAATGAATCAAGAACTCCATTTTCATTGTAAAATC---------------------------------

4016 Unigene8649_R-bungei Unigene1235_R-tanguticus

TCTATAAGAATGCCAAGTACTCGTAAGAAACAGTGTGATCAGAGTTCGGTTATTTCGAGTATAACTCCTGTTGATAATTGCTCGTCGCGGTGGATTCGTAATAACTCGCAACTTCCACATTCTTCCTTATCCAATCATCAAAATTTGAAGATGAACTCTGCCCCAAAAAAGTTTTTTGATGTGAAGGAGCTCCATAACCAGGATTTGTCTCCAGCTCAATCAACCGGCCGATCTCACCATGAAGTGGGCAATGCTGGAGGCAACCTTGACGGAAAACACGTCTCATATCAGTCTGGTCTTACTGAAGCTTACGGGCAACCAATGAAAATCAGCGAATCAATCTTTCCACAGGGGACTCCGAATTTCTCTTTCACTCAATCGCAGTCAATGGTCTTCATGCCATACCCTTGTGCTGATCCACAGTATGGCAGCTTTCAACATGCTATAGGGACTATCATGCACCCACAATTTGGGGGTATATTCCCGGGTCGAGTTCCACTGCCACCTGATTTCGGAGAAGATGAACCCATATACGTCAACTCGAAACAATATCATGGAATCCTTAGGCGAAGGCAGTCACGGGCCAAGCTCGAAGCCCAGAACAAACTGCTCAAAGATAGAAAGCCGTATCTTCACGAGTCACGGCATCTGCATGCACTGAAGAGGGCTAGAGGTGGCGGTGGGCGTTTTTTGAAGAAAAAGAACCTAGAGCAATCAAACGAAACGGGTGCTAATAGCAATTCAGAGACTGACAAGACGGGTGCCGCTATAACATCTCGTTCAGTTAACGACAACCTGAATCAACCATTGAATCCTTGGTTGACAGAGTACCACCATCCTCATATGAATGAAACAGTTCAAAATGGAGGAAGGGGAAGT

------------------------------------------------------------------------------------------TGGATTCCTAATAACACACAACCTCCACATTCTTCGTTATCCAATCATCATAATTTGGGGATGAAAGCTTCCCCGAAAAAGTATTTTGATGTGAAAGAGCTCCATAACCAGGATTCGTTTCCAGCTCAATCAACCGGCCGATCTCACCATGAAGTGGGCAATGCTGGAGCCAACCTTGATGGAGAACACATCTCATCTCAGTCTGGTTATACTGAAGCTTACGGACAGCAAATGAAAAACAATGAATCAATCTTTCCACAGGGGACTCCGAGTTTCTCTTTCACTCCATCACAGTCAATGGTCCTCATGCCATACCCTTGTGCTGATCCGCATTATGGCGGCTTTCAACATGCCATGGGGACTATTATGCACCCACATCTTGGGGGTATGGTACCTGGTCGAGTTCCACTGCCACCGGATTTTGGAGAAGATGGACCCATTTACGTCAACTCAAAACAGTATCATGGAATCCTTAGGCGTAGGCAGATACGGGCCAAGCTCGAAGCCCAAAACAAACTGCTCAAAGATAGAAAGCCGTATCTTCACGAGTCACGGCATCTGCATGCACTGAAGAGGGCTAGAGGCGGCGGTGGGCGCTTTTTGAAGAAAAAGAACTTAGACCAATCAAACGAAATGGGTGTTAATAGTAACTTGGAGACTGACAAGATGCGTGCTGCTATAACATCTCGTTCCACTAACAACAACCCGAATCAACCATTGAATCCCTGGTTCTCAGGATACCACCATCCTCATATGAATGAAACTGTTCAAAATGGAGGA---------

4135 Unigene22691_R-bungei Unigene135_R-tanguticus

ATGAGGGAGAAGAATTCTCAAAATATTAAAATGATTGACCGGAATTGGGCCACAAAGCGCAAGCGGAAACGACTTCCGAGTGGGACAGATGTATCCAGTGACAGAGAAACTGTTTCTGTACCTGTAGGTTCTCCAAAGGACAGTCCTCTAGCTAAACGCAGGTTGAGCAGCGATGGAACTTTGGGCCCATCTCCACACAAGGAGAAAGGAAATGATGGGTATTACTACGAATGTGTAGTATGTGATCTTGGAGGCAACTTGCTGTGCTGCGATAGCTGTCCTCGAACATATCATCTCGAGTGCCTTAATCCACCCCTCAAGCGTACTCCTCCTGGCAAGTGGCACTGCCCTACTTGTGAGAAAAGTAATTGCGTAATGTCCATAAGGGCTGCTGACTCGACATCAAAATCTCGGACAAAGCTAATTATTAGAAGATGCAAGTCCGAA---AAGTCCTCTGACTTCAACAAGACACCGGAGATGGTACAAATATCCGACACAGATAAATCATCTTGTATAGAAAAGCCGCGCTCATCTCTTAAACTTCTATTTACTAAGAAAAAGCCGAACTCCACCGACGCGGATGTCTCCTGTGGAATGAAGTTGAGTTCTGCTTCCCATGGCGAATCTATCAAAGATCCTCCACCACCTGAAAATGCTGACGTTGTGGAAAAACCAACTCTTCCACCTGAAAATACTGAAGTTGAGGAAAAACGTACGCTTGCTTGCATAGACATGCCCAGGAACAAC------CCTGCAAGTTTTGCCATGGAGGAGACTCTTACTTCTGACCGAGTTTTGGATGTCGAATCAAATGATGGGGATGGGATTCCAGATAGGATTCCTGACCTCCCTGGTGACAATGGCATTGCTGAAGAGAAGTTTAATTCTTCTGTGGGATGTACCACAGAGAAACCTAAAAGGAGGAAAAAAAGTAAAGGAAAAGAAAGTGGAACAAAAAAGAGTAGGAAGGATAAGAACAAAAGCCCTGTAAAAACTCCTGAGAAGCATGATAAGATAACATCTCCATCTCCTAAAAAGAGCAGACCTCGACAGAGAGTTAAGTTTGCCATCGACGAGATTTCTGAATCTCCGTCAAAACCAGATCTTGGAGAAAAATGTCCAGCCATTAAACAAAAACACAAGAAACGCTTGCAGGGAAAATCACATTCATCAAATGAATCAGATGAAGTGCAAGTTGCAGAGGACGAAAGAACTTATGAAGAAAGTGCTCTC---GAAAGTCACCAGGTCGACCGGATTTTGGGTTGTAGA------------------------------GCGCAGAGTATCATTACTTCACTTAACCCAGTTGGATGCTCTATTTCTCTTCCAGACGCGGTATCTAAGGACAGTTCTTTAGGGGCCTCAAGTGATACAGCTTCTTTATTGACCCCAGAAAGTAGCAAAGAAAGTAACAAAGAGTTATTAGGCGATAATCCCTCTGGTGACAAAGTTGTAGATGCGGAGGATGCTAAAACTTCTGCTGTGAATGACAATAGTAGCAGTAAAAGTACCAAGAATTGTACCAAAGCAGATCCAGTACATGTTTACCGAAGATCTGTTGCCAAAAAGAGCATGCGCGTTGATTCCAAGGATTTACCAGGAAGATCTATACAGGGCCAAGGCCCTTCAAGTGCGAATGCTGCGGTCCCAGACGAATCGACCGAAAAGGACAGTAATTCACAAAAAGTTGCGGATATATTAGTGCGTGTAGAAGAGAATGTTGCTCAACGTGTCAATTCAGGCGCTCAAGGTGACTTTATGGAAAATGGAACTTCTGAAACTTCCCTTCCCTGCTCCACAAAAGATGTGGAGTCAGATAAAAAATCAAACAATTACACAGAGAAAAAGGGAGAGGCAGCCGTGGACGAGTCACTCTTAAATAAAGAAACCTCCCAGTTTGAATATTTGGTCAAGTGGGTGGGTCAATCACATATACACAATAGTTGGATTTCAGAAGCTCAACTGAAAGTTCTGGCGAAGAGAAAATTTGACAATTACAAGGCCAAGTTTGGTAACACAATCATAAATCTTTGCCGTGAAGAGTGGAGCCAGCCGCAAAGAATAATTGCCCTTCGTGTTAGGGAGGATACTACTGAAGCTTACGTGAAATGGTGTGGTCTCCCTTATGATGACTGTACGTGGGAAAGATTGGATGAACCTGCAATTGGAAAATGTCCACATTTGGTTACCGATTTTAAGCACTTCGAACAACAAACCATAAACAATGATGCTGGAAAGGCAGGTGCCACGAAGAGTAAGATTAAGTCGCAGTCAATTGAGATACACCCACTCACACAGCAA

ATGAGGGAGAAGAGTTCTCAAAATATTAAAATGATTGACCGAAATTGGGCCACAAAGCGCAAGCGGAAACGGCTTCCGTGTGGGACAGATGTATCCAGTGACAGAGAAACTGTTTCTGTACCTCTAGGTTCTCCAAATGACAGTCCTCTTGCTAAACGCAGGTTGAGTAGCGATGGAACTTCGGGTCCATCTCCACACAAGGAGAAAGGAAATGATGGGTATTACTACGAATGTGTAGTATGTGATCTTGGAGGCAACTTGCTGTGCTGCGATAGCTGTCCTCGAACATATCATCTCGAGTGCCTTAATCCACCTCTCAAGCGTACTCCTCCTGGCAAGTGGCACTGCCCTACTTGTGAGAAAAGTAATTGCGTAATGTCCATAAGGGCTGCTGAGTCGACATCAAAATCTCGGACAAAGCTAATTATCAGAAGAAGCAAGTCCGAAAGTAAGTCCTCTGACTCCAACAAGACACCGGAGATGGTACAAATATCCGACACAGATAAATCATCTTGTATAGAAAAGCCGCGTTCATCTCTTAAAGTTCTATTTACTAAGAAAAAGCCGACCTCCACTGAAACGGATTTCTCCTGTGGAATGAAGTTGAGTCCTGCTTCCCATGGCGAATCTATCAAAGATTCTCCACAGCCTGAAAATGCTGACGTTGTGGAAAAACTAACTCCTCCACCTGAAAATACTGAAGTTGAGGAAAAACGTATTCTGGCTTGCATAGACATGCCCAGGAACAACCCTGCACCTGCAAGTTGTGCCATGGAGGAGACTTTTACTTCGGACCGAGTTTTGGAGATCGAATCAAATGATGGGGATGGGGTTCCAGATAGGATTCCTGACCTCCCTGGTGATAATGGCATTGCTGAAGTGAAGTTTAGTTCTCCTGTGGGATGTACCACAGAGAAACCTAAAAAGAGGAAAAAAAGTAAAGGAAAAGAAGGTGGAAAGAAAAAGAGTAGGAAGGATAAGAACAAAAGCCCTGTAAAAACCCCTGAGAAGCATGATAAGATAACATCTCCATCTCCTAAAAAAAGCAGACCTCGACAGAGAGTTAAGTTTGCCATTGACGAGATTTCTGAATCTCCGTCAAAGGCAGATCTTGGAGAAAAATGTCCAGTCAATAAACAAAAACATAAGAAACGCTTGCAGGGAACAGCGCATTCATCAAATGAATCAGATGAGATGCAAGTTGCAGAGGACGAAAGAACTTCTGAAGAAAGTGCTCTCGTCGGAAGTCACCAGGTTGACCGGATTCTGGGTTGTAGAGCCCAGAGCATCAATACTTCACTTGACCCAGCGCAGAGCATCAATACTTCACTTAACCCAGTTGGATGCTCTATTTCTCTTCTAGACGCGGTATCTAAGGACAGTTCTTTAGGGGTCTCAAGTGATACAGCTTCTTTATTGACTCCAGAAAGTAGCAAAGAAAGTAACAAAGAGTTATCAGGCGGTAATCCCTCTGGTGGCAAAGTTGTAGATGCTGAGGATGCTAAAACTTCTGCTGTGAATGACAACAGTAGCAGTAAAAGTACCAAAAATTGCACCAAAGCAGATCCAGTACATGTTTACCGAAGATCTGTTGCCAAAAAGAGTATGCGGGGTGATTCCAAGGATTCACCAGGAAGATCCATACAGGGTCAAGGCCCTTCAAGTGCGAATGCTGCTGTCCCAGACGAATCAGCAGAAAAGGGCAGTAATTCGCAAAAAGTTACATATGAATTAGTGCATGTAGAAGAGACTGTTGCTCAACGTGTCAGTTCGGGCATTCAAGGTGACTGTACGGAAAATGGAACTTCTGAAACTTCCCTTCCCTGCTCCACAAAAGATGTGGAGTCAGATACAAAATCGAACAATAACACAGAGAGAAAGGGAGAGGCAGCCTTAGATGAGTCACCCTCCAATAAAGAAACCTCCCAGTATGAATATTTGGTCAAGTGGGTGGGTCAATCACATATACACAATAGTTGGATTTCAGAAGCCCAACTGAAAGTTCTGGCGAAGAGAAAATTTGACAATTACAAGGCCAAGTTTGGTAACACAATCATAAATCTGGGCCGTGAAGAGTGGAGCCAGCCGCAAAGAATAATTGCCCTTCGTGTTAAGGGGGATACTACTGAAGCTTACGTAAAATGGTATGGTCTCCCTTATGATGACTGTACGTGGGAAAGATTGGATGAACCTGCAATTAAAAGATGTCCACATTTGGTTACTGATTTTAAGCACTTCGAACAACAA---------------------------------------------------------------------------------------

9312 Unigene1120_R-bungei Unigene2829_R-tanguticus

---------------GCGGAATGGCTCACCGAACTTGAAATTGATGAGGATGAAGACCATGTCTTAATTGACAGAATGGAAATGGATTCTCTCGATGACATAACTCCAGAACAATTTGCAGCTCTATACAATGAGGACTTACAACACACATTTGGTACCTTCAGCGACAACGACAAAATGTCCCAGATCTGCTATGGGAGACCAACAAAGAAACTCAACCGTTCTACTTCTACTACTATTGATAGTTGGAACTTTGACGCAAGTGAACAAAGATCTTCCCCTGAAGCTGCTTCCTTTCCCGTTGTGGTCTGTTACGGAAATCAGGCCAGTTCTCCTAATCCCAAATCTGGTGGAAAGAATAAGAAAGTTGGCAAGCTGATTGCTAAGCCTCTCGGCACTTTAGAACATGTGATAGCAGAAAGGAAAAGGCGTGAGAAGCTAGCTCAACAATTTATAGCATTGTCATCTCTTGTTCCGGGCCTAAAAAAGATGGACAAGGTTTCAGTACTCGAAGCTTGTATGCAGCACGTAAAACACCTCCAAACAAGAGTTCAAGTACTTGAAGAACAAACTTCCAAGAAAACAATGGAATCTGCAGTCTTTCTCAAGAAATTTCAAGTTTCTTCCGACACTGATTCATGTTCAACCGGAAAGAGCAACAGTGATTTCTCGATTGAGCAACTTCCTGAAATCGATGCAAGAATATCAGATAGAAATGTTCTAATACGAATTCTCTATGAGAAGCGTAGAGGAGCGATGGCGCTAATACTGTTGGAAACAGAAAAGCTCAACCTTAATGTGACTAGTATTACTGTCCTTCCATTTGGGTGTTCCACATTCGACATAACA------------------------------------------------------------------------

ATGGAAATGCTTTCTGCGGCATGGCTCACCAAATTTGAAATTGAAGGGGATGACGACCATGTCTTAATCGACAGAATGGAAATGGATTCGCTTGATGACTTAACTCCAGAACAATATGCAGCTCTATACAATGAGGACTTCCAACACACATTTGGTACCTTCAGCGACACCGAAAAAGTGTCTCAGATTTTCGATGAGAGACCAGCAAAGCAACTCAAGGGTTCTGTTACCACT------GATAGTTGGAAGTCTGACATCACTGGACAAAGATCTTCCCCTGGAGCTGCTTCATTGCCCCCTATGGCCTGTTATGGAAATCGGGTCAGTTCTCCTAATCCCAAATCTAGTGGAAAGAATAAGAAAGCTGACAAGCCAAGTGCTAAGCCTCCCGGCACTCTGGAACATGTGATAGCAGAGAGGAAGAGGCGGGAGAGGCTTGGTCAGCAATTTATAGCGCTATCCGCTCTTGTTCCAGGCCTAAAAAAGATGGATAAGACTTCGGTACTCGCAGCTTGTATGCAGCACGTAAAACACCTCCAAACAAGAGTTCAAGAACTTGAAGAACAAACTTCCAAGAAAACAATGGAATCTGCAGTCTTTGTGAAGAAATTCCAAGTTTCATCTGACACTGATTCATGTTCAACTGGAAAGAGCTCGGGTGATTTCTCGATCGAGCAACTTCCTGAAATTGAAGCAAGAATGTCAGATAGAAATGTTCTGATGCGAATTCACTGTGAGAAGCATAGAGGAGTGATGGCGCAAATCCTGTTGGAAACTGAAAAGCTCAACCTTAATGTGATTAGTTCTAGTGTCCTCCCATTTGGGAGTTCCACATTCGACGTAACCATCATGGCTCAGATGAATGCTGATTGCAACATGACTGTCAAGGATCTGGTGAAAAAGTTAGGCTCGTCTATC

827 Unigene12908_R-bungei CL2834.Contig2_R-tanguticus

---------------------------------------------------------------------------------------------------------------------------------------------------------------------------------------------------------------------------------------------------------------------------------------------------------------------------------------------------------------------------------------------------------------------------------------------------------------------------------------------------------------------------------------------------------------------------------------------------------------------------------------------------------------------------------------------------------------------------------------------------------------------------------------------------------------------------------------------------------------------------------------------------------------------------------------------------------------------------------------------------------------------------------------------------------------------------------------------------------------------------------------------------------------------------------------------------------------------------------------------------------------------------------------------------------------------------------TCTCCATCTCCACCAGCTCGGCGCAGACCTCCATCTTCACCACTAACTCGGCGAAAGTCGCCATCTCCAGTGAGGAGAAGATCTCCCAGACAGAGAAGAAGGTCACCATTGCGACCTCCTACACAAAAATACAGGGGAGGTAGCCCTTATAGGAGGCGCAGTCCTGCGTACCAGAGGCATAGAAGCTCGAGCAGGGATCATGACGTTAGAAATAATGGTGCGGGGTCAAGAAGGTATCAGGACGACTATAGGCCTGAAAGGGATGGCAGTACAAGTTCCCCTCTCATTCCTGGTAAAGGGAAAGTTAAAGGCTCTGGAGTGGACAATGCATCACGTCAGCCACCTATATCTTTGAGGTCACCTCAGAGGGATCCAACTGATGAGAATGATGTTGGTAGGAAAATACCTGCTCTGCTACCTTCCCGTGAGGTTTCTCCTAGTCATTCTGGATCTTCGATACATACT------TCAAGTGCTAGTGAAGACCAGAGGGATATGGCAACTTATGCTAGTGGTGCAAGTCTTGCGAGGCAAACATTGTTGGACGATAGT---AGGACAGTAAGAGAGTCCCGCACTCGCCTTTCAAGGGTGAATGATAGCCAGAGATCAAAATCTCCTCAAAGGAGGGTTGGGCATTCTCACAAAGAAGGTGCTCACAGAAGTATTCCTATGAAGGACTCCCAGGACGGAGAGTATACTCCTGAGAGATCAGGTGCCCGTCAATCAGGAGAAGATGTGTGTAGTGTAGATGATATCGAGATAGGAAGGAAAGATCGCGAGAACAGGAGTGAAAAAGCTTCATTGAGGATTGAGTCAGTTACTCCCAGTAAAACTACTCCCCAGTCAGTTGAATACAGTCCTGGAAAGGCAGAGGTAAAAGGATTTACATCCGAAAATGCTGGTGATGAACATAAATTATCTCCTAGAGATGGAAATAATGTTGATGAGAAGCTACACTCTCATTTACGTGATGTCGTGGGCAGTGTCATAGATTCAGAGAATGAACATAACAAATCGAAAGGCTTGGAGAGAAAAAAGCATAAGAGGCATGACAGACATCATACGGCTTCAGAGGATGACAGTGGTTACGACTCTCATATAGATGAGAGGAAAGATGCAAAAAGGCGAAGAAAGGAGGAGAAGAAATCGCGAAAAGAGGAGAAACGTCGGCGACGTGAAGAGAGGCATCGTAAAAAGGAAGAG

ATGTCGGGAGGATTTTTCCGGGGTACATCAGCTGATCAAGACACGCGATTCTCTAATAAACAAGCGAAATTAATGAAATCGCAGAAATTCGCTCCTGAATTGGATCATCTGGTGGATATGAGTAAAGTGAAGATGGATGTAGTTAAACCGTGGATTGCTACAAGGGTTACGGAGTTTCTTGGTTTTGAGGATGAAGTGTTGATTAACTTTATATATGGCCTTCTCGATGGAAAGGAGATTAATGGAAAAGAAGTGCAAATCCAGATCACCGGTTTTATGGAGAAAAACACCGGGAAGTTCATGAAAGAGCTTTGGAATCTTCTTCTCAGTGCCCAGAAGAATGCCAGTGGAGTTCCTCAACAGTTCTTGGATGCTAAAGAAGAGGAAATTAGGAAGAAGAAGGCTGAGTCAGATCTAATAAGCCAGGAAATTCAGAGGAAGAAAGAGATTGAGCATGAGAAGCAGAAGAAAATGGACGAGGAGTTCGCCGCAAAGGCTGTGGATGATGCATCTGACCCAGTTCCAAAATATTCGACTGCTCAACCTGATGATGACAAAGTATCTGGCGGAAGGAATGGGCCGAGAAAGAAAAACAGGAGCCCCAGATCTCCACGATTATCAGAGCGTCCCGCTTCTCCCCGCCGAATTGCTCGCTCTGGTTCAATCAGCAAATCATTTTCCAATTCCAGGAGCTATTCAGAGGACAGTCACAAATCAAGGAGCAGGTCCTTATCTCCTCAATCACGACGGTATCTTTCACCAAGAAAGCGTTCTTTGTCTCCTCGCCGCAAGTATTCTCCTCGAAGATATCGTTCACCCTTAAGATGGAGATCACCACATTCAAGGAGAATGTCACCACCCTATTCACGAAGGAGATCACCCACTCCTAGAAGACGCAGGTCTCCGTCCCCCATGCGCCGAAGTTCACCCTCTCTGAGGAGGCGCAGGTCACCTGTTATGAGGAGACGCAGATCCCCTACTCCAGTGAGACGCAGGTCACCTTCTCCTGTGAGACGTCGATCTCCACCCCCTATGCGCAGGAGATCTCCTACTTTACATCGTAAATCCCCTTCACCTGTGCGGAGCAGATCTCCCCTTGCTACCCGCAGAAGATCACCAACTGCATCTCCATCTCCTCGGCGCAGTTCTCCATCTCCACTTCTTCGACACAGGTCTCCATCGCCTCCTGCTCGGCGCAGGTCTCCATCGCCTCCTGTTCGGCGCAGGTCTCCATCGACTCCTGTCCGGCGCAGGTCTCCATCTCCACCACCACGTCGCAGGTCTCCATCTCCACCAGCTCGGCGAAGACCTCCATCTTCACCACTAACTCGGCGTAGGTCGCCATCTCCAGTGAGGAGGAGATCTCCCAGACAGAGAAGAAGGTCACCATTGCGACTTCCCACACAAAAATACAGGGGAGGCAGCCCTTATAGGAGGCGCAGCCCTGCGTATCAGAGGCATAGGAGCTTGAGCAGGGATCATGATGTTAGAAATAATGGTGCTGGGCCGAGAAGGTATCAGGACGACTATAGGCCTAAAAGGCCTGGTGGTACAAGTTCCCCTCTCATTTCTGGTAAAGGGGAAGTTGAAGACTCTGGGGTGGATAGTGTTTCACGTCAGCCACCTATATCTTTGAGGTCACCACAGAGGGATCCAACTGATCAAACTGATACTGGTAGAAAAGTACCTGCTCCGCTACCTTCCCGTGAGGTTTCTCCTTGTCATTCTGGATCTTCGATACATACAGGAAAATCAAGTGCTAGTGAAGACCAAAGGGATGTGGCAACTTATGCTAGTAGTGCAAGTCCTGCGAGGCAAACATTGTTGGATGATAGTAGGAGGACAGTAAGAGAGTCCCGGACTCGCCTTCCAAGGGTGGATGAGGGTCAGAGAACAAAATCTCCTGAACGGAGAGTTGGGCATTCTCAAAAAGAAGGTGCTCACAGAAGTATTCCTACGAAAGACTCCCAGGACGAAGAGTATTCTCCTGAGAGATCAGGTGCTCATCAATTAGGAGAAGATGAGTGTAGTGTAGATGATATCGGGATAAGAAGGAAAGACCGCGACAACAGGAGTGAAAAAGCTTCATTGAGGGTTGATTCACTTAGTCCCATTAAAACCACTACCCACTCAGTTGAATACAGTCCTGGAAAGGCAGAGGTAAAAGGATTTACATCCGAAAATGCTGGTGATGAACATAAAGCATCTCCTAGAGAAGGTAATAATGTTGATGAGAAGCTACACTCTCACTTACGTGATGTCGTGGGCAGTGGCATAGATTCGGAGAATGAACATAACAAATCGAAAGGCTTGGAGAGAAAAAAGCATAAGAGGCATGACAGACATCATTCGGCTTCAGAGGATGACAGTGGTTACGACTCTCATATAGATGAGAGGAAAGATGCAAAAAGGCGAAGGAAGGAGGAGAAGAGATTGCGAAAAGAGGAGAAACGCCGGCGACGTGAAGAGAGGCATCGTAAAAAGGAAGAG

6865 Unigene23646_R-tanguticus Unigene10533_R-bungei

GAGTTGCAAGAGAAGATCAAAAGCATGGAACATGTGGAGGAGATCGTTCAGAACTTACAACAATTGAAGAAGAAACTTGCCTGGTCATGGGTATATGATGTGGACAAGCAAATCGAGGAGCATGTTGCTAAGCTTGAAAAGTTGAAAGAACGTGTGCCTACTTGTCAACTCAAAATTGACCGGCAACTGGTTAAGGTGGAGGAATTAAAGGAACACCTTTCCAAAAAAAAAGCTCAGGTTTCCAGTATGATGGAAACAACCTCTGAACTGAAGAAAAAAAAGGAAGAGTTGCGGCAAAATCTTTCTTCGGCAACGAAAGAAAAGCTTGAGCTTGAAGAGGAAAAGTTCCGTAAACGTAACCAAATTCAGAAGGTGGTTGAGCGTGTTAAGTTGCTTGAAAAGCAAGTTACGGATATTCAAGAGCAACATTTGAAAGATACACAGGCTGAAGAATATCAGATGGAGGAAGTACTGAAGGGGCTACAGGATGAAGTTGACATAGTAAAATCAAGCATAACAAGGTTGAAAGAAGAGGAGAATTCCTTGTCTGAACAGCTAGCAGTCGCTACGAGCAGGATTGAAAGTATTGTTTCTGAGATACAAGATAATGAAAGAAGACATCGTGATAGTGTTAATCAAATTGATAAACTCCGACAACATCAGAGTAATCAGGTAACGGCCTTTGGAGGAGATAAAGTGTTGCAGCTTTTACGGAAAATTGAGGAGAATCATAGAAGGTTTACAATGCCCCCTATTGGTCCCATCGGATCTCATGTGAAATTAAAAGATGATGCATGGGGCATAGCTGCTGAGAGCGCCATCGGGAAGATTCTAAATGCATTTATTGTAACAAATCACAAAGATTCTCTTGTTTTGCGAGAATGTGCTAAGATGGCATACTACCATCACCTTCAGATTATTATCTACGACTTCAGCAGACCAAAGTTGAATATCCCCAATCACATGCTACCGGATACACGACATCCAACTACATTTTCCCAAATAATTACTGACAATCCAACCGTGTCAAATGTTTTGGTGGATATGGTTGGCGCTGAGAGGCAAGTGCTTGTTAAGGATTATGATGTGGGCAAAGCGGTTGCATTTCATCAAAGAATTCAAAATCTTAAAGAAGTTTATACATCAGATGGGACTAAAATGTTTGCTCGTGGATCTGTCCAGACAACTCTTCCGCCAATGAAACGGAATAGAGCTAGCCGTTTATGCAGTTCCTATGATGATCAAATTCAGAAATTTCAGAATGATGCTTCGAAGTTTGAAGAACAAGTGCATCATGGTCGAGGCAGGAAAAGGGATGCAGATCAAGCTTTTAAGGAACTTAATACTAGATATAACAACGTAAAGAAACAACGGGAGTCTAAAGAAAGGGATTTGATTCGCAAGGACCTAGATGTACAAGATCTGAGGAATACA---------------------

---------------------------------------------------------------------------------------------------------------------------------------------------------------------------------------CAACTGGTTAAGGTGGAGAAACTAAAGGAAGACCTTTCCAAAAAGAAAGCTCAAGTTTCTGGTATGATGGAGATAACCTCTGAACTGAAGAAAAAAAAGGAAGAGTTGCGGCAAAATCTTTCTTCGGCAACGAAAAAAAAGCTTGAGCTTGAAGAGGAAAAGTTCCGTAAACGTAACCAAATCCAGAAGATGGTTGATCGTGTGAAGTTGCTTGAAAAGCAAGTTACTGATATTCAAGAGCAACATTTAAAAGATACACAGGCTGAAGAATGTCAGATGGAGGAAGTACTGAAGGGGCTACAGGATGAAGTTGACATAGTAAAATCAAGCATAACAAGGTTGAAAGAAGAGGAAAATTTCTTGTCTGAACAGCTAGCAGCTGCTAGGAGCAGGGTTGAAAGTATTGATTCTGAGATAAAAGATAATGAAAGAAGACTTTGGGATGTTGATAATCAAATTCGCAAACTCCGGCAGCATCAGACTAATCAGGTAACGGCCTTTGGAGGAGATAAAGTGTTGCAGCTTTTACAGAAAATTGAGGAGAATTATAGAAGGTTTACAATGCCCCCTATCGGTCCCATCGGGTCTCATGTGAAATTAAAAGATGATGCATGGGGCATTGCTGCTGAGAGCGCCATCGGGAAGTATCTAAATGCATTTATTGTGACAAATCACAAAGATTCTCTTGTTTTGCGAGAATGTGCTAAGATGGCACACTACAATTACCTCCAGATTGTTATCTATGACTTCAACAGACCAAAGTTGAATATCCCCAATCACATGCTGCCAAATACACGGCATCCAACTACATATTCCCAAATAATTACTGAAAATCCAACCGTGTCAAATGTTTTGGTGGATATGGTTGGTGCTGAGAGGCAAGTGCTTGTTAAGGATTATGATGCGGGCAAAGAGGTTGCATTTCATCAAAGAATTCAAAATCTTAAAGAAGTTTATACATCAGATGGGTCTAAAATGTTTTCTCGTGGATCTGTCCAGACGACTCTTCCGCCAATGAAACGGAATAGAGCTAGCCGTTTATGCGGTTCCTATGATGATCAAATTCAGAGATTTCAGAATGATGCTTCGAAGTTTAAAGAACAAGTGAGTCATGGCCAAGGCAGGAAAAGGGATGCAGAACAAGCTTTTAAGGATCTTAATACGAGATATAACAACGTAAAGAAACAACGAGAGTCTAATGAAAAGGATTTGATTCGTAAGGAGCTAGATGTACAAGATCTGAGGAATACACAGTCTGCTCAAGCTAGTTCT

3527 CL8991.Contig2_R-tanguticus Unigene21535_R-bungei

------------------AAGTTTGGTTCTAAGAAGAAGACTCCG---GTGTTCGAAGGAGGATCATACGATGTGTGTGAGCGAACAGTTGAGACGTCCGATTCAGACTCAGCATCTCCTCCAAACCAGTTGTTGATCATTTCGCCGACGTCCAAAGGCAAATACCCTGTTATCGTCTTTCTTCATGGCTACGCCTTAAGCATTTTCTTCTACCGAGAGCTTCTTAAACATATAGCAAGTCATGGCTACATAACTGTTTCTCCACAGTTGTCCGTTGGATTCCCGTGCAAGGACTACGATGAAATTAAATCATCAGCTGAAGTAACAAATTGGTTACCTACAGGCCTTCAATCAGTACTACCCGAGAAAGTTCAAGCAGACTTAGAGAAGCTCGCTCTAGTTGGCCATAGTCGAGGTGGTAAAACAGCTTTCACTCTCGCCTTGGAGCCTAGTAAGACCTCTCTAAAATTCTGCGTATTAGTAGCCGTCGACCCTGTGGCAGGATTTGCAAAAGGAATTGAAATACAACCCACCATTCTAACCAATGTACCAGCCTCTCTCAGGACAGGATTTCCAGTTATGGTCATTGGTACTGGTCTAGGAGGCTTATTATGTGCTCCAACTGGAATGAACCACCAAGAATTCTTTTATGAATCGCAACCTCCGTGTTATCATTATGTCGCAAAAGATTATGGTCCTTTGGATATATTAGATGATGATCTCAACATTATGGGGAAGATAATGACTTGTTTTTGTACAGCTGGGAAAGGAGCAAAGGATCCGATGAGAAGATGTGTAGGCGGACTTATTGTTGCCTTTCTAGAGAAGTACTTGGATGATGAAGAGGATCATCTTGAGGACATTGTTGAAGACCCCAGTATTGCTCCAGTAGCACTTTCTCCATGTGAATATGTTAGGTCA

AATCCAGACATCAAGAAGCTTGTTTCGAAGAAGAAGAAAACTCCGGTGGTGTTCGAAGAAGGATCATACAATGTGTGTGAGCTAACAGTTGAGACATCCGAATCGGACTCAGCATCTCCTCCAAATCAGTTACTGATCATTTCACCGACGTCCAAAGGCGAATACCCTGTTATCGTCTTTCTTCATGGCTACGCCTTGAGCATTTTCTTCTACCGAGAGCTTCTCAAACATATTGCTAGTCATGGCTACATAATTGTTACTCCACAGTTGCCCATTGTGTTCCCGTGCTCGGACTACGATGAAATGAAATCAATAGCTGAAGTCACAAATTGGTTATCTACTGGCCTTCAATCAGTACTGCCCGAGAAAGTGAAAGCAGACTTAAAGAAGCTCGCTCTAGTTGGCCATAGTCGAGGTGGTAAAACCGCTTTCACTATTGCCTTGGACCCAAGTAAAATCTCTCTAAAATTCTCCGCATTAGTAGGCGTCGACCCTGTGGCAGGATATGCAGAAGGATTCGAAATAAAACCCACAATTCTCACCAATGCACCAGCCTCTCTCGCGACAGGACTTCCAGTTATGGTCATTGGTACTGGTATAGGAGGG---TTATGTGCTCCAACTGGAATGAACCACCAAGAATTCTTTTATGAATCGCAACCTCCGTGTTACCATTATGTCGCAAAAGATTATGGTCATATGGATATGTTAGATGATGATCTCGGCATTATAGGGAAGATAATGACTTGTTTATGTAAAGCTGGGAAAGGTGCAAAGAAATCGATGAGAAGATGTGTAGGTGGCCTTATTGTCGCCTTTCTAGAGAAGTACTTGGATGATGAAAGTGATCATCTTGATGACATTGTTGAAGACCCCAGTTATGCACCAGTAGCACTTTCTCCATGTGAATATGTTAGGTCA

20 CL1041.Contig5_R-tanguticus Unigene27516_R-bungei

---------ATGTCTTCAATGGAGGCGTACACAGGCTTATCTCCAACAGCCTTTTTCACCATCCTCGCTCTCATGTTCGGTGTCTACAAATTCGTCTGCGCCTTCTTCGTCCACCCCGATGATTCCGATGAATCTAACAAAAAACCATCATCATCATCAATTGCTACAGTGGAGAAGGAGAAGGCTTCTGCTACTGTTGCTGCTCCTGTTCAAGTAGAGGAGAAGAAGGCCGTCTCTGCTGCTGCTGTTCAAGAGGAGGAGAAGGCTGCTACTGTTGCTGCTGCTGGTAAAGAAGTGGGCGATTTGAGTTGGGAACAGCTCCGAGCTTATGATGGTTCTGATCCTGCCAAACCCTTGCTCATGGCCATCAAGTCCCAGATCTACGACGTCTCCCCTTCAAGGATGTTTTATGGGCCTGGCGGTCCATATGCCTTGTTTGCAGGTAGGGATGCAAGTAGAGCCTTGGCACTCATGTCATTTGACCCGGATGACCTTACTGGGGATCTCGACGGCCTAGAGCCGTCAGAGCTTGAAGTGCTGCAGGATTGGGAGTATAAATTCCAAGAGAAGTATAAGAGAGTCGGGCAGATTGTCACACCAAGCAGAAATGAGCAAGCTTCCCCAGAAGAGTCTGGGAGAGAAAAACAA

TCCTTAATGGCGTCAATGGTGGAGTCGTACACAGGCTTATCTCCAACAGCCTTGTTCACCATCCTCGCTCTCATGTTTGCTGTTTACAAATTTGTCTGCGGAATGTTCGTC---------------------GATTCTTCCTACCCATCATCAACGGAGGTCAGT------------CACAAACAATTACCTGCTGCTGATGCTCCTCCTCGAGTAGAG------------GCTGCTCCTGATGCCATCGGTGAACAAGGAGATGATGGCTTA---------------------GTTGGGGATTTGAGCTCCGAGCAGCTCCGAGCTTATGATGGTTCCGATCCCACCAAACCCTTGCTCATGGCCATCAACTCTCAGATCTACGACGTCTCCCCTTCCAGGATGTTTTATGGGCCTGGTGGTCCGTATGCCTTGTTTGCAGGTAGAGACGCAAGTAGAGCCTTGGCACTCATGTCGTTTGACCCAAATGACCTTACTGGGGACCTCGACGGCCTAGAGTCGTCCGAGCTTCAAGTGCTGCAAGACTGGGAGTATAAATTCCAAGAGAAGTATAAGAAAGTTGGGCGGATTGTCACAACAACCGGAAATGAGCACACTTCCCCAGAAGTGACT---------------

5708 Unigene28109_R-bungei Unigene20892_R-tanguticus

ATGGAAAATCAAAATCCTCCACCAATTCGTCCATGGTTTCGCCTGGCATCCCAAATCAGGGTGGAACCCCCACCCCAGCCAAGGCCTCCTGGTCCTATCCGGCCTACACTTACTCAAACACTTAGTAGACCTCCAATTGCTTCTCAGCCACAACAAACTCAAGCTCCTGTTCCAGTTGCTCCTCCAGCACCAGTGCAGACAGTAGCACCTCCTGCACCACCCTCACCTAGGCTACCACCTTCGGCTCCAATTTCATCTTCTCCAACTCAAGCATCATTG---------CCATTACCGAAACCGTCGTCGCCACCACCTAGAACATCAGTACCATCATCCCCAACCCCCAAAATCTCACCCCCACAACCCAAAACATCACCAATGCCAAATACCACACCGCCACAGCGCAAAATGTCAGTGCCACCTTCCCCTGTTCAAAAAGCTATCTCCCCTCCTAAATCTCCCCCTCCACAACCCAAAATGACAGTCCCACCATCTCCAAAATCAAAACCTTACACTCCACCAAAAGCTTCACCCATTGCCAAATCCTCCTTACCATCAAACCCAAAGCCATCATCACCATTTCTCCCGAAACCTGAAGCAGATGAATATATCAAGTTTGGGAAGCCCAGAACCCCAACAACTCCACCACAATCCCCAACGATCAAACCTTCTGCATCTCCTCCATCTCCTCTGACACTTCCACCTTCTCAACTAAATCCAGTTCCGGAACCAGAAAGCAAGAATGTGGTAATGGATGCACATAAAAGTACCTTA------AACGAGAAGAGCAAGGAAGCCACTGACCTCCATCATAACAACATTCCCCATAACAAGACCAAGGGAGGTATGGGTAGCAGTGGGAATGCAGTGCATCACAAGAGAGAAAAAGAAAAAGTTTCTCCCAAGAAAACACATGAAGAACATGAGGAAGAAGGTATGAGAGTGATAACAATCGCAGGAGAGAATAATGGAGCCTGTATGGAGCTTGGGTCTTCGAAGAACAAAGGAAATCTGCAACCCCAGAATCTTATCAAAAAGAAAGGAGAT---GACTCAAGCAGCAGCAGCAGCGATGAGGGGCAGTCAAAGAAGAAAGATAATCGAATGTCGGGATCTTCTACCTTACCAATGAAAGCATTTGTGAACAGTAATGTGCAGGGCATTAACAACTCCATCCTCTTCCACAGTTCTTGTTCTCACAGTGATCCTGGTGTTCACCTTGTGTTCTCCAAAAAA

ATGGAAAATCAAAATCCTCCAGCAATTCGTCCATGGTTTCGCCTGGCATCCCAAATCAGGGTGGAACCCCCACCCCAGCCAAGGCCTCCTGGTCCTATCCGGCCTACACTTACTCAAACACTTAGTAGGCCTCCAATTGCTACTCAGCCACAACAAACTCAAGCTCCTGCTCCTGTTGCTCCTCCAGCACCAGTGCAGACAGTATCGCCTCCTGCACCACCCTCGCCTAGGCTACCACCTCCAGCTCCAATTTCATCTTCTCCAACTCAAGCATCATTGCCAGCCTCGCCGTTACCGAAACCGTCGTCTCCGCCACGTAGAACATCAGTACCATCATCCCCAGTCCCGAAAACCTCACCCCCACACTCCAAAACATCTCCAATGCCAAATACCACACCGCCACAGCGCAAATTGTCAGTGCCACCTTCCCCTGTTCAAAAAACTACCTCCCTTCCTAAATCCCCCCCTCCACAACCCAAAATGACAGTCCCGCCATCTCCAAAATCAAAACCTAACTCTCCCCCAAAAGCTTCACCAATTACCAAACCCTCTTCAACATCAAACCCAAAGCCATCATCCCCATTTCCCCCAAAACCAGAAACAAATGAATATATCAAGTTTGGGAAGCCCAAAACCCCAACAACTCCGCCGCAATCCCCAAAGATCAAACCTTCTGCATCTCCTCCATCTCCTCTGACACTTCCACCTTCTCAACTAAATCCAGTTCCAGAACCAGAAAGTAAGAGTGTGGTAATGGATGCGCATAAAAGTACCTTCCCTAGAAATGAAAAGAGCAAGGAACCCAGTGAACTCCATAATAACAACATTGTCCATAACAAGACCAAGGGAGGTATGGGTAGCAGTGGGAATGCAATGCATCACAAGAGAGAAAAGGAAAAGTTTTCGCCCAAGAAAACAAATGAAGATCATGAGGAAGAAGGTATGAGAGTGATAACAATAGCAGGAGAGAATAATGGAGCATATATGGAGCTTGGGTCTTCCAAGAACAAAGGAACTCTTCAGCCCCAGAATCTTAGCAAGAAGAAAGGAGATGCTTCAAGCAGCAGCAGCAGCAGTGATGAGGGGCAGTCAAAGAAGAAAGAGAATCGAATGTCGGGATCTTCTACCACACCAACGAAAGCATTTGTGAACAGTAATGTGCAGGGCATTAACAACTCCATCCTCTTCCACAGTTCTTGTTCTCACAGTGATCCTGGTGTTCACCTTGTGTTCTCCAAAAAA

9731 Unigene13467_R-bungei Unigene29132_R-tanguticus

CCACCTCAATATGATGCAGCTCCTGATGATTCAATCAGGCCTTTTATATCAACATTTTCGATGCCCTTAGACACCAATTCCATCACGTCCCAACACTTGGACTCTGGCTCTGCCACACATAAAGTGTCTGATAGAGTTCTTCCGGCTTCTGCCTCCGATGACATTCCTGTAAGCAGAGGTTATTCAAGTATTGATGGAACTGTATATGATACGCCTAAGGTGGAAGATAGTCTTTCATCCGAAAAACAACCAGACCTTCCTCCTCCTAAGCCAGCTGCTATAGAAACTGTTCGTCAAATCGAAACTTTATGCCAGTATATTGCTAAAAATGGTTCCGGTTTTGAAGTCACAGTCCGGGCAAAGCAACATGGGAACCCAAAGTTTGCTTTTTTGTTTGGCGGTGAGCCTGGCAGCGAAGCTGCAGTTGCACATGAATATTTTCAGTGGACGAAAAGAAATTGTCTTAAGGAGGAAGTTAAAATGCCTAATGGATGCGAGCCTTTAAAAGTTGAATCTTCTACATGTCCAAGTGGTTACTCAAATGAGGATGCATGCCATTCAGCTGCAGAATCTGACATGGATATGGAAGATGACAGTCACCAGTTCAACAAAAAGCAGATTATTGTTGCGTCTGCTAAAGTTTCAGAAGAGCTCGAGGTTCTTAATGTGAAAGATCTGCAACCTGGAAGTCAATGTTCTTCAAATCCAACAGGAAATGTTTCATCTGAATTTCAGCCAAGAATTGACATTCCAATATTGGCTAGAGAAGAAAAAGAATCTGTTCACACTCCTGTTCGGAACTCATCTGGAGCTTCTGAGGGTACATTAGATGACATCACTCACAAACCTGTCAGACCAGTTGACAAATTAACTCTGCCTAAAGCTTCTCCATCTGTTGCGGTACGTAGCAGCTCCAAGGAAGTCTCAGAATCAGTTAGCAATATGGGAAGCCCGTTCCGACTTATACAAGACTATGCTTCTGATGAGAGTGTTGAAGCTGATGTCAGCACTGAAAGTATTTCCCCCCCAATTCCAGTTGAGGAGACATGTTTGTCCAAGCAGTTGCTAAAGGAGGTGACCAGTGTGGAGACTAATTCAGGTTCCTTGAGTGTTCTTCTGCATGAAACAGAATTCACAGAGTCATATCCTACTCGCAAGTCAAGTGTATCGATTAAAGCAGATAAGGTGGTAGATGCGACTCATGTAGCATCCCCTATATTAGATACATTTGCTAAAACTAATGAGCTTCATAATGACAATCACGACTACCAACCATCTAATGATCCGGATCATGGGGATTCAATGCAAGGTGATGATGGTGTTGATTCTCAAAGTGGAAAGCATAACATGAAAAACGAAAGTCAAGGTTCACCTGCACTGAAAGTTGACGAATTTGGGAGAATGGTGCGAAAAAATGCGAGTGACAGTGACTCTGATAGTGATAAGGAGCATTACAGCGGGAGACGTCATAGAAGAGGCCGCAGTCGATCTCCCTTGGAAAGGAGGAGGAGTCGCAGTCCACGAAGAAGAAACGAGAAACGAAACCGATCTCGCAGTTGGTCTCCCAGAAAGCGAAGAAGCACGAGCAAAAGTAGGTCTCCACCTTCTGTTAGGCACAAGGGTGAATTTTCTGGGGAGAAATCGAGACGCGACAGAGATCAGACTCCTTTTTGTTTTGACTTCCAGATAGGAAGATGCTACCGCGGAGCTTCGTGTCGTTATTCACATCATGGACAGGGAGATTCAGTCAGGCGCTATAGAGGTAGACAAGATTGG---------------------------------------------------------------------------------------------------------------------------------------------------------------------------------------------------------------------------------------------------------------------------------------------------------------------------------------------------------------------------------------------------------------------------------------------------------------------------------------------------------------------------------------------------------------------------------------------------------------------------------------------------------------------------------------------------------------------------------------------------------------------------------------------------------------------------------------------------------------------------------------------------------------------------------------------------------------------------------------------------------------------------------------------------------------------------------------------------------------------------------------------------------------------------------------------------------------------------------------------------------------------------------------------------------------------------------------------------------------------------------------------------------------------------------------------------------------------------------------------------------------------------------------------------------------------------------------------------------------------------------------------------------------------------------------------------------------------------------------------------------------------------------------------------------------------------------------------------------------------------------------------

CCACCTCAATATGATGCACCTCCTGATGATTCAAGCAGGCCTTCTATAACGACATTTTCGATGCCCCTAGACACTAATTCCATCTCGTCCCAACACTTGGACTCTAGCTCTGCCACACAGAAAGTGTCGGATAGAGTTCTTCCAGCTTGTGCCCCCGACGACATTCCTGTAAGCAAAGGTTATTCAAGTATTGATGGAACTGTATATGCTATGCCTAAGGTGGAAGATACTCTTTCATCTGAAAAACAACCAGATCTTCCTCCGCCTAAGCCAGCTGCGATAGAAACTGTTCGTCAAATCGAAACTTTATGCCAGTATATTGCTAAAAATGGTTCCGGTTTTGAAGTCACAGTCCGGGCAAAGCAACATGGAAACCCAAAGTTTGCTTTCTTGTTTGGCGGTGAGGCTGGCAGTGAAGCTGCAGTTGCACATGAATATTTTCAGTGGACGAAAAGAAATTGTCTTAAGGAGGAAGTTAAAATGCCTAATGGATGCGAGCCTTCAAAAGTTGAATCTTCTACATGTCCAAGTGGTTACTCAAATGAGGATGCATGCGATTCAGCTGCAGAATCCGACATGGATATGGAAGATGACAGTCACCAGTTCAACAAAAAGCAGATTATTGTTGCGTCTACTAAAGTTTCAAAAGAGCTCGAGGTTCTTAATGTGAAAGAGCTGCAACCTGGAAGTCAATGTTCTTCAAATCCAACAGGGAATGTTTCATCTGAATTTCAGCCAAGAGTTAACACTCCAATATTGGCTAGAGAAGAAAAAGAATCTGATCACACTCCTGTTCGGAACTCATCTGGAGCTTCTGAGCGTACATTAGATCTCATCAATCACACACCTGTCAGACCAGTTGATGAATTAACTCTGCATAAATCTTCTCCATCTGCTGCGGTACACAGCAGCTCTAAGGAAGTCCCAGATTCAGTTAGCAATATGGGAAGCCCGTTCCGACTTATACAAGACTATGCTTCTGATGACAGTGTTGAAGCTGATGTCAGCACTGAAAGGATTTCCCCTCGAATTCCAGTTAAGGAGACATGTTTGTCCAAGCAATTGCCGAAGGAGGTGACCCGTGTGGAGATTAATTCAGGTTCCTTGAGTGTTCCTCTGCATGAAACAGAATTCACAGAGTCATATCCTACTCACAAGTCAAGTGTATCGATCGAAGCAACTAAGGTGGTAGATGCGACTCTTGTAGCATCCCCTTTATTGGATGCAATTGCTAAAACTGATAAGCTTCATGATGACAATCACGACTACCAACCATCTAATGATCCTGATCATGGAGATTCAATGCAAGGTGATGATGGTGCTGATTCTCAAAGTGGAAAGCATAACAAGAAAAACGAAAATCAAGGTTCACCTGCATTGAAAGTTGACGAATTTGGGAGAACGGTGAGAAAAAATGCG------AGTGACAGTGACTCTGAAAAGGAGCATTACAGCGGGAGACGTCATAGAAGAGGCCGCAGTCGATCTCCCTTGGACCGGAGGAGGAGTCGCAGTCCACGAAGAAGAAATGAGAAACGAAACCGATCTCGCAGCTGGTCTCCCAGAAAGCGAAGAAGCATGAGCAAAAGCAGGTCTCCACCTTCTGTTAGGCACAAAGGTGAATTTTCTGGGGAGAAATCGAGACGCGATAGAGATCAGACTGATTTCTGTTTTGACTTCCAAATAGGAAGATGCTACCGCGGAGCATCGTGTCGTTATTCACATCATAGACAGGGAGATTCAGTCAGGCGCTATAGAGGTAGACAAGATTGGAGCATAAATAATGCACGTGGAGATTCTCGTTCAAGGAGTGAGTATGACATCGTAAAAGAGGATGCGACTAGAACAGAAGATAGCGGCTGGGATAAAAAAACTGTTGCTGGCACATTAGTAGAGGATGCTCTGCCTGTTAGTTTGGTAGAATCTCAAGAGGAAATTGAGCGAAGGCCTGAAAAGGAGCCAGCGTTGGAGACACCTATTGTTCAAGATGCTGAAATTTCTACGCTGCCAGATGAGGTTAGCCGGCCCTTGTTGACATCTGAACTCTCTATCCCTGAAATGTCTGGTAGACCTCCAAGTGAACCTTCCCTAGCAGATAATGCAGTTCATCACCCACCTCAAGCTAATGGCAATTCCCTGGAAAACACTGGCCATTCTCTTCTTGCTGAGCATCATCTACCTGTCCAGACTATAGATACAAGACACTCTTTACCTCCTGTTAGGGAGCTGCGTCCCCCACAATTGTCTGGTGATAATTACCAGTTTCAGCATTCTCAATTACCCCCTCCACCACCACCACCACCTCCCCCAGCCACTGCCTCAACCGAAATGTATCGTCCTTATCAAGCTTCAACAAGTGGTCAACATTCTCAGTCACCAGCTATTTCAAAACCTTCTTGGACTTCGCTTCCTCTACCACCACCACCTCCTCCTAACATGCATGTCTCTACTTCTACAACTGTTACCCAGGGTTTCCACCCCTTGCAGTTTCAACAATATCCAGGTCCCGGTCCTGGACCTATGATGAGGCCTTACCAGCAGCATGGACAACCTAGTAACTCTCAGGCCATTGACCAGCATCTTCCAACATATCCTCCAACAAGCGAATTCCATCGACCTCCCCTTCACTCTGACGGTATAAGGCCAAATCAACAATTTGATTACCCCAATGCTTTTAGAGATGAACGCTTTCCTCAATCTTCAGTGCTTGATGGAAACCGTTATCATCACACTACTCCTCTACAACCTTTGCCAATGCACCCGCCTCGAGATGAACGCCATTATCCTCATCAGGTCCACGACACTTCTCGGAACTTCCAGTCACTACCACCGAATCAGTCATTCCCAGATGGTAATTCATATCACAATCCATTCCCAAAGGAGCATACTTTCACACAGCATCACCCTCAACAACCAACCTTTGGCTTGCAATGGCCTACTGAAAATGCAGGTCCATCTATGAGGAAATACCCATTGGGCGTTGATGATGAAAGTATACCACCTCTCTTGGCACCAAGAATTACAGCACACTATAATCCATATGCATCTACATTTGAAAAAGTACCTATACCAGATTCTAACAAGTCCCAGGGCCAGATTTTACCCAAGTCAGGCAACCCGTATGACCCATTGTTTGATAGTATCGAACCATCATCAAAAGCTGATAAAATAGAAGAAAAAGAAGCTACATCTCCAGAAAACGATGAATTTGGTGACACCGGTGCTGTGGAGAGTGATGACAGCCCAGATGATGATAAGCCCACGGGAGACGTTGAAATTGATGATCAGGCCCGTTCGAAAAACAAGATCAAAAAAAGTAAGAGTAAAATCAAGGGAAAGGATTCTCGGTCGACTAAAGTTTTCAAAGTTGCCCTAGCTGAATTTGTCAAGGATGTTTTAAAACCTTCATGGCGACAAGGCAATATGAGCAAAGAGGCATTTAAGACGATTGTGAAGAAAACTGTAGATAAGGTAGCGAATGCCATGAAGAGTCATCAGATACCCAAGGGACAAGCCAAAATTGATCAATATGTGGAGTCGTCGCAAAGAAAGTTGACCAAACTTGTAATGGGCTATGTGGATAAGTATGTGAAA

9333 Unigene28331_R-tanguticus CL1808.Contig2_R-bungei

---------------------------------------------------------------------------------------------------------------------------------------------------------------------------------------------------------------------------------------------------------------------------------------------------------------------------------------------------------------------------------------------------------------------------------------------------------------CATCCCGCTGATAAAGAAAACCTATCAGAGTCCAAAACATTCCTGTCAGCGAATGCAATGACTGGAACCTCTGGTTTGAGCAAGAAAAAGGAAGGGGTTGACTTGCAATCACATCCTGGCTCCTCGAAATTATCACAAGTTTATACTCAATCTAGGGTATCCAAGGGTGATCCTTTAATTGTGGAGCCTCCATTCGAAATAAAGAGAACGATGGATAAAATTGTTGAGTTTATTTTGAGAAATGGCAAAGAATTTGAAGCAGTGCTTATAGAACAAGATCGCACCAATGGACGATTCCCGTTTCTTCGTCCATCCAACCTATACCACTCATATTATCTGAAGCTAATTGAAGAGTCAAAGCAAGGTGGTAAACAGTCTTCTCTACCCAAAGACATTGAGACTTCATCGAACGGATATTCTAGTCATGATTTGGAGCACGACCCAGATCAGAAAGAGAAGTTTAGGATGGTTATTACAAGTTCAAAG---GATGCTCAAGAACCAACAGCATCACCCAAGCAGTCCACCCAACAACACCAATATGGTATGAGTGTTGATACAGTTGCAGCCATCCTTCAGGCAGCAACTGGAAGAGGGCCGAAGACCAAAGCACCCTTGGTTGAGCAAGGAATTGGTAGTGATGATTCAAGCTTGGGCACTCTATCTCCCAGTGCTATAGGTTTGAGTGACGTGTCAGTTTCCAAGAAAAGAGACCTTGCTAGTGAGGCCGACTCCTCTGAAGTGGGTGTGGGGTTAACAAAAGAGCAGAAGCTAAAGGCGGAGAGATTGAAGCGTGCAAAAATGTTTGCAGCCATCATAAAGAGCGGTGGCACAGTAGCTTCGCCCCAGACGAATGATGCAGAACCGCCAGAGACTGAAACAAAGGATAGTGGTTCGTTGGAAATCGTTACCAGAGAGAGAGAAGGTAGTTCTGCTCCAGTTGGTTCCCCAGATAAACACAGAAAATTGGATTCTGAGGATAATAAGGAGAGAAGATCAGGGAAAAGGCACCATCATAGGTCTAGAACAAGGGTAGATGATGACGGAGACGAAGAAAAGCACTCCAGGAAGAAACATCGTTCTCGTCATGTGTCTTCACACAGAGATGAAAATAGAAGAAGACGACACTCATCTTCCAAAGACAGAGAATCTGAGTATTATAGGCGCAGGTACCACGGCAGCTCTTCTTCTGAGGATGAGCGACATCATCACAAAAGTAGCAGGTCTTCTTCGATTAGGCCTAGTAGAGAAAGAAAAACCGAGCCTGAGACTAGTAGAGGAATGGATAGTTCAACAACTCCAGCAGCTCAACCACCCACCGGTGTTACTGAGATATCTGATGATTTGAGAGCAAAAGTTCGTGCGATGCTGTTAGCAACCATG

TTGTTGTCTTTGCAGCCCCCTACTGAGAAGTTACATCAAATCATGGCGAGGACTGCGATATATGTCAGTGAGCATGGAGGACAAGCAGAGATTGTCTTAAGGGTAAAACAGGGGAACAATCCTACATTTGGATTCTTGATGCCCGATCATCATCTTCATGCATATTTCAGATTCCTTGTGGATCATCCAGATGTTTTAAAGTCTGATAGCAACGATAAAGCAGAAGTATGTATGAAAAATGAGAAAGAGCAGAAACAGGCAACTGTTGCAGGAGAGGCATTGTCCTTGCTTGGGTCTGTATATGGTTATGGAGAGGACGAAGATGGTGCAGATGCGAGGTTCTCAGAGTCTGATAAGGCCACTAATAAAGAAAGTGCTAGTAGTGGCCTTCTTCACCATGGATCAGTGCTGTCTGTAGTGTCCGGGTCTTCTGCAGGCAAGAATACTCATTCCACTGATAAAGAAAACCTATCCGAGTCTAAAACAATCCCATCTGCAAATGCAATGACCGGAACCTCTGGTTTAAGCAAGAAAAAGGAAGAGGATGCCTCACATTCACTTTGGTCCTCTTCAAAATTATCAAAGGTTCTTGCTCAATCTAGTGCATCGAAGGTTGATCGTTCAATTGTGGAGCCTCCATCTGAAATGAAAAGAACGATGGATAAAATTGTTGAGTTCATTTTGAAAAATGGCAAAGAATTTGAGGCAGTACTTGTAGAACAAGATCGCATTAATGGACGATTCCCGTTTCTTCATCCATCCAACCTATACCACTCATATTATCTGAAGCTAGTTCAAGAGTCAAAGCAAGGTGGTAAACAGTCTTCTCTACTCAAAGACACTGAGACTTCATCGAACGGAAATTCTAGTCATGATTCGGCGTACGATCTAGATCAGAAAGAGAAGTTTAGGATGGTAATTACAAGTTCAAAGAGGGAGGCTCAAGAATCAACAGAACCACCCAAGCAGTCCACCCAACAA---CAATATGGTATGAGTGTTGATTCAGTTGCAGCCATCCTCCAGGCAGCAACTGGAAGAGGGCCGAGGACCAAAGCACCCTTGGTTGAGCAAGGTATTGGTAGTGATGATTCAAGCTTGGGCACTCTATCTCCCAGTGCTATAGGTTTGAGTGATGTGTCAGTTTCCAAGAAAAGAGACCTTGCTAGTGAGGCTGACTCCTCTGAAGTAGGGGTGGGGTTAACAAAAGAGCAGAAGCTAAAGGCGGAGAGATTGAAGCGCGCAAAAATGTTTGCAGCAATCATAAAGAGTGGTGGCACAGTAGCTGTGCCCCAGACGAATGATGCAGAACCGCCAGAGACTGAAAAAAAGGATAGTAGTTCATTGGAAATCATTGCGAGAGAGAGAGAAGGTAGTTCTGTTCCAATTGGCTCCCCAGATAAGCACATAAAATTGGATTCTGAGGATAATAAGGAGAGAAGACCAGGGCAAAGACCCCATTATAGGTCTAGAACAAGGGTAGATGATGACCAAGATGAAGAAAAGCACTCCAGGAAGAAACATCGGTCTCATCATGTGTCTTTGCACAGAGATGAAAATAGAAGAAGAAGACACTCATCTTCCAAAGACAGAGAATCTCGGCATCATAGGCGCAAGTACCATGACAGCTCTTCTTCTGAGGACGAGCGACATCATCACAAGAGTAGC------------------------------------------------------------------------------------------------------------------------------------------------------------------

899 CL2996.Contig2_R-tanguticus CL5323.Contig1_R-bungei

---------------------------------------------------------------------------------------------------------------------------------------------------------------------------------------------------------------------------------------------------------------------------------------------CTTGTGGGTTCATGCAATGGGTTGCTATGTTTTACCAATCACTCGAAGCCTTATCAGGTGTATGTTTGGAATCCGTGCACTGGAGAAAGCATGCAGTTTCCGCTTAATCCATCTCAATACAAAGATACTTATTACTCTGGTTTTGGTCACGATCCTACCCGTGACGAGTATAAGCTGATGACAATGTCGGATGAAGGAGTGAATGAGGTGTGGACTCTTGGGTCCAATTCATGGAGAAATGTTGGAGTGGCCCCATTCGTGCCACTGGCCTCGCGTAGTGTTTTTGTGGATGGAGCTCTTCATTGCCTATCCTCCCGTTCAACTGTTATTGGAATTGGTTCTTTTTATGTGGCAAAAGAGGAGTTTCAATTTGTTCCAATACCTAGGAAGGTTGTCGTCAATTTTTCACTCATGGGCGTGTTG---AGAGGTCGTATTGCTGTGGCTTGTGCAGCCAATGTTTCTTCATTTGATGAACTTGATATATGGGTAACCGAGCACTATAGGATAGAAGAGTCGTGGACGAGA------------------------------------------------------------------------------------------------------------------------------------------------------------------------------------------

TCCGAGGATACTTCTTTAGACATCTTCTCCAGATTACCTGTCAAGGCGGTGATGCGATGTAGGTGTGTGTGTAGATCATGGCGCGTTTTGCTCTCCACTCCTCATTTCATCAATATGCATCTCCAGTGTTCATCTTCCAATCGTTCGTGTAGACGTGATATTGTCCTTCTTGACACCCGTGTAAGGGACATGCCCAAGTATGCTCTTACTCTTGTGGAAAATTTCTGGTCCCGAGACGAGAGATCGAGACTTCTTCTAATGCCTTTACAACTGACTTCTGTAGTTCTTGTGGGTTCATGCAATGGGTTGCTATGTCTTACCAACCATTCAAAACCTTATCAGGTGTATGTTTGGAATCCATGCACTGGAGAAAGCACACAGTTTCCCCTTAATCCATCCCAATACAAAGATCCATATTTCTGTGGTTTTGGTCACGATCCTACCCGTGACGAGTATAAGCTGATAACAATGTCTGACGAAGGAGTGAATGAGGTGTGGACTCTTGGGTCCAATTCATGGAGAAATGTTGGCGTGGTTGCTTTCACGCCACTGGTCTCGACTAGTGTTTTTGTTGATGGAGCTCTTCATTGCATATCCTCTCATTCAGGTGTACTTGGAATTGGTTCTTTTCATGTGGCAACAGAGGAGTTTCAATTTGTTCCAATACCTAGGAGGATCTTCAACGAAATTTCAATGTTGGGTGTGTTAGGAGAAGGTTGTATTGCTATGGCATTTCCAAGGAACGGTTTTTTAAACGTTGAACTTGATATATGGGTAAACAAGGACTATAGGATGGAAGAGTCTTGGACGAGACAATATGTATTGAGCATCAACTTTGTACACCATATAGAGATTTTGTATGACTGGGAATTACTCATTGATCATGCAGATAAGGTTGCTGCTGCTGCTCCTGCTTCTGTGGATGAAAATCTGGCACTTGAAGGGGTTATCAAGATCACTAGCGCATATGTTCACGTTGGAACTCTTGTTTCGCCTATA

5292 Unigene19589_R-tanguticus Unigene20679_R-bungei

---------------------------------------------------------------------------------------------------------------------------------------------------------------------------------------------------------------------------------------------------------------------------------------------------------CGTTCAAGAAAGCCTGTGGAAACCCCATCATCCAATGCTGCCTCTGGTAGCACTAGCATTGCCAACTTCCAGAACCTGCCCTTGCAGGGAATGGGAATGTGCAACCCATCATCCCAGTTACAATTA------GACCCCGTCTCCTATGAAGCTGCAACCAAAGGATACAGGTATCATCAAGGAATGAAACAAGAAGGAGATGACCATTGTCTCTTAGCTGATGCTTGTGTTAATGCAAGAGGCGTGGGGATGGGCACTTGTGTAGAAAGCAACTGGCGTTTGATGCCAACCCCAGTA---TCCTCAATTTCTCAGTCAAAACCGAGGATGCAGTCAGATTATTCCCAGATGCAAACAGTCCAAGATGTTAGGCAAGCAGGCATGACGCCTTCTGTATTGTCAAAGCAGCATTGCTTCTTTGGCGACGAGTATGGTTTAGCAGAGCCTGCGAAACAAGAGGGACAGCCTCTTCGGCCTTTCTTTGATGAATGGCCTAAAATGAACAGAGATTCATGGTGCGACCTTGACGAAGAGAGATCCAACCGAACA---------ATTTCAATACCAATGGCTTCCTCTGACTTCTCTGCCACAAGTTCGCAATCGGCCAATGATGAT

CCTTTCACCCCTTCTCAGTGGCAAGAGCTGGAGCATCAAGCTTTGATCTTCAAGTATTTGATGGCCGGTTTACCAGTTCCTCCGGAGCTCGTCATCCCCATTAAGAGGAGCTTCGACTCCATTTCCCCTAGGTTCTTCCACCATCCCACAATGGGGAGTTACTGTTCCTTTTATGGGAAGAAACCGGACCCTGAACCAGGGAGGTGCAGGAGGACCGATGGGAAGAAATGGAGGTGCGCCAAAGCTGCTTATCATGATTCCAAGTACTGCGAGCGCCACATGCACCGAGGCCGCAACCGTTCAAGAAAGCCTGTGGAAACCCAAACACCCACCAACTCATCATCCACTGCTGCCTCTGTCAACTTCCAGGGAATGGCG------GCAATGGGAATGCGCAACCCATCATCCCAGTTGCAAATGCAAATCGACCCCCTCTCCTACCAACCTGCTACC---------AGGTATAATCAAGGAATGAAACAAGAAGGAGATGATCATGGTCTCTTAGCTGATGCTTGTATGAATGCAAGAGGCGTGGGGATGGGTACTTGTGTAGAAAGCAACTGGCGTTTGATGCCAACCCAAGTACCATCCTCATTTTCTCAGACAAAACCGAGGATGCAGTCGGATTATCCTGAGATGCAAACAGTCCAAGATGTTAGGCAAGCAGGGTTAGCACCTTCTGCATTGTCAAAGCAGCATTGCTTCTTTGGCGACGAGTATGGTTTAGCAGAACCTGTGAAACAAGAGGAACAGCCTCTTCGACCTTTCTTTGATGAATGGCCAAAATTGAACAGAGATTCATGGTGCGACCTTGATGAAGAGAGATCCAACCGAACTCAGCTATCAATTTCAATACCAATGGCATCCTCTGACTTCTCTGCCACAAGTTCACAATCGGCCAATGATGAT

9187 Unigene28053_R-tanguticus Unigene28352_R-bungei

---------------------------------------------------------------------------------------------------------------------------------------------------------------------------------------------------------------------------------------------------------------------------------------------------------------------------------------------------------------------------------------------------------------------------------------------------------------------------------------------------------------------------------------------------------------------------------------------------------------------------------------------------------------------------------------------------------------------------------------------------------------------------------------------------------------------------------------------------------------------------------------------------------------------------------------------------GATTCTATAGACAGTACTTCTCAGAGTGATACAACTAGTTCTTCTGGCTCGAGCTTCTATGGTCCATATGGTTCTAAAGTTTCTTACCAGTTCATCCCTAAATCAGATTTCTGGGAAGAAGCTCTTGGTTCAAATGAATTTGAGATTGATGTTTATGATGACTCTACTCAATCCAGTCTCAGCGAAGCCGGTGAGGATAATTTAGCTGACAGTAAGTTGCGGACATCAGAGACCTCCCATCAAGGTGTTCGCCACTTGCATGAAAAACTGAACCCTAGTATTCACTTGCCTGAGGCTACAGCTTCTTGTATTGTGTGGAAGAAATCAGCCAGAAGAATTATTTCGTCGGAACCTCTGGTTGCAGATACTTCCAAGTCAAAGAGTTCTCCAACAATGAGATCTGAAAAACCAACTTCTGTGAAGAATACAAGCCGTAGCACACATTTATTTGGACTAGGAGCTAGATCATCATCAGTTAATACATCTCGTGGCGATAAACTTTCTGGTGGTAGGGGACAATATGTTTCAGATATTGACTCCTCTGCAACTGCCACCATAAAGAAAATGCCTACTGGATCATCTGGAACTGTTAATTCCATGACGAATGTTGGTAATGGGTTAAAGACATCTGTGAAAAAAGCTGTTCAGCCATTGAATGTTTACAAGCACTCAAAACACTATGCTTATGGACTTGGAATGGAAACTGTTGAAAAGTACAAATATAAGATGCTTTTTCCGTATGAACAGTTTGTCGAACTTTATCACTGGAAGGATGTTGAGCTGCACCCATGTGGACTTAAGAATTGTGGAAACAGCTGCTATGCTAATGCGGTTCTCCAGTGCCTGACATTCACACGGCCTCTTGCTGCTTATCTTCTTCAGCGTTTTCATTCTAAAGCCTGTCCAAAAAAAGTCTGGTGTTTCATGTGTGAATTTGAGGGTTTAATTTTGAAGATGGACGGGAAATCCCTGTTATCCCCAGTTAGTATACTATCACAACTAAAAAACAACGGAAGCCATCTTGGTGATGGACGACAAGAAGACGCTCATGAATTTTTGAGATATGTGATTGATACAATGCAATCTGTTTGCTCCATTGATACTGGGGGAATTGGTGTGGTCCCTTCAGCTGAAGAAACATCTCTCATAGGATTGATATTTGGGGGTTACCTTCAATCTAGGATAAGGTGCATGAAGTGCCACGTAAAATCTGAAAAGCATGAAAGAATGATGGATCTTACTGTTGAGATACAAGGAGGGATTGGAACTCTCGAGGAAGCTCTCAAACAGTTTACAGCCACTGAAATCTTGGACAAGGAGAACATGTACAATTGTCTCAGATGCAGGTCATATGAAAAGGCCAGGAAGAAGTTGACAATATTTGAAGCTCCTAATGTCCTTACAATTGCTCTTAAACGATTTCAGAGTGGACAAAATGGGAAGATTAATAAACCAGTGAAATTCCCGTTGGTCCTAGATCTATGTCCTTACATGAGTAGGACAACAACCGATCGTTCCCCTGTCTATAGTCTTTATGCCGTGGTAGTTCACTTGGATACAAACAATGCAACTTCATCAGGGCACTATGTATGTTACGTCAAGACTATCCAGGGGAAATGGTTCAACATCGATGACAGCACTGTAAAGCCTGTGGACTCTGAGACAGTCTTATCTCAAAAAGCATACATGCTCCTCTATGCAAGAATCTCGCCACGGGCTCCTAGTTCGGCACGGACTGCTAAAACTGTCAAAAACCCCATCAGCACTACCACAGCTACAGGCCCAAGAGGTGTTACAGAAAGGCCCGAAGACTATCCTTATTGGACAACGCTGGATCCATACAATAGAAGGTTTGGTCCAATGCAGGTAGATTCGTCAAGTGATAATTCTTCCCTACTTAGCTGCTCAGACGAAGGATCTTGTAGTACTGAAAGCACCCGGGATTCAACTAGTGATGATTTTCCTGAATACATACATACGACGGATTCATATCGGGTGTTTGAAAGTCCGCCCATTTTGGATTCTAACACTAGTAAAAATTGTAGTGATTTAACTTATAGTGAAAATAATAGTAGTAATAACGAGACTGACAAGGAGAGATTAGGACATAGTAGGCTTATAGATGTAAATTCAGGTGTACCCCAGAGGAGAATATCGCGAGAGACTAATGCA

CTTCAATTAGGGTTTCAGAATTCGGTTTTGATTTTAGTTTTTTTATCTGGTTTAATAACGAGTTTTGTGATTCGTTGGAAATGGAACAAATCAGTAGAGAGAAAGAAGGAGGTGTTGAAGCTTTTGGCTATGGCGTCAGAGGAAGCAGCCAGAGTAGAGATTGAAGCTGCTGTTGAATATGGTTCTGTTTATGTTGTGAGCAGGCAATCTCAATGTGCTGTTTGTTTTTCTCCTACGACTACTAGGTGTTCTAGATGCAAAGCTGTTAAATACTGCTCGGGTAAGTGTCAAATTGTACACTGGAGACAAGGTCACAAGGATGAATGTTATCCTGCAAAGAATACCTTTCAGTACAATGGTCCTGATACTGATTCTGACCAAAGGGCTGCCTCTCAAGGAATTCAAAATGAGGGCAATGTCAATAGTGTTGAGGTCGAAGAAAGCTGCAAGGATAAAGTGGGTGAAACATACCTCAATGGAGTTGGTTCTTCTATATCCAACAATTCTGATCAGAAGGTTGGCGGTGAGCTTGGGGAAGCACCTGCTGTTTCTGATAGCGTTAGTACATCATTTGTTGAGTCCCTTACTGTCACTGCGGAAGAACCTTTAGTGGACACTATTCTCCCTGAACCTGTTTCAGACACTCCTGTTGGATTAGAAAAGTCTGTGTCCAGAGATATGTCTCAGGACGTGCCTGATATAGGAGGTAGTGATAATGATGTTAAGCTAACTAAGTCACCATCTCCAGAGCATGAACCTTTAGTTGATGCTTCTCCTGTAGACTCAGTTTCAAACTCCAATGATTGTCTAGATCCGCCTCTGTTTGATGATACTGCCGAGGATGTTGAAAGCAAAACAGATTTTAATATCTCAAATCTAACTGAATCGCCAAGCTCATTGTTCAGTACATCTGATGATTCTGTAGACAGTACTTCTCAGAGTGATACGACTAGTTCTTCTGGCTCAAGCTTCTATGATCCATATGGTTCTAAAGTTTCTTACCAGCCCATCCCCAAATCAGATTTCTGGGCAGAAGCTCTTGGTCCAAATGAATTTGAGATTGATGTTTATGATGACTCTACTCAATCCAGTCCCAGTGAAGCCGGTGAGGATAATTTAGCCGACAGTAAGTTGCTGACATCGAAGCACTCACAGCAAGGTGTTCGCCACTTGCATGAACCACTGAACCGTAGTATACACATGCCTGAGGCTACAGCTTCTTGTATTGTGTGGAAGAAATCTGCCAGAAGAATTCCTTTGTCGGAACCTCTGGTTGCAGATACTTCCGAGCCGAAGAGTTCTCCAACAACTAGATCTGAAAAACCAACTTCTGTGAAGAGTACAAGCCGTAGTATACTTTTTTTTGGACTAGGAGCTAGATCATCATCAATTAATACATCTCGTGATGATAAACTTTCTGGTGGTAGGGGACAATCTGTTTCAGATATTAACTCCTCTGCAACTGCCACCATACAGAAAATGCCTACTGGATCATCTGGAACTGTTAATTCCATGCCGAATGGTGGTAGTGGGTTAAAGACATCTGTGAAAAAAGCTCTTCAGCCATTAAATGTCTACAAGCACTCAAAATACTATGCTCCTGGGCTTGGATTTGACATTGTTGAAAAGTACAAATATAAGATGCTTTTTCCGTATGAAAAGTTCGTCGAGCTTTATCATTGGAAGGAAGTTGAGCTGCACCCATGTGGCCTTAAGAATTGTGGAAACAGCTGCTATGCTAATGTGGTTCTCCAGTGCCTGACATTCACACGGCCTCTTGCTGCTTATCTTCTTCAGCGTTTTCACTCTGCAACATGTCCAAAAAAAGACTGGTGTTTCATGTGTGAATTTGAGAGTTTAATTTTGAAGATGGACGGGAAATCCCTGTTATCCCCAACTAGTATACTCTCACAATTAAAAAACAACGGAACCCATCTTGGTGATGGACGACAAGAAGATGCTCATGAATTTTTGAGATATGTGATTGATACCATGCAATCTGTTTGCTCCATTAATACTGGGAAAATTGGTGTGGCCCCTTCAGCTGAAGAAACATCTCTCATAGGATTGATATTTGGGGGTTATCTTCAATCTAGGATAAGGTGCATGAAGTGCCACGTAAAATCTGAGAAGCATGAAAGAATGATGGATCTTACTGTCGAGATACAAGGAGGGATCGGAACTCTTGAGGAAGCTCTCAAACAGTTTACA------------------------------------------------------------------------------------------------------------------------------------------------------------------------------------------------------------------------------------------------------------------------------------------------------------------------------------------------------------------------------------------------------------------------------------------------------------------------------------------------------------------------------------------------------------------------------------------------------------------------------------------------------------------------------------------------------------------------------------------------------------------------------------------------------------------------------------------------------------------------------------------------------------

2176 Unigene7071_R-bungei CL5898.Contig1_R-tanguticus

------------------------------------------------------------------------------------------------------------------------------------GAGAAAATTGATTCAGCTATGCTTATTCTTCGTTCAAGTCCAACGACAGAGGAATGGAATAGAATAGTAGCAGCCACCAAAAATGGTGTTGCACTGACCGGCAGCATTGCGTCCAGACCAGCTGGACCAATTATTGGCCTAGTGGATATTGGCTGCTGTGATGATGCTTATTTGTTTATAATGTCCCTTCCAGGTGTTAGTAGGGACGCAGGAAATTTCAGTTGTGAGATTGAAGCAAATGGGAGGGTGATAGTTGAAGGAGTCACTCTGACAGGCCTGAAAAAGGTTTGCAAGAGCTCACAGATTTTTGAAATGCAGACTCAAAATCTTTGTCCACCTGGACCTTTCTCGATCTCGTTTCAGTTGCCAGGCCCAGTCGAAACCAGAGGATTTAGTGGTAATTTTCGAAATGATGGGATATTTGAAGCTATTGTCCTAAAGCGA

AAGGGCTGCAGTTATCTGTCCTCAATGACATCGAAAGATCCTCTAGGACACCTACCTAGTAGAAGGTCTCCTAGGCAGAAAAACAAAGAACTGAATCACTTAGAAGCCAATGAAGCATCGCATAAACCTGTGGAGAAAATTGAATCAGCTATGCTTATTCTTCGTTCAAGTCCAACTACAGAAGAATGGAATAGAATAGTAGCAGCCACCAAAAATGCGGTTGCGCTGACCGGCAGCATTGCGTCAAGACCAGCTGGACCAGTTGTTGGCCTAGTGGATATTGGCTACTCTGATCGTGCTTATTTGTTTATGATTTCCCTTCCAGGTGTTAGCAGAGAT------GAAATTTGTTGTGAGATTGGAAGAAACGGGAAGGTGTTGGTTAAAGGAATCAGTTTGACAGGCCAGAAAAAGGTTTGCATTAGCTCACAGATTTTTGAAATGCAGACTCAAAATCTTCCTCCTCCTGGGCCTTTCTCGATCTCGTTTCAGTTGCCAGGCCCAGTCGAGCCCAGAGGATTTATTGGTAATTTTCGAAATGATGGGATTTTTGAAGGTGTTGTTCTAAGG---

9341 Unigene24041_R-bungei Unigene2834_R-tanguticus

CGTTCAAGAAAGCCTGTGGAAGGCCAGACAGGCCATGTTGCTGCTGGACCAGTTACGGCGAAAGAGATGCCAACACCTTCTCCTTCGCCTGCTTCAGTATCCACTGCTGGTGGAGCATCCGCTCGCCACAGTGTTGCACGTCACCAGATTGAGAGCTTGCAGTCTGCAGGGCTCGATGTTTCATCAAACTCTCACATGAATGGGATTTATCCAAACAATGGAGGCCTATCCATGCCTCCTCCATCCATAAACATGAAACCTAAGGACACCCCAGTTTCAGTTACGAAGCCACAAATCTCATGTAAAGAGCCCACCGGAACCGAATTTGGATTAGTTTCCTCTGATTCGCTTCTAAGT------ACTATACAAAACAGCTCCTACAATGATAGCAGAAAGTTTGGTATATCCTCTGACCTCAACAACCAAGAGACACGATTGCAAGATCCCTTTCGCCATTTTATTGATGATTGGCCCAAGAATCCACCTGAAAACTTCCCTACTACCTGGCCTAACATTGAGGAAACCCAGTTAAAACAAACTCAGCTCTCCATATCAGTCGCAATGTCCTCAGAATTCTCATCATCGTCTTCCTCACCAGCTCTCTCACCCCTTAGGTTGTCACAGGAGTTTGATCCAATTCAGATGAGTTTAGGGGCGGATAGTTTTCCAAAGGAATTGACCCAAAAGCAAGTGAACTGGGTACCTATAGCTTGGGAGTCTTCCGCTTCCATGGGCGGACCATTAGGGGAAGTCTTGAACCGCACCAATAGCTGTATATCTGGGGATGGAAAGAACTCCTTGGAAGCTCTAGATCTCATGACCGAGGGGTGGGATGGTGGCCTTCAAATGGAGGACTCTCCCACTGGTGTCCTACAAAGGACTTCATTTGGGTCCCTTTCTAACAGCAGCACTGGCAGTAGTCCTAGAGCTGAAACCAACTTCCATCTAGGAGGAAACTTGTGTGACACTTTCCTTCCAAGCACTCTGCTAAATTCTTCTTCCATG

CGTTCAAGAAAGCCTGTGGAAGGCCAGACAGGCCATGTTGCTGCTGGACCAGTTACGACGAAAGAGATGCCAGTGACTTCTCCTTCGCCAGCTTCAGTAGCCACTGCTGGTGGAGCATCCAATCGCCACAGTGTTGCACGTCACCAGATTGAGAACTTGCAGTCTGCAGGGCTCGACATTTCATCAAACTCTCACATGAATGGGATATATCCAAACCACGGAGGCCTATCCATGCTTCCTCCAGCAATAAACATGAAACCTAAAGACACCCCAGTTTCAATTACGAACCCAGAAGTCCCATGCGAAGAACCCACTGGAACCAAATTCGGATTAATTTCCACTGATTCGCTTCTAAGTACTACTACTACACAAAACAGCTTGTACAACGATAGCAGAAAATTTGGTATTTCCTCTGACCTCAACAACCAAGAGCCACGAGTACAGGATCCCTTTCGCCATTTTATTGATGATTGGCCCAAGAATCCACCTGAAAACTTCCCTAATACCTGGCCTAACATTGAAGGAACCCAGTTAGACCGAACTCAGCTCTCCATATCAGTCCCAATGTCCTCAGAATTCTCATCATCGTCTTCCTCACCAGCACTCTCACCCCTGAGGTTGTCACGGGAGTTTGATCCAATTCAGATGAGTTTAGGGGCGGTTACTTTTCCAAAGGAACCGGCCCAAAAGCAAGTGAACTGGATACCAATAGCTTGGGAGTCTTCCTCTTTCATGGGAGGACCTTTAGGGGAAGTCTTGAACCGCACTAATAGTCGTATATCTGGGGATGGAAAGAATTCCTTGGAAGCTCTAGATCTCATGACCGAGGGGTGGGATGATGGCCTTCAAATGGAGGACTCCCCCACTGGTGTCCTACAAAAGACTTCATTTGGGTCCCTATCTAACAGCAGCACTGGCAGTAGTCCTAGAGCTGAAACCAACTTCCATCTAGGAGGAGACTTGTGTGACACTTTCCTAACACGCACTCTGGTAAATTCTTCTTCTATC

4539 Unigene16314_R-tanguticus Unigene33218_R-bungei

CTGGGTGTTTCAATTGGCGCGGTTGACAATATGAAATCGGATAATACATGTCTTGAAACTTTTCCAATTGAACAGTCTGAGGTGACGGTGGTAGGTTTGAATCAGATATATGCCGCGGATGTAAAGGTTCGAGATAATGTTGTGATGGAGAACATGAAAGATCTAGTGCCAATTACAGCTCTGGAATCCTTGCCACCAGACACCCCGAAACAATTGCACAAGGTATCAGATCAATCCCCAGAACTTAATTGTCTTGTAGCTGTACATAATAACGTGCGGAAGAATTATCCCCCTCGAAGAAGAATTTCAGCTTCACGAGATATTCCATTATGTTGTACCACGAATGCTTCATGTTCTATCCAAGAAGTTGATTACTTGATAGACGTCGGCACTAGTGATGATGACAAGGTTTGTGGGGAGAACATAATAGAGGTGCTTGATAAAAGTGAATCAAAGAAAACAACAGAGGGAACTGATGAAATCGCACACGAGAGCATGCTGCAG

---XGTGTTTCAATTGGCGCGGTTGATAACTTGAAATCGGATAATAAATGTCTTGAAACTTCTCCAATTGAACAGTCTGAGGTGACGGTGGCGGATTTAAATCAGGTCTATGCCGCAGATGTAACAGTTAGAGATAATGTTGCGATTGAGAACGCGAAAGATCTAGTGCCAATTACAGCTCCGGAATCCTTGCTGCTAGACACCCTGAAACAATTGCACAAGGTATCAGATCAATCCCCAGAATCAGATCATCTTGGAGCTGTAGGAAATAATGTGCGGAAGAAATATCCTCCTCGACGAAGAATTTCAGCTTCACGAGATATTCCTTTACGTTGCACCACCAATGCTTCATATTCTATCCAAGAAGTTAATAACTCAATAAATATCGGCACAAGT---GATGACAAGGTTTGTGGGGAGAACATATTAGXX------------------------------------------------------------------------

6889 Unigene23699_R-tanguticus Unigene31329_R-bungei

------AAAGTTAAGCTCGAAAGAATAAAAACACTATGG------GCCGAACTTGAATCAGAATGTGTG---------------------------------------------------------------------------------------------------------------------------------CAGATGATGGTTCTAAGGGTATCCATGCATTGCAATGGTTGTGCAAGAAAAATCCAGAAACACATTTCAAAGATGGATGGAGTTACTTCCTTCCAAGTAGAGTTGGAAAACAAGAAGGTTGTTGTAATTGGAGACATACTTCCCTTTGAAGTATTGAAGAGTATTTCAAAGGTCAAGAATGCAGAGATATGGACATCCCCT

ATGGGGAAGCTGAGTTTTGGCAAGGTGTTGGATTGTTTTTGTCTCTCTTCTGCATCAAATTCATGTTTCTGCATAAACTCGTTCGAAGATGAAGATTTTAATGAGAGAAAGCCACTTATGGGAACAGATGAAAGTCGTTTTGTGAGATTAAGTGATGTTGTTAATACAAAGCAGACTCTGGCATTTCAGTTAAAACCCAAGATGGTCGTTCTAAGGGTATCCATGCATTGCAATGGTTGTGCAAGAAAAATCCAGAAACACATTTCAAAGATGGATGGAGTTACTTCCTTCCAAGTAGACTTGGAAAACAAGAAGGTAGTTGTGATAGGAGACATACTTCCCTTTGAAGTGTTGGAGAGTGTTTCAAAGGTCAAGAATGCAGAGATATGGACATCCCCT

3143 Unigene26897_R-bungei CL8124.Contig1_R-tanguticus

ATGAAGATGAAGATGAAGATGAGTTTGGTATTATTAATTTTCTTTGTT---------------GTTGCAGAAGCATCAGAAGCATCAGAAGCAGCTAGTAGAGATGTT---GTGTTTGTTAAGAAAACAGTTTCATCTCACAACATCGTCATCTTCTCCAAATCATACTGCCCGTACTGTAAGAGAGCTAAATCTGTCTTCAAAGCGTTGAACCAGACACCATACGTGATTGAACTAGACGAGAGAGAGGACGGATGGGGTTTACAGAGCGCCCTCGGAGAAATGGTGGGCAGGCGTACAGTTCCGCAAGTGTTTATCAATGGTAAACACATTGGTGGTTCAGATGATACTGTTGAAGCATACGAAAGTGGGGAATTGGCTAAGCTCCTTGGCGTTGTTGAAAAAGATGATGAT

ATGGCAATGAAGATGAGATTGGCGGCAGCAGTCATATTATTATTAGTATCTCTCACACTGCACCTCCATGTTGTTTCAGCAACAGCAGAATCATCAACAGAAGATCTGGGAAGCTTTGTTAAGAAAACTATTTCATCTCACCAAATCGTCATCTTCTCCAAATCATACTGCCCGTACTGTAAGAGGGCTAAATCTGTCTTCAAAGCGTTGAACCAGACACCATACGTGATTGAACTAGACGAGAGAGAGGACGGATGGGGTTTACAGAGCGCCCTCGGAGAAATGGTGGGCAGGCGTACAGTTCCTCAAGTGTTTATCAATGGTAAACACATTGGTGGTTCAGATGATACTGTTGAAGCATACGAAAGTGGGGAATTGGCTAAGCTCCTTGGTGTTGTTGAAAGAGACGATGAT

9559 Unigene12931_R-bungei Unigene28753_R-tanguticus

XXTGATAGTGTTGAAGATGGTAGTAGAATAAGTAATGAAGAAACTAATTGTTCAATTTCTGAGCAAGAAAGCACTGAATGTAGCAGTTCACAAGGAAGTTCTTTCCAACAATCTGGCATAGTTGAAAAAAGCAGGACAGAAGAGGATAACACATCTGACTCTCTTAATGAGAGCTGCACCAACTTGGAAAGATCAGTTTGCAGAAGAACTTTTTGTGGAGCAATTGAGGAAGTGCTAAATAACACCAGCCAGGATTCTGGAATGGAGACTTTGACCCCGAACCCAGAATTTGATAGTTTATCAAACGGGAACATGGAGAAAATAAAGTCCAACAACGGTATCGAGAGTGCAAGGAATGAAGGGACAACCTCTAACTCCAATATGTCAACCAGGTTATTGAGTTACTTCCAGATAAAACCCAAGAAAGAAATAAATAAATCTCGAGAATTTGAAGGATTTAAAAGTCTACTGACCAGTTCATCTTCTACAACTAATAAGCTGGGATCTTCAGTAATCAGACGAATCCGTGAAGATGAAGTGACCAAGGATTTTCAGCAGGCTAAACAGTATCAAGCATTTCTTCTACGCCCCGGAGACCAGATCGTT

XXTGATAGTGTTGAAGATGGTAGTAGAACAATTAATGAGGAAACTAATTGTTCAACTTCTGAGCAAGAAAGCACTGAATGTAGCAGTTCACAAGGAAGCTCTTTCCAACAATCTGGCGGAGTTGAAGAAAGCAGGACAGAAGAGGAGAATACATCAGACTCTCTAAAGGAAAGCTGTACCAACTCGGAAAAATCAGTTTGCAGAAGAATTTTTTGTGGAGCAATTGAGGAAGTGCTAAATAACACCAGCCAGGATTCTGGGATGGAGACTTTGACCTCGAACTCAGAAATTGATAGTCTATCAAACGGGAACGTGGAGAAAATAAAGTCCAACAATGGTATTGAGGGTGCCAAGAATGAAGGGACAACCTCTAGCTCCAATATGTCAACCAAGTTACTGAGTTACTTCCAGCTAAAACCCAAGAAAGAAATAACCAAACCTCAAGAAATTGAAGGATTTAAAAGTTTACTGACCAGTTCATCTCCTTGTACTAATAAGCTGGGATCTTCAATAATCAGGCGAATCCGTGAAGATGAAGTGACCAAGGATTTTCAGCAGGCTAAACAGTATCAAACATTTCTTCTGCGTCCAGGAGACCAGATCGTT

6826 Unigene23553_R-tanguticus Unigene22152_R-bungei

------------------------------------------------------------------------------------------------------------ATATCAATGCTTCCAATTCTTAATCAATGGGTTGAAGAAGGTAATAACGTTACTAAAGGGGATATTGTTTCCATTATTAAGAGGCTTAAAGGCTTCAATCGCTTCAAACATGCTCTCCAGGTATCTCAGTGGATGAGTGATGAAATGTGTTTTGAGCTTCAACCAGGTGATATGGCTGTGCGTCTGGGCTTGATTTCAAGTGTTCATGGCATTGAACAAGCCGAGAAGTATTTTGGTGAAGTAGCAGAGCCATCCAAAATTCTCCCAGTTTACTATTCCCTTCTTGAATGCTATGCACACAACAATTCCACAGAAAAAGCTGAGTCCCTCATGCAGCAGATGAAGGAGATAGGTTTTGTCAACACGCCAGTTGCTTACAATATACTACTAAACCTCTACTATAAGCTAGGGCAGTACGAGAAACAAGACGCCTTGATACACGAAATGAAAGAGAGAGGAGTAGTTCCAGATACGTCTACCTATAGCATTCGGTTAGCTTCGTATGCAGACACGGCTCAAATCAACGATATGGAGAGTATTTTGAGGACCATGGAAAAGGAGCCAAAGGTGGTTCTGAATTGGCATACTTATGGCACAGCTGCAAATGGATACATGAAAGCTGGAAAGATGGATAAGGCATTAGAGTTTTTGAAGAAATCAGAGGAATTGGTCCCACGTGCAAAGAAAAAGTTTGTCTATCATTTTCTCCTTACTATGTATGCAAGCGCAGGGAAGAAAGACGAATTATACAGGATTTGGAATGTTTACAAGTCAACAGAGAAAGTGTATAACATGGGTTATCGTTGTATGATAAACTCACTTATAAAGCTGGGTGATATTGCTGCTGCCGAGAAGATTCTAGAAGAATGGGAATACGTCAACATTTCGTATGACTTTCGAATTCCGAATTTGTTGGTTGCTGCTTACTGCAAAAACGGGCTTGTGGATAAGGCAGAAATGCTTGTTCAGAAAGCGGTGGAGAAAGGGAAGGAGCCTCTTCCAACTACGTGGGAGATTTTAGCAACTACCTATAATGAATTTAATCAGATACAAAAGGCAGTGGAGGCTGTGAAAAATGCGTTATTGGCAAAACGGCCTGGTTGGAGGCCCAAACGGCCGACGTTGGCTTTCTGTTTGTTGTACTTGAAACAACAAGGAGAGGATAAGAAGATAGGGGAGCTTTTGAGATTACTTGGGGTACCAGTTCATATGTCGGGAGATGATTGTGAGAGATTGTTGGATTATATATATGGAGGAGAA

TATGAATCAACAAGAAACCAAACATCATCATCATCATCATTATTTCTAGCTAAGAAGAAGAAGGAGGAGAAATTGCAGAGTAGAATAATTCCAGCAGGGGAGCCATGGGTTTCAATGCTTCCAATTCTCAATCAATGGGTTGAACAAGGTAATAACGTTACTAGAGGTGATATTGTTTCCATCATTAAGAGGCTTAGAGCTTTCAAACGCTTCAAACATGCTCTTCAGATATCTCAGTGGATGAGTGATGAAATGTGTTTTGAGCTTCAACCAGGTGACATTGCTGTGCGTCTGGGCTTGATTTCAAGTGTTCATGGCATCGAACAAGCCGAGAAGTATTTTGGTGAAGTAGCAGAGCCATCCAAAATTCTCCCAGTTTACTATTCCCTTCTTGAATGCTATGCACACAACAATTCCACAGGAAAAGCTGAGTCCCTCATGCAGCAGATGAAGGAGATGGGTTTTGTCGACACGCCAGTTGCTTACAATATACTGCTAAACCTCTACTATAAGCTAGGGCAGTACGAGAAACAAGACGCCTTGATACAAGAAATGAACGAGAGAGGAGTAGTTCCAGATACGTCTACCTATAGCATTCGGTTAGCTTCGTATGCAGACACGGCTCAAATCAACAATATGGAGAGTATTTTGAGGACCATGGAAAAGGAGCCAAAGGTAGTTATGAATTGGAATACTTGTGCCACAGCTGCAAATGGATACATGAAAGCTGGAAAGATGGACAAGGCATTAGAGTTTTTGAAGAAATCAGAGGAACTGATCCCACGTGAAAAGAAAAAGTTTGTGTATCATCATCTCCTTACTATGTATGCAAGCGCAGGGAAGAAAGACGATTTATACAGGATTTGGAATGTTTACAAGTCGACAGAGAAAGTGTATAACATGGGTTATCGTTGTATGATAAACTCACTTATAAAGCTGGGTGATGTTGCTGCTGCCGAG------------------------------------------------------------------------------------------------------------------------------------------------------------------------------------------------------------------------------------------------------------------------------------------------------------------------------------------------------------------------------------------------------------------------

7469 Unigene24814_R-tanguticus Unigene21851_R-bungei

GCAAAAGAAGATGCACCAAAATGGGGGAATGACAAGCATGAAGGTCCTTCCCTACAGAGTGGCAGTAAAGGTGGCAATTTTGCAAGTCGTAATCCAGAGTCTCGAGATGTAGACAGGGGGCGTAATTTCCGGCCAGATGATAAGGACAAGAGATTGCCTAGAGGGGATGATGATAAGCGAAATGAAAGAAATCAGGACAGACGATCAGAAAAGTATGAGAAAGAAGCTAATCACAAAGGGCGAGGTAGACAAGAAGGTGAGAGTGTATCAAGAAAATATGAATCTGAACCAAGTCCAAGAGATGATTGGGATCGAGCAAGAAGAGGGGATCATGATAGAAAGGAGAGGCCAGGAAGGCACGAGTTAGAACCAAATAAAAAAGAAGACCATGATAGGAAAACAAGGGAGAACAGGTCAGGATTGCGTGAATCAGATAAAAGGCCAAGAGAGGACCCTGATAGAAAAGAGAAGTCAGGAAGGCCGGAGTTAGAATCATATAGAGGAGAAGATGGTGATAGGAAAGGAAAAGAGGATCATGATAGAAAGGAGAAATCAAGAAGACGCGAGTCAGAATCATATAGAAGAGAAGATGGTGATAGGAAAGAAAGAGAGGATCTTGATAGGATGGAGAAGTCAGGAAGACATGTGTCAGAATTGAATCGGAGAGAAGATGCTGATAGGTCAAGACGGCATGAGTCAGAACGAAATAGAAAAGATGATTATGATCGGAGAGATAATGTTAAACGATCACGGAGTAATGAAGATTCATCTTCCCGTCATCACAGG------------------------------------

---------------------------------------------------------------------------------------------------------------------------------------------------------------------------------------------------------------GAAAAGTATGAGAAAGAAGCTAATCACAGAAGGCGAGATAGACAAGAAGGTGAGAGTGTATCAAGAAAATATGAATCAGAACCAAATCCACTAGATGACTGGGATCGAGCAAGAAGTGGGAATCATGATAGAAAGGAGAGACCAGGAAGGCACGAGTTAGAATCAAATAAAAAAGAAGACCATGATAGGAAAGCAAGGTTGAACAGGTCAGGATTGCGTGAATCAGATAAAAAGCCAAGAGAGGACCCTGATAGAAAGGAGAAGTCAGGAAGGCTTGAGTTAGAATCTTATAGAGGAGATGATGGTGATTGGAAAGCAAAAGAGGATCATGACAGACAGGAGAAATCTAGAAGACATGAGTCCGAATCATATAGAAGAGAAGATGGTGATAGGAAAGAAAAAGAGGATCTCAATAGGAAGGAGAAGTCAGGAAGACGTGTGTCAGAATCAAATCCGAGCGAAGATGCTGATAGGTTAAGACGGCATGAGACAGAACGAAATAGAAGAGATGATTATGATCGGAGAGATAATGTTAAACGATCACGGTGTAATGAAGATTCATCTTCCCGGCATCACAGGGAGGAAGATGAGGTGCAGGCAAGGCGATCACGGAGG

7184 Unigene24248_R-tanguticus CL2525.Contig1_R-bungei

TTGGACTTGAGTCATTGTCGTGAATTAACTCAAGTACCAGATGGGGTTGGAGGTTTGATCAATCTTAGATATGTGGACTTGAGTCGCTGCTTTGAGTTGGTACTCTTACCACATCGAATTGGAGAGTTGACCAACCTTCATCAACTGAATTTGAGTCGGTGTTTTAAATTAATTCAAATGCCAATAGGGATTGGAAAGTTGAGTTGCCTTGAGACATTAGAGACGTTCGTTGTAGGCGTAGAAGACAGTGGAAGAAATATCACAGAACTGCAAGGACTAAACCGTCTACGAGGAGATTTGGAGATAAGTGGACTTGAGCACGTGAGAAATGGAGCAGAGGCGAAACAAGCCAATTTAGCAGCAAAGCAAAACCTTTGTTCTTTGGAGCTGAGC---TGGGACAACATCAGCAGCAGCAAGAACAACAACAAGAGCGAAGATGTGATCCAATGGCTCCAACCT---CATGAACGTTTAAAGAAGTTGCGTATTACCGGA------------------------------

---------------------------------TTACCAGATGGGATTGGAGGTTTGATTAATCTTAGGTACGTGGACCTGAGCTCATGCTCTAAGTTGATACACCTGCCACAGCGAATTGGAGAATTGAGCAACCTTAGACACCTGAATTTGAGTGGGTGTTATGAATTAATTCAAATGCCGATAGGGATTGGCAAGTTGAGTTGGCTGGAGACACTAGAGAGGTTCGTTGTAGGCCCAGAAGACAGTGGAGCGGATATCACAGAATTGCAAGGACTAAACCATCTACGAGGATATCTGGAGATACAGGGACTTGAACATGTGAGAAATGGAGCAGAGGCCAAACAAGCCAATTTAACGGCAAAGCAAAAACTTTCTTCTTTGAAGCTGCAGTGGTGGCGGGACATCTGCAGCAAC---------------AGTGAGGATGTGATCCAAGGGTTTCAACCTAGTCATGAAAGTTTAAAGGAGTTATCTATTTCTGGATATGGAGGCTTAGTATTTCCGAGTTGGATA

5904 Unigene21404_R-tanguticus CL3850.Contig4_R-bungei

------------------------------------------------------------------------------------------------------------------------------------------------------------------------------------------------------------------------------------------------------------------------------------------------------------------------------------------------------------------------------------------------------------------------------------------------------------------------------------------------------------------------------------------------------------------------------------------------------------------------------------------------------ATGATGGGATTGGCAGTTGGTATGGTTTTCGTGGTGGCTTTTGGGCCTTTTTTGTATCATGGACAGATACAACAAGTGATTGCTCGCATGTTTCCTTTCGGTAGGGGATTATGCCATGCCTACTGGGCTCCAAATTTTTGGGTATTCTACATAATTCTAGATAAACTACTGTCTTTCATTCTCAGAAAACTTGGTTTCACCATCAAGGTACCACTAGCTTCCTTCTCTGGAGGTCTAGTCGGAGACGCCTCAGCTTTCTCCGTACTTCCTCAGGTGACCCCATTGGTAACCTTCGTACTAACCCTGCTTGCCATCTCTCCATGTCTCATAAAAGCATGGAATAATCCTCAAAGAGGTCTTATCACAAGATGGGTAGCCTATGCTTATACATGTGGGTTTGTTTTCGGATGGCATGTCCATGAAAAGGCATCCCTTCATTTTGTCATTCCTCTAGCAATAAATGCAGTACAAAGTTTCGAAGATGCAAGGCACTACTTCTTACTATCAATAGTGTCATGTTACTCGATGTTCCCCCTTCTATTTGAAGCGAAAGAGTACCCTATAAAAGTGCTTTTACTTATATTGCACTCTCTTCTCATGTGGGCGGGTTTTGCCTCGCTGTTCGGTAAAAATACACCGCCAAAAACGTTGACAAGTAAAAAGATGAAGGTCAAGATTTCAAGGGGAAAATCTTCAGATGCTGGCGTGGAAGGTGGAATGGTGATAGGATGGGTCGAGTTGGGTTATTTGGTAGGTCTTGTGGGGGTTGAGTTATGGGGACTGATTTTGCACCGTTATCTTTTAGGCAATAGGCTTCCTTTTTTGCCTCTTATGTTGGTGTCTGTGTACTGTTCTCTGGGTATTATGTACTCGTGGTTGTGG---------------------

GAACTCTGTTGGTTTGCAGGTATAGCCACCTGCATCAAAATTCTCCTCATTCCTTCGTACCGAAGTACAGACTTTGAAGTTCATCGCCATTGGCTGGCTCTCACTTCCTCTCTTCCTCTCTCTCACTGGTATTCAGATCAAACAAGTCAATGGACTCTCGATTACCCACCACTGTTTGCTTACTTTGAACGATTTCTTTCCATATTTGCCCATCTCGTTGACCCCAAGATTGTGGATCTTCATAAGGGTTTGGATTACAATGCTACATCAGTTATATATTTCCAGAGACTCAGTGTTATTGTGTCTGATTTGTGTTTGTATTATGGAGTTTATCGATTGAGCAAAAATCTAGGGTTTTGGAGAAAGAGAATGCTTTTGGTGTTGATCATCTGGTCTCCGGCCTTACTTATTGTAGATCACATGCATTTTCAGTATAATGGATTTCTTATTGGAGTTTTGTTGGTATCACTTTCATTATTGGAGGAAGGGAACGATTTGATGGGCGGATTTTTTTTCGCAATCTTGCTATGTTTAAAGCATTTGTTTGCAGTGGCCGCACCAGTGTATTTTGTGTATTTGTTGAGACATTATTGCAGAGGGGGAGTTCTTAAGGGATTTGGCAGGTTTTTGATGATGGGATTGGCAGTTGGTACTGTTTTCGTGGCGGCTTTTGGGCCTTTTTTGTATCATGGACAGATACGACAAGTGATTGCTCGCATGTTTCCTTTCGGTAGGGGATTATGCCATGCCTACTGGGCTCCAAATATTTGGGTATTCTACATAATTTTAGATAAAATACTGTCTTTCGTTCTCAGAAAACTTGGTTTCACCATCAAGGTACCACTAGCTTCCTTCTCTGGCGGTCTAGTCGGAGACTCCTCAGCTTTCTCCATACTTCCTCAGGTGACCCCATTAGTAACCTTTGTTCTAACCCTGCTTGCCATCTCTCCATGTCTTGTAAAAGCATGGAATAATCCTCAGAGAGCTCTTATCACAAGATGGGTAGCCTATGGTTATACATGTGGGTTTGTTTTCGGATGGCATGTCCATGAAAAGGCGTCCCTTCATTTTGTCATTCCTCTAGCGATAATTGCAGTACAAAGTTTAGAAGATGCAAGGCACTACTTTTTACTATCGATAGTGTCCTGTTACTCGATGTTCCCACTTCTATTTGAAGCGAAAGAGTACCCTATAAAAGTTCTTTTACTTATTTTGCACTCTCTTCTCATGTTGGCGGGTTTTGCCTCGCTGTTTGGTAAAAAGGCACCGTCAAAAACGTTGACAAGTAAAAAGGTGAAGGCCAAGATTTCAAGAGGAAACTCTTTTGATGCTGACGTGGAAGGTGGAATGGTGATAGGATGGGTCGAGTTGGGTTATTTGGTAGGTCTTGTGGGGGTTGAGTCATGGGGACTGATTTTACACCGTTATCTTTTAGGCGATAGGCTTCCTTTTTTGCCTCTTGTGTTGGTGTCTGTGTACTGTTCTGTGGGTATTATGTACTCGTGGTTGTGGCAATTGAAGTGTATCTTAAAA

5440 Unigene20129_R-tanguticus Unigene17410_R-bungei

------------------------------------------------------------------------------------------------------------------------------------------------------------------------------------------------------------------------------AATCCCGTGACACAAAATAACCCCATTATTGAGGAGGGATTTTTCGAAAGAATGCGAAGATTTGCTTTTGGTCCAAATGATGCTTCTTCTGACAAGACTGATAATAACAATCCTGCAATCCGTTCTACTACTACTGATAGCTCGGAGAAAGTGGACGTCGATGGAAAATCCGCTTCCATCTCAGCCGTGTCATCAAATTCAAATGAATCAAACTCGGAAGAAAAGGCTACAATAAATAGTGAAAAGGAAGGTATGTTCAGGAAACTTGTGAGAATTTGGAAAGGTAGCACACAGCCTTATAATACACTGGAGCAGCTCAGTGAAGATGTGAACCAAGTGAACAGTTCTATTGCCAGCAGTGAGATATTTTCTAAAGCTGCTTTTTGGGAGGATATGCTATCATTTCTTCAAAGCTATAAGGGTGCAGCCTTGATCTCGCAATCGAAGACCAGGGAACAAATAGCTCAGCAGTTGCAGAAGGGAGGTCCAAAAGTTCTTCACACTCTCACAGTAGGAGATCTCAATCGCCTTGTCGATTTACTAATTTCGGAAAAGAAATGGGTTGTAGAAGAAAACACCTCCAATACATTTCCTTTCAAACTACTCCCCGTATCTAAAAATCCGGTCTCTGATTCTTCAAACCCTCTGAGCTCAATATTTTCGGGGAAACCCTCTCAGTCCCACTTGCAA---------------------------------------------------------------------------------------------------------------------------------------------------------------------------------------------------------------------------------------------------------------------------------------------------------------------------------------------------------------------------------------------------------------------------------------------------------------------------------------------------------------------------------------------------------

TCAAAACTTGCTAAGACAGTTGATTCCATAGTGAGAAGGTCGGCAGAGTTGGAGACTAATGACGAAGATAGGAGGAGAACTGTATCTGCAAAATTAACAGGCAGTAAAAGTCCCCATACCCTAGATGCCGCCGAAAAATCATTATTGGCAGCAAAAGTGTCCCCCAAAATTGATGCTCCAATTTCCGTTAAAGAAAATATAGATTCAGCGGAACGTCAATCCAATCCCATGACACAAAATAACCCCACTATTGAGGAGGGATTTTTCGAAAGAATGCGAAGATTTGCTTTTGGTCCAAATGATGCTTCTTCTGACAAGACTGATAATAACAATCCTGCAATCCGTTCTACTACTGCTGATAGCTCCGAGAAAGTGGACGCTGATGGAAAATCTGCTCCCAACTCAGCCGTGTCTTCAAATTTAAATGAATCAAATTGGGAAGAAAAAGCTACAATAGATAGTGAAGAGGAAGGTATGTTCAGGAAACTTGCGAGATTTTGGAAGGGTAACACACAGTCCTATAATACAATGGAGCAACTCAGAGAAGATGTGAACCAAGTGAACAGTTCTAGTGCCAGCAGTGAGATATTTTCTAAAGCTGCTTTTTGGGAGGATATGCTATCATTTCTAGAAAGCTATAAGGGTGCAGCCCTGATCTCCCAATCGAAGACCAGGGAACAAATGGCTCAGCAGTTGCAGAGGGGAGGTCCAAAAGTTCTTCACACTCTCACAGTAGGAGATCTCAATCGCCTTGTCGATATACTAATTTCCGAAAAGAAATGGGTAATAGAAGAAAACACCTCCAATACATTTCCTTTCAAACTAGTCCCTGTATCTAAAAATCCGGTCTCTGACACTTCAAACCCTCTGAGCTCAATATTTTCGGAGAAGTCCTCTCAGTCCAACTTGCAGGTAGCACCGCCAAAAACGAAAAGTGAGGTACTCAGGGATTGTCAAAGGTTGGTAGCTGATATCTTGAAGGAAAGCCCACAAGGATTCGATTTACGTAGCTTTAGGAGGTGGTTTCGTGAAAGGTATGGATACTGCATAGATAGTAAGAAATTTGGGTACAAGAAACTGGGGCACATGCTAGAAACAATTCCGGGTATAGCTATTGACTGTGATCGTATGGTACCGTCTTCGGAAAAGATTATTACACCCTACAGTGTTGAGAATAACCCCACGGCAAAGGTAAATAATTTGATTTCGGAGATATTATCTGACGATGCAGGGAAGAAGCGAACTACTGCTCCAGAGTTACTTTCTTGTGGTGCAGGGAAGTCAACTGATGTAGAAGAGTTAGACAGATATTCGGATTTGGATTCTGAGAAAGATGGGTCAGATGGGAAAAGTTCAATAAATGATAGTGGTGATGGAAGCAGCAGCTCTTTGTTACTTGTGCTAGATTCGTTTTATAATAAGAATGATGATAGGCAGCAAGGACAGGGT

4912 Unigene17995_R-tanguticus Unigene31731_R-bungei

---------------------------------------------------------------------------------------------------------------------------------------------------------------------------------------------------------------------------------------------------------------------------------------------------------------------------CAATTACTTAACCTGATGGCAGAACAACTATCCGATGTTGTAATCCAATCCAATACATGGAAGGTGCTAGACACTAGCTTGGTGCCTTTTTGTCTACGCTCCATTGGTACTTCTATAGGAATGCGTCAGAATGAAGAAACATCTGTTTACCAATGGAGTGATCAGTCAGTCATGCGCGGGCAAACTAGTCTTCTAAATGATTCCATTGCAGATAAACAACCCATCCCACCTCTACTCGAGTCTTTACCACTACCCATATCCTGCCATGTTTTAACTTCTTTACTTCTTACTTCATTAACAAGTTCTGAAGCTCAACAAAATCCACCAGAAAGAATGTTGCTCAAGGGGTCTTCTGCAGATGTTTTTTCTAGAAATTTACTGTGGGATATCTGTGCCATGGCCATACAGATGCTTTCACAAAGTCCAGAACACAGGTCATGTGCAACTCACCTTCTCCTTCCACATATTTTGAGGATATTTGCACGCCAATCATCGTTTCAAGTCTCTGTGCACGGGAAAATACATGTACTCTCTAGGGGTACATTCTTCCAGAAAATTTGGAAGCATTGTGGAGCGCTGTTTTCTCATGGACCTGGAGAACGGAGAGATGCA

GTTTCATCGCTGGTAAAAAGCTTGTCATCATCGCTGGTAGTAGTATCACCAGCAGCGATTCCATCAATTTTGGAATGTATTTTGACTTCAATCCAATCTCTCTCTTCTACTTCCCTCTTTCATTCACTGCTCCAAGCTTTCTCCAATCTCCTCTCTTCAATGGATTGCGGGCAATCAAATACTAGCTCCATATCATCTTTCACAACTGCACTTTGTTACCTCTTAAAGAAATCAGGAACTGATCACAATGCTTTGCAAGCATTTATTTGGGAAGGTTATCTTCCAACAATGAAGATTATAAATGTGAATCACCACGAACTACTTAACCTGATGGCAGAACAACTATCCGATGTTGTAACCGAATCTAATACATGGAAAGTGCTAGAAGCTAGCTTGGTGCCTTTTTGTCTCCGGTCCATTGGTATATCTATAGGAATGCGTCAGAATGAAGAATCATCTCGTTATCAATGGAGTGAACAGTCAGTCATGCACGCGCAAAGTAGTCTTCTAAATGATTCCGTTGTAGATAAACAACCCATCCCA------CTCGAGTCTTTACCACTGGCCATATCCTGCCATATTTTAACTTCTTTCCTCCTTACTTCACTAAGAAGCTCTCAAGCTCAATATAATCCACCAGAAAGAATGTTGCTAAAAGGGTCATCTGCAGATGTTTTTTCTAGAAATTTACTGTGGGATATCTGTATCATGGCCATACAGATGCTTTCACAAAGTCCAGATCACAGGTCATGTGCAACTCACCTTCTCCTTCCACATATTTTGAGGATATTT------------------------------------------------------------------------------------------------------------------------------------

2703 CL7090.Contig1_R-tanguticus Unigene34793_R-bungei

---------------------------------------------------------------------------------------------------------------------------------------------------------------------------------------------------------------------------------------------AGGCAGTGGCATGAAGGCACACCTGAAGAGAAAAAGTTGCCTCTTGAAGGCACAAATCCTCCCCCACTCCGTTCAATAAGTCACACTTCCTTTAAGAGATTGAAAAATTCGCATGTTCAGCTGTCATCCTTTTCAAATAACCTACCTGATAATCAACCACTGCCTGCAACAAATCATTCTTCATTTGCGACACATTATACTAGTCACAGTGGTGGCGTAGCACGTCATAATGAAGTAATCTCTCATAACCAACAAGCATCTATGGTGAGCAAAGCAACGGTTTTCAAATCAAAGAAAGGTCATCAACTTTCAGATGCTCACCATATCAAGAAAAGATTTGCTACGGTTGAGATTCGCGCAACGAATCAGCTTATTCACGAGGTTGAGAGGATCATCTATGGTGGAACTTACCCCAATCAAGATCAAATGGAGAATGCGAAATTGTTACTGAAAGAGCAAGAAAGAGCTATTATAGACGCCCTAGAAAAACTTGCTGAATTTTCAGATGATGAGGAC---------------------------------------------------------------------------------------------

AAGATTCCTTCAATGACGCTATCTCATGGAAGGAATTTAACTGATATGGATGTTGAACTACATCAAATTGAGTCAGAGGCATACGGTGCTGTTTTGAGGGCACTCACTGCTAGAGGTGGCCCTACTTGGGATCACCTTGATACTTTATTCAGTCTGCGAAAGGAACTTAGAATTTCTGAAGCCGAGCACAAAAAATTCATTGCACAAGTTGCTTCAGATGACACTGTCAATATGATAAGGAAGTGGCGTGAAGGCACAGCTGGAGAGAAAGAGTTGCCTCTGGAAGGCACAAATCCTCTCCCACTTAATTCAATGAGTCAAACTTCCTCTAAGAAGTTGAAAAGTTCGCATGTTCAGCTCACATCTTTTTCAAACAACGTACCTGATAATCATTCATTGCCAGCAGCAAATCCTTCTTCATTTGTGACACATTATAGTGGTCACAGTGGCGGCATTGCACGTCCCAAGCAATCAATCTCTCATAACCAACAGGCATCTATGGTGAGCAAAGCAGTGGTTGTCAAATCAAAGAAGGGTTATCAACTCTCAGATGTTCACAATATGAAGAAAAGATTTGCTACAGTTGAGATTCGCGCAACAAATGAGCTTATTCACGAGGTTGAGAGGATCATCTATGGTGGAACTTACCCCAATCTAGGTCAAATGGAAAATGCGAAATTGTTACTGAAAGAGCAAGAAAGAGCTATCATGGATGCCCTAGAAAAACTTGCTGAATTTTCAGACGATGAGGATGAGTTGCAGAAGAATGGGCATAAAAAGGGGGCACACTATGCACTTTATGATCAGCTAGATACTACTCAAGGTTTTTACCGGAATTCTATGGAT

2245 CL6065.Contig2_R-tanguticus Unigene16747_R-bungei

------------------------------------------------------------------------------------------------------------------------------------------------------------------------------------------------------------------------------------------------------------------------------------------------------------------------------------------------------------------------------------------TTACCTCTGTCACCGCGCAGGTGCAGGTCTGGGCTCCGT------------CGTGATCGACCTAGCCCTTTAGGACTAAATGGAAAGACGTATATTCATTCTGCAACCACTCATGAGGATAGCAGTGAGAAGGTTACTGCAGAAAATGGTGACTTGAGAGTGTATGATATTCAGAGGTCGGTGCGTCATCATCAAATATTCACGGAACAACCCGAGAATGAGAAGGATCCTTTTCTTCAGCGCCCCAACAAAAAACAACGGACTAAAAAGTCACCAGATGATCCTTTTTTTACACCTAGCAAGGAACCTGTTGAACGACATTTTGTTGATGACTGGGAAATGGTGCAGCAGGGTGGTAATTCTGGTTCTGCCAGAAGCCCCCTCCGTGCGCCTCTTGGGATACCATTTTGCTCAGCCAGTGTAGGTGGGTCCCGTAAGGCTCTGCCTACGACTAATATTAGTAGCTTTGCAATCTCGTCCGACAGTGGCATGTTATTCGATACTGGAACATTGAGGAGACGTATGGAACAGATCGTTGGAGCACAAGGTCTTGAAGGGGTGTCAGTTGACTGTGCTAATCTGTTAAACAATGGGTTGGATGTGTACCTGAAGCGGTTGATACGGTCTTGCATTGAGTTAGTAGGGTCAAGGTCAGGACCTGAACCAGCACTACTCTCTGCAAACAAACATCCACCATACAGAAAACCTATGAATGGATTAAAACCAGAATATCAGTTACAGATGCAAAGTAGTAGTGGGCCCAGTGGCTGTATTCAAGAATTGAGCACGCGCTGTCCAATATCTCTACTAGATTTGAAGGTAGCAATGGAGCTTAATCCACAACAGCTTGGAGAAGATTGGCCGTTACTGCTTGAAAAAATTTGTATGCATGAATGCGAGGAA

CAACAGCAATCACGGATCAATCTGACTGACTTGAAGACTCAGATAGTGAAGAAGCTTGGGCCAGATAGGTCCAAGCGGTATTTTTATTTTTTGAATAGATTACTAAGCCAGAAGATAAGCAAGAGTGAATTTGATAAGTTATGTCTGCGGATTCTTGGACGCGAGAATTTGGCGTTGCATAATCAGTTGGTACGTTCAATTTTGAAGAATGCTTGCCATGCCAAGGTTCCGCCGACTGTATGTCAGAAGGAAGCTACACAAAAATCTACGGTGAATGTTACAAAGAAATCTCCTTTAGCTGAAGATGGGTATCGGGAAAGTGGGTCGTCTACCCTTACTGCAGGACAAAGCTCACATATGTGGTCAAATGGGAATGGTTTACTTCTGTCACCTCGCAGGTGCAGGTCTGGGCTCCGTGATCGTTGGTCTCGTGATCGACCGAGCCCTTTAGGACTAAATGGAAAGACATTTATTCATTCTGCAACCACTCCCGAGGATGGCAGTGCGAAGGTTACTGCAGAAAATGGTGACTTGAGATTGTATGATATTCAGAGGTCGGTGCATCATCATCAAATATTCACGGAACAACCTGAGAATGAGAAGGATCCTCTTCTTCAGCGCCCCAACAAAAAACCACGGACTAAAAAGTCACCAGATGATCGTTTTTTAACACCTAGCAAAGAACCTGCTGAACGACATTTTGCTGATGACTGGGATATGGTGCAGCAGGGTGGTAATTCTGGTTCCGCCAGAAGCCCCCTCCGTGCGCCTCTTGGGATACCATTTTGCTCAGCCAGTGTAGGTGGGTCCCGTAAGGCTCTGTCTACAGCTAATATTAGTAGCTTTGCAATATCGTCCGACAGTGGCATGTTATTCGATACTGGAATATTGAGGAGACGCATGGAACAGATCGTTGGAGCACAAGGTCTTGAAGGGGTATCAGTTGACTGTGCTAATCTGTTAAACAATGGGTTGGACGTGTACCTGAAGCGGTTGATACGATCTTGCATTGAGTTAGTAGGGTCAAGGTCAGGATCTGAACCAGCACTATTTCCTGCAAACAAACATCCACCATACAGAAAACCGATGAATGGATTAAAACCAGAATATCAGTTACAGATGCAAAGTAGTAGTGGGCCCAGTAGCTGTGTTCAAGAA---------------------------------------------------------------------------------------------------------------------------

7507 Unigene24904_R-tanguticus CL3477.Contig2_R-bungei

GAAGAACAAGAGTGGTCCTCTACGCCAAAGTCGAGTATTGGAAAATCAACGTCTGAAATAGTTATTGATATCAGTGACAGTGATAGCGACAAAGAAAGTGAGGCAATTCCAAAGGAAACCCTTCCATACGATGTTGCTACAAGGACATATCCAAGTCCCAAAAATGAGGAAAATAAGAGCAGTTCTTGTGAGAAATTTCCATCAGCTTCAACACTTAAACGGAAACGGTTACGCAAAATAGTTGACAGTGATAGTGATGATGGTTCAGACAATGAGACTAGGGTAGAAAGATTTCCAAAGAAGGTCGTGGCGAAGAAATGTTCAAGTCAGAATGGCTATGAAGTTAACAACACTTGCGAAAGAGAGCGGGCACTCAAAGTCACAAACAGTGACAGCAAGGAAGATGATAATTCCGTAAGTAAGGATCCACCTAAGAATGAGATTTCCAAGATCTTATCTGGAATTAGGAAACGGGAAGCTTGTTGGAAGTATGAAGTTGACATGGTTAAATCATTTGAAAGAGATCCCCTACTCTGTATGAAAGCTGTTTGTACACTATATAGACGTCGCTTGTTTGAGAAAAAAGCCGATGGAGACTCTTCAAAGCTGAACAACCGCAGGTTTAGCCTATCCCGTGAAGAGAGGGGAACTAGCATAGCCAAGTTTCTTCTGGATGGAAATGAGGAGTGTGAGATGAAGAAGTCTGTTAAGGAGTTGGAGATGTATGATCCAAAAGGATTACAAGAATGTCAAAAATTGGCATCTCTCTATTCCAATCAGTTATTCACAATTTATCAGAACAAGGAAGACCCATTCTTC

---------------------------------------------------------------------------------------------------------------------------------------------------------------------------------------------------------------------------CGGAAACGATTACTCAGAATAGTTGACACTGATAGTGATGATGGTTCAGACAACGAGACTAGGGTAGAAAAATTCCCAAAGAAGGTCGTGACGAAGACATGTTCAAGTCAGAAAGGCGATGAAATTAACAGCAGTTGCGAAAGCAGGCGGGCACTCAAAGTCACAAACAGTGACAGCAAGGAAGATGATAATCTTGTAACTAAGGATCCACCTAAGGATGAGATTTCCAAGATCCTATCGGGAATCAGGAAATGGGAAGCTTGTTGGAAGTATGAAGTTGACATGGTTAAATCATTTGAAATAGATCCCCTACTCTGTATGAAAGCTGTTTGTACACTTTATAGACGTCGCTTGTTTGAAAAGAAAGCCGATGGTGGCTCTTCAAAGCTGAACAACCGCAGGTTTAGCCTATCCTGTGAAGAGAGGGGAACTAGCATAGCCAAGTTTCTTCTCGATGGAAATGAGGAGTGTGAGATGAAGAGGTCTGTTAAGGAGTTGGAGGTATACGATCCCAAAGGATTACAAGAATGTCAAAAATTGGCATATCACTATTCCAATCAGTTATTCACAATCTATCAGAACAAGGAAGACCCATTCTTC

6147 Unigene22026_R-tanguticus Unigene26913_R-bungei

ATGGAAAATAAACACCCCACTACCACCCCCACGAACCCTAGAGGCGTCTGTGAAAAGCTCTTTGATGCGATTACAGTTACCCCTGGTTTTCGTTCACTTCGTCGCATTTCTTACCACCCACAAGACTCCCCACCGTCGTCTGTAGACTCTCATACTAAAACCAATGGTCTCCACCATCCAGTAACGCCCGAACCAGTCAAGCCCACGCCAGAGAAGAAAGTAGCGGAAGTAATTCAGGCTAATATGCCACCACCTCTGCCTTTATCATCAAAGCCACATGCCACAGTGAGTCCTAAGATGGATACAACTCCAATTCCAAATCCAGACCAGGCTCAAATGAAGGTAAAA---------------------------------------------------------------------------------------------------------------------------------------------------------------------------------------------------------------------------------------------------------

------------------------------------CCTAGAGGCGTCTGTGAAAAGCTCTTTAATGCGATTACAGTCACCCCTGGTTTTCGTTCACTACGTCGCATTTCTTTCCACCCACAAGACCCCCCACCGCCATCTGTAGACTCTCATACTAAAACCAATGGTGTGCACCACCCAGTATTACCCGAACCAGTCAAGCCCACGCCGGAGAAGAAAGTAGTGGAAGTAATTCAGGTTAACACGCCGCCGGCTCTACCTTTATCATCAAAGCCACATACCACAGTCAGTCCAGAGATGGATATGACCCCAATTCCAAATCCCAGCGAGGTCCAAATGAAGGCAAAACATAAGGTGGATTCGGCTAGTCCGGTAAAGGTCCACAAAGGAAAAAGTGGTGGAAAATCTCGCCTGCATGGAGAAAAGAAGGATGAAGAGAAGAAACCTGTGAAGGTTGAGACACCACCACCTGAAGTGGATGGTGAGAAGAAAGGTAAACTTGCGAATATAAATGAGAAGGCGAATGATTATATTCATCGTGCCAAGATGAAGATCAGAACGACTACTAGGGTTGATGGTGCTGGTGGTGGTGAAGCC

7798 Unigene31132_R-bungei Unigene25417_R-tanguticus

---------------------------------------------------------------------------------------------------------------------------------------------------------------------------------------------------------------------------------------------------------------------------------------------GCTTGTATGGAGCTTTTACATGCTTTGAGCTTAGCAAAATCTTCTGTTCCTGCTATGTTGAAGAATTTGAAGGAAATACAACCTTGGGGTTGTAAGAAGATATTTGAAGAAGATCAAGAACAAGAAAGGGTTTGTTCAGAGAAAGAATTGATGGTGTATAAAGCTTTATTGGTTAGAAACCATGTAAAAAACTGGGTATGCAGAATTGTTGATTCAGGCATCCGTGGCAGTGGTGGGGGTATACTAGAGAAATTTGGAGGAGTTTGCAATTCATCTTTGATGAAGTTTCAATTCAGTGTTTGTGAAGGAATGAAGAAAAAGGGATTTGTCAAAGAGGTTGGTGACATAAACAAAGGAGTAATGCAACTTTTGGCTGTT---CCAGAGGGAGAATACAAGGAAGAAATATTGGAATTGACAGAAAGGGTGGAACTGTTGGGGAAGCTGTTGCAAATGGTGAAGGAAGATGTCAATAAATTGTTCTCAGATTCTATGGTGAAGAGA---------------------------------

ACAACAGCTTTCAACACTTTTGAAACTGAGGTACTGAACAATTTATATCAGTTATCTCTGCATTCAAAACCAGGCTCAGTAGAAGAAATCTACACATTTTCTTGGATACAAACCTCTCTGCATATACTACCCAATATAGACAGATCATTTGCAAAGTTCATTGCAGAGATTGATTATCCAGTTACAAAATGGAAAACCTTTCACGCTGAAGAGTATCTCAAGGGAAATTTAAGTCTCTTGGATCATTTTAACTGTATTTCTTCATCTCTTAATCATATGAGCTTAACTTGCATGGAGCTTTTGCATGCTTTGAGCTTAGCAAAATCTTCTGTTCCTGCTATGTTAAAGAATTTGAAGGAAATACAAGCTTGGGATACTAAGAAAACATTTGAAGAAGATCAAGAACAAGAAAAGGTTTGTTCAGAGAAAGAAATGATGGTGTATAAAGCTTTGTTGGTTAGAAACCATGTTAAAAACTGGGTATGCAGAATTGTTGATTCAAGCATCCGTGGCGATGGTGGGGGTATAGTGCATGAATTTGGTGGAGTTTGCAATTCATCTTTGATTAAGTTTAAATTCAGTGTTTGTGAAGGAATGAAGAAAAAGGGTTTTGTCAAAGAGGTTGGGGATATTAACAGAGGAGTAATGAGACTTTTTGTTGCTGGAACAGAGGGAGATTACAGAGAAGAAATAATGGAAGTGACAGAAAGGGTGGAAGTGTTGGGGAAGATGTTGCAAATGGTGAAGGAAGATGTCAATAAATTGTTCTCCGATTCTTTGGTGAAGAGAGATGAGTTAATACATAGTTTTGAGCAATGTAAA

8034 Unigene25854_R-tanguticus CL7033.Contig2_R-bungei

AGTGATGGCCTTTGTTCGTTTTGTGAGGATTATGCTACCCAGGCAGTAGAATATCTCTCTAACAATGAAACTCAGATAAAAATCCTTGACAGTCTTCATCTCAGTTGTTCTTACACGCACGGTTTCAAGAAGGAGTGCCTGAAAATGGTGGACTATTATGTTCCTCTCTTCTTCTTAGAGATTGAATCAGTAGAGCCTGAGAAATTGTGTCACGAGATGAATCTCTGTGACATGGTATTTACTTCTAAAGATAAATGCACTGTTTGCCATGAAACTGTAGGTGAGATTCTCGAAAAACTGGAGGACCCCGATACACAGTTGGAGATAATTGAAATCCTGTTAAAGGCCTGTGCAAAGACCGAGCAGTATGCTAAAAAGTGTAAGAGATTGGTATTCGAGTATGGACCTTTGATCATGGCTAATGCAGCAGAGTTTTTGGTGAAAAACGACTTGTGCACAGCGATTGGTGCCTGCAATGCTACTAGCACTACTACA

AGTGATGGCCTTTGTTCATTCTGTGAGGATTATGCTACCCAGGCAGTAGAATATCTCTCTAACAATGAAACTCAGACAAAAATTCTTGACAGTCTTCGTCTCAGTTGTTCTTACACACACCATTTACAGAAGAAGTGCCTGAAATTGGTGGACTATTATGTTCCTCTCTTCTTCGTAGAGATTGAATCAGTAGAGCCTGAAAAATTGTGTCATGAGATGAATCTCTGTGACATGGTATTTACTTCTAAAGATAAATGCACTGTTTGCCAGGAAGCTGTAGCTGAGGTTCTCACAAAATTGGAGGATCCCGATACACAGCTGGAGATAATCGAAATCCTGTTAAAGGCCTGTGCAAAGACCGAGCAGTATGCTAAAAAGTGTAAGAGATTGGTATTCGAGTATGGACCTTTGATCATGGCTAATGCGGAGAAGTTTTTGGTGAAAAACGACGTGTGCACAGCGATAGGTGCCTGCAGTGCTACTACCACTGCTACG

4856 Unigene17689_R-tanguticus Unigene20141_R-bungei

---------------------------------------------CTTGTTACTTCTCCAAATCCATTGGCGAATTCCAATCGTTCTCGCAGATCTCCCCCTTCGTTCGATTTCGAGTTGGATCTCAAAGGCTTCAACAATAACGCTTCATTTCCCTCATCTTCTAAGAAGAAGAATACAGTCATCGCAACCACCACTTCTGTTCTCATCAAATCAAACACCACCAAGATGTCTCCATCTCAGAAAACCGTAGTAACGATCTCAGATTTGAAGGATCTCGTCTCATCTCGCTCTGATTCTCTCAAGCGTAATATTGAAATCTCTCACTCCGACATCCTCAAAGAACTTGAAGCTTCTCATTCTAGACTTTCCAAACGATTCAAGATGCACACGCAGGCATGCCAGCAAGTGATGGAGGAAACAAATAGCGAGAATGACAAGTTATCAGATTGGATTATGGAGAATGTTGCATCAGTATCGTCTTCATATGCTGAAATCATCAAAACAGCAGAAGCCTCTGCAAATCAGGTTTGCAAAACATCAATTCCTGCTCTGGCACAGTCAACGGAGAAAGCTATTGAGAGCCTACGGAAACTTCAC---------------

AAGAGGAGATCGAAGCCAACAGCAGCAGCAGCAGCAGCAGAAAGCCTTATTACTTCTCCAATTCCATTGGCAAATTCCAATCGTCCTCGCAGATCTCCCCGTTCGTTCGATTTCGAGTTGGATCTCAAAGGCTTCAACAATAACGCTTCCTTCCCCTCATCTTCAAAGAAGAAGAATACAGTCATCGCAACCACTACTTCTGTTCTCATCAAATCAAACACTACAAAGATGTCACCATCTCAGAAAACCGTAGCAACGATTTCAGATTTGAAGGATCTCGTCTCGTCTCGCTCTGATTCTCTCAAGCGTAATATTGAGATCGCTCACTCCGACATCCTCAAAGAGCTTGAAGCTTCTCATTCTAGACTTTCCAAACGATACAAGATGCACACGCAGGCATGCCAGCAAGTGATGGAGGAAGGAGATAACGAGATTAACAAGTTATCAGATTGGATTATCGAGAATGCTGATCCAATATCAAGATCATATGCAGAAATCAGCAGAACAGCAGAAGCCTCTGCAAATCAGGTTTGCAAAACATCAATTCCTGCTCTGGCACAGTCAACGGAGAAAGCTATTGAGAGCCTACGGAAACTTCACAGGATTCCGATAAAT

528 CL2182.Contig1_R-tanguticus CL61.Contig1_R-bungei

------------------------------------------------------------------------------------------------------------------------------------------------------------------------------------------------------------------------------------------------------------------------------------------------------------------------------------------------------------------------------------------------------------------------------------------------------------------------------------------------------------------------------------------------------------------------XXTCAAGATTTTGATGGGGCACTGCAATCACTAGTATCTCAATGTCATAATCTCCGATTTTTGCACTTTAGGAATCACATTGGACATGAACGCCTTCAACTTTCTAAGCTATCTCTTTCGTTACCTCGGGTGCAGAGTCTTCACATTGGCACTGGTTCAGCATATCCTTTAATAGAGATTTTTGCTCCAAATTTGCGATGCTTGAGAGTTCATGGTCCATTCTTTTGTAATGTACGTCTATGGGATTCTCCCCACTTAGAAGAAGCCTACTTGCTCTTTCTAAACTTGTCGTTAACCAAAGTGAGGCAATCCCCCCCTGATCTATCTCCTTACGAACGTCCCCACTTTTGGGGTTCCACTTTGCAAATCATCGCAAACATTAAATCTTTGCACCTCATGCATGCTGTATTCAAGGTAATGTACACAAAGGACTATATGAGTGGAACAAGAAGATTTGCTCTCAACAATCTAAAAGAACTCATTCTGTATAACATGCGATTGGATGCTACCGTATATCCCGTTGTGAATTTCTTGAGAGGATGCCCTTATTTGGAGATGTTATGTGTTACCTATGGTGTTGCCCGATCAGAACCAAATCTCCTTGTCAAAATAAAGAAGTTAGCTATTGAGCCCAATGATGAAGATCGAAAGCTTTATTGCGATTTCCATGGATATATCTTTCAACACCTCAAAAGGGTAAAGCTGAGAGGAATTCAAGGTGATGCAAAGCAAATGATACTTGTCAAGTTT------------------------------------------

ATAATTGAGGATATGAGGACTCGAGATCGAATAAGCGCTTTACCTGATGAAGTCATTCATCACATATTATCGTTGATGAAGGTGGAAGAAGCGGTTCCAACTAGTGTTTTATCGAAAAGATGGAGATATCGATGGACTTTCATGTCTAAACTCTGCTTCAATTATTACAGTGATGGTTCCAAAGTTTTTGAAAAACTTGGAGACAAATTTCATAGTTACACACCTCGTGGAAATTGTAATATCGCAGCAATTGATGGATGCATAAAACAGTATCAGGCAAAACAACTGCAACAAGTTTCTATTAAACTTTGTGAAAACAAGGATGATGCATCGGAATGCATACAAGAATGGCTCAGATTTGCCACTGAACGGAAAGTTGAAGATGTTCGTGTTGACGCTATCATCGGGGTGAACCCCTCGACCTGGTACAACGTCCCTAACCTTTTGTTTTCATCTCAATGCTTACAATCGTTGTCGTTGGGTCACTGTAACTTATATCCAACTTCTGATATCAAGTGCTTTGATTCTCTGGTATCTCTATCTCTGACTGACTTTCAAGATTTTGATGGGGCACTGCAATCACTAGTATCTCAGTGTCATAATCTCCGGTTCTTGTACTTTGCGAATCACATTGGATATGATCGCCTTCAACTTTCTAAGCTATCTCTTTCCTTACCTCGGGTGGAAAGTCTTTGCATTGGCTCTGGTTCAGCATATCCTTTAATAGAGATTTTTGCTCCAAATTTGCGATGCTTGAGAGTTCATGGTCCATTCTTTTGTAATCTACGTCTATGGGATTCTCCCCACTTAGAAAAGGTTTACTTGCTCTTTCAAAACTTGTCCTTAACTAAAGTGAGGCAATCTCCCCCCGATCTATCTCCTTACGAAGGTCCCCATTTTTGGGGTTCCGCTTTGCAAATCATCACAAACATTAAATTTTTGCACCTCATGCATGCTGCATTCAAGGTCATGTACACAAAGGACTACATGAGTGGAACAAGAAGATTTACTCTCAACAATCTAAAAGAACTTGTTCTGTATGGCACGCGATTGGATGCTACCGTATATCCCGTTGTGAATTTCTTGAGAGGATGCCCTTATTTGGAGAAGTTATGGATTAGCTATGGTGTTACCCGATCAGAGCCAAATCTCCTTGTCAAAATAAAGAAGTTGGTAATTGAACCCAATGATGACGATCGCAAGCTTTGTTGTGATTTTCATGAATTTTTCTTTCAACACCTCAAAAGGGTAAAGCTGAGCGGAATTCAAGGTGATGCAAAGCAAATGATACTTGTCAAGTTTCTCTTGGGAAAGGCGGCATCTCTTGATAACTTGAGAGTGGAG

3730 CL1974.Contig1_R-bungei CL981.Contig1_R-tanguticus

GAAGAGATAGCATATGATTATTGTCCACATAGCGATAAAGGTCCTCATAACTGGGGAAATCTTTGTCCTGAATGGGTGATTATTAACGACGGGTTAGAGCAATCTCCAATTGATATATCAAACGATGATGTAAAGGTAGTCGAAAACACAGATAATCTGCAGATAAATTACAAACCAGGACACGCAATACTACGTAATAGGGGAACTGACGTACAGCTTGCATGGGAGGGAGATGGTGGATCCGTTAATATCAACGGTGAAGATTACCCTCTAGTCCAGTGCCACTGGCACTCTCCTTCTGAGCATACCATTGATGGAAAAAAGTTTGCTTTGGAGCAACACTGTGTTCACCAAAGAACAGATCCTACCACAGGCGTCAGCACAATAGCCGTAATTGGAATTCTATTTGACGTTGGACGATCACAACCATTTCTTTCAGAGTTGGAGGAGTCCATAAAGAAAACAACTGATGAGACTACAAAAGAGGTAGACGTAGGTTTGATTGATGCAACAGACATAGACTGTTGCAATGCTGAGTACTACAGATACATGGGCTCCCTCACAACTCCCGCTTGCAGCGAAGGCGTTATTTGGACAATCGATAGCAAGGTGCAGACTGCTTCAATTGTGCAAATTGAATTGTTGAAAAACGCGGTTCGGAAGTATGCAAGGAATAATGCTCGACCCCTACAGAAACTCAATGGTCGTGACGTC

---------------------------------------------------------------------------------------------------------------------------------------------------------------------------------------------------------------------------------------------------------------GGTGAAGATTACGTTCTAGTCCAGTGCCATTGGCACTCTCCTTCTGAGCATACCATTGATGGAAAAAAGTTTGCTTTGGAGCAACACTGTGTTCACCAAAGAACGGATCCTACCACGGGCATCAGCACAATAGCCGTAATTGGAATTCTATTTGACGTTGGAGAATCACAACCATTTCTCACAGAGTTGGAGGAGCCGATAAAGATAATAACTGGCGAGTCTACAAACGAGGTAGACGTAGGCTTGGTGGATGCAACAGACATTAACATTTCCAGTGCTAAGTACTACAGATACATGGGCTCTCTCACTACTCCCTCTTGCAGTGAAGGCGTCATTTGGACAATCGATGGGAAGGTGCAGACTGCTTCAATTGTGCAAATTGAATTGTTGAAAAACGCGGTTCAGGATTTTGCAAGGGATAATGCTCGACCACTACAACCACTCAAC------------

583 CL7696.Contig2_R-bungei CL2301.Contig4_R-tanguticus

------------------------------------------------------------------------------------------------------------------------------------------------------------------------------------------------------------------------------------------------------------------ACTGCTGAAGAAGATTTAATA---CCTGTTCCTGAAGATAGTTCACCAGAACCCCTGAAAAATACTTTGGAAAATGATGTCAGTGTGAAAGTTGCACAGAAACCACAGAGTTTGTCACCTTATTCTGTATATGCTGATCTCAAACCACCATCGTCTCCCATTCCAGTGGCCCCCAGCAGTGCAAAAGTAAAAATAGATGTCGTACAAGCAGTATCAGAACCACCTATTGAAGCAAGTCAGAATAATGTTTCAGTAATAACAGCCACAACCACTGATACACAAACAGACCCGCCCAAAGTGCAGCAAACAAGGCCTCTTTCCCCTTATTTTGCTTACGAAGATTTGAAACCGCCAACTTCTCCAATCCCGTCCTCTCCAAAA

AAGGTGGTGGAAGTAATTGCTGAAACAACTGCACCATTAAAACCATTTGGTGAGCTTCTTTCAAAAATACCGTCTCAACGAAAAGATACACCCTTACCGAAGGATATTTCTCCTGTAGCCACAAGTTCTGTGACTAAGGAGGGATCTGTCAAGACAAAAATGGCAGGGGCTGCTTATATTCTAAATGAAGATTTGAAACCACCACCACCTCCTCCTACTCCAACCTCAAGCTTGTCTGAAACTTCTACAAAGGATGCAACTGCTGAAGAAGCTGTAGTAGTACCTGTTCCTCAAGATAGCTCACCAGAATCCCCGCAAGATACTTTGGAAAATGGTGTCAGTGTGAAAGTTGCACAGAAACCAGGGAGTCTGTCACCTTATTCTGCATACGCTGATCTCAAACCACCATCATCGCCAATTCCAGTGGCCTCCAGCGGTGCAAAAGTAAACGTAGATGCTGTACAAGCAGTATCAGAACCACCTATTGAAGCGAGTCAGAATAATGTTTCAGTAATAACAGCCACAACCGCTGATGCACAAACAGACCCACCCAAAGCGCAGCAAACAAGGCCTCTTTCCCCTTATTTTGCTTATGAGGATTTGAAACCGCCAACATCTCCAATCCCATCCCCTCCAAAA

5449 Unigene20153_R-tanguticus Unigene27108_R-bungei

------GCTTACATAAACATTCCTCACTCTACAGCATTGCGGTTCACAAAAGCATTAAAAATGTCGAAGTCGTCTATTAGTGTTGCATTCCAAGTTTCGGAAACAGTTCCTGCTCGTGTGCTTTCAACGGAACGGAGGCCGAGCTTCATCAAGAGGTTGGAAACCATCAAAGAAGAAGGTGATGTTCATGGCCGCAATGGAAAATCAACTCGTGTTGAAGGCGTAGCAACTGATTTCGGAAGGAGACGTCCATCAGTTGTTGCAGGG

XXGCTTACATACCTCAGTCTA---------CTAGCATTCCAGTTACCAAAAGCATTAAAAATGTCGAAGTCGAGTGTCAGTGTTGCATTTCAAGCTGCGGAAACAGTTCCTGCTCGTGTTATTTCAATGGAACGGAGGCCGAGCTTTATCAAAAGGTTGGAAACCATCAAAGAGGAAGGTGATGTTCATGGCCGCAATGGAAAATCAATTCGTGTTGAAGGCGTAGCAACTGATTTCGGAAGGAGACGTCCATCAGTTGTTGCTGGG

7710 CL931.Contig2_R-bungei Unigene25282_R-tanguticus

ATGACACAAGAAAATCAAGGGACCACTGTAGCTGTCCCGAATCATAATTTGCCTGTGAAGCGGAAAAGAGGCCGTCCTCGCAAGGATAGGAGTCAAGGACCATCTCAAGTACAAGGGCAGGTGCTTAGTACACCTACCCCGACACAAGGGCATGTTCCTGTAACACCTATCCCCACACAAGCGCAGGTGCCTATTGCACCTCCTCCTCCCCAAGGAGTGGTCAATATTACACCTACCACCTCACAGGTCCAGGTGCCTATTCCTCCTACCCCAGTACAAGGGCAGGTGTCTATTCCTCCTTTCACCATAACAGAGGCGAAAAATCATCAGAGCAGGAAACCAGATATAGTGCCAGACACAAAGAACGATATGGTGGGCCAAGCAGTATTTGGAATCCTTGATGGATCATTTGAAGCTGGATACTTATTAACAGTTAAAGTTGGAAATACCAACACTGTTTTGAAAGGTGTTGTCTTCCAGCCTGGGTCATCAGTTCCAGTATCTGTGTCAAATGATGTGGCACCACATATCAAGATGTTGAAAAGGGCAGATGTGCACATGCCCCATAATTCTCCAACAGCTTTCTATCAAGCGCCACCAATAGCCACACCCCAACCGTCTAAAGTACCGCCTACAGTCGCACCCCAGCCGGCTCAAGTGCCGCTTATAGTCACGCACCAATCATCTCAAGTGCCTCCTATAGTCACACATCAACCATTTCAAGTGCCGCCTATAGCCACACCGGAACCCTCTCAAGTAGCGCCTATAGCTCAGCAAAGTGTTCAAGCTTTGACTCAGTCTAACGATTTACTACCTGCACATCCTCAGCCAGAACTAAAGCATGATGGGACTGTAATTCAGCCAACAGTGCTGGAAATGCCCCAGGCTCCGGCCGTATCAGACGCTTCAGGTAATGAAGATGAACTACTCAAAACACAGTCATCTGTT

ATGACACAAGAAAATCAAGGGACCACCGTAGCTGTCCCGAATCATAATTTGCCTGCGAAGCGGAAAAGAGGCCGTCCTCGTAAGGATAGGAGTCAAGGACCACCCCAAGCACAAGGGCAGGTGCTTATTACACCTACCCCGACACAAGGGCAGATTCCTGTGACACCTATCCCCACACAAGTGCAAGTGCCTATTACACCTCCTCCCCCGCAGGGACTGGTGAATAATACACCTACCGCCTCACAGAGCCAGGTGCCTATCCCTCCTACCCCAGCACAAGGGCAGGCGTCTATTCCTCCTTTCACCATAACAGAGACGAAAATTCATCAGACCAGGAAACTGGATATAGTGCCGGACACAAAGAACGATATGGTGGGTCAAGCAGTATTTGGAATCCTTGATGGGTCATTTGAAGCTGGATACTTATTAACAGTTAAAGTTGGAAATACCGACACTGTTTTGAAAGGTGTTGTTTTCCAGCCCGGGTCATCAGTTCCAGTATCTGTGTCAAATGATGTGGCACCACAAGTCAAGATGTTGAAAAGGGCAGATGTGCACATGCCA------------------------------------------------------------------------------------------------------------------------------------------------------------------------------------------------------------------------------------------------------------------------------------------------------------------------------------------------------------------------------------------------

7429 Unigene24731_R-tanguticus Unigene9808_R-bungei

XXCTACCAAGACATTTTTTCGAGGTACACAGATCTCAGCAATATGAATCGAGGTTACTATGTGGAGAACTATACCATCTGGCCTATGGCAAACAACCCTCAAACTTATGCTTTCGAAAGCGAAAACCATGCCAATCAGTACCACAGAGGTGGCCGACCTGCTACGGTTAATCACAACAAGCACTATCCTCCTGTCATGGAAACAACTACTACTGAGGTTCTTTACCCCACAAACCTTCACGAGAAAAGGATGAGTAAGTTTCTGGGAACCAAAGATCACCGTAATTCTGCTGGAGATGGAAATCATGGCAAGTACCAAGATGGTCAGCGTGCAAAAGGTGATTATCTGGAATACGTTTCTCACCTAGAAACCATGCCCAGCATGGCCTTGGGAATGGTTGCTCCTCGAGTCCCTTACCAACACGGTCTGCAGGACAAAGAAGTGGTGTTTGAGGAGTACGTGACTACTGATGAGGAATATGACCCAGATATGAACGAAGTGATAAAGAGGAAGGTGACAACTAGGGTTGAGAAGAGAGCGGTGAAC

------XXACCAAGACATTCGAGGTACACAAATTTCAGCAATATGAATCGAGGTTACCATGTGGAGAACTACACCATCTGGCCTATCACAAACAACGCTCAAAGCTATGCTCTCCAAAGCGAAAACCATGCCAATCAGTACCACAATGGTGGTCGACCTGCTACGGTTAATCACAACAAACACTATCTTCCTGTCATGGAAACAACTACTACTGAGGTTCTTTACCCCACAAACCTTCATGAGAAAAAGACGAGTAACTTTCTAGGAATCAAAGATCACCGTAATTCTCCTGGAGATGGAAACTATGGAAAGTACCGAGATGGTCAGCGTACAAAAGGGGATTACCTGGAGTATGTTTCTCACCTAGAAACAATGCCCAGCATGGCCTTGGGAATGGTTGCTCCTCGAGTCCCTTACCAACATGGTATGCAGGACAAAGAAGTGGTGTTTGAGGAGTACGTGACTACTGATGAGGAATATGATCCAAACACGAATGAAGTGATAAAGAGGAAGGTAACAACTAGGGTTGAGAAGAGAGCGGTGAAC

9583 Unigene28808_R-tanguticus Unigene20429_R-bungei

CAACAACAACAAGCTCTAAACTGTCCTCGTTGCAATTCTGCCAACACCAAATTTTGTTACTACAATAACTACAGCTTATCGCAACCACGCCATTTCTGTAAGGCGTGCAAGAGGTATTGGACTCGAGGCGGAACTCTACGAAGTGTTCCTGTTGGTGGGGGGTGCCGGAAGAATAAGAAGCTTACTAAGAGACCAAATTCTGATTATCAGCTCATCTCAACTCTTTCGACTACCAAATCTACCGGTAATATGAATCCAATTTCCAATAATGCTGCTTATGATATTAATCAGTCTCAGTTCAATGCTTTCAAACCAGTTTTTCCGACCCTAGGAGCCAGTAGTTTTACAGGTCATGGTCTTACAAGTTTTCCACGTTTCGGAGATTCTCCTTCAAGCATCTTGAGTGGTTTAAAGGAAACTATAGATGATAATCCATGTCAAACATTGTTTCCTCTTCAAGGCCTACTAATGGCTAGTTGTGGAAGTGGAAGTAACAAGGAAGCAAAGTTTGGAGGAGGAAATGACCCAATGAACAATAACACGGTCATGGGAGCGCATGGCATTGAACAGCTTATTAATTCTTCATTTGATTCATCGGTCTATTGGAACTCCATTGGTGCTTGGCCAGATCCCTCAAACTATGGATCCTCGGAGACTTCTCTAATC

CAACAACAACAACCTCTAAACTGTCCTCGTTGCAGTTCTGCCAACACCAAGTTTTGTTACTACAACAACTACAGCTTATCGCAACCACGCCATTTCTGTAAGGCGTGTAAGAGGTATTGGACTCGAGGCGGAACTCTACGAAGTGTTCCTGTTGGTGGGGGATGCCGGAAGAATAAGAAGCCTACTAAGAGACCAAATTCGGATTATCATCTCACCTCAACTCTTTCGACTACCAAATCTACCGGTAATATGAACCCAACTTTCAATAATGCTGTGTATGATATTAATCAGTCCCAATTCAATGCTTTCAAACCAGTTTTTTCGTCTCTAGGCGCCAGTAGTTTTACAGGTCACGATATTACAAGTTTTCCTCGGTTAGGAGCTTCTCCTTCAAGCATCTTGAGTGGTTTAAAGGAAACTAAAGATGATAATCAATATCAAACATTGTCTCCTCTTCATGACCTACTAATGGCT------GGTTATGGAAGTAACAAGGAAGCAATGTTTGAAAGAGGAAATGACTCAATGAACAATAACATGGACATGGGATCGAATGGTATTGCACAGCTTATTAATTCTTCATTTGATACATCAATCTATTGGAACTCCATTGGTGCTTGGCCAGATCTCTCAAATCATGGATTGCCGGATACTTCCCTAATC

5257 Unigene19490_R-tanguticus Unigene30374_R-bungei

GTGGTTGTTACCATGAATCTAAGTTCATGCTTGTTTTTATTTCTGTTAATCAGTGGGCCGATATGGCAGACAAGTTACAGGGGAAGAGGATATGCTTCTGCACTAGCACCG---------ACAACTGAGAACAACTTCATCAATGGCGACTTCTCTAATGGCCTGTTTTACAGTGTGAAGGCAAATCGAGGTAGAAGAGTTAGGACACCAAGGAAACCTGCTTCACCTTTGCCAAATAGGGCTGGTCATATGTCTGTACCTCCAACACCACCCTTTCCTCCACCTTGCCCTCCGGTCATT---------CCGCCC

---------------------XGTTCATACTTGTTTTTTGTGTTGATAATCAGTGGGGCGATATGGCAGAATAGTTACAGAGGAAGAGGATATGCATCTGCACTCGCAGCGAGAGAGAAGACAACTGAGAACAACTACATCAATGGCGACTTCTCTAATGACCTGTTTAACAGTGTGAAGGTGAATCGAGGTAGAAGAGGTAGGAGATCAAGGAGACCTGCTTCACCTTTGCCTAATAGGGCTGGTCATATGTCTGTACCTCCAACACCACCCCTTCCTCCACCTTGTTTTCCTGTAGTTGACACCCCGCCCCCX

3353 CL8618.Contig2_R-tanguticus Unigene28963_R-bungei

CTAGATACTTCTATCAGGCTGCTTAGTTTGCGTAAGAAGTTGCCTACTTTTCAAAATATTTGCCTTGTGGTGAAAGCACTTACACAAAGAATGTTGTCCTGTAGAGATCTCGCACAGATTAAATATTTGCTTCCAGAAAGCATTTGTATAAAGAAGATCCTCATACATGACGCGAAAACCCTCTGCATGATGCCAGACATGAAAATTACATTTCTATTGGATGTTATAGAACCTAAACCCAATCAATCTGCGTTTATGGCCTTGCGCGAAGTTTTCCATCAGAGGCTTTTAGATTTCTTTAACCAGCATACAGAGAGTTCTGACATCCCAGAGGCCATGCTGCCAGAGCCATTTAGTCAAAGAGATAAATCTGTTCTCCCTGGGCCACTCGCTGAAACCTCACGTATTGAATCCCCTCATGCCGACTCTGAGTCATTATCTCTGTCGTCTTCCTTTGGGCAATCTATCAGAAAACACTTCTCTCAGAAGATAATTGTCCCTGAATCAGAAAAGACTGAACTACTAGCATCC------CCTCCAATTTCAGTAAACTTAGATGATGAAACCAGTTCAGTTTCTGACTCTGCTAGCATTACTCTCCCTGATGCCTTGATCTCCAGTTCACAGAACTCAAGTTGCAGTGCAAGTGAAAGCCCTCCTAAGAAGCATGCCCCAGTTTATCATCATACAGTGACTGTAGAAACACCAGTGCAGCCAACACCAGCAAAAAGATTGGCTGAGCTGAGCTCCTGTGAAAAGAAGGATAATGATAATAGGGTGATAGGTCTTACTGCTGCAAAGAGATCACTAACTTTTGACGACCGCCTTGAAACTACAGTAACAAAGAACACTGATGCAGATTATAAAATGGTACAAGAGACTACCAGCCACAAAATGGATCCAACTGATCTCGTGAATATAATTCAAAGCATATTTAAGTCGGCGGCTAGCGGCTCTTTAATCACCAAACAGGAGCTTTTGCAAAAGATAATAGCAAATAATTTGGATATTGTTGACATAAGAGAAGCTGAAGAACAGCTTAAGCTACTGGAAAAGGTGGCTCCTGATTGGATTTTCCAGAAGTTGGTACCTAGTGGTGATGTTTTGTATAGTATCAGAAAAGTCTCGGATTTGGAGTTGATTCGTGCAAAGCTG

CTAGATACCTCTATCAGGCTGCTTAGTTTGCGTAAGAAGTTGCCTACTTTTCAAAATATTTGCTTTGTGGTGAAAGCACTTACACAAAGAATTCTGTCCTGTGGAGATCTCGCACAGATAAAATATTTGCTTCCAGAAAGCATTTGTATAGAGAAGATCCTCATACAGGACGCGAAAACCCTCTGCATGATGCCAGACATGAAAATCACATTTCTATTGGATGTCATAGAACCTAAACCCAATCAATCTACGTTTATGGCCTTGCGCGAAATTTTCCATCAGAGGCTTTTAGATTTCTATAACCAGCATACTGAGAGTTCTGACATCCCAGAGGCCATGCTGCCAGAGCCATTTAGTCAAAGAAATCAATCTGTTCTCCCCGGGCCACTCGCCGAAATCTCACTTATTGAATCCTCTCATGCTGACTCTGAGCCATTATCACTGTCGTCCTCATTTGGGCAATCTATCAGAAAACACTTCTCTCAGAAGATAATTGTCCCTGAAACAGAAAAGACTGAGCTACTAGCATCCTCTGTATCTCCAATTTCAGTAAACTTAGATGATGAAACCAGTTCAGTTTCTGATTCTGCTACCATTACTCTCCCTGATGCCTTGATCTCCAATCCAGAAAACTCAAGTTCGAGTGCCAGTGACAGCCCTCCTAAGAAGCTTGCCCCAGTTTATCATCATACAGTGACTGTAGAAACACCAGTGCAGCCAACGCCAGCAAAAAGATTGGCTGAGCTGAGCTCCTGTGAAAAGAAGGATAATGATAATAGGGTGATAGGTCTTACTGCTGCAAAGAGATCACTGATTTTTGATGACAGTCTTGAAACTACAATAACAAAGAACATTGATGCAGATTACAAAATGGTACAAGAGACTAACAGCCACAAAATGGATCCAACTGATATCATGAATATAGTTCAAAGCATATTTAAGTCGGTCACTTGTGGCTCTTTAATCACCAAACAGGAGCTTTTGCAAAAGATAATAGCAAATAATTTGGATATTGTTGACATAAGAGAAGCTGAAGAACAGCTTGAGCTACTGGAAAAGGTGGCTCCTGATTGGATTTTCCAGAAGTTGCTACCGAGTGGTGATGTTTTGTATAGTGTCAGA------------------------------------

11106 Unigene7601_R-tanguticus Unigene36758_R-bungei

XXGGAGAAAGTAACTGAGGACACAAGCTGCGGAGAGATCAGAGATATTGCTGGTATCGCTGTCAAAGATTTCGCTCGCAGGATGAACCTGCCCCAACCGGAGGTGATGAAAGTGGTAGATGCAGAATATACTGCTGATTTTTCTTGTTTCATACTGTACAACCTCAATATCTTTATTAGATGCTTGGATCCACAGCAGGAGGATCCACAGCAGGATCCCCCCAAAAAGTATGAGGGTGTCATCCAAAGCATGGTGTGGAGGGGACTGATCTACGAGGAAACGGATATATTTCAGATACCAAGCATACTACCATTTGGGGACATGAGAGAAAGTTGCAAGAATCTCACGCGTCAGAGACCAGACTTTAAGGATTTTGCCTGGGACAAACTCTTGGATAAACTTGAAGATATGGGCTTATCGATCGATCACCCAACTAACACCTCGATGCCCGTCCTTGCATGGGAGTGTCAACCCCTGCATAGTAGATACATTGAAGGAGGAATTGAGGAGACAATTGGGCACTCCATCATTGTCCGTGTTAAACCTCCCAGAGGCAAAAAGTCCAAGCCTGGACTACACGGTTTGTGGAGACTTAACACCTCCGAGAGAAAATGGATTCAAACATTCAGCCCCGAACTGACAGATAGACTAAAAAAGGGTATACGTGAGGGGCAGGAAAAAACATATCCTCCTCGCATTGTTACACATGATGAAAAAACGGGAAGAGCTGTGTGTAACATTAAAGCTCTAGTACXX------------------------------------------------------------------------------------------------------------------------------------------------------------------------------------------------------------------------------------------------------------------------------

---------------------------XXTGGAGAGATCGCACAAGTTGCTGATATTGCTGTCAAAGATTTCGCTCTCAGGATGAATCTGCCTATACCGAAAGTGCTGCAAGTCTTACAAGCAGAATATACAGCCCATTTCGCTTGTTTCGTCTTGTACAACCTCAATGTCCTTTTGAAAGTCGTG---------------GATCCACAGCAAGATCCCCCAATGGACTGTCACACTGTCATCCAAAGCATGGTGTGGAGGGGAGTGATCTACGAGAAGACCGAGATATCTCCACTAGAGACGAGACTACCACCTACGGACATATCAGAAAATAGCAAAAATCTCACACGTCAGAGACCAGACTTGAAGGAATTTGCATTATACCAACTCTGGGGTAAACTTCAGGATATGGGTTTGTCGATTGATCACCCAACTAACACCTTGATGCCCATCCTTTTGTGGGCGTCTCACCCCCTGGATAAAAGAGAAGTTGGGGCAGGATTTGATGAGACAATTGAGCACACCATCATTGTTCGTGTTAAACCTCCCAGAGGCAAAAAATCTAAGCCTGGAATGCACGGTATGTGGAGAGTGACCACCTGCGAGAGAAGATGGATTCTTACGTTGAGGCCCGAGCTAACAGATAGACTCAAGAAAGGTGTCCGTGAGGCGCTGAAGAAAACATACCCTCCTCCTACTATTGAACATGACAAACAAACTGGAAGAGCTGTGCGTGTGGTTAAAGCTCTAGTCCATTTGGATCCCATGTGTTTGGAAACTCCTCGAATAGTCACCTTGGCGCTGTTTGCCTTGGATAATCACTATAGGAAAAAGGGACCAAAGCTCAAGTTCGAGAGAGTTGTAGATGTGATGTTGAGAACCGACTTTATGTACCTTCTTACCCTCGAAGCCTCTGACGAAGAGAATATTACTCACATCTTCATGACATCGGTGACAGTATGGTCAGACTCGGGACTCATAGAATCAACACCGCTTGTCAGACAGGAAAATGATTTGCCCAAGGXX

214 CL149.Contig11_R-tanguticus Unigene14689_R-bungei

CTTTTAATAGATGAGGAGGGAGACATAATGATGCACGATGTCGTCCGCGATGTGGCTATATCAATTGCAACGGAAGACCACGGGTTTATCGTGAAAGCTGGAAGGAATCTGAGTGAGTGGCCAGATATTGAGTTGGGTAATTGCAAGAGATTGTCAATGATGTATAACTCGATCAAGCAGCTTCCGGTATCTCCGATCAAAGCACCATACCTTCGAGCCCTGGTTTTGGGTCACAATTACAGTTTAACCGAACTCCCACCTGATGTTTTTGCTGATATGAAATGTCTAATGACACTTGATCTTGGAAGAACATCCATCCAATCCCTTCCCCCATCATTGTCCTTCCTTCAAAACCTCCGAACCCTTCTTTTAAACCAAACA---GCTCTGAGAAATTTATCCCCCATTGAGAAGTTAGAGAAACTAGAGATACTTAGTCTACGTGAGAATGAGGAAATCATCAATGAATTTCCAGAAGAGATGGGAAATTTGAGTAATCTTAAGGTTTTGGACCTTACAATGACTTCCTTTAATAAAAGCATCAACCCGAACCTAATTTCCAAGTTACACCGATTGGAAACATTACACTTG

---------------------------------------------------------------------------------------------------------------------TGGCCCGATATGGAGTTGGGTAATTGCAAGCGATTGTCGATGATGTATAACCCCATCAAGGGTCTTTCCACGATTCAAATCAAAGCCCCGTACCTCCAAGCCCTGTTTTTGAACGACAACACTTCTTTAACTCTACTCCCATCTGATTTTTTTGCTGAGAGGAGAAGTCTAATGACACTTGATCTTAATAGAACAGCTATAAAATCCCTTCCAACATCATTGTCCTGCCTTCAGAACCTCCGCACCCTTCTTTTAAATAAAACATATTCTCTAACGAATCTATCCCCCATTGAGAAGTTA------------------------------------------------------------------------------------------------------------------------------------------------------------------------------

8515 Unigene26725_R-tanguticus Unigene20489_R-bungei

TCCACTTATTCACCAGGTTCTCCAACCCCACCAAGATATCCATTTCCTGCAGTACCTATGCTGTTCAGAGATGATCCAGACTCTGATCGGTCAGGAAATCCAGTCGCTTTGCCTGGGATTGACAGTCCATCGCCCATCAGAGATTCTATACATGTGGACAGAACTGACAGAGAAGGCTCACTTTTTATTTCTACTGGTAAACCAGCAGACGTTAATGGGAATAGACCAAGTTTATCTGACATGCCTACATCGATTGCTAAAGAGAAGAACCATGCAGATAGTATTTCCTTAGATAATCTAGTGTCTGGTATCACATCTAATGTACTTGGGTCCCTAGAGGACAGCCGAACTAAAGTTAGTGACCACTTGGATCTTAGTTCGAATGAACATTATATGGCCGTGGAGGAAAAGGCTGAGGAGATGCAGCAACCAGAA---GTGGGCAAAGAGAATACCCGACAACAAAGTAACATGGATCCAGATAAGCTTGCCACAGATTCCGCTCATTCATACGATCTTGTAGATGAAAACTCAACTAAGTCTAGTAACCAAATGGATCAGTGTGTAAATGGATCTCACGAGGGCCTGATCAACGAGCTCGAAAAGACACACCAGCCTGAAACCATGGTGCATGACTTGCATGGAAGTGAAAATCATGTGGATTGCAGCTTAAATGGGTTTGATATTGACATGGATGACAAGGTAGAGGATGTGGTAGAACCTAGCTGTGAGAGATTCACTGAAGAAAATGCTAGTCAGTGTGAAAAGCTATGCCCTCCTCCTATAGACAAAGATCTTCCAAGTGCTAGCCCATCAGAAAATGAAGTTAGACTTCCTGAAGATAATAACGAGGTAATTCGGGTAGCATCTAGAGAATCATTGAATGTCGAGAAATCATCTTCACAGGTTGGTGGAAGGAAACAAGTTTCGAAAAGGAAAAGCCTTGCA---GTAGGAGAAGAAGAGTCATTGAACCTTGGAAGCCCAAGCCTCCCACAGAACCTTGTAAGAGAAAACCCCCCACAGAAAAAGGCAGCACACAACCCTGTAAGAAAAACACGGCAAGTGTCACGTAGGAAGAGCCTAATAGGCGCTGGTACTGAATGGAAAGCGGGTGTAAGAAGAAGCACTCGAATGCGAATGAAACCACTCGAATATTGGAAAGGGGAACGTCTCATTTACGGAAGAGTGCACGAAAGTCTGGTGACGGTGATTGGATGCGAGAAGTGTGGTTCTCCTTGTGGGAACAATGGAGGTCCTAAACTGAAG------------

---------------GGTTCTCCAACCCCACCAAGATATCCGTTTCCTGCAGTACCAATGATGTTCAGAGATGATACAGACTCAGATCAGCCAGGAAATCCAGTCTCTTCGCCTGGGATTGAGAGTCCATTACCCATCAAAGATTCTATACATGTGGACAGCACTGATAGAGAAGGCTTACTTGTTATTTCTACTGGTAAACCAGCCGATGATAATGGGAATGGACCAACTTTATCTGGCATGTCTACATCAACTGCTAAAGAGAAGAACCGTGCAGATAGTATTTCCTTAGATAATCTAGTGTTAGGTATCACCTCTAATGTACTTGGGTCTGTAGAGGACAGCTCAACTAAAGTTAGTGACTGCTTGGGTCTTAGTTTGAATGAACATTATATGGCCGCGGAGGAAAAGGCCGAGGAGATGCAGCAACCAGAAGTGGTGGGCAAAGAGAATACCCGTCAACAGAGTAACATGGATCCAGATAAGCTTGCCACAGATTCCGCACATGCACACAATCTTGTAGATGGAAACTCAACTAAGTCTAGTAACCAAATGGACCATTGTGTAAATGGATCTCATGAGGGCTTGACCGACAAGCTCGAAAAGACACACCAGCCCGAGACCACCGTGCACGACTTGCATGAAAATGAAGATTATGTGGACCACAGCTTAAATGGGTTTGATATTGACATGGATGACAAGGTTGAGGATGTTGTAGAACCTAGCTGTGAGAGATTCACTGAAGAAAATGCTAGTCAGTTTGAAAAGCTATGCCCTCGTCCTATAGACAAAGATCTTCCCAGTGCTGGCCCATCAGAAAATGAAGTCAGACTTCCTGAAGATAATAACGAGGTACCTGGGGAAGCGTCTAGAGAATCATTGAATGTCGAGAAATCGTCCTCAGAGGTTGGTGGAAGGAAACAAGTTTTGAAAAGGAAAAGCCTAGCAGTAGTAGGAGAAGAAGAGTCATCGAACTTTGGAAGCCCAAGCCTCCCACAGAACCATGTAAGAGAAAATCCCCCACACAAAAAGGCAGCACACAAACTTGTAATAGAAAAACAGCGAGTGTCACGTAGGAAAAGCTTAATAGGAGCTGGTACTGAATGGAAAGCGGGTGTAAGAAGAAGCACTCGAATGAGGATGAAACCACTCGAGTATTGGAAAGGGGAACGTCTCATTTACGGAAGAGTGCACGACAGTCTGGTGACGGTGATTGGATGCGAGAAGTGTGGTTCTCCTTGTGGGAACAATGGAGGTCCTAAACTCAAGGTTTTTAATTAC

965 CL3162.Contig3_R-tanguticus Unigene23815_R-bungei

ATGTACAAGACAAAGCTTCAAGAGATTTGTCACCAACGGCTATGGAGTTTACCGACTTACTCATCAATCAAAGATGGACCAGATCACTGCCCTCGATTCAGAGCTTCTGTAGTCGTTAATGGCATCTCCTTCGATTCTTCTGATTTCTGTAAATCTTCTAAAGAAGCACAAAATCAAGCTGCAAAAGTCGCTTTCCATCATTTCAATCTCAATGCTTCTCCAAATTCATCGGTTCCTTCTTCAATTGCTTCTTCGAGTGCAAGTAGTATAGAAACAGTGGTAGTAAAGAATGAGGTATCAAGTAACTGTATGGTTTATGGAAGCTCTTCAGGTGTCAGTGATGGTCGTGAACTTGCAGATGTAAACCACGTGTACAAGATTCAGCTACAAAACTATGCTCAGAAAAAAAGCCTCGGCTTACCTGTTTATTCTCATGTACGCGAGGGCTCACCTCATGCCCTTCGCTTTAAAGCTACAGTCAATGTAGATGGACGAACCTTTGAGAGCCCAGAATTCTTTCGCACACTAAAAGAAGCAGAGCATGCTGCTGCGAAGGCCGCCTTTTCCTCGTTGTCAACGAATGGTATTCAAGAGGATGATTGTAGTTTATACAAGAACCTTTTACAAGAGTTGGCTACAAAGGAAGGCTTTTCCATTCCAGTGTAT---ACAACCAAATCTGGCTCTTCTCAGGTTCCAACATTCACTTCTAGTGTAGTAGTCGAAGGTGATATTTTCCATGGGATAGAATCAAAAACCAAGAAGCAAGCCGAGAAAAATGCTGCAAAGGTTGCTTATATTTCACTTAGAGAGCGACAAATGAGTAGAATCCCTGCACAGAGCGGTGTACATGAAGCTTTTGAATTCATCTATCCCAACTCTTCAAACTCACAACCCAACATCGCTGCGGACTTTCAACTCAAACTCAATCTTGAAGGTCAATCTAGTAGAAAACCTCCGAGTAGCGAAGCACACGATGGTATTGAAATTATATATCCCGACTCTTCCAGCTCAAACTCCTCCATCATTAGCGACTTAGAACAAAAGCTGAAATTCAAAGATAAAGATGAGGACGTTGTTGATTCATCAGCTCAAATTATCACT---------------AAAGCAGAAGATCTCTCAACGGCATTGCAAAATGCATCGGGATCTTCATCGCAGGATGGATCGTCATCTCCTACGACAGTTTTGGAGTGTCTTGACCGTGCAGTGGCCGAACAGAATGCAAATCAAGAGACTAAAACAGAATGCACGTGGTTGTGTAACAGGGTTTTGGTTTATCCTCGTAAGCTGGATTTGAATCTTCCTATGGGTGTCACTATGTTGCCAATTAGCGATGACCAGTGGGTGGCTGTGAGT

ATGTACAAAACAAGGCTTCAAGAAATTTGTCACCAAAGGCAATGGAGTTTACCGACTTACTCATCAAACAGAGAAGGACCAGATCACTTTCCTCGATTCAGAGCTTCAGTAGTCGTTAATGGCGTCTCCTTTGATTCTTCTGATCTCTGTAAATCTTCTAAAGAAGCACAAAATGAAGCTGCAAAATTCGCTTTCCAACATTTCTCTCTCAATACATCTTTGAAT---------CCTTCTTCAATTCCTTCTTCGAATGGGAGTAATGGAGAAACAGTGGTAGTAAAGAATGAAGAATTAAGTAATTGTACAGTTTATAGAAACAATTCAGGTGTCACTGATGCTCGTGAATTTGCAGATGTAAACTACATGTACAAGAGCCAGCTACAAAACTATGCTCAGAAAAAAAGCCTCAGCTTACCTGTTTATTCTTGTGTACACGAGGGCACACCTCATGCTCCTCGCTATAAAGCTACAGTCAATGTAGACGGACGAACTTTTGAGAGCCCAGAATTCTTTCGCACAATAAAAGAAGCAGAGCATGCTGCTGCAAGGGCTGCCCTTTCCTCATTGTCAACGAATGGTATTCAAGAGGATGATTGTGGTTTATACAAGAACCTTTTACAAGACTTGGCTAAAAAGGAGGACTTCTCCATTCCAGTTTATACAACCACCAATTCTGGCATTTCTCATATTCCAGCATTCATTTCTAGTGTAGAAGTCGAAGGTGATACGTTCCATGGGATAGAATCAAAAACCAAGAAGCAAGCCGAGCTAAATGCTGCGAAGGTTGCTTATATTTCCCTTAAAGAGCGTCAAATGAGTAGAATCCCTGCTCAGAGTGGTGTACATGAAGCTTTTGAATGCATCTATCCCAACTCTTCAAGCTCACAACCAAACATCACTGCAGACTTCCAACCCGAACTTAATCTTAAAGGTCAATCTAGTAAAAAACCTCCGAGTAGCGAAGCGCACAGTGGTATTGAAATTATATATCCAGACTCTTCCAGCTCAAACTCCTCCATCGTTATCGATTTAGAACGAATGCTGAATCACAAAGATAAAGATGAGAATGTTGTTGATTCATCAGCTCAAATTATCAGTAAAACAGTAGAAGTCAAAGCAGAAGATCATTCAACGGCATCGCGAAATGCATCAGGATCTTCATCACAGGATGGATCGTCATCTCCTACGACAGTTTTGGAGTGTCTGGAACGTGCAGTGACCGAACAGAATGCAAATCAAAAGACTAAGACAGAATCCACATGGTTGTGTAATAGGGTTTTGGTTTATCCTCGTAAACCAGATTTGAATCTTCCTAAGGGTGTCACTATGTTGCCAATTAGCGATGACCAGTGGGTGGCTGTGAGT

1687 CL4746.Contig2_R-tanguticus CL3781.Contig1_R-bungei

---------------------------------------------------------------------------------------------CTGTTGAGGGTGGTGACTAGTGATAAACGAACACCACTACACATTGCTGTGCAATATAAGCATCTTGAGATTGTGGAACTTTTGGTCGAAGCCGCCCCTGATCTTGAGTATTCTGCTAATGCTACTGGCAAAACCCCACTTTTAATGGCTTTCGAAGAAGCATTATCAGGAGATCAGAATACAGCTGAAATCAGAAAGCTTCTAATATGGAAACAACCAATGCAATGTAGACTACCTACAGGCGAAAACAATTGGACTTTAATACATCATGCTACACACAAGGGAGACTTAAGTGCAGTTAAAGAAATCATTCACTTCTGGCCAGACTGTTATGAGTTGGTCGACAAGGAAGGCCAAAATATCCTTCACCTTGCTGTTAAGTTTGAGCAAGTTAACATAGTGAAATATGTTTTGGAACTGAGAAATGTAGCTGACAATGTTTTGAATAAGAAAGACAACTATGGTGATACTCCGTTGCACCTTGCTACAGAAAGTGCTAATCCAAGCATAGCTTTAACTCTGCTTGATGATCCCAGAGTCAGCAAGACGGTCGAGAACATGCAAGGAAAAAAAGCCATGGATAAGGCGTCGAGCGTATTATTGGGAGAGATGATAAAAGGGATGTATCCAATCCAATGTGCAATCAAAGGAGATGTGGACTTCTTTAGAGCAATTCACGTTGATATTCTTCGAGAGTCCAAAGATGAAGATGGTGAGTCTGTTCTTTCTATTGCTGCGAGGAAGGGTCACTTGAATTGTTGTGAAGTGATTTCTAGAAAGTGTCCGGAGCTCTTATACCAAACAACCAAAAACGAAATCACCATACTTCATACAGCAGCAGCTAACGGAAAAGATGATATCGTCAAATTTCTCCTT

AATGATTCAAAGAATACGGCGCTGCACTCTGCTGCCCTTGGAGGCCACCTTAAGTCAGCAAAAGTATTGATCGAAGCTGATCGCAATAAGAAGCTATTGGGAATGGTAGCTCGTGAGCAAAGTACAGCACTGCACTTTGCTGTAAAAAATCACCATCTTGAGATTGTGGAACTGTTGGTCGAAACTTACCCTGATTTTGAGTATTCTGCTAATAATGAAGGCAAAACCCCACTTTTTATGGCTTGGGAAGAAGCTTTATATGGTGACCAGAAAACAGCGGATATCAGAAAGTTTTTAATAAGGAAACAACCAACTCAATGTCAACTACCTATAGGCGACAACAACTGGACATTAATACATCATGCTACACGCAAGGGAGACTTAAGTGCAGTTAAAGAAATCATTCACTTCTGGCCAGATTGTTGTCAGTTGGTCGACAAGGAAGGCCAAAATTTCCTTCACCTTGCTGTTAAGTTTGAGCAAGTTGACATACTGAAGTATATTTTGGAACTAAGAAATATAGCTGACAATGTTTTGAATGGGAAAGACAACTACGGTAACACTCCCTTGCACCTTGCCACAACAAGTGCAAATCAAACCATAGCTTTAGTTTTGCTTGATGATCCCAGAATCAGCAAGATGGTAGAGAACAAGCAAGGAAAAAAACCTTTGGATATTGTGTCAAGCGTGTTATTGGGAGAGTTGATAAATGGGGTGTATCCAATCCAATGTGCAAGCAAAGGAGATGTGGAC------------------------------------------------------------------------------------------------------------------------------------------------------------------------------------------------------------------------

6011 Unigene28948_R-bungei Unigene21692_R-tanguticus

CTCGTTTATTCTCTCTATAACCTAATTGTCACGGTGATCATGAAATCCGCCTTTGGATTTATTACCTTCAACATTATTCTGCTCGTCGTTCTAGTTGGAAGCTTTCGACCCTCAGAGAGATCAAAATATTTCAGCCTAGGATCAAAACATTACAATCCTTTCCTTGAAGAAGAATTGGAAGTGACTGAAAGGTATGAAAAGAAGAAAGTTGGTGGTCTTGATTTTAAAGATGACCATTATGCTTTTTCTTATGTTTATGAAGGATACGAAACAGATGATGAAGATGATGACGAATATGAATATGTTTATCGTAGTTCTGACGACGAAAGTTGCACGAGGGACGATGATACCGAGGAAAAAGAATTAATTCTTGGTAGTTCTGAAGAGGGTAGCAGAAATATTGAAGTTGTTATTTGTAAAAAAGAAAGTCCTTTGGGTACTTCTGTTACTGGTAAATGTTTTGAAGATTATGATTTTTTCAAGATAAGGTGCGAAGATCACATTTCTAGGCAACATAATAGATGGAAGGAAGAATGGTTGAAAGAAAAT---------

------------------------------------------------XXCTTTGGGTTTATTACCTTCAATATTATTTTGGTTGTCGTTCTAGTTGGAAGCTTTCGACCCTCAGAGGGATCAAAATATTTCAATCTGGGATCAAAACATTTCAATCCTTTCCCTGAAGCAGAATTGGAAGTGGTTGAAAGGCATGAAAAGAAGAAAGTTAATGATCTTGATATTAAAGATGGTCATTATGCATTTCCTTATGTTTACGAAGGATATGAAACAGACGACGAAGATGATGATGAATATGAATATGTTTATCGTAGTTCTGACGATGAAACTTGCACGAGTGACGATGATATTGATGAAAAAGAATACATTCTTGGTAGTTCTGATGAGGGTAGCGTGAATAATGAAGTTGTTATCTATGAAAAAGAAAATTCTTTGGATACTGGTGTAAAT---------TATGAAGATTATGATATTCTCAAGAAAAAGTGTGAAGATCTCATTTCTAGGATGCATAACGAATGGAAGGCAGAATGGTTGCAAGAGAATGATACTAGC

6116 Unigene13705_R-bungei Unigene21947_R-tanguticus

GAACAAGCAGATCCTTGGGACGATTCTGCTCTCCTCAACGCTTTCACTCACGCAATCACCAAATACAAGCAAATGCACAAGAAAGAAGCATCTCATAATCATAATCATAGCGGTGACCAAGTTTCTTCGATTACAATA---------AGTGCGAAGACTCTGGAGAATTCTAATGATCCTTCACCTACAAAAGAAATTCCGCAAACTGACCTTCACCCCCAAGAAGTCTATACAGAGACATCAGATGTTCCTCCTATGCAAGAAGTACTTGAGGGATATCAAAATTCACAAGGAGTTGATACATACAACAATTTACTGAATCAGTATTACGAGCTGGAGGAGCAGAGGCAAAATGTTTTACAACAGCTTCAACAAACAGGGTATTGGAATAACCAGTATGCTACTGAAGCTTTAGGTCAAGAAATCCGGGACTTGAACCAACAAGCATTCCATCCAATGACAACCTCGTATTGTTGCCCGTATGTCTGTCCTTGCTTGGCAACTCCATGTTCTTCAATTCCTTCTTGTTCGTTGGGCGGAGCTTCAGTTAGTAAAAAGGATATTGAGTCCACTTCTATTGCATGCACACAAGGCTCTCAAAAGTTATGCTCTCGCGAAGATGACAGTATTGTTAAGACCGCAATGGGAGCTGCAGAACTAGCAATGTCGTCGATAAATAAGGTGCATGAAGGTTCATCAGAGGGGAAAGTG---------ATCGGCCCAGAGACTGACATCTCGGTAGTTCTGAATGCTTGGTATTCAGCTGGGTTCTACACTGGCAAGTATCTTACAGAGCAGTCCAATGCAAAAGAAAGACGC

GAACAAGCAGATCCTTGGGATGATTCTGCTCTCCTCAACGCTTTCACTCACGCAATCACCAAATACAAGAAAATGCACAAGAAAGAGCCATCTCATAATCTCAACACAAACGGAGGAGAAACTCATACCGAAGAAATTACAATGAGCAGTGCAAAGATTGTGGAAAGTGCTAATGATTCTTCACCTACAGAAGAAATTCAGCAAACAGACCTTCAACCCCAAGAAGTCTATACAGAGACATCAAATGTTCCTCCTATGCAAGAAGTAGTTGAGGGATACCCAAAGTCACAAGGAGTTGATACATACAACAATTTACTGAATCAGTATTACGAGCTGGAGGAGCAGAGGCAAAATGTTCTACAACAGCTTCAGCAAACAGGGTATTGGAATAACCAGTATGCCACTGAAGCTTTAGGTCAAGAAATCCAGGACTTGAACCAACAAGCATCCCATCCAACGACAACCTCATATTGTTGCCCATATGTCTGCCCTTGCTTGGCAACTCCATGTTCTTCAATTCCTTCTTGTTCCTTGGACGGAGCTTCAGTTAGTAAAAATGATATTGAGTCAACTTCTATTGCATGCACACAAGGCTCTCAAAAGTTATGCTCTGGCGAAGATGATAGTATTGTTAAGACTGCAATGAGAGCTGCAGAACGAGCAATATCATCCATAAATAACGTGCATGAAGGTTCATCAGAGGGGAAAGTGAAGCAAGGTATCACCCCAGAGACTGACATCTCGGTAGTTCTGAATGCTTGGTATTCAGCTGGGTTCTACACTGGCAAGTATCTTACAGAGCAGTCCAATGCAAAAGAAAGACGC

3530 CL9001.Contig2_R-tanguticus CL5882.Contig2_R-bungei

CCAGATATGGATGACATAAGGTGGAAAACCCAGCTCCAAAATTGTTATCGCAAGAAGATCTTCACCGAAATACTGGATACATCAATGAGACACCTTCCCCCTAGGAGCCCTGCAGAATTTGTTGAAGCTACAGAATCTGTTATAAGAATTGAGGAAAATATTTATACTACTGCCACTACCCAGTCGGATTACTTACACAGGATTTCACTCAAGATGCTTTCAATGGAAGTGAACTCAGAAAATAGT------

CCAGACATGGATGACGCAAGATGGCAAAACCAGTTCCAAGTTGATTTTCGACAGAGGATTGTCTGCAATATATTGGATACTTCAATGAAACATCTTCCTATTCGGGGTCCTGCAGAATTTCTTGAAGCTACGGAAGTTGCTGTAAGATTTGAGGAAACTGTTTTTACTACTGCCACTAACGAGCCGGATTATCTACACAAAATTTCATTGAAGATTTTGTCAATGGAAGTGAATTCAGAAAATAGTAATGCA

4223 Unigene1420_R-tanguticus Unigene27862_R-bungei

ATGGCTCATCACCCTTGCCTTTCATGTCTTGTATCCTTTCTAGTTCCCATGATCACATTTTCTCTCATGAGCAGTGATGTTAGCGCTGCTCGCCAACTACTGGAGATGACCTTGCCTGAGATTCCAGAGCTCCCCAAGCCTGAGTTGCCAACTCTACCTGAAATCCCAACTCTTCCAAAACCAGAACTTCCACCTATGCCTAAAGTTGAAGTACCAAAGTTGCCTGAAGTCCCGACTTTGCCAAAACTTGAACTGCCACCACTCCCCAACACTGAAGTACCAAAACTTCCTGAAGTTCCAACTTCTCCAAAACCCGAATTATCACCATTGCCCAAGATTGAAGTTCCAAAATTCCCGGAAGTCCCAACTTTACCGAAAATCGATGTACCAAAGCTACCCGAACTTCCTCCTTTTCCCAAACCAACTTTGCCCACTATTCCTACCCTTCCCAAGGACATAATACCCATCCCAACAGAAAACCCT

ATGGCTCATCACCCTCGCCTTTCATGTGTTGCACCCTTTCTAGTTCTCATGATCACATTTTCTCTCATGAGCTGTGATGTTAATGCCGCTCGCCAACTACTGGAGACGACCTTGCCTGCGATACCAGAGATCCCCAAGCCTGAGTTGCCAACCCTACCTGACATCCCAATACTGCCAAAACCAGAACTTCCACCTATGCCTAAAGTCGAAGTACCCAAGTTGCCTGAAGTCCCGACATTTCCGAAACCTGAATCGCCACCACTCCCC---ACTGAAGTGGCAAAACTTCCCGAAGTTCCAACTTTTCCAAAACCCGAATTACCACCACTGCCTAAGGTTGAAGTTCCGGAATTCCCTGAAGTCCCAACTTTACCAAAAGTTGATGTACCAAAGATACCTGAACTTCCTTCTCTTCCCAGGCCAACTTTGCCAACCATCCCTACCCTTCCCAAGGAC---ATTCCCATCCCAACAGAAAACCCT

6000 Unigene21655_R-tanguticus Unigene17726_R-bungei

CGGGTATTAGGCAGCCATCGATCGGAAAGCTCTTCTGCTGTAGATGACATAACTTATCAGGTGGATCCGTTTGAATCTTCATCTGACTGTTCATCTTGGGAAAGTTCTGAAGAGCAAAACGATAATGAAAAGGCCATCAAGACTAATGACAGTGTAGTTAAATCTGAAATAAGATCTTCATCTGCTTCAGGTTATTTCAAAACCATTTCCAATGCAAACACCTTGTTAGATCCATCTCAATGTCAAAAGCAATCCACCCTGGAATGTGTTTCATGTACAAACATTGAAGCAATTGATCCCATAGAGAAAAATGGTGATCACCCCGAGGAAACAAATGGCAGCCATCCTTCTGGACATTTTAGATCAGTAAATGCAACATGGTGTAGATTCTCAGAAAATAGATATGATGATGATTACAATCATGTGCATTCATGGCCACTAGGGGGACTATATAAAAACCCTTTCTCCAATGACAGAGGACTTTCGTGTGACTCAAAGTCACTTTTCATTAATTCAAACCTGCAGGCGCCTGACATATCTGAGGAAGTGCTTGTGGGGAAAGTTTCAATGTTGGGGGAACAATCTATTTCTGATGATTCTTTCCCTCATCAAACCAGAGAGGACCTTGATTTCAGAAACCATACTTTATCCACTTCACATATTGCTACTTCCTGGAAGCTCAAGTACAATTGCAAGGTTTTTACTATGAACCCCATG---------------------------------------------------------------------------------------------------------------------------------------------------------------

------------------------------------------GATGACATAACTTATCAGGTGGATCCGTTTGAATCTTCATCTGACGGTTCATCTTGGGATAGTTCTGAGGAACAAAACGATAATGAAAAGTACATCAAAACTAATGACAGTGTTGTCAAATCTGAAATAAGATTTTCATCTGCTTCAGGTTATTTTAAAACCATTTCCAACGCAAACACCTTGGTAGATCCATCTCAATGTGGAAAGCGATCCACCCTGGAACGTGTTTCATGTAGAAACGTCGAAGCAATGGATGCCATAGAGAAAAATGGTGACCACCCCGAGGAAACAAATGGTAGTCATCCTTCTGGGGATATTAGATCAGTAACTGCAACATGGTGTAGATTCTCAGAAAATAGATATGATGACGATAACAATCATATTCATTCATGGCCACTAGGGGGACTATGTAAAAACCCTTTCTCCAATGGCAGAGGACTTTCATGTGACTCTAAGTCACCCTTCATAAACTCAAACCTGGAGGTGCCTGACATATCTGAGGAAGTGAGCACGGGGAAAGTTTCAATGTTGGCGGAACAATCTATTTCTGATGATTCTTTCCCTCATCAAACCAGAGTGGAGCTTGATTCCAGAAGCCATGCTTTATCCACTTCACATATTGCTACTTCCTGGAAGCTCCAGTACAGTTGTAAGGTTTTTACTATGAACCCCATGCTAACAAAAAATGTTTGGTGTCACTCAATAGACAATTCAAGAGAGAGATCTAGTGTGGACTATAAACTATCATGTCTTTCATACTTTGACTTTTCATCAGTAGAGGATCCTCGGATGGTATTCTCGGGGAAGTTGGTTGATGGTCCTGAACGTGGATTT

5413 CL2066.Contig1_R-bungei Unigene20051_R-tanguticus

------------------------------------------ACATCTACATCGAGAGGAGAAGCAGGAAAGATTTAT------TACAATCACACCGATTCGTGCAATTTATCAAGATGGACGACAAAAGAATGTCACTCATTCATGTACAATGGTAGAGCTTGGCAACAAGTTTCTGATTTCTATTCCAGTTTAGTAAACGGAAGATATTCACTACCTCTCCTACTATTCCAATTCCAAAAACAGAGGCCTTTGAACACGGAGCCAAGAGTTAAT---------AATGAAGCTATGGAATCTGAGTTGGAAGGCGTCTCTATGGAAAGTAGAAGCGGC

CCATTTCAAATTCGCAGAATTTCAACAACAATGGCTACAGAAGAACAAAACCCTAATTCTAATTCAGCAAAGATTTATTCTCATTACAATCACATCGATTCTTGCAATTTCTCCAGATGGACGGCTAAAGAATGTTATGAATTTATGCACGAT---AGACCGTGGAAATATGTTGGCGATTTCTATTCCAATTTAGTTAATGGAAGATATTCACTACCTCTACTGTTA------TTCCATAAAGACAGGCCTTTGTACACAGAGCCAAGAATTGATGAAGCTATGGAATCTGCTGAGGAATCTGAGCTGACATGCGTCTCTATGGAA------------

2464 CL7396.Contig2_R-bungei CL6582.Contig3_R-tanguticus

ACGACGAGGACTCCCTTCAGCGATCTTAGCCAGGTCGATGCAGACCTTGTGCTTGCCCGGACTTTACAGGAACAGGAAAGGGCGTATATGATGTTGAGAATCAATGGGGAAGGAGGAGGGAGTATTAGTGACTATGGAGGAAGCTCTGATGCTGGAAGTTTTCTCCAAGAAGATGAAGATGAAGATGATGATGAAGATTTTCATGATGATGATTCCAACGACGATGCCTTTGATGCTCAACATGAAAATTTAGAAAACCCCCATCATTCTGATGAAGCTTTTGCCAGAGCTCTTCAGGACGCCGAAGACCGCGAATTGGCTGCACGTATGATGGCTCTTACTGCTGCCACCTGGGATCAACAACACTCTGACGACGACGATAACGACACCAACTCTCAAGATACATGGGAGGAGGTAGATCCAGATGAATTATCATATGAGGAACTACTTGCCCTGGGTGATGTTGTTGGAACTGAAAGCAGAGGACTTTCGGCAGATACGATTGCTTCTTTGCCTTCAATGAGCTATAAATCACAAGGCAATGAGGATAGCAGCACCGACCAATGCGTTATTTGTCGGTTGGAGTATGAGGATGAAGACACCCTGACCGTTCTTTCATGCAAGCATATGTACCACTCCGAGTGCATAAATAACTGGCTTCATATCAACAAGGTGTGCCCTATCTGCAGTGCCGAAGTGTCCACTTCT

---------------------------GGGACTATTAGTAGTGACTAT------------------------------------------------------------GAAGGAGGAGGAAGCTCTGATGAT---------------GAAGCTGGAAGTTATCTCCATGATGATGAAAATGAA------------------TTTGATGATGATTCCAACGATGATGCATTTGATGTC---CATGAGAATTTAGAAAACAACCTCGACTCCGATGAAGCTTTTGCTAGAGCTCTTCAGGACGCTGAACACCAACAATTGGCTGCACGTATGATGGCTCTTACTGCTGCCACTTGGGATGAAGACCACTCTGACGAC---------GACACCAACTCTCAAGATACATGGGAGGAGGTAGATCCAGATGAATTATCATATGAGGAATTAATTGCACTGGGTGATGTTGTTGGAACTGAAAGCAGAGGACTTTCGGCAGATACGATTGCTTCTTTGCCTTCAATAAACTATAAATCACAAAGCAATGAGGATAGCAGCACCGACCAATGCGTTATTTGTCGGTTGGAGTATGAGGATGAAGACACCCTGACTGTTCTTTCATGCAAGCATATGTACCACTCCGAGTGCATAAATAACTGGCTTCATATCAACAAGGTGTGCCCTATCTGCAGTGCTGAAGTGTCCACTTCT

8201 Unigene26183_R-tanguticus Unigene22712_R-bungei

AAAAGTCAGCTTGCAGATTTACATAGCGTCGAGGTCTCAAAGAATATGGAAACAGAGAAGCAGCTTAAATTTTTTCAAGGCTGTGTAGCTTCTGCATTTGCGGAGCGGGATAACGCGTTGATGGAGGCCGAGAAGGCTAAAGAAACGGAAGGTCTTATGTTACAGGAAGTGAATACTTTGCGACGAAGGATAGAGGAGATGACTTCAAATTTCCTTGAAGATAAGAAATCACTTACCTCACTGCATATTGAACTGTCGAAACTGAAAGAACAGAACGAAAATTTTCAGAAGGTTATCACCAAGTTTTATGATATACGACAAAGCTTTTCGAAGGTTGATGAGGACCCATGTTTGGATGATAAATGTGAATATCTTTTGCAAGATACTCCAGAAACATGGAGTTTCAATAGTAATACGGAAAATCATGCTTCTGAGTATATTACTGCACTGAAAAAGGAGCTGGAAACATTGAGAACCTCTGTGGATAGACATCAAGACAAAATGCGCATGGGGTTGGAAATTGAAAATCACCTGAAAAAGAGGGTCCACGAATTGGAAAAGGAGAAGATTGATTTTGATGATATGATCAAGAGTAAAATATCCAAGTTGCAACATTGTCAAACCCAGCACAGAAGCGAAGTCATTGACATACTTCAGCACGAGAAGGCACAGTTCAAAACAATGCTTGATGTGCTACCTGAAATGATAAAACGATTTCACATGAACCCGACGCGAGATTATGACCATTCAGAAGGAGATGCAGAAACTGTTGATATTGAATGTCGCGATGTGCATATAAATACTGATCTTGATGTGAACTTGGCGTCAGAGGAGAAGACTACATCTTCACCAATTGCCATTCATGAAGGAAACGCTGGTACTTCTGAGGCCCTTGTGCAGGCATTGCAAGAAAAGAATACTCATCCGCTCCCACCAATCAAAACGTCAGAAGAAACATGTGATGACTCTGATGTTTTTGCACAGGCATTGAAAGAGAAGGTTTCGGCTCTTTTGCTACTTTCACAGCAGGAAGAAAGGCATTTATTGGAGAGGAACGTAAATGCAACTCTATTTAAAAAATTGGAGGAGTTGCAAAGGAACTTGCTACAGGTGACAAATGAGAAGGTGAAGGCTCTCATGGAGCTGGCACAACTAAGGCAGGATTACCAACTTCTCCAAGAAAACTCAAAACAGGCAACCTTAGTATCTGAAATTAGCGAAAAAGACATTGTGGCACTGGCTGATCATGGAAAAGACGGGAAACTTAAAAATATGCTCAAGAAAACGTATCTTGGACATTGGATGAGTCGAACCGATTATGGAGGAGCTGATGCCCACCTCGACTTTGCAAGGCTGAAGATTGAAAACGCAACCCTCAAAGAAAGCATTGAAAGCATGGAGCATTTAACATCCTGTGTCAATAGACTTCGCCGCTCACTGTCAAAGGCGAAAGATTCTTCAAGTCTTGAGAATATTATTACAGAAGCAACACATGTAAAGACGGCCCTTGGGAGTTCACTTCCTGTGAGCTGGTCAGCTGAAGCCGACGACAGTGAGCCGTTTGAGCCAGGTAAAGAAAAGGTGGACTCGGTTTCGGCAGCTGGGTTTGAGATGGTGGAGCTTGTAATTCTAGCTGCTCATATTCTGAAAGAGAGAATAGTCGACAAGGCTGCAAGC

------------------------------------------------------------------------------------------------------------------------------------------------------------------CAGGAAGTGAATACTTTGCGAGAAAGGATGGAGGAAATGAATACAAATTTCCTTGAAGAAAAGAGATCACATGCCTCCCTGCATATTGAACTGTCGAAACTGAAAAAACAGAACAACAATTTTCAGAAGGTTATCACCAAGTTTTATGATATACGACAAAGCTTTTTGGAGGTTGATGAGGACCCATGTTTGGATGATAAATGCGAATATCTTTTGCAAGATACTCCAGAAACATGGAGTTTCAATAGTAATACGAAAAATCATGCTTCTGAGTATATTACTGCACTGGAAAAGGAGCTGGAAGCATTGAGAACCTCTGTGGATAGACACCAAAACAAAATGCGCATGGGGTTGGAAATTGAAAATCACCTGAAAAAGAGGGTCCACGAGTTGGAAAAGGAGAAGATTGATTTTAATGGTATGGTCAAGAGAAAAATATCCAAGTTGCATCATTGTCAAACCCAGCACAGAAGTGAAGTCATTGACATACTTCAGCATGAGAAGGCACAGTTCGAAACAATGCTTGATGTGCTACCTCAAATGGTAAAACAATTTCACATGAACCTGACGCGAGATTTTGGCAGTTCAGAAGGAGATGCAGAAACTGTTGATATTGAATGTCGTGATGTGCATATAAATACTGATCTTGATGTGAACATGGTATCAGAGGAGAATACTTCATCTTCACCAGTTGCCATTCATGAAGGAAACGCTGGTACTTCTGAGGCCCTCGTGCAGGCATTGCAAGAAAAGAATACTCATCCGCTCCCACCGATCATCACATCAGAAGAAACATGTGATAACGCAGATGCCTTTGCACAGGCATTGAAAGAGAAGGTTTCTGCTCTCTTGCTACTTTCACAGCAGGAAGAAAGGCATTTATTGGAGAGGAACGTAAATGCATCTCTATATAAAAAATTGGAGGAGTTGCAAAGGAACTTACTACAGGTGACAAATGAGAAGGTGAAGGCTCTCATGGAACTGGCACAACTAAGGCAGGATTACCAACTTCTCCAAGAAAACACAAAACAGGGAACCTTGTTGTCTGAAATTACCGAAAAAGACATTGTGGCACTGGCTGATCAAGGAAAAGACGGGAAACTTAAAAGTATGCTCAAGAAAACTTATCTTGGACATTGGATGAGTCGAACCGATTATGGAGGAGCTGATGCCCACCTCGACTTTGCAAGGCTGAAGATTGAAAACGCAACCCTCAAAGAAAGCATTGAAAGCATGGAGCATTTAACATCCTCTGTCAATAGACTTCGCCGCTCACTGTCAAAGGCGAAAGATTCTTCAAGCCTTGAGAACATTATTACGGAGGCAACACAAGTAAAGACGGCCCTTGGGAGTTCACTTCCTGTGAGCTGGTCAGCTGAAGCCGACGACAGTGAGCCATTTGAGCCGGCTAAAGAAAAAGTGGACTCGGTTTCGGCAGCTGGGTTCGAGATGGTGGAGCTTGTGATTCTAGCTGCTCATATTCTCAGAGAGAGAATAGTCGACAAGGTTGCAAGC

2825 CL7373.Contig1_R-tanguticus Unigene22543_R-bungei

GCAGCAGCAGATGGCAAAGACATTGCAGAAGGAACAGTGACTCTTGGAGAACCTATGTCTACCTCTTCTGATGTTAGACCTACTGAGAGCTTCGACAAAAACTCTAGTGACACTGGCGTTTCTGACAATTACGCTGTTACAAATGCTACTGGCACAGTTGCGAGCTTAGCTGCATCCACATCTCTTCCTACTCCGGTATTCAATGTATCGTCTGTTTCGAGTAAACCAACTGGTGCTCCATTCAGCTTTTCTTCTAGAGGTGTTGACAAGGCTACCCTGCTTCCATTATCATCGGCATCTGTTGTGACTGAGTCCATTGGCCTGAATGGGCAGCAGTCAGAAGCGAAGGTTGAGGTTGTGAGCAGAGAGAGCGGCGAAAATGTAAAGGCTGATGATGAATCTTTATCCAAGACAGTACCAGCCTCCCTTGGAATATTTTCCTTTCATGCTTCAAACAATTCAACTCTGAATAACGAGTCAAATACTTCATCTTCTCTACTCACAGCCACTACTGATAAACCCAAAAGCAATGGTCTATTTGCACCCCTCAGCAGCATTACGACAACCACATCTGTTACTGCTCCTAACGTAACCCCAAGCACCCCTAGTTTTATATTTGGAGCTTCAGCTGCGCCAGCAAGTGGGACAGTGAGCACTCCAGCATTTACTTGGAACTTGCCGACACAGTCTACCACATCGTCACAAACAGCTGGGTTAACCGGGCCGATTTCCTCCAGCACCAGTGGATCTATTTTTGGTTCTTCATATATGCCTTCCCAAGCAAGTTCCAGTACTCAGTCGGCTGCTCCATCATCTGTTTTTGGGACCTGTTTCTCCAACACAGGTTTCAACTTTGGATCTTCTTCAAGTGCACAAATACCCTCATCCTTAGGCAGCTCTTCACCTTCCATGTTCTCATTCACTGCAACTGGTACTCCCACTCCAACTCCACCGCCCCTTTGCAATATTAACGATGAGATGAGCGTTGAGAAACCATCCAGTTCATCTCAACCATCTCCATTTGTATTTGGT---------------------------------------------------------------------------------------------------------------------------------------------------------------------------

GCAGTAGCAGATGGCAAGGACATTGCGGAAGGAGCAGTGACTCTTGGACAACCTATGTCTACCACTTCTGATGTTAGCCCTTCAGAGAGCTTCTATAAAAACTCCAGTGACTATGGCGTTTCTGACAATTACACTGTTACAAATGCTACTAGCACAGTTGCGAGCTTAGCTGCATCCACATCTCTGCCTTCT---------CATGTATCGTCTGTTTCAAGTAAATCGACCGGTGCCCCGTTCATCTTTTCTTCCAAAGGTGTTGACAAAGCTACCCTGCTTCCATTATCATCGGCATCTGTTGTGACCGAGTCCATGGGCCTGAATGGGAAGCAGTCAGAAGAGAAAATTGAGGTTGTGAGCAGAGAGGGTGGTGATAATGTAGAGGCTGATGGTGAATCTTCATCCAAGACAGTACCAGTCTCCCTTGGAATATTTTCCTTTCAAGCTTCAAACAATTCAACTCTGAATAACGAGTCAAATACTTCCTCTCCTCTACTCACAGCCACTACTGATAAATCCAAGAGCAATGGTCTATTTGCACCCCTCTGCAGCATTACGACAACCACATCTGTTACGGCTCCTAACGTAACCACAAGCACCCCTAGTTTTATATTTGGAGCTTCAGCTACGTCAGCAACTGGGACAGTGAGCGCTCCAGCATTTTCTTGGAACTTGCCGACACAGTCTACCACATCATCACAAACAGCTGGGGTAACTGGGCCGATTTCCTCCAGCACTAGTGGATCTATTTTTGGTTCTTCATATATCCCCTCCCAAGCGAGTCCCAGTACACAGTTGGCTGCCCCATCATCTGTATTTGGAACCTGTTTCTCCAACACAGGTTTCAATTTTGGATCTTCTTTGAGTGCACAAATGCGCTCATCCTTGGGCAGCTCTTCACCTTCCATGTTCACATTCACTTCAACTGCTACTCCCACTCCAATTCCACTGCCCCCTGTCAATAATAATGTTGAAAGGAGCATTGAAGAACCATCTACTTCATCTCAACCATCTCCATTTGTATTTGGCGGACCTTCATCATCAGCAGGAGGATCCAGTATGTTTCAGTTTAGCACCCAACAGAATCCATTCTCTGCAGGAGCAGGAGTATTCACAATTGGAGCAGGTGGCGGCGGTGGTGGGGACAATGACAAATCCAAGCGAAGAATTGTGAAAATTAATAGGAATAAATCGAAGAAA

9096 Unigene27873_R-tanguticus Unigene27567_R-bungei

CCCAAGTCAATTGCATCGAAGAAGAAAACTAAGGAAACAACTGTTGAGGAAACAAGTAAAGATGTAGAAGAACAAACTATTCCACCACCAGCAGTGAAGAAAAACAAATCGGTTGCAGCGAAGAAAACTAAGGAGACAGTAGTGCTGGCGGATGAAGGGAAAGATGTAGAAGGAGCAATAGCAAAGAGAAAGGGTAAGCCTGCGGCGAAAAGGACTATACCAGAGGAGAAAAAGAAGGTTGTAGCTGCAGAGATGGAAGAGGAAGAGGAGTCTGAGTCAGGACCAGATCCATCTGATGAAAGCGCTGAAGAAGATGAG---------GAGGAGGTGAATTCGAGTTGTAAGACTATTGTGGTTGAATACTGCAAACAGTGCCAGCAGTTTAAGAAAAGGGCTGTCATGGTGAAGGAAGGCTTGGAGAAAGCATTCCCAAACATCAAATTGCTTCTTAACCCAGATAAGCCTAGAAGGGGATGCTTTGAGGTCCGAGTGGAAGGCGGCGAAGTCTACATCAGTCTCCTGGATATGAAGCGACCATTTCAAAAGATGAAGGAGCTCGACATGGAAAAGGTCGTTGCCGACATTGTCAAGAAGGTT

------------------------------------------------------------------ACCACCGATGTCGTCGTCCCACCAGCAGCAAAGAAAACTAAACAGGCTGCAGCGAAGAAGAAGAAGAAGGAGACAACTGTGGAGGAAGATGGAATGAAA---GATGCAAAAGTAGCAAAGACAAAGGCTAAGCCAGCAGGGAAAAGGACTATACCAGAAGAGAAAAAGAAAGTTGTAGTTGCAGAGATA---------GAGGAACCTAGTTCAGGACCAGATCCATCTGATGAAAGCGCTGAATCAGATAATGCTTCTTCAAAGGAAACATCTTCCAATTCCAAGACCATTGTGGTTGAATACTGCAAACAGTGCCAGCAGTTTAAGAAAAGGGCTTTTATGGTGAAGGAAGGCTTGGAGAAAGCATTCCCGAACATCAAATTGCTTCTCAACCCAGATAAGCCTAGAAGGGGATGCTTTGAGGTTCGTGTGGAAGGTGGCGAAGTGTACATCAGTCTCCTGGATATGAAGCGACCATTTCAAAAGATGAAGGATCTCGACATGGAAAAGGTCGTTGCAGACATTGTCAAGAAGGTT

4766 CL377.Contig2_R-bungei Unigene17245_R-tanguticus

TTGATGGGTTTACTTAGATTTTTGCGTTGCCTGTTATGTTGCTATGAAGAGGAAGAAAACAGAGGTGTACATAGGTACCAAACTATTCCAGATTTTGTACAGACACCCAATCATCAAATTCAATGGAAATATACAGCCCCATCCCCATCTACTTATATTAGTACTTTAGGACAGAGTTCAACACCAGTTTCCAATTACTCTAAGAATGTGGCTTCCACTAAGCAGCAATCAATCAGAACATCACCCTTTCAATCTGCCATGAAATCTCCCCATTTGGCGAATGTAATATCATCGTCAATAGGGTCTCCTGCATTTATTAGCACCTCAACTACAACGATATCGAAAGCAAAATGTTTAGCAAAGAGTCCAGTTTCAACTTCCAATTATACCAATATCAACAAGACACCGTTGATCGAACGTAGCACGGAATCCCCCCAATCGGTGAAAATATCATTGCCTAAGCCCCAAATAGACCGTTACTCACAAAAGAGAGTAGCAACCCTTCTTGCAGCGTCACCAGACGTAATCCCTTCCGCAAAGAGTTCAATTCTATCTTTTGATAATGCCAGTTCAGATGGCACCCAGATTTCCATAAAGAATGTTGCATCATCTAGTAATAAGAAGAAAAAGAAAGCATCATTGACTGAACCTAAAGGAAAATCCCCCCAGCCGGTGCAAAAGTCAGTACACAAGCCTCAAGTTACGAACGAACGAAATTCTTCTTTAACTCCATCCACTTCTACCTCACTACTATCTTCATTCAAACCACCTCCTCTCACTCCGGTAGAAACTTGTACATCTCCGTCTTCCCAAAATACACCCAAAAAAAGCAATTATGTTTCGGGGCCAGAGGCTACCACTCCTCTTTATTCAGTTCCCGAAGACTTCAAGGAATTGATCAAGAACGACATTGTACCTCAAGTTCTACAGAGATATTTGACTCCCGCAAATTACAAAAACTTCTTTGAGGCTTTGATGTATGCTGAGGACTACTACCATGAGAAATGGAGTAAGTACCTCTTAAAAGATGTCAAGCTAGAGTTGCATGAGGCAGCAATATACACAAAACCAAGAATGGGCACAAACATGAATAGAAATCGAAAGAACAAACACACGAATGAGAATGAGACGAAGGACAAGGTTTTTGTAGCCTTCAGAATTGATTCTATTCGTGAGAGACGACCTTTTCTCCTGTCAAGAGACTATGTAAATTTACGGCCTTCAGGAAAGGGAAGGGAAGTCGAACACTTCCAGGGGGTTCTTTTCCGTGTGGTACAGAGAAATCTCGTGTTAGCAGAGTTTGGAGATGATTTTTATCTGCAGCATTCTTCAAGCCGCCAATATGATGTTAGTTTCTCATTTAACAGGGTGTGCTTAAAACGATCTTACCAAGCACTGACAGCCACAACCGACGCATTGCTTCACAACTTTCTCTTCCCGGGCCAAATGCCTAGAGTCAAT------GACCAACCTTATTTCACACCCTACCTTCATGATCTGGACAGAAAACAGTTTTCTGCAGTCAAAAATATATTGGGGCTAAACAGCTCTCCCCCTTTTCTTGTCGAAGGTCCACTGGTACTTATTAAGCGGGGAAATGCAATGAAAAAATCAGAAACGTATTTTGTCATCCAAGAAGCCGTTCAACAAATCTATCGAAGCTCTCCAGGTAGCCGGATCCTAATAAATGCACCTCGAAATATTATCTGTGATTGGATGATGACGAGCTTGATGTCTGAAATCCCCGAATCTGATATTTTCCGAGCGAATGCTGCATTTCGAGAGAAAGACGAAGTTGATGGTTTTATACTTCCTTTGTGCCCATTCGAAGGAGAGTGTTTCACATGTCCACCTCTCAAGGAACTCCAGGAGTTCAGGGTTGTATTAACAACATATATGAGCAGTTTCAGGCTTCATAATCAAGGCATAAGTACAGGCCATTTTAGTCATATATTTCTAGTTGATGCTTCGTCAATAATGGAGCCTGAGATGATTGTGTCACTCTCTAATCTGGCCGATGAGAAGACAGTAGTTGTCGTCACTGGTGCTTCTGGAAACTGTTCGGGTTGGGTTCGATCTGATATGGGGCAAAAATATGGATTGAAAGTGTCATACTTTGAGAGGCTTATGAAAAGACAGCCCTACTGTACTCTTGATCCATTGTTTGTTGCACGTATTATAGATAACGAGGACAAT

---------------------------------------------------------------------------------------------------------------------------------------------------------------------------------------------------------------------------------------------------------------------------------------------------------------------------------------------------------------------------------------------------------------------------------------------------------------------------------------------------------------------------------------------------------------------------------------------------------------------------------------------------------------------------------------------------------------------------------------------------------------------------------------------------------------------------------------------------------------------------------------------------------------------------------------------------------------------------------------------------------------------------------------------------------------------------------------------------------------------------------------------AAGAACAAAAACATGAATGAGAATGGGATGAAGGACAAGATCTTTGTAGCCTTCCGAATTGATTCTATTCCTGAGAGACGGCCTTTTCTCCTGTCAAGAGACTATGTACATTTACGACCTTCAGGAAAG------GAAGTCGAACCATTTCAGGGGGTTCTATACCGTGTGGTAGAGAGAAATCTCGTGTTAGCAGAGTTTGGAGATGATTTTTATCTGCAGCATTCTTCAACCCGCCAATACGATGTTAGTTTCTCATTTAACAGAGTGTGCTTAAAACGATCTTACCAAGCACTATCAGCCACAACCGACACATTGCTTCACAACTTTCTCTTCCCTGGCCAAACACCTAGAGTGAACATGCTTGACCCACCTTATTTCACCCCCTACCTTCATAATCTAGGCAAAGAACAGTCATCTGCAGTCAAAAATATATTGAGGCTAAACAACTCTCCACCTTTTCTTGTCGAAGGCCCACTCGTACTTGTTAATCAGGGAAATGTAATTAAAAAATCAGCAACGTATTATGTTATCCAAGAAGTCGTTCAACAAATTTATCGAAGCTCTCCAGGTAGCCGGATCCTAATAAATGCA------------------------------------------------------------------------------------------------------------------------------------------------------------------------------------------------------------------------------------------------------------------------------------------------------------------------------------------------------------------------------------------------------------------------------------------------------------------------------------------------------------------------

7605 Unigene25099_R-tanguticus CL2993.Contig2_R-bungei

XXGGAGAGCGCTCTAGCAAGAGTCATTAGTTTGTCAAGTGATCTTGCAAAAAGAAAGAATGAAATGAAAGTTCAAAAGAAAAGGTATAAGAGGCTGGAAACCCGATTGGCCAAGTTAAAAAACGAAACTTCAGATGTGATTACTGATACACAGGTCTTGGCAAGTGTAGACAAACATGGGAAGAGAGATAAAAGTAAACCAGAGAAAGTTCAAGGTGACACCAATGGAGTGTTAAATGGTGCAAGTTTATCCAAGTTGAAAATCAAACTGAAGCCTTTGAGTTGTGAAGTTGTTGACAGTGTCCGTGACTGTGTGGAGGCTGATTCTACCTGTCCTTCTGAGACCAAACCTAGCGGTGGTACTGCAGTTACGTCTGAATTTCCTTCAACACTCAAACGAAAAAGACGACGACGTTACATTTCTAGATGTGACGGTGAGGGTGATGGTCAAGTTCGAATTTGCAAAGCAAAGGTGGAACATACCAAAACAACACCCATTGAACAATGTGAGGGAACACCGAAGACTGCCGTCCCCTTAACACTTTTCGCTACGAGCGGGGATAAGTGTAAAAAAGTAAAAAGCCCGCCTTCTGAAAATGTCAAAGATAGAGCAAAGTCCTCCGAGAGGCAATCAAAACAGAGCCCGATGTCTGAAAGTGCCAAAGAGAAAGCAAAGCTAGCTGAGAGGCAATCAAAACGGACAGTTCATCCTCCAAAGCGGCTAAATCTT

------------------------------------------------------------------------------------------------------------------------------------------------------------------------------------------XATAAAATTAAACCTGAGAAAGTTCAAAGTGACGCCAATAGAGCGTTGAATGGTGCAAGTGTATCCAAGTTGAAAATCAAACTGAAGCCTTTGAGTTGTGAA------------GTTGGTGACTGTGTGGAGGCTGATTCTAACTGTCCTTCTGAGACCAAACCTAGAAGTGGTGCTGAAGTTACTTCTGAATTTCCTTCAACTCACAAACGGAAAAGACAACGACGACATATTTCTAGCTGTGTCGGTAAGGGTGGCGATCAAATTCGAAGTTGCAAAGCAAAGGTGGAACATACCGAAACAACACCCACTGAACAATGTGAGGGAAAACCGAAGACTGACGGACCCTTGAAACTTTTCGCTACGAGTGGGGATGAGGGTAAAAAGGTAGAGAGCCAGGCTTCTGAAAATGCTAAAGATAGAGCGAAGTCCTCTGAGAGGCAATCAAAACAGAGTCCAACATCTGAAAGTGCCAAAGAGAAAGCAAATCTAGCAGAGAGGCAATCAAAACGGACAGTTCATCCTCCAAAGCGGCTAAATCTT

2599 CL6864.Contig1_R-tanguticus CL1818.Contig1_R-bungei

AAAGAGGCAGAAACAGAGAAAATATATGCAGGTGAAAAGGTTGAAACTATAGAAGAGTTGAAACAGAGTGATTTAATTGATGAAATAAACGATCAAGATTTCATCGATATCGGAGAAAACACTAGTCTTGAACAGGAGCAATTCACAGAGGATATCAAAGAAACAAGACTAGAAAATGAGGGAGAAGGAGAACTTGCGAATCAAAACGTTGAAGTTGTGAAAGTAGAAGAAACGGCAAAAAAAATTGTAGGTATAATAAAGAAACCAAAGGCGGCATTAAAGAAAAAAGCTACTCAGCCTTCTTCAAAAACTGATAAAAAGGTGATGGCTAAAGAGAAACCAAGCGGCTCTTTTCAGAATTCAAAATTGTCAAAATTACCCAGCTTCAACACCAAGGTCGATACTCAAAACGAGAATGGGGCAAAGAAAATATCAGAGCTGAAGCGTGGAATGAGTAATGCTTCAAGATCCACAGTTGTCAGAAATAGCACTGGCACCATTGAGAAGGCTCAAACGAAGGATATACACAGTAAGAATACACAAGCAAAGACAAAGGAGGGAGAGGAGACACAACCAAATAAGGTAAAGAAAAGTAATTCTTTCAAGGCTTCACCATTACCCAGCTTTTATCTTAGGAAGGATCCAACTACAAAACCTGAGCCGAAGAAGGACGGAGAGGAGGCAAAGCTAGATAAGATCAAGAAAAGTAACACTTTCAAGGCTTCACCACTCCCGAGCTTTTATCATAGAAAGGATCCTCCTCCAGTAGTGCCTGAGTCGAAGAAGGTCACCACAACACCTCCTAAAACTTCAGAACTTGGACATCAAAGCAACTTGTTTGCTGAAGGGATAAAAATCGACAAAGAGGACAGAAATAATAAGACTGCTTCAAGAACAATCAGCACCACTGCAAAAGAGACCATACACAAACTACTAGAAGGTGCTTGGGAGGATCCAAGCACTCAAAAGACGAAG

AAAGAGGCAGAAACAGAGCAAATATATGCAGGTGAAAAGGTTGAAACTATAGAAGAGATGAAACAAAGTGATTTAGTTGATGAAATAAATGATCAAGATTTCATCAGTATCGGAGAAAACACTAGTCTTGAACAGGAGGATTTCACAGAGGATATCAAGGAAACAAGACTAGAAAATGAGGGAGAAGAAAAACTCGCAAATCAAAATGTTGAAGTTGTGAAAATAGAAGAAACGGCCAAAAAACATGCAGGTATGATAAAGAAACCAAAGGCGGTACTAAAGAAAACAGCTATTCAGCCTTCTTCACAAACTGATAAAAAGGTGGTGACGAAAGAGAAACCCAGCGGCTCTTTGCAGAATTCAAAATTGTCAAAATTACCCAGCTTCAACTCCAAAGTCGATCCTCAAAACGTGAATGGGGCAAAGAAAATACCAGAGCAGAGGCGTGGAATGAGTAATGCTACAAAGTTCACAGTTGTCAGAAATAGCACTAGCACCATCGAGAAGGCGCAAGCAAAGGATATACACAGTAAGAATACACAAGCAAAGGCTAAGGAGGGAGAGGAAACACAACCAAATAAGGTAAAGAAAAGTAATACTTTCAAGGCTTCACCATTACCCAGCTTTTATCTTAGGAAGGCTCCAACTACAAAACCTGAGCCAAAGAAGGATGGAGAGGAGGCAAAGCCAGATAAGATTAAGAAAAGTAACACTTTCAAGGCTTCACCACTCCCGAGTTTTTATCATAGAAAGGATCCTCCTCTGATAGTGCCTGAGTCAAAGAAGATCACCCCAACACATCCTAAATCTTCAGAACTTGGACATCAAAGCAACTTGTTCGCTGAAGGGATAAAAATCGACACAGAGGACAGAAATAACAAGACTGCTTCAAGAACAATTAGCACCACTGCAAAAGAGACCATACACAAACTACTAGAAGGTGCTTGGGAGGATCCAAGCACTCAAAGAGCAAAG

4167 Unigene5984_R-bungei Unigene1384_R-tanguticus

CTTTCTGGTGCTAGTGCGAAGTCCAGTTTTATGAATCAAAATGATGGATTCGGAAGCCAAGGTGAGGCATCAGGAGCACCAACAGAGAGTGGTGGGTGGGGTTCTGCCCCTTTGACTACTGAAAGGAGTACTTGGGGCGATTTATCGGCATCAGACAATTTGTTTGGCTCCTCTTTGACAACCCTCTTCAGTTCTACCACTGCGCCTGAAAAAGAGGAAGAAGATGACCCTTGGGGTACCAAAGCAACAGCTACAAAGAGCACAAGCAGCTGGGGCAATGCAACATTATTTGGTGAAGATTCTGGAAAAGCTGGAGGAGATGACTCTTGGGGGACCAAAGTAACTTCTAAGAAACCAATCGATAGCTGGGGCAATGCAACAGGAGGAGACCCCGAAGGAGGAGGTGACCCTTGGGGAACCAAAGCATCGACTTCCAAAGGAGTTACAGAGAGCTGGGGCAATGCTACAGGTCGAGTCCTTGAAAAAGACGGAGGAGATGACTCTTGGGGAAGCAAAGCGACGACCTCTAAAGAAGCTACAAACAGTTGGGGCGGTGGCATAGGCTCTGCTCTAGAAGAACCTGCAAAAGGCCAAGTTGAAGACTCGGACCCTTGGGGAAGCAAACAAACTCCTAAAAAACCGACAGAATCATCATGGGGCATCCCAAACACCGACGACAAAGGGAAAGATGTAGCAGAAAAAGTCGAAAGTGGATGGGGAAGTGCAGCAATTGTGCAAAACAATGACACCGGAAAAATTGTGGCTGGAAATGATTGGGGAGCAGGTGTTGGAAATAATGGTCAATCAAACCAAGATGATTCATGGGGCAAAGCTACACAAAGTTGGAAGGTGAAAGATGATTCAAGCAATGTGAACTCAGAATGGGGCAAGCCAAATGATGGGGCAACAAATACTGGTTGGAACAGCCAGAAAAGTAGTGACGGTGACCGAGGATTCAACGGGAATAAAAGAGGTATGGACAACCAACTGGATAGCTTGAATAAATCAAGGGATTTTGATGGGGGACGGGGTTCAGGTGGAAGAAGAGGTAGGGGAGGATTCAGAGGAAATGGAGACCAGCCTGGTGGTGGTAGAGGCAGAGGTTTTGGGAGGGGTCGTTCTTCTAATTGGAATAGTGGAAGTCGGGACAATGAAAAGACCAGTAATGATTTTGGGGGCCAAGGATCTACCTGGGGAAATTCCTCACAAGGTGCAGAAGAAAAAATGAGTCAAGGGGGTGGTTGGGATAACAAAGCAAGTAGCTGGAAAAGTGGGGTAGCCGGTGCAGATGCGAAAAAATCTGCATGGGTGGACGCAAGTGGGGACCAAGGGGCTGGTTGGGGGAATGCTTCACAGGATGATGGGGGAAAGGCAAGTCAAGGAGATGGCTGGGGTAGCAGATCCAGCAGTGGTGCAACTGATGCAGGTGGGAACACCTCAAGTGGCTGGGGTCGGGCAAACAAGTCTGATACTAATCAGGCCTCTGGGTGGGGCAGTGGGGCTGCTGATTTGGGTGCAAACACGCCATCTGGTTGGGGCAGTGAAAGCATTTTAGGTACTCCGTCGAAAAAATCAGATACTAATCAGGCCTCTGGTTGGGGCAGTGGAACAGCTGATTGGGGAGCTAAC---AAGTCAACAGGCTGGGGAAGTGGAGATGCTTCTGGAGACAATGGTGGTGGAAAATCTTATGGCGGGGGAAGGACATCAGGAGGATGGGGTGGAGGAGGACGTGACAGTGACGGGGGTGGTAGAGGTTTTGGCCGAGGAAATAGAGGCAGAGGTCGTGATGGTGATTCCTCAGATTTTTCTAATCGGGGTAGAGGTGGTTTTGGACGCGGTGGA

---------------------------------------------------------------------------------------------------------------------------------------------------------------------------------------------------------------------------------------------------------------------------------------------------------------------------------------------------------------------------------------------------------------------------------------------------------------------------------------------------------------------------------------------------------------------------------------------------------------------------------------------------------------------------------------------------------------------------------------------------------------------------------------------------------------------------------------------------------------------------------------------------------------------------------------------------------------------------------------------------------------------------------------------------------------------------------------------------------GGAAATGGAGACCAGTCTGGTGGTGGTAGAGGCAGAGGTTTTGGGAGGGGTCGTTCTTCTGATTGGAATGGAGGAAGTCGGGACAATGAAAAGACCGGTAATGATTTTGGGGGCCAAGGATCTACCTGGGGAAATTCTTCACAAGGTGCAGGAGAAAATACAAGTCAAGGAGGTGGTTGGGATAACAAAGCAAGTAGCTGGAAAAGTGGGGCAGCCGACGCAGATGCGAAAATATCTGCATGGGGTGACGCAAGTGGAGACCTAGGGGCTGGTTGGGGTAATACTTCACATGATGATGGAGGAAAGGCAAGTAAAGGAGATGGATGGGGTAGTAAATCAAGCAGTGGTGCAACTGATTCAGGTGGGAACACCTCAAGTGGCTGGGGTGGGGCAAACAAGTCCGATACTAATCATGCCTCCGGTTGGGGCAGTGGAGCTACTGATTTGGGTGCAAGCAAGTCATCTGGTTGGGGCAGTGAAAGCATTTTAGGTACTCCACCAAAAAAATCAGATACTAATCAGGCCTCTGGTTGGGGTAGTGGAGCCGCTGATCCGAGAGCAAATATTAAGTCATCTGGTTGG---------------------------GGCGGCGGAGTTGCTGATTCAGGTGCAAATAAGTCATCTACTTGGGGCAGTGGAGCCGCTGATTCG---GGTGCAAATAAG------------------------------------------------------------------------------------------

7605 Unigene25099_R-tanguticus CL2993.Contig2_R-bungei

XXGGAGAGCGCTCTAGCAAGAGTCATTAGTTTGTCAAGTGATCTTGCAAAAAGAAAGAATGAAATGAAAGTTCAAAAGAAAAGGTATAAGAGGCTGGAAACCCGATTGGCCAAGTTAAAAAACGAAACTTCAGATGTGATTACTGATACACAGGTCTTGGCAAGTGTAGACAAACATGGGAAGAGAGATAAAAGTAAACCAGAGAAAGTTCAAGGTGACACCAATGGAGTGTTAAATGGTGCAAGTTTATCCAAGTTGAAAATCAAACTGAAGCCTTTGAGTTGTGAAGTTGTTGACAGTGTCCGTGACTGTGTGGAGGCTGATTCTACCTGTCCTTCTGAGACCAAACCTAGCGGTGGTACTGCAGTTACGTCTGAATTTCCTTCAACACTCAAACGAAAAAGACGACGACGTTACATTTCTAGATGTGACGGTGAGGGTGATGGTCAAGTTCGAATTTGCAAAGCAAAGGTGGAACATACCAAAACAACACCCATTGAACAATGTGAGGGAACACCGAAGACTGCCGTCCCCTTAACACTTTTCGCTACGAGCGGGGATAAGTGTAAAAAAGTAAAAAGCCCGCCTTCTGAAAATGTCAAAGATAGAGCAAAGTCCTCCGAGAGGCAATCAAAACAGAGCCCGATGTCTGAAAGTGCCAAAGAGAAAGCAAAGCTAGCTGAGAGGCAATCAAAACGGACAGTTCATCCTCCAAAGCGGCTAAATCTT

------------------------------------------------------------------------------------------------------------------------------------------------------------------------------------------XATAAAATTAAACCTGAGAAAGTTCAAAGTGACGCCAATAGAGCGTTGAATGGTGCAAGTGTATCCAAGTTGAAAATCAAACTGAAGCCTTTGAGTTGTGAA------------GTTGGTGACTGTGTGGAGGCTGATTCTAACTGTCCTTCTGAGACCAAACCTAGAAGTGGTGCTGAAGTTACTTCTGAATTTCCTTCAACTCACAAACGGAAAAGACAACGACGACATATTTCTAGCTGTGTCGGTAAGGGTGGCGATCAAATTCGAAGTTGCAAAGCAAAGGTGGAACATACCGAAACAACACCCACTGAACAATGTGAGGGAAAACCGAAGACTGACGGACCCTTGAAACTTTTCGCTACGAGTGGGGATGAGGGTAAAAAGGTAGAGAGCCAGGCTTCTGAAAATGCTAAAGATAGAGCGAAGTCCTCTGAGAGGCAATCAAAACAGAGTCCAACATCTGAAAGTGCCAAAGAGAAAGCAAATCTAGCAGAGAGGCAATCAAAACGGACAGTTCATCCTCCAAAGCGGCTAAATCTT

**Sub-file 3. BBH_ML_chi-tan.cds_Selected**

137 CL1302.Contig1_R-tanguticus Unigene31257_R-chinensis

GAGTGTATTAAATCTTACCATCCACACTGTGTGGATAAGGATAAAGAATTTTTAGATTCAGGAAAGCGCTGGGTTTGTGGTTGGCACCAATGTTTCATCTGTAGAAAAACTGCAACACTACATTGCTTTGGCTGCACGGATGCTGTATGCAAGAAATGCATTAGCTCTTCAGAGTTTGTGTGTGTTAAAGGGAACAAGGGTTTCTGTGCTAACTGCTTAAACCTTGCTCTACTCGCTACACAAAATGTGGATGTTGATTCTGATGGGGTTAAGATAGACTTCAAGGATAAAAATACATATGAGTGTTTATTCAAGGAATATTGGGAGATACTAAACGAAAAAGAAGGATTAACTTTGGAAGATCTCCGTTCGGCTGAGGTTCGGTTGAAGAAGGGGGTGTGGTATAAAGACGAGTCTGATTCTGAAGAAGGTGACAAAGACGATCAAGACTTCGACGACAATATGGATGGCAATGCGGAAATTGATGCAGTTCCAATAACAGGAAAGGATCTGAAAGTGTCTACTCCTAGGATGCGAAGGAGAAAGTCGAAATCCAAAAAGAGGGAGTTTGATGGATGGGGATCAAAGGTACTTAGGGATTTCCTAAAATCTATTGGTAAGGATTCAAGTACTCCGATTTCTCAAAATGAAGTGGGTGAGATCATCGATAAGTATATACGTGACAATAAGCTGATCGATTCAGACAGAAAGAAGAAGGTTAATTTCGATGCAAATCTCGTGTCTGTTTTTGGCAAGAAAACTGTGAACCGTCACAAAATCAATGAACTTCTGGAAAGTCATTTTGCTGAGAACCTAGAGGAATCGGAAAATGATGAGTTGAGTTATAGTTCAGATGAAAAGGACAATGGCACCGATATTGCTTGCAAAAGGCAGAGGACTAGCTTAAGCAGGAAAACTGAAGAAAAGGAAAAGGTCTTGGAAACTCCTCGAAGCCCTTTTGCATCTATTACAACTAACAACATCAAGCTGGTTTATTTGAAAAGAAGTTTAGTTGAAGATCTTTGCAAAAGCCCAGGAACTTTCGATGATAAAGTGATGGGAACATTTGTGAGGGTCAAATCTGATCCAAATGACTACTTCCAGAAGAATGAGTATCAGCTACTGCAAGTTACAGGAATCACAAAGGTCCAGGGAACCAACAACGTCAATGAGAATGTCACCCTGCATGTTTCCAGCATGAGGAAAGGCTTTCAAATTCACATGCTGTCAGATGAGGACTTCTCCGAGGAAGAAGTTGAAGATCTGCGTAAGAGAGTTAAAGATGGTTTGCTTAAGAAGCTGATGACTGTAGAGCTTGAGGGAAAGGCCAGAAGTCTCCATGAGGATTTAACTAAACATTGGATCAATAAAGAGCTTATCCGCCTAAAATACCTCAGTGACCGAGCAAATGAAAAGGGATGGCGTAGAGAGCTATATGAATACTTGGAGCGCATGAAGTTGCTCAAGACAGAAGCTGAACAGCTGCGTCTTTTAAAAGAGATTCCTAAGGTAGCTGCTGAGGAGCTAGAGCTGGAAATCGAATCCCCAGAACTACCTACAGATACAAGTCCAGTGAAAGTTTACTCGCCTAGATCAATTCTTCTCCGTTCTGATTCTATACCCGATGCTATACCAGGCCCTAATGATGCTATTTGTGTTATCGGAGACGACACCGATAAAAGAGCTATTGAGAATATATCTGGAGGAATTGGAAATGGTACTATAGTAAATACCAGTATGGCAGTTGAGCAAGATTGGGTAGTTGTGCAAAATTCAGTGGGGACAGAAGTGTTTGATCATGATGCTATTGAAAAGGCTACTACATTAGCAGTAGGTGTGCAAGATTGGGTAGTGGTGCAAGATTCGGCGGGGACAGAAGTGATTGAAGATGATACTTTTGAAAAGGCTTCTGAATTAGCCGTAGGTTATATTGCTGATGTTACATTGGATGCTCAAAACCAACGTGAAACTGATGATATGGAAAAGATTCGAGAAACTAGAGGTGCAATTGAGGCTCAAAACCAATTCGACATCTCGATGGAGGGTAGATCAAGCAATAGTTCTTCACTAATGTCAAATTTGGATACCGAACGA---------GGAAATGAGGGTAAG---------------------------GAGATAATTGATTTGGACAGTGACGACGAAGAGGGTTTGGGC---------------------------------------CGAGTGGACATGGAGCCACCAGATAAGATCTGGTATTATATGGATCCCGATGGCATCACACAAGGCCCATTTGACTTGTCACAACTAAAATGTTGGCAGGAAGGGGGCTATTTCGAACCAAGTTTCGAGGTTTGGAAGGTAGGGCAGTCTAGGGAACAAGGAAGATTGTTAACTCATGTTCTTCAGTCGATGTTT
[truncated: 1,837,544 more chars]
